# Dacentrurine stegosaurs (Dinosauria): A new specimen of *Miragaia longicollum* from the Late Jurassic of Portugal resolves taxonomical validity and shows the occurrence of the clade in North America

**DOI:** 10.1371/journal.pone.0224263

**Published:** 2019-11-13

**Authors:** Francisco Costa, Octávio Mateus

**Affiliations:** 1 GeoBioTec, Faculdade de Ciências e Tecnologia, Universidade Nova de Lisboa, Caparica, Portugal; 2 Museu da Lourinhã, Lourinhã, Portugal; State Museum of Natural History, GERMANY

## Abstract

The stegosaur species *Miragaia longicollum* was erected based on a partial anterior skeleton from the Upper Jurassic of Portugal. Until then, almost all stegosaur specimens in Portugal and Spain had been identified as *Dacentrurus armatus*, the sister taxon of *M*. *longicollum* and only other member of the clade Dacentrurinae. The holotypes of the two species have little overlap, since the holotype of *D*. *armatus* is mostly a posterior skeleton, so the classification of other specimens to either species is unclear and the validity of *M*. *longicollum* has been questioned and debated. Here we describe a largely complete specimen of *M*. *longicollum* discovered in 1959 in Atouguia da Baleia, Peniche, Portugal, consisting of both anterior and posterior portions of the skeleton. Comparisons to the holotypes of dacentrurines and other stegosaurs shed light on the convoluted relationships of this group. We conclude that *M*. *longicollum* is valid and rather different from *D*. *armatus*, and provide a revised diagnosis of *M*. *longicollum*, as well as revised diagnoses for *D*. *armatus*, Dacentrurinae, and the first diagnosis of the genus *Miragaia*, granting stability to these taxa and allowing new considerations to be given on the classification of other Iberian stegosaurs. This new specimen is, to date, the most complete dinosaur described from Portugal and the most complete stegosaur described from Europe. *Miragaia* shared anatomical features that show a close affinity to *Alcovasaurus longispinus*, confirming this to be the first known dacentrurine stegosaur in America, coherent with the hypothesis of an ephemeral land bridge between North America and Iberia that allowed faunal exchange.

## Introduction

The stegosaurs–meaning “plated” or “covered lizards”–were quadrupedal herbivorous dinosaurs characterized by a double row of parasagittal dermal plates and spines from the neck to the tip of the tail [1]. Stegosauria is one of the most iconic and unmistakable groups of prehistoric animals, best known by the largest and most famous genus, *Stegosaurus* Marsh, 1877 [2], which could measure up to nine meters in length, had a small head, front limbs shorter than the hind limbs, a double row of large bony plates over its body and two pairs of spines at the tip of the tail. However, other species of the group had relatively smaller plates, could have a large spine over each shoulder and be decked with spines over most of their tails and backs (instead of plates). The plates most likely served adaptively for display and facultatively (at least in *Stegosaurus*) for thermoregulation [3–6], while the set of long bony spines at the tip of the tail (known as *thagomizer*) were probably a very efficient weapon against predators, as evidenced by damaged bones and biomechanics [7,8]. The closest relatives of stegosaurs were the ankylosaurs, with whom they comprised the Thyreophorans (meaning "shield bearers", also known as the armoured dinosaurs)–along with some basal armoured genera, linked by the presence of keeled osteoderms on their dorsa [9]. The first fragmentary remains of stegosaurs were found in the 1840s [10,11], but the first stegosaur skeletons were found and described in the 1870s in North America and Europe [2,12], leading to the erection of the clade Stegosauria in 1877 [2]. The stem-based Stegosauria is defined [13,14] as all taxa more closely related to *Stegosaurus stenops* Marsh, 1887 [15] than to *Ankylosaurus magniventris* Brown, 1908 [16]. Stegosauria is a small clade, with only 10–15 genera known (often monospecific), depending on the accepted validity for each taxon [1,9,17,18]. Stegosaurs are known unambiguously in North America, Europe, Asia, Africa and South America, from the Middle Jurassic to the Early Cretaceous [19]. Although a number of studies have included phylogenetical analyses of Stegosauria [1,9,17,20–24], since most stegosaur species are only known from partial or fragmentary specimens—with only a few species known from nearly complete specimens–the clade’s taxonomy and systematics are still some of the most mysterious and highly debated among dinosaurs [23].

### Systematical difficulties of dacentrurine stegosaurs

**Fig 1:**
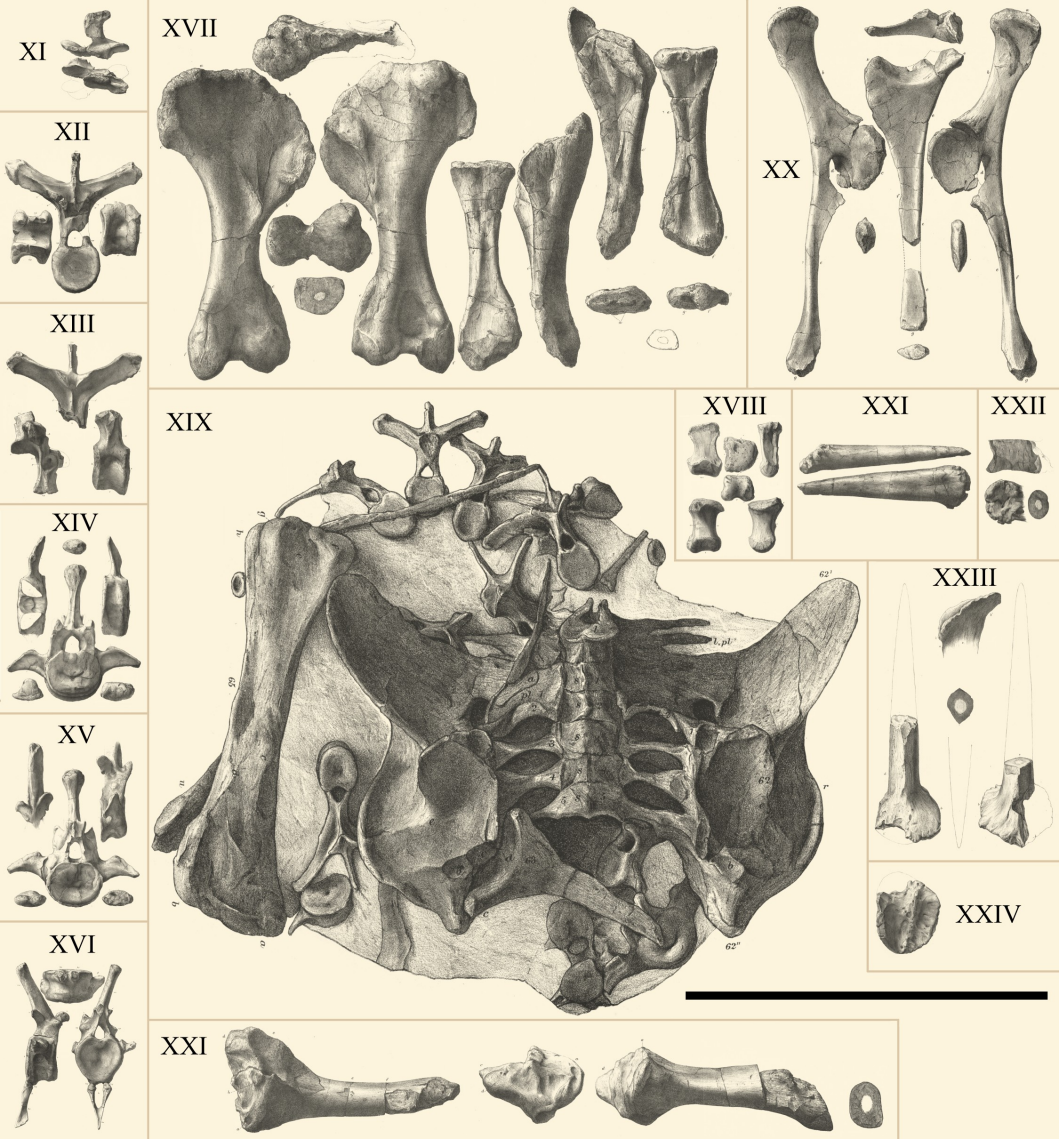
Illustrations of the holotypes of *D*. *armatus* and *O*. *hastiger*. **XI–XXII**, holotype of *D*. *armatus* (NHMUK OR46013) from [12] labelled the same as the plates therein, **XXIII, XXIV**, holotype of *O*. *hastiger* from [31] labelled the same as the plates therein. **XI**, posterior cervical neural spine, **XII**, **XIII**, dorsal vertebrae, **XIV**, **XV**, anterior caudal vertebrae, **XVI**, mid caudal vertebra, **XVII**, humerus, radius and ulna, **XVIII**, fourth left metacarpal, **XIX**, ilio–sacral block, femur, left ischium, and various dorsal and caudal vertebrae, **XX**, right pubis and ischium, **XXI**, spine and right tibia, **XXII**, spine and section of sacral vertebra. See Owen [12,31] for further details. All skeletal elements to same scale according to [12,31], Scale bar equal to 1m.

*Dacentrurus armatus* (Owen, 1875) [12] was the first representatively complete stegosaur to be described worldwide, two years prior to *Stegosaurus*, giving the first glimpse of the complete anatomy of these animals. Owen originally named the genus *Omosaurus* Owen, 1875 [12], but Lucas [25] noted in 1902 that it had been already used for a crocodilian [26], thus proposed *Dacentrurus* as the new genus name–in allusion to the powerful tail spines–keeping *O*. *armatus* as type species. The holotype of *D*. *armatus* (NHMUK OR46013; Natural History Museum, London, United Kingdom; Fig 1) is a partial skeleton from the Lower Kimmeridge Clay (Upper Jurassic) of Swindon, Wiltshire, United Kingdom [27]. Owen first described and figured its remains in 1875 [12], Galton [27] redescribed in 1985 the holotype and photographed the individual skeletal elements and, in 2008, Maidment *et al*. [9] gave the latest review of the species. The specimen would have been 7 m long and comprises three cervical vertebrae, 16 dorsal vertebrae, 11 caudal vertebrae, left humerus, radius, ulna, carpus, metacarpals, ilio-sacral block, both ischia and pubes, right femur, partial right tibia, calcaneum, metatarsals, a dermal plate, and a tail spine (*sensu* [9]).

In the decades following the discovery of NHMUK OR46013, various more incomplete stegosaur specimens were found throughout Europe, essentially all being referred to *Omosaurus* (and later *Dacentrurus*) as the genus was becoming somewhat of a “wastebasket” taxon for stegosaur remains found on the continent (much like *Megalosaurus* Buckland, 1824 [28] had become for theropods; [9,27,29]). Various species were named based on these specimens, most being later synonymized with *D*. *armatus* [9,23,27,29], while a few were re-referred to new genera (such as *Loricatosaurus priscus* Maidment *et al*., 2008 [9] and *Miragaia longicollum* Mateus *et al*., 2009 [22]; [23]). Given the incomplete nature of the holotype of *D*. *armatus*, these various specimens historically referred to it (most often much more fragmentary) were used in attempts to clarify the taxonomy and diagnosis of the genus and species, resulting in inconsistency and inconclusiveness of what *Dacentrurus* and *D*. *armatus* are. This meant that the phylogenetic relationships and position of *Dacentrurus* has suffered various interpretations (in great part also due to the poorly resolved and unstable phylogeny of the whole Stegosauria clade [9]), so *Dacentrurus* has been found as a basal stegosaur [1,9,21], as the sister taxon to *Hesperosaurus mjosi* Carpenter *et al*., 2001 [20], but currently it is more consensually found as one of the most derived genera and sister taxon to *M*. *longicollum* in the clade Dacentrurinae [17,22,23]. As [9] concluded that only the holotype can be safely referred to *D*. *armatus*, currently the species can only be unambiguously diagnosed (*sensu* [9] and [22]) by the straight dorsal surface of the ischial shaft; preceding publications gave more extensive diagnoses, but, with following reviews, univocal diagnostic characters became scarcer, as some were found to be diagnostic of Dacentrurinae; [1,9,22]. [30] suggested some revised autapomorphies for *D*. *armatus* based on the relatively common ilio-sacral block (i.e., anteriorly short and broad preacetabular process of the ilium; a smooth curvature between the anterior margin of the sacral plate and the medial margin of the preacetabular process) but these are based mostly on Portuguese specimens tentatively identified as *Dacentrurus*, not on the holotype.

Two years after naming *O*. *armatus*, Owen also described a pair of tail spines (NHMUK OR46320; Fig 1XXIII and 1XXIV) as the holotype of *Omosaurus hastiger* Owen, 1877 [31], from the same division of the Kimmeridge Clay [29], characterized by two longitudinal ridges on opposite sides that give a fusiform transversal cut to the spines [12]. *Omosaurus lennieri* Nopcsa, 1911 [32] was the first stegosaur described from France, based on a partial skeleton from the Lower Kimmeridgian of the cliffs of Octeville, Cap de La Hève, Normandy, but this specimen was destroyed in 1944, a victim of the Second World War. Only illustrations of the specimen remained (see [29,32]), and it consisted of seven cervical vertebrae, seven dorsal vertebrae, a sacrum with seven fused centra and sacral ribs, the left ilium, the posterior ends of one pubis and one ischium, the first caudal vertebra and one femur [29]. After reviewing the material ascribed to *D*. *lennieri* material, [29] regarded it a junior synonym to *D*. *armatus* and [9] classified the holotype as *Dacentrurus* sp.

In 1908, a partial stegosaur skeleton was collected near Alcova (Wyoming, USA), from the Kimmeridgian-Tithonian of the Morrison Formation and, in 1914, Gilmore described and named it as a new species within the genus *Stegosaurus*–*S*. *longispinus* Gillmore, 1914 [33]. The specimen included 42 vertebrae, the right femur, sacrum, partial ilia and ischia, anterior process of left pubis and two posterior caudal spines, but water damage from a broken pipe destroyed the specimen in the 1920s, except for the right femur and casts of the two caudal spines [18]. [18] expanded the holotype’s description resorting to the remaining material and photographs, concluding that it represented the new genus *Alcovasaurus* Galton and Carpenter, 2016 [18]. The phylogenetic relationship of this species to other stegosaurus has never been clear, since scarse material and information remains to compare it with other stegosaurs (including the holotype of *Miragaia*, with which no overlapping well described skeletal elements are known). *A*. *longispinus* has therefore been essentially left out of cladistic studies, except for a phylogenetical analysis of Stegosauria [17] that found it outside Eurypoda, and the first phylogenetical super-matrix of Thyreophora [24] which found it as the sister-taxon to *Tuojiangosaurus multispinus* Dong *et al*., 1977 [34] (the first and so far only time *Alcovasaurus* has been recovered within Stegosauria by a phylogenetic analysis).

In Portugal, the first non-fragmentary dinosaur reported and properly prospected was the partial skeleton of a stegosaur from Pedras Muitas, near Baleal (Peniche), in 1942, as a result of methodical geological prospection on the central west coast of Portugal by Georges Zbyszewski and Octávio da Veiga Ferreira [35]. This specimen comprises of an anterior caudal vertebra, a metacarpal, a partial ilio-sacral block, an ischium, a partial pubis, a partial ilium, a right femur, a deformed dorsal vertebra, and fragments of three dorsal vertebrae [35,36]. The stegosaur from Pedras Muitas was found in the Kimmeridgian beds at the top of the cliffs and is a particularly large stegosaur (the femur is 110 cm tall), being later identified by [35] as *O*. *armatus*. Like almost all other stegosaur specimens found and described by [35], it has since been on display in the Museu Geológico de Lisboa, Lisbon, Portugal.

The discovery of the Pedras Muitas stegosaur caught the attention of Lapparent and Zbyszewski on the fossil reptiles from the central west coast of Portugal, resulting in an increase of dinosaur findings in Portugal since then [35], including at least four more stegosaur specimens described by the authors [35]. The most remarkable new stegosaurian specimens were three partial skeletons from Alfeizerão (near São Martinho do Porto), Atalaia (Lourinhã), and the Murteiras ravine (Foz do Arelho), all classified by the authors as *O*. *lennieri* due to their smaller size and relative slenderness (see [29] and [35] for detailed illustrations and photographs of the specimens).

The Alfeizerão specimen, attributed to the Upper Lusitanian (current Kimmeridgian) was the first stegosaur found in Portugal, in 1908, but it was not described until 1957 [35]. The authors reported two dorsal, three mid caudal and three posterior caudal vertebrae, two humeri, the right tibia (later recognized as the right femur with the proximal end missing [29]), a caudal spine, several rib fragments and a misidentified stegosaur egg (revealed later to be a concretion [37]).

The Murteiras specimen was found in July 1945 in a Kimmeridgian to Portlandian (current Tithonian) cliff and comprised, according to [35], of two posterior cervical centra, three dorsal vertebrae, an almost complete ilio-sacral block with five sacral vertebrae (including two presacral dorsal vertebrae fused to it), eight anterior caudal vertebrae, three mid caudal vertebrae, several rib fragments, a left femur, and two caudal spines. Some more vertebrae (two cervical, one anterior caudal and three mid caudal vertebrae) were also found mixed with this specimen, but considered originary from a different animal (an *O*. *armatus*) due to their bigger size [35].

The Atalaia specimen, a smaller animal from the Lower Tithonian, included a fragmentary but mostly complete ilio-sacral block, the right femur, four caudal vertebrae, the neurapophysis of an anterior caudal vertebra, several ribs, and a caudal spine [35]. Additional stegosaur material from other localities was also reported by [35]: a right femur from São Bernardino (IST A; Instituto Superior Técnico, Lisbon, Portugal) and the base of a neural spine from Porto Dinheiro (misspelled “Portinheiro”) were classified as *O*. *armatus*; some caudal vertebrae (two anterior, three middle and four posterior) from Areia Branca (MG 4862; Museu Geológico de Lisboa, Lisbon, Portugal, former MSGP, Museu dos Serviços Geológicos de Portugal), a spine from Porto Novo, a spine base from Pombal, and an isolated caudal vertebra with no provenance information (found in the storage of Museu Nacional de História Natural e da Ciência, Lisboa, Portugal) were identified as *O*. *lennieri*.

Lapparent and Zbyszewski [35] also described a partial upper jaw from the Sinemurian of São Pedro de Moel as a possible stegosaur, naming it *Lusitanosaurus liasicus* Lapparent and Zbyszewski 1957 [35]. The authors however drew similarities to *Scelidosaurus* Owen, 1861 [38], so it was more likely a basal thyreophoran, but further analyses are not possible since the specimen was lost in the fire at Museu Nacional de História Natural e da Ciência, Lisbon, in 1978. *Astrodon pusillus* Lapparent and Zbyszewski, 1957 [35] was named on a badly preserved partial postcranial skeleton from the Kimmeridgian of Casal da Pedreira (Lourinhã), thought by the authors to be a new sauropod species, but it is more likely a juvenile stegosaur [29, 39]. Galton [39] identified the proximal end of the right femur, a possible calcaneum, a metatarsal, the possible posterior end of a pubis, the distal end of the right radius, a metacarpal, an ungual phalanx, a dorsal centrum, a sacral centrum, a caudal centrum and two partial sacral ribs.

In 1991 Galton [29] reviewed and redescribed all the stegosaurian material previously described in Portugal, referring almost all of it to *D*. *armatus* [29]. Further material identified as *D*. *armatus* from the Tithonian and Kimmeridgian of Portugal has been reported in 2003 by [40], including various specimens not yet described, housed at the Museu da Lourinhã. [41] reported a stegosaur from Moçafaneira (Torres Vedras, Portugal) with dorsal centra wider than long and a deep prepubic process, hence identifying it as *D*. *armatus*. It included an almost complete dorsal vertebra, three dorsal centra, nine dorsal neural arches, a partial sacrum, the anterior process of the right pubis, and fragments of the osteoderms. A partial specimen from Batalha is the only stegosaur so far found in Portugal that has been conclusively identified as not *Dacentrurus* or a close relative [21], as the authors found the most affinity phylogenetically to *Stegosaurus ungulatus* Marsh, 1879 [42] and synapomorphies of *Stegosaurus*, being the first supported occurrence of the genus in Europe [21]. A preliminary study [43] reports a *Stegosaurus* leg from Vale de Pombas (Peniche, Portugal), but uses two synapomorphies for Stegosauria and one for Thyreophora to implausibly classify it to the genus. All stegosaur findings in Portugal are Jurassic except for a stegosaurid tail spine (ML 1920; Museu da Lourinhã, Lourinhã, Portugal) from the Berriasian of Praia da Calada (Mafra) briefly reported by [44].

Stegosaur tracks in the Late Jurassic of Portugal are very common, seemingly more than of any other dinosaur, or in other localities [45]. In the Lourinhã Formation, 38 isolated tracks have already been described, 34 of which were attributed to the ichnogenus *Deltapodus* [45–48]. At this point, it is undetermined what genus or species made these tracks, but it can be hypothesized that dacentrurine stegosaurs are the trackmakers of *Deltapodus* since these match the most in size, morphology, distribution, chronology and abundance (for further details, see [45]).

Since 1995, a rich record of stegosaurian material has been reported from Spain [49–61]. Most of the Spanish specimens are partial skeletons or collections of vertebrae from the Tithonian-Berriasian Villar del Arzobispo Formation in Teruel and Valencia. These specimens have been mostly identified as *Dacentrurus* sp. or aff. *Dacentrurus* sp. due to their resemblance to *Dacentrurus armatus* and *Miragaia longicollum*, but with noticeable differences [9,53,56,57]. A very few bones were also found in the Lower Cretaceous (Hauterivian to Aptian) of Teruel and Burgos [57,58,60,61], but the classification of most of these as stegosaurian is dubious [9].

Maidment *et al*. [9] studied first-hand all the stegosaur material previously described and referred to *Dacentrurus* (or *Omosaurus*) and concluded that only *D*. *armatus* was a valid species of *Dacentrurus*–with only the holotype referred to it–and only a few specimens were complete enough to pertain to *Dacentrurus* sp. The authors noticed some slight differences to the holotype in the Iberian material–particularly in the Portuguese material–but considered it too incomplete to determine autapomorphies and erect potential new species. From Portugal, only the partially complete specimens from Pedras Muitas, Alfeizerão, Atalaia and Murteiras were classified as *Dacentrurus* sp., while all other possible stegosaur specimens were considered either Dinosauria indet. or Thyreophora indet. (see [9]).

**Fig 2:**
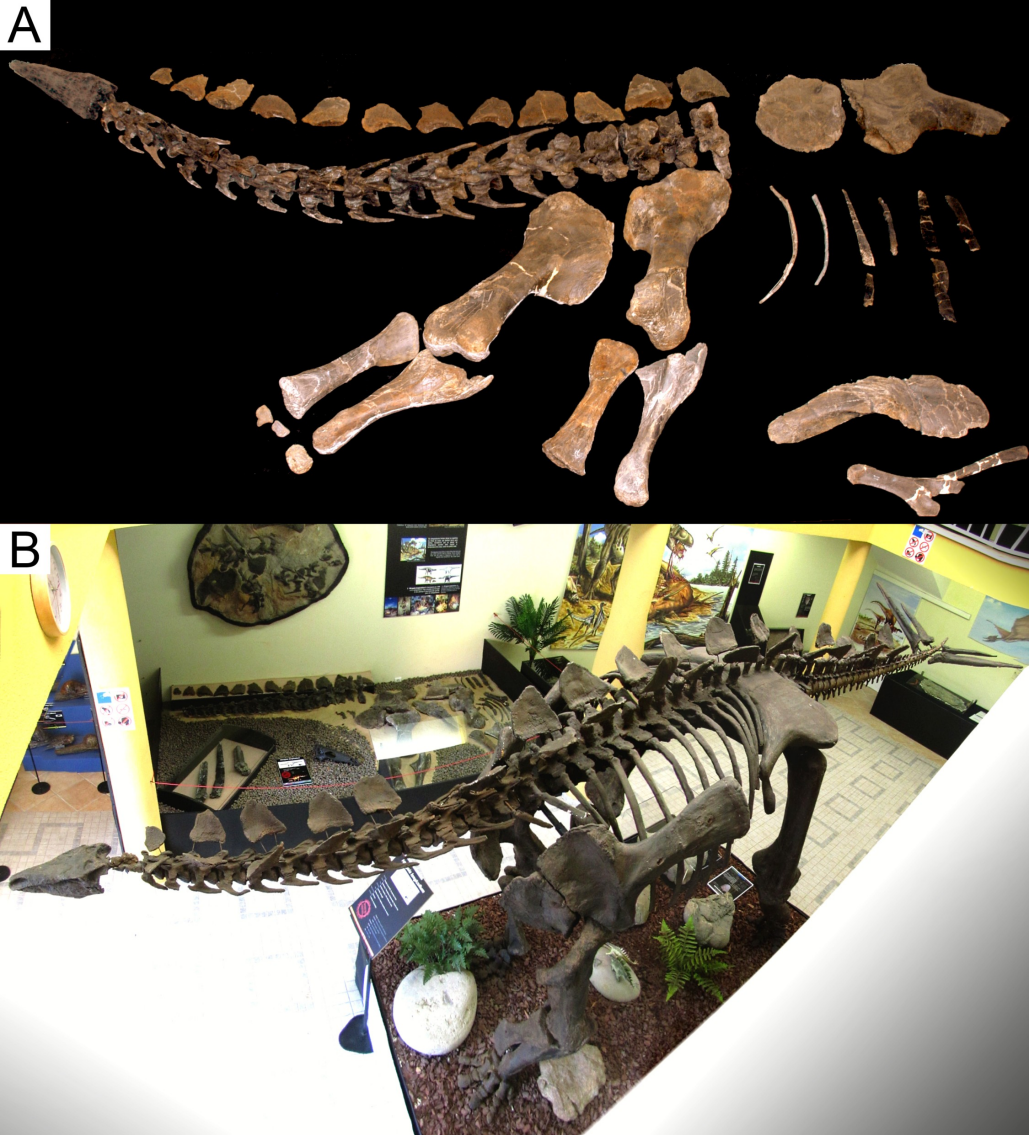
Holotype of *Miragaia longicollum*. **A,** Holotype of *Miragaia longicollum* (ML 433) and pelvic bones of referred juvenile specimen (ML 433–A). **B**, *Miragaia* material in the Museu da Lourinhã: Full body replica (foreground), holotype, juvenile specimen and isolated thagomizer spines (background, spread on the floor), replica of most of the holotype material as found in the field (background, mounted on the wall) and fossilized tracks (under full body replica).

The stegosaurian dinosaur *Miragaia longicollum* was named in 2009 after a specimen (ML 433) from the Upper Kimmeridgian-Lower Tithonian Praia Azul Member of the Lourinhã Formation (Miragaia Unit, most likely earliest Tithonian) of the village of Miragaia, Lourinhã [22]. It comprises the nearly complete anterior half of the skeleton: a partial cranium, 15 cervical vertebrae with associated ribs, two dorsal vertebrae, both coracoids, scapulae, humeri, radii and ulnae, one metacarpal, three phalanges, 12 rib fragments, one chevron, one dermal spine, and 13 dermal plates ([22]; Fig 2). A juvenile referred specimen (ML 433-A) was also found with the holotype, that includes some dorsal vertebrae, the left ilium, and both pubes. The genus was named for the type locality, which also means ‘wonderful goddess of the Earth’, and the species for the Latin for ‘long neck’. The species was diagnosed by [22]: anterior tip of the premaxilla is drawn into a point; anterolateral rim of the premaxilla projects ventrally; at least 17 cervical vertebrae; mid-cervical neural spines possess a notch at their base with an anterior projection dorsal to it; mid and posterior cervical and anterior dorsal neural spines with transversely expanded apices; and paired, slightly outwardly convex, triangular cervical dermal plates with a notch and projection on the anterodorsal margin. ML 433 is missing the atlas and axis, so *M*. *longicollum* would have had at least 17 cervical vertebrae, which means that it had one of the longest necks among non-avian dinosaurs (outnumbered by only a few of the longest sauropod species), at least four more neck vertebrae than all other known stegosaurian species and eight more than basal Thyreophorans and ornithischians [19,22]. This elongation suggests an unique ecological specialization among stegosaurs (likely for high browsing) and was achieved by cervicalization of two dorsal vertebrae and by elongation and addition of vertebral elements [22].

The holotype of *Miragaia* was for some time, like almost all Portuguese stegosaurs previously found, thought to pertain to *Dacentrurus* [40,62]. Both forms are very similar morphologically and were found to be sister taxa by [22], leading to the erection of Dacentrurinae, sister taxon to Stegosaurinae (the clade that includes *S*. *stenops*, *Hesperosaurus mjosi* and *Wuerhosaurus homheni* Dong, 1973 [63]; *sensu* [64]). Dacentrurines are characterized by dorsal centra wider than long, cervical ribs fused to the cervical centra, presence of the olecranon horn in the ulna and a process projecting dorsally on the anterior tip of the prepubis [22]. Most of the synapomorphies for Dacentrurinae were previously used to diagnose *Dacentrurus* and *D*. *armatus* (e.g., [1,27,9]), but were found to be shared by both species [17,22]. Besides the autapomorphies named by [22], further differences of *M*. *longicollum* to *D*. *armatus* can be found in the dorsal plates (in *D*. *armatus* they have a thick central portion like a modified spine, while in *M*. *longicollum* they have a generally thin transverse structure) and in the proportions between the hindlimb elements [22,56].

The relationship of *Dacentrurus* and *Miragaia* and the limited overlapping material between the holotypes has led some authors (e.g., [56,65]) to hypothesize that *Miragaia* may be a junior synonym of *Dacentrurus–*mainly since the diagnostic characters for *Miragaia* were defined on anterior elements, mostly unknown in the *Dacentrurus* holotype and other Portuguese stegosaur specimens. However, the lack of overlapping material also means that the synonymy cannot be assuredly corroborated [56,65], as the autapomorphies of *M*. *longicollum* may not apply on the missing anterior skeleton of *D*. *armatus*. [56] describes stegosaur material from the Tithonian-Berriasian Villar del Arzobispo Formation (Teruel, Spain) that includes some elements that overlap the holotypes of *Miragaia* and *Dacentrurus*. Despite a noticeably rich record, the skeletal elements are mostly isolated and poorly preserved, limiting comparative interpretations [56]. The authors recognize some differences and some similarities to the English holotype of *D*. *armatus*–such as an ischium with straight dorsal surface–and to *M*. *longicollum*–such as cervical vertebrae more alike than previously thought [56]. The authors suggest the Teruel material could be assigned to aff. *Dacentrurus armatus* or cf. *Miragaia longicollum* (conservatively it was assigned to aff. *Dacentrurus* sp.), suggesting that these could not be differentiated and *M*. *longicollum* should be understood as aff. *Dacentrurus armatus* until the autapomorphies of each were reliably confirmed. Despite this putative and conservative conclusion, some subsequent publications (e.g., [53]) follow it therein by considering *M*. *longicollum* as a junior synonym to *D*. *armatus* (using the autapomorphies of *M*. *longicollum* as diagnostic of *D*. *armatus*) and naming some of the Spanish material to this species–regardless of the previous recognitions that the Iberian material differs from NHMUK OR46013 and may be referable to a different species. [17] found–with a re-analysis of the phylogenetic relationships of Stegosauria using a revised character list from [23], with added continuous characters–that *M*. *longicollum* is the sister taxon to *H*. *mjosi* and not to *D*. *armatus*. However, the authors recognize that the pairing is poorly supported, whereas the grouping of *D*. *armatus* with *M*. *longicollum* appears more supported if the continuous data is excluded, but conclude that the results still favor that *M*. *longicollum* should not be synonymized with *D*. *armatus* (and also that *H*. *mjosi* should not be considered a species of *Stegosaurus*; [17]).

Although a plethora of additional material with affinities to *Dacentrurus* and *Miragaia* has been published since the earliest discussions on the validity of *M*. *longicollum*, no consensually supported synonymization or differentiation of *D*. *armatus* and *M*. *longicollum* has been achieved yet. As stated by most of the studies discussing the validity of *M*. *longicollum* (e.g., [17,56]), more complete specimens are needed to conclusively and directly compare the species and, if that is the case, define the characteristics that differentiate them (“The relationships of *Dacentrurus armatus*, *Miragaia/Dacentrurus longicollum*, and the large amounts of isolated stegosaur material known from the Iberian Peninsula will probably only be satisfactorily resolved once overlapping material between the holotypes of *Dacentrurus armatus* and *Miragaia longicollum* comes to light”; [17]).

**Fig 3:**
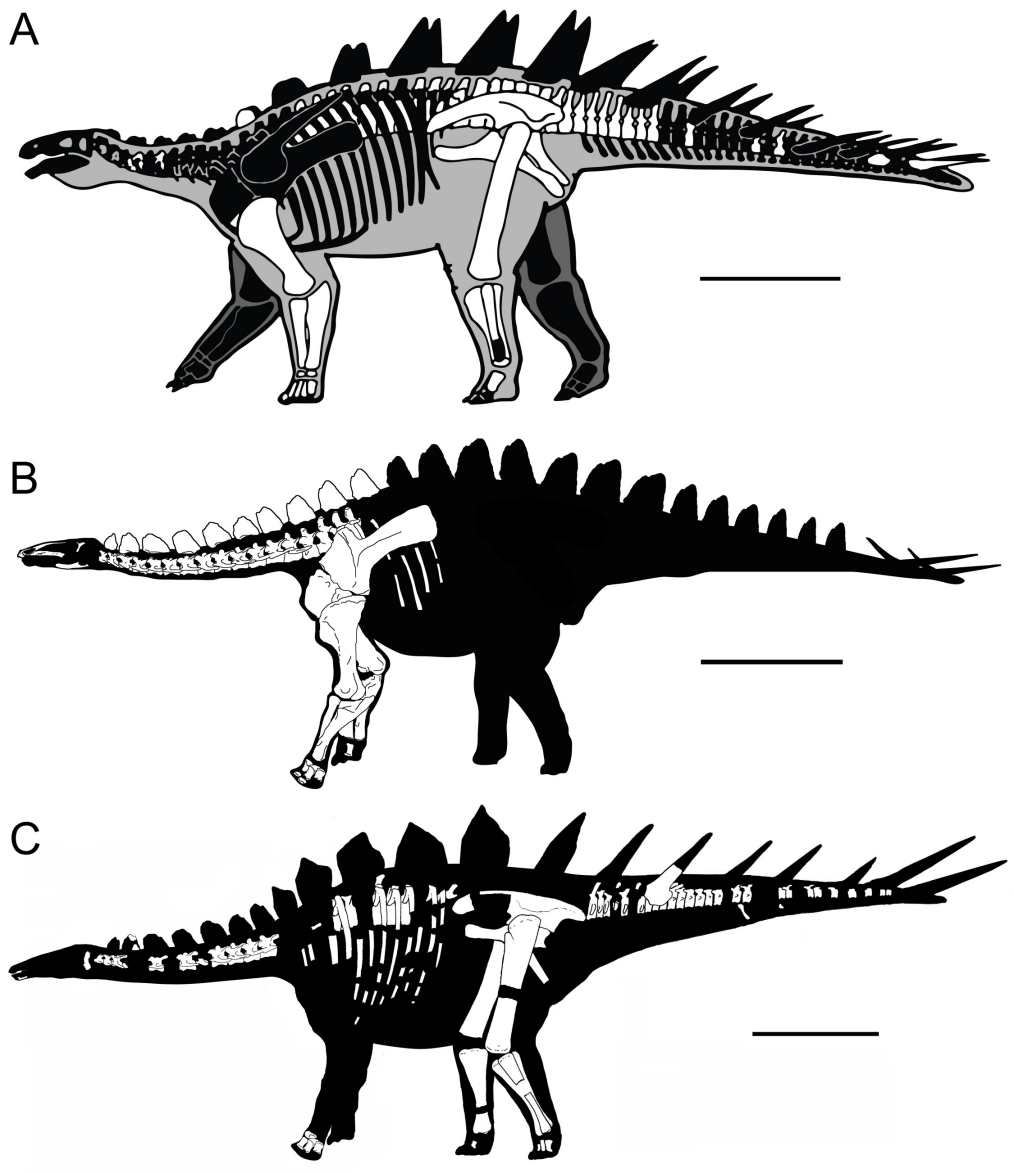
Skeletal elements found of *Dacentrurus armatus* NHMUK OR46013, and *Miragaia longicollum* ML 433 and MG 4863. **A,** NHMUK OR46013 (modified reprint from: http://www.paleofile.com under a CC BY 4.0 license, with permission from Tracy L. Ford, original copyright 201), **B**, ML 433 (illustration from [22]), **C,** MG 4863 (preliminary reconstruction adapted from illustrations by Simão Mateus, Oliver Demuth and from [22]).

MG 4863 (Museu Geológico, Lisboa, Portugal), a recently prepared and considerably complete stegosaur specimen from the Lourinhã Formation of Atouguia da Baleia (Peniche, Portugal; Fig 3C), has clear affinities to *Miragaia* and *Dacentrurus* and overlaps several bones of the holotypes of both (Fig 3A and 3B), making it the ideal comparative ground between these species–effectively a “Rosetta Stone” specimen. If comparisons can conclusively identify it to one species and not to the other, it would confirm that these are two different species, eventually providing revised diagnoses.

The main purpose of this paper is to provide a detailed description of the osteological anatomy of MG 4863, with the objective of comparing it with other stegosaurs (primarily the holotypes of dacentrurines), discussing and conclusively determining the taxonomical validity of *Miragaia longicollum*, as well as to review the diagnoses of dacentrurine taxa and attempt to grant stability to the clade Dacentrurinae.

## Material

### Material studied

**Fig 4:**
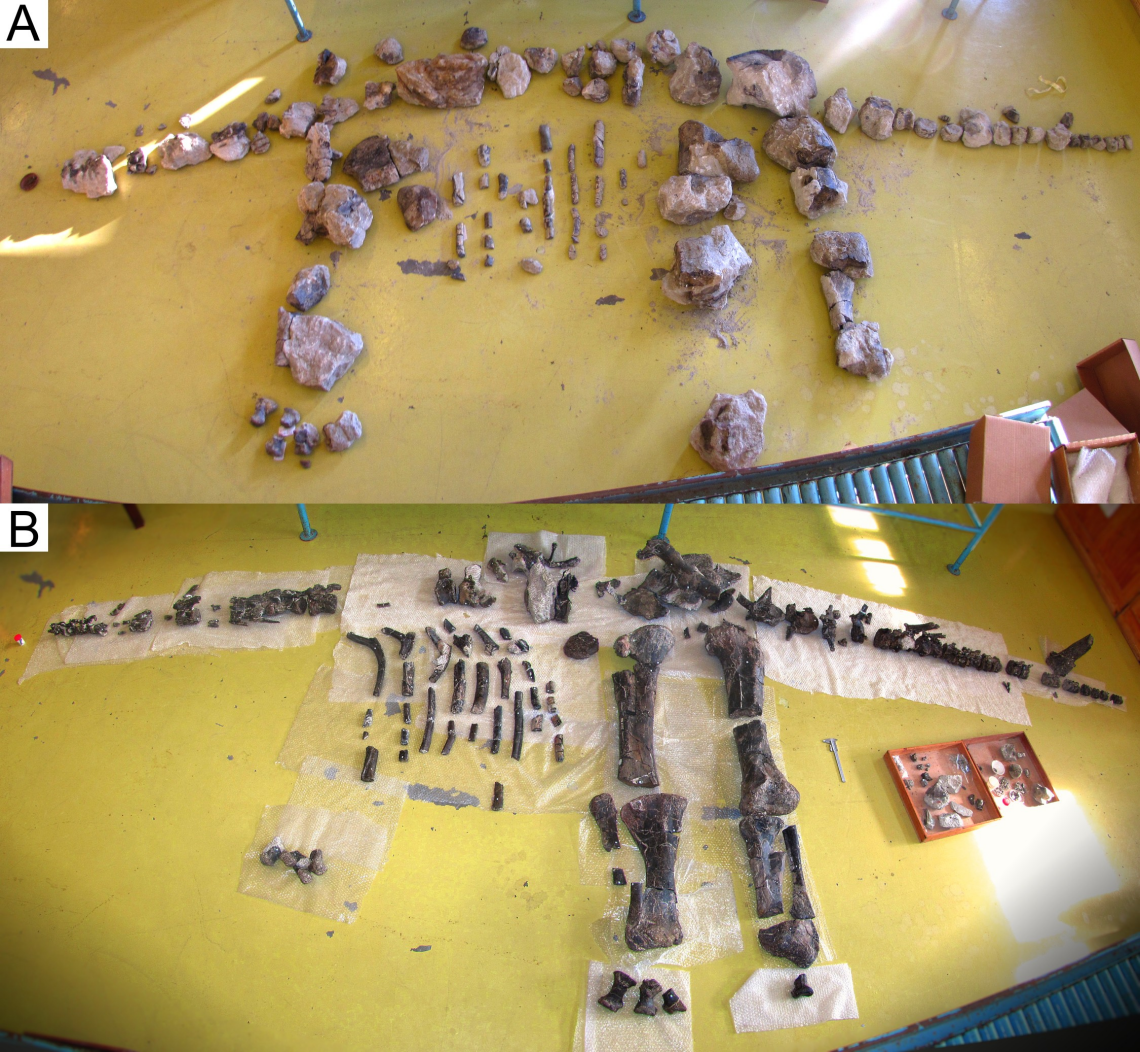
*Miragaia longicollum* MG 4863. **A**, skeleton of MG 4863 laid out in preliminary articulation before preparation (September 2015), **B**, the same laid out in semi–articulation after preparation (May 2017; unidentified skeletal elements are on the boxes on the right; caliper is 20 cm long, but the photograph should not be used for scale purposes due to distortion of panoramic photography).

The stegosaur from Atouguia da Baleia herein described has been stored for long in the Alfragide campus of LNEG (Laboratório Nacional de Energia e Geologia, Portugal), where it is still housed. At the beginning of this study (September of 2015), the specimen was unprepared (Fig 4A), undescribed, had no collection number or field notes, was identified only by the label “Atouguia da Baleia” in some of the boxes it was stored in, some of its material was mixed and stored together with unprepared material of the holotype of the ankylosaur *Dracopelta zbyszewskii* Galton, 1980 [66] (MG 5787) or mislabeled in some boxes as “*Dracopelta*” and/or “Ribamar” (Ribamar was previously the believed provenance of MG 5787; [67]), and it was unclear what was the exact location, year and author of its discovery. In 2009, Bruno Pereira (ML) noticed the unprepared specimen while updating the fossil record of LNEG, and notified OM about it shortly afterwards, who recognized its affinities to *M*. *longicollum* and that it was considerably complete. OM also noticed that it included various cervical vertebrae and portions of the posterior skeleton, recognizing that it could be used to further compare *M*. *longicollum* and *D*. *armatus*. In 2015, the stegosaur became the main subject of the Master’s thesis in Paleontology of one of the authors (FC), resulting in the study herein.

MG 4863 was almost fully prepared during this study. Its remains were mostly encased in various blocks of sandstone, purportedly in the state they were collected in the field, with little to no information of how these were arranged, connected or if any fragments were missing. Since field maps or notes of the find were not present, it was not possible to fully analyze how articulated or complete the specimen was (including if there may have been additional missing elements that were either left in the field or could have been stored somewhere else). Although attempts of reconstruction of the skeleton revealed that it was possible to fit various sets of blocks back as they were originally connected, it was not possible to recreate a general field map of the specimen. Some bones or blocks presented numbers written in red crayon over them, presumably contemporaneous to the collecting, but their meaning is unclear without field notes and most have watered down to the point of being indistinct, thus providing no useful information during the preparation and interpretation of the specimen. During this study, the skeletal elements of MG 4863 were successfully separated from the mixed remains of the holotype of *D*. *zbyszewskii*, mainly by figuring the characteristic geochemical properties of the matrixes of each specimen (see [68]). This data and the lack of doubled bones confirm that all stegosaur bones under MG4863 belong to the same and a single individual.

The skeleton of MG 4853 is relatively complete, with some major body regions well represented, comprising: partial left dentary and left quadrate, ten cervical vertebrae with cervical ribs, five dorsal vertebrae, 35 partial dorsal ribs, 25 caudal vertebrae, two chevrons, a caudal spine, left ilium, left sacral ribs, left pubis, partial left ischium, femora, tibiae with fused calcanea and astragali, fibulae, four metatarsals, three metacarpals, partial radiale and various fragments of vertebrae, plates or unidentified bones (Fig 4B). The stegosaur specimen MG 4863 is currently the most complete dinosaur described in Portugal (as complete or more than the lectotype of *Lourinhasaurus alenquerensis* Dantas *et al*., 1998 [69]; [70]), the most complete stegosaur found in Europe (more complete than the holotypes of *D*. *armatus* and *L*. *priscus* or other specimens referred to these; [9]) and one of the most complete stegosaurs in the world. A complete list of all the skeletal elements of MG 4863 is given in Table 1. Almost all skeletal elements of MG 4863 suffered some form of deformation (such as compression, breakage, crushing or twisting) but the level of detailed preservation is remarkably high, retaining textures nearly identical to when in life (such as rugosities, bone fibers and cancellous bone). a 2 mm crocodylomorph tooth, one fish tooth, up to five fish scales and a squamate parietal there were found associated with MG 4863 during laboratory preparation.

**Table 1:** List of fossilized skeletal material of MG 4863 in the LNEG collection.

Col. sub-number	Material	Taphonomy
**MG 4863–1**	Dentary, left	Anterior tip of left dentary
**MG 4863–2**	Cervical vertebra and ribs 11	Missing tip of left cervical rib
**MG 4863–3**	Cervical vertebra and ribs 12	Missing tip of right cervical rib
**MG 4863–4**	Cervical vertebra and ribs 13	Missing postzygapophyses, left side of neural arch and left rib
**MG 4863–5**	Axis	Almost complete, but crushed and laterally deformed
**MG 4863–6**	Cervical vertebra 3	Laterally deformed
**MG 4863–7**	Cervical vertebra and ribs 4	Missing left cervical rib and laterally deformed
**MG 4863–8**	Cervical vertebra and ribs 5	Anterior half of centrum and tip of right prezygapophysis.
**MG 4863–9**	Cervical vertebra and ribs 7	Missing most of the ribs and zygapophyses, laterally deformed
**MG 4863–10**	Cervical vertebra and ribs 9	Missing tip of right cervical rib.
**MG 4863–11**	Cervical vertebra and ribs 14	Missing posterior half of right cervical rib and parts of neural arch
**MG 4863–13**	Dorsal vertebra 7	Missing most of the top of neural arch
**MG 4863–14**	Dorsal vertebra 2	Centrum and neural arch
**MG 4863–15**	Dorsal vertebra 3	Centrum and neural arch
**MG 4863–16**	Dorsal vertebra 6	Centrum and parts of neural arch
**MG 4863–17**	Dorsal vertebra 5	Almost complete
**MG 4863–19**	Caudal vertebra 28	Centrum and pedicels
**MG 4863–20**	Caudal vertebra 3	Missing distal half of neural spine and most of prezygapophyses
**MG 4863–21**	Caudal vertebra 5	Missing distal half of neural spine and most of prezygapophyses
**MG 4863–22**	Caudal vertebra 8	Missing parts of neural arch
**MG 4863–23**	Caudal vertebra 9	Missing parts of neural arch
**MG 4863–24**	Caudal vertebra 10	Missing parts of neural arch
**MG 4863–25**	Caudal vertebra 11	Missing parts of neural arch
**MG 4863–26**	Caudal vertebra 12	Missing parts of neural arch
**MG 4863–27**	Caudal vertebra 13	Missing parts of neural arch
**MG 4863–28**	Caudal vertebra 14	Missing parts of neural arch
**MG 4863–29**	Caudal vertebra 15	Missing parts of neural arch
**MG 4863–30**	Caudal vertebra 16	Centrum with a pedicel
**MG 4863–31**	Caudal vertebra 23	Centrum
**MG 4863–32**	Caudal vertebra 24	Missing tip of right prezygapophysis
**MG 4863–33**	Caudal vertebra 27	Complete vertebra, sheared neural arch
**MG 4863–34**	Caudal vertebra 1	Missing tip of neural spine
**MG 4863–35**	Caudal vertebra 19	Missing left postzygapophysis
**MG 4863–36**	Caudal vertebra 34	Missing prezygapophyses
**MG 4863–37**	Caudal vertebra 32	Missing post- and prezygapophyses
**MG 4863–38**	Caudal vertebra 2	Missing neural spine and right caudal rib
**MG 4863–39**	Spine, left anterior	Missing distal half
**MG 4863–40**	Femur, left	Missing mid-section
**MG 4863–41**	Femur, right	Missing distal end, proximal end broken
**MG 4863–42**	Tibia and tarsals, left	Complete, slightly deformed
**MG 4863–43**	Tibia and tarsals, right	Severely deformed, distal end broken
**MG 4863–44**	Fibula, left	Proximal and distal ends
**MG 4863–45**	Fibula, right	Missing proximal end
**MG 4863–46**	Plate	Posterodorsal fragment of cervical plate
**MG 4863–47**	Dorsal rib, right	Proximal and medial section
**MG 4863–48**	Dorsal rib, right	Medial and distal section
**MG 4863–49**	Dorsal rib, left medial	Distal section
**MG 4863–50**	Dorsal rib, right medial	Proximal section
**MG 4863–51**	Chevron, posterior (?)	Chevron blade
**MG 4863–52**	Chevron 17 (?)	Complete chevron
**MG 4863–53**	Pubis, left	Missing end of postpubis
**MG 4863–54**	Indet. frag.	Possibly tip of preacetabular process of ilium
**MG 4863–55**	Caudal Vertebra 18	Missing right prezygapophysis
**MG 4863–56**	Caudal Vertebra 30	Missing parts of neural arch
**MG 4863–57**	Caudal Vertebra 36	Centrum and pedicels
**MG 4863–58**	Caudal Vertebra 7	Centrum and pedicels
**MG 4863–59**	Ilium, left	Missing preacetabular process
**MG 4863–60**	Cranial fragments	Various fragments, probably from dentary
**MG 4863–61**	Dorsal rib, left medial	Mid fragment
**MG 4863–62**	Dorsal rib, left	Mid-anterior section
**MG 4863–63**	Dorsal vertebra 10	Dorsal transverse process and postzygapophysis
**MG 4863–65**	Ischium, left	Iliac peduncle of left ischium
**MG 4863–66**	Metatarsal 4, left	Complete metatarsal
**MG 4863–67**	Metatarsal 3, left	Complete metatarsal
**MG 4863–68**	Metatarsal 3, right	Distal half
**MG 4863–69**	Metatarsal 2, left	Distal half
**MG 4863–70**	Metacarpal 1	Missing most of proximal half
**MG 4863–71**	Metacarpal 3	Missing proximal articulation
**MG 4863–72**	Metacarpal 2	Missing proximal articulation
**MG 4863–73**	Radiale (?)	Anterior margin
**MG 4863–74**	Dorsal rib	Tuberculum
**MG 4863–75**	Dorsal rib, right	Anterior fragment
**MG 4863–76**	Dorsal rib	Fragment
**MG 4863–77**	Dorsal rib, left	Proximal end of capitulum
**MG 4863–78**	Dorsal rib, left	Proximal fragment (tuberculum)
**MG 4863–79**	Dorsal rib, right	Proximal section, probably part of MG 4863–83
**MG 4863–80**	Dorsal rib, left medial	Mid-section
**MG 4863–81**	Dorsal rib, right medial	Mid-section
**MG 4863–82**	Dorsal rib, right	Mid fragment
**MG 4863–83**	Dorsal rib, right	Mid-section, probably part of MG 4863–79
**MG 4863–84**	Dorsal rib, right	Proximal end of capitulum
**MG 4863–85**	Dorsal rib, right	Medial section
**MG 4863–86**	Dorsal rib, right	Distal section
**MG 4863–87**	Plate	Proximal fragment of a cervical plate, possibly part of MG 4863–46
**MG 4863–88**	Dorsal rib	Distal section
**MG 4863–89**	Dorsal rib, right	Distal section
**MG 4863–90**	Dorsal rib, left	Mid fragment
**MG 4863–91**	Dorsal rib, left	Mid fragment
**MG 4863–92**	Dorsal rib, left	Distal end
**MG 4863–93**	Dorsal rib, left	Mid fragment
**MG 4863–94**	Dorsal rib, left medial	Mid fragment
**MG 4863–95**	Dorsal rib, right	Mid-distal fragment
**MG 4863–96**	Dorsal rib, left	Anterior fragment (tuberculum)
**MG 4863–97**	Dorsal rib	Mid-anterior fragment
**MG 4863–98**	Caudal frag.	Apex of anterior caudal neural spine
**MG 4863–99**	Dorsal rib	Mid fragment
**MG 4863–100**	Cervical rib 17, right	Mid fragment
**MG 4863–101**	Caudal vertebra 4	Proximal fragment of caudal rib of anterior caudal vertebra
**MG 4863–102**	Caudal vertebra 37	Missing parts of neural arch
**MG 4863–103**	Caudal frag.	Apex of anterior caudal neural spine (from Cd6 or Cd7)
**MG 4863–104**	Cervical frag.	Anterior process of cervical rib
**MG 4863–105**	Cervical frag.	Cervical rib fragment
**MG 4863–106**	Cervical frag.	Cervical right postzygapophysis
**MG 4863–107**	Cervical frag.	Cervical rib fragment
**MG 4863–108**	Cervical frag.	Cervical right postzygapophysis
**MG 4863–109**	Indet. frag.	Indeterminate fragment, possibly cranial
**MG 4863–110**	Cervical frag.	Cervical rib fragment
**MG 4863–112**	Dorsal frag.	Dorsal transverse process
**MG 4863–113**	Indet. frag.	Indeterminate fragment
**MG 4863–114**	Indet. frag.	Possibly distal end of ischium
**MG 4863–115**	Indet. frag.	Possibly plate fragment
**MG 4863–116**	Indet. frag.	Indeterminate fragment
**MG 4863–117**	Indet. frag.	Possibly osteoderm fragment
**MG 4863–118**	Indet. frag.	Possibly osteoderm fragment
**MG 4863–119**	Indet. frag.	Possibly osteoderm fragment
**MG 4863–120**	Indet. frag.	Possibly osteoderm fragment
**MG 4863–121**	Indet. frag.	Possibly osteoderm fragment
**MG 4863–122**	Ossified tendons	Broken sections
**MG 4863–123**	Indet. frag.	Probably part of the pelvic girdle, most probably from the ischium
**MG 4863–124**	Indet. frag.	Indeterminate fragment
**MG 4863–125**	Indet. frag.	Probably part of the pelvic girdle
**MG 4863–126**	Indet. frag.	Probably part of the quadrate
**MG 4863–127**	Indet. frag.	Probably part of the pelvic girdle, either from ischium or pubis
**MG 4863–128**	Indet. frag.	Indeterminate fragment. Possibly from *D*. *zbyszewskii*
**MG 4863–129**	Indet. frag.	Indeterminate fragment
**MG 4863–130**	Indet. frag.	Indeterminate fragment
**MG 4863–131**	Indet. frag.	Possibly part of the pelvic girdle
**MG 4863–132**	Indet. frag.	Indeterminate fragment
**MG 4863–133**	Indet. frag.	Indeterminate fragment
**MG 4863–134**	Dorsal frag.	Tip of dorsal transverse process

### Locality and geological context

**Fig 5:**
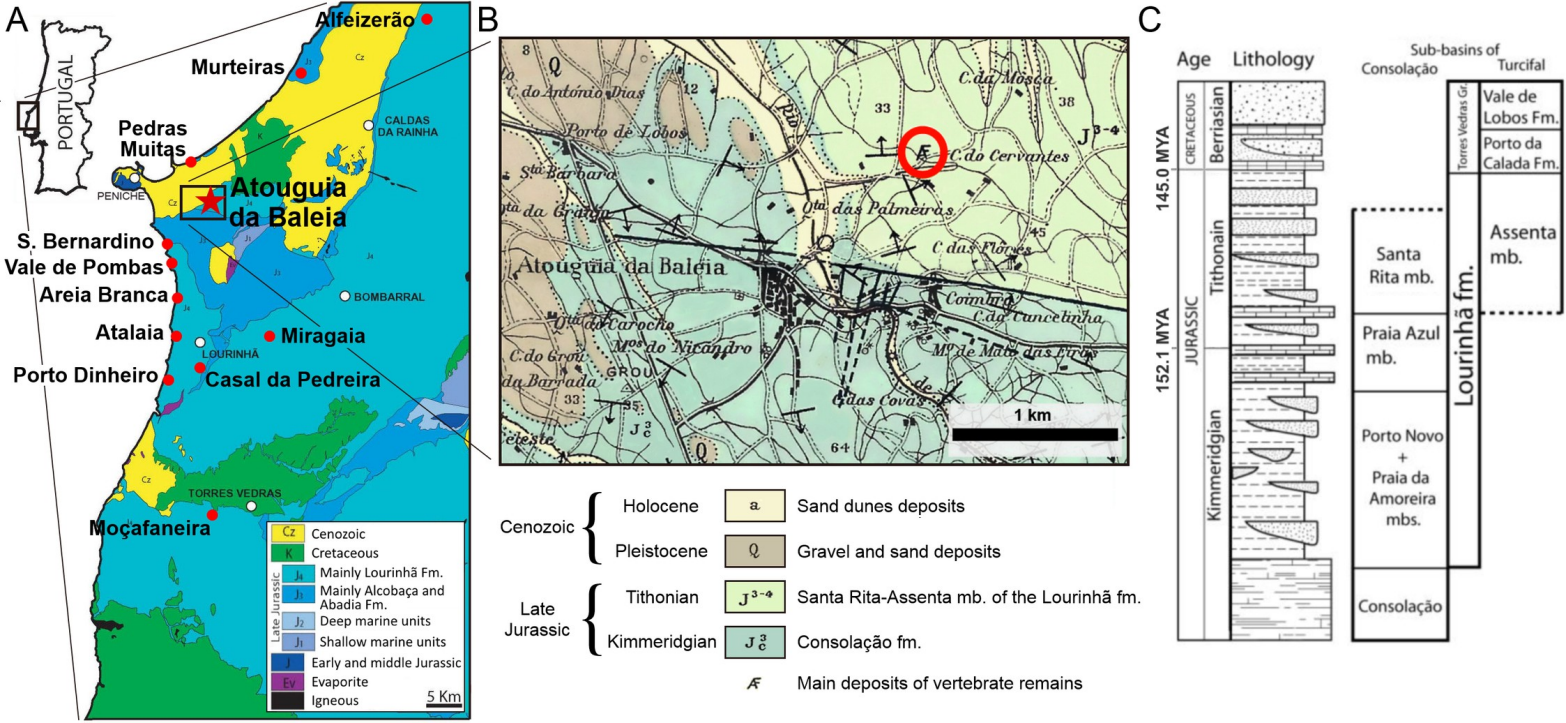
Geological context of dacentrurines in Portugal and locality of *Miragaia longicollum* MG 4863. **A**, Fossil sites of published and described occurrences of dacentrurine stegosaur skeletal remains in the Lusitanian Basin (including the presumed location of the Atouguia da Baleia specimen studied herein). Occurrences are marked in red with respective label of location (Murteiras, Pedras Muitas, São Bernardino, Areia Branca, Atalaia, Casal da Pedreira and Porto Dinheiro from [35]; Vale de Pombas from [43]; Miragaia from [22]; Moçafaneira from [41]). Simplified geological map of the central west coast of Portugal adapted from [73]. **B**, Detail of Geologic Map of Portugal at scale 1:50 000, sheet 26–C (Peniche) [72] including Atouguia da Baleia and the likely provenance of MG 4863 (circled in red), label simplified and adapted *sensu* [74]. **C**, Lithostratigraphy of the Lourinhã Formation from [74].

Georges Zbyszewski was most probably the author of the discovery of MG 4863, as almost all the dinosaur material stored in the LNEG was collected by him while conducting his extensive work of geologic cartography in central-west Portugal from 1942 to the 1970s for Serviços Geológicos de Portugal (current LNEG; [35,68]). The geologic survey of Atouguia da Baleia was carried out in 1959 [71] and the resulting Geologic Map of Peniche at 1:50 000 scale (sheet 26-C; [72]) includes only one coordinate near Atouguia da Baleia marked as a main deposit of possible dinosaur remains (Fig 5B), but of which no resulting published material is known [68]. Considering this and other sources of information co-supportive (for further details see [68]), it is most plausible that MG 4863 was collected in 1959 by Georges Zbyszewski about 1 km NE of the center of Atouguia da Baleia (39°20'45"N, 9°18'58"W; Fig 5A and 5B). The locality was visited by the authors of this paper in 2015, but since it is now covered by cultivating fields no observable outcrops for sampling and stratigraphic analysis are present in the area (however, local samples of the geology match the matrix from the specimen in sedimentology and geochemistry; [68]). As such, and considering that the existence of a field map of the discovery is currently unknown, it is not possible to trace exact horizons of the stratigraphy of the supposed provenance of MG 4863, so the geologic and paleontological context of MG 4863 herein presumed is based and limited to the most plausible inferences given by the available information (at least until future confirmation or correction, potentially by accessing the field books of Georges Zbyszewski which are in private custody at the time of writing).

If the mentioned coordinates are definitely the locality of MG 4863, then the specimen was found in the beds identified in [72] as “J^3-4^—Upper sandstones with plant and dinosaur fossils”, which is equivalent to the Tithonian aged Santa Rita-Assenta Member of the Lourinhã Formation (*sensu* [74]; synonymous also with the Bombarral Formation in [75], and the Portlandian in [76] and [77]; Fig 5C). The description by [72] of this area as “muddy sandstones with intercalations of mudstones and marls” also matches the matrix of MG 486.

The fossil sites with described stegosaurian skeletal remains in Portugal are all part of the Lusitanian Basin, more precisely are all purportedly part of the Lourinhã Formation (Fig 5A; see [22,35,40,41,72,76]). The Lourinhã Formation currently can be considered as Late Kimmeridgian to Late Tithonian [44,74], and is comprised of alternated sequence of sandstone and mudstone of continental fluvial facies (such as distal alluvial fans and upper deltas) punctuated by episodes of marine influence [73,74]. Underlying it are the Kimmeridgian limestones from shallow marine to brackish environments of the Alcobaça Formation, while at the top it is bounded by the sandstones of the Porto da Calada Formation, mainly Tithonian-Berriasian boundary in age. The Lourinhã Formation is composed (*sensu* [74]) of three units: the oldest Praia da Amoreira-Porto Novo Member, the Praia Azul Member and the uppermost Santa Rita-Assenta Member.

The Santa Rita-Assenta Member is essentially Tithonian aged, dominated by mudstones with frequent levels of caliche and intercalated by cross bedded sandstones (it may also include levels of bioclastic limestones, conglomerates and carbonaceous fragments; [74]). The Santa Rita-Assenta Member is also where the aforementioned remains of the stegosaurs from Areia Branca, Atalaia, Pedras Muitas and São Bernardino are probably original from (Fig 5A; [35,72,76]).

The Praia Azul Member is dated latest Kimmeridgian to earliest Tithonian and composed of mainly marls and mudstones with rare sandstone bodies deposited by a transitional system of shallow coastal plains, deltas, sandy bays and brackish lagoons. The holotype of *M*. *longicollum* (ML 433) came from the top unit of this member, the Miragaia Unit, likely early Tithonian aged [22,75]. Also from this member probably came the previously mentioned stegosaur remains from Vale de Pombas, Moçafaneira and Casal da Pedreira (Fig 5A; [35,41,43,76]).

The latest Kimmeridgian aged Praia da Amoreira-Porto Novo Member is composed of mudstones and sandstones deposited by upper fluvial meander with some marine influence, where the stegosaur remains from Alfeizerão, Murteiras and Porto Dinheiro were probably hailed from (Fig 5A; [35,72]).

The Lourinhã Formation is the Late Jurassic unit with the most abundant record of vertebrate fossils in Portugal and Europe, with a complex ecosystem as evidenced by the vast diversity of the fossil record [73]. The Lourinhã Formation has been repeatedly compared with the Morrison Formation in North America for their similarities in fauna, paleoenvironment, age and sedimentology (e.g., [21,73,78–81]). Although the proto-North Atlantic started expanding during the Hettangian, and by the Kimmeridgian and Tithonian when much of these formations were deposited it already separated Eastern North America and Iberia by more than 300 km, a regression during the Callovian-Oxfordian with eustatic sea-level drop and uplift resulted in ephemeral land-bridges connecting the two landmasses [80]. This resulted in exchange of macrovertebrate populations, including large dinosaur genera such as *Torvosaurus* Galton and Jensen, 1979 [82], *Allosaurus* Marsh, 1877 [83], *Ceratosaurus* Marsh, 1884 [84], *Supersaurus* Jensen, 1985 [85] and *Stegosaurus*, which are exclusive to the Lourinhã and Morrison Formations, but show evidence of speciation between the American and Portuguese forms [80]. Most of the other taxa occurring in the Lourinhã Formation (particularly those with Iberian distribution) show European affinities (such as the dacentrurine stegosaurs) or even Asian and African affinities [56,73,80].

## Methods

The preparation was mostly executed by FC; when others assisted with preparation (acknowledged), the preparation was instructed and supervised by FC. The preparation took place in the Alfragide campus of Laboratório Nacional de Energia e Geologia (LNEG) from September 2015 to August 2017, in a total of approximately 3500 working hours. The preparation consisted of: removal of thicker matrix with chisels and hammers; removal of matrix adjacent to the fossilized bones with a pneumatic air scribe (model HW-70 by The Stone Company); consolidation (hardening) with Paraloid B72 solved at 5% with acetone; surface cleaning with acetone; gluing of broken elements and fragments with adhesives (Paraloid B72 solved at 50% with acetone and cyanoacrylate); filling and reconstruction of intermediate missing parts with epoxy putty. During the removal of the matrix and reconstruction, it was prioritized to retain the existing taphonomic condition of the fossils, so bones articulated by matrix in life or near life position were not separated, and only exceptionally were bones reconstructed with plaster. Bruno Pereira and OM undertook in 2009 some preliminary consolidation and gluing with Paraloid B72, solved respectively at 5% and 50% with acetone, and re-labelled some of the skeletal elements that could be safely identified to either the stegosaur specimen or *D*. *zbyszewskii*. The specimen had initially no collection number, so MG 4863 was ascribed to it during this study. Each skeletal element was ascribed an individual collection sub-number by adding a numbered suffix, roughly representing the order of preparation (see Table 1 for full collection list of MG 4863). Due to constraints in time and the availability of the preparation equipment, not all fossilized material could be fully prepared at the finishing of writing, so in the case of some of the skeletal elements (mainly dorsal vertebrae, parts of the pelvic girdle and some unidentified elements) their interpretation and description herein was limited. During preparation, MG 4863 was successfully separated from the holotype of the ankylosaur *Dracopelta zbyszewskii* at first by noticeable differences in anatomy, the matrix’s sedimentology and reaction to hydrochloric acid (HCl), and afterwards corroborated with studies of the mineralogy of the matrix through X-ray fluorescence spectrometry (XRF) and X-ray diffraction (XRD; see [68] for more details). The description of the specimen MG 4863 includes detailed photographs of the identified skeletal elements in ‘flattened dice’-like arrangement, and virtual tridimensional models of some selected bones (see S1–S6 Figs), which were also figured in matching orientations to the fossil photographs. The comparisons of MG 4863 were first and foremost made with stegosaurian holotypes, to allow valid discussion on classification, but some comparisons were also made with stegosaurs from Iberia (to comment on their identification) and with exceptionally complete stegosaur specimens with comparable skeletal elements. First-hand analysis and comparisons were made with the holotype of *M*. *longicollum* (ML 433), ML 433-A and all the stegosaurian material herein referred from Museu da Lourinhã (Lourinhã, Portugal) and Museu Geológico de Lisboa (Lisbon, Portugal).

## Results and discussion

### Systematics

DINOSAURIA Owen, 1842 [86]

ORNITHISCHIA Seeley, 1888 [87]

THYREOPHORA Nopcsa, 1915 [88]

STEGOSAURIA Marsh, 1877 [2]

STEGOSAURIDAE Marsh, 1880 [89]

DACENTRURINAE Mateus *et al*., 2009 [22]

#### Definition

From [22]: all stegosaurs more closely related to *Dacentrurus armatus* (Owen, 1875) [12] than to *Stegosaurus armatus* Marsh, 1877 [2].

#### Revised diagnosis

Modified from [22] and [17]. Dacentrurinae possesses the following synapomorphies: (i) centra of dorsal vertebrae wider than long; (ii) olecranon horn present on ulna; (iii) anterior end of anterior pubic process expanded dorsally; (iv) the supracetabular process of the ilium extends anteriorly beyond the anterior edge of the acetabulum (modified and reinterpreted from [30]).

The following characters are also found to be unique to dacentrurine stegosaurs and likely diagnostic: (v) fusion of some of the cervical ribs to para- and diapophyses of cervical vertebrae (modified from [22]); (vi) mid and posterior caudal centra wider than tall (reinterpreted from [18]); (vii) mid and posterior caudal centra taller than long (reinterpreted from [18]); (viii) mid and posterior caudal centra with deeply concave lateral sides (reinterpreted from [18]).

#### Comprising taxa

*Dacentrurus armatus* (Owen, 1875) [12], *Miragaia longicollum* Mateus *et al*., 2009 [22], and *Miragaia longispinus* (Gillmore, 1914) [33].

*Dacentrurus* Lucas, 1902 [25]

#### Diagnosis

As for type and only known species.

*Dacentrurus armatus* (Owen, 1875) [2]

#### Holotype

BMNH 46013, comprising three cervical vertebrae, 16 dorsal vertebrae, 11 caudal vertebrae, left humerus, radius, ulna, carpus and metacarpals, ilio-sacral block, both ischia and pubes, right femur, partial right tibia, calcaneum, metatarsals, a dermal plate, and a tail spine [9]. Only specimen referred to the species (*sensu* [9]).

#### Type locality and horizon

Swindon, Wiltshire, UK. Kimmeridge Clay, Kimmeridgian, Late Jurassic [1,9,27].

#### Synonymy

*Omosaurus armatus* Owen, 1875 [12]: 45

*Stegosaurus armatus* Lydekker, 1890 [90]: 251

*Dacentrurosaurus armatus* Hennig, 1925 [91]: 242

#### Revised diagnosis

Suggested revised diagnosis, modified from [9] with additional characters named herein. Differs from all other stegosaurs in possessing: (i) straight dorsal surface of the distal ischial shaft; (ii) anterior end of the prepubis expanded ventrally; (iii) apex of cervical neural spines expanded posteriorly; (iv) cervical transverse processes borne at mid height of the prezygapophyses.

*Miragaia* Mateus *et al*., 2009 [22]

#### Diagnosis

*Miragaia* possesses the following synapomorphies (with some characters reinterpreted and modified from [18]): (i) transverse processes present in all caudal vertebrae; (ii) neural arch of mid and posterior caudal vertebrae one third or less the heigth and width of the centrum; (iii) outline of mid and posterior caudal centra is apple-shaped, with deep excavation of the neural canal; (iv) lateral ossification of the posterior rim of the posteriormost caudal centra.

The following feature is probably also unique to the genus *Miragaia*: (v) two pairs of elongate posterior dermal tail spines (~90% of femoral length) with slender shafts.

#### Comprising taxa

*Miragaia longicollum* Mateus et al. (2009) [22] and *Miragaia longispinus* (Gillmore, 1914) [33].

*Miragaia longispinus* (Gillmore, 1914) [33]

#### Holotype

From [18]. UW 20503 (formerly UW D54; American Heritage Center, Samuel H. Knight Collection), the right femur and casts of the posterior pair of dermal caudal spines (other casts: USNM V 8036; United States National Museum, Washington DC, USA). Illustrations in [33] of posterior caudal vertebrae (Fig 67 in [33]), left ischium (Plate 25, Fig 4 in [33]), right femur (Fig 45.2 and 68 in [33]) and caudal spines (Fig 66 in [33]) plus photographs (UW AHC) of most of the original 42 vertebrae and *in situ* photographs of the sacrum still in the quarry, three anterior caudal centra, parts of both ilia and ischia, anterior process of left pubis, right femur, and two posterior pairs of dermal caudal spines.

#### Type locality and horizon

Approximately 2.4 km east of Alcova, near Alcova Reservoir in Natrona County, central Wyoming, USA. From unknown locality high in equivalent strata to the Brushy Basin Member, Morrison Formation, Kimmeridgian-Tithonian, Upper Jurassic [18].

#### Synonymy

*Stegosaurus altispinus* [33]: 81 (*nomen dubium*)

*Stegosaurus longispinus* [33]: 111

?*Kentrosaurus longispinus* [92]: 89

*Natronasaurus longispinus* [93]: 21 (*nomen dubium*)

*Natronasaurus longispinus* [94]: 8 (*nomen dubium*)

*Alcovasaurus longispinus* [18]: 185

#### Revised diagnosis

Modified from [18]. Differs from all other stegosaurs in possessing: (i) femoral condylar articular surface confined almost exclusively to the distal surface;

Likely also diagnostic: (ii) posterior dermal tail spines with subequal bases; (iii) posterior dermal tail spines widest at ~25% of length.

Also differs from Morrison *Stegosaurus stenops*, *S*. *ungulatus* and *H*. *mjosi* in having six pairs of sacral ribs [18].

#### Referred specimens

Possible referred specimen from [18]: the circular base of a large spike from the Morrison Formation at Griffin Ranch near Bone Cabin Quarry, Como Bluff, Wyoming, USA.

*Miragaia longicollum* Mateus et al., 2009 [22]

#### Holotype

ML 433, an almost complete anterior skeleton: partial skull, forelimbs, pectoral girdle, cervical series, two first dorsal vertebrae, ribs, and cervical osteoderms [22].

#### Type locality and horizon

Miragaia, Lourinhã, Portugal. Praia Azul Member, Lourinhã Formation, Upper Kimmeridgian-Lower Tithonian, Upper Jurassic [22].

#### Synonymy

Aff. *Dacentrurus armatus* [62]: 157

Aff. *Dacentrurus armatus* [56]: 230

*Dacentrurus longicollum* [95]: 64

*Dacentrurus longicollum* [17]: 3

*Dacentrurus* sp. [19]: 9

#### Revised diagnosis

Modified from [22]: Differs from other stegosaurs in the presence of the following autapomorphies: (i) anterior tip of the premaxilla is drawn into a point; (ii) anterolateral rim of the premaxilla projects ventrally; (iii) at least 17 cervical vertebrae; (iv) spinopostzygapophyseal lamina extending anteriorly from the epipophyses, accompanied medially by a pair of lower parallel ridges, passing laterally on the neural spine and culminating on an anterior projection on the base of the neural spine with a notch ventral to it (revised autapomorphy from [22]); (v) mid and posterior cervical and anterior dorsal neural spines with transversely expanded apices; (vi) paired, slightly inwardly convex, triangular cervical dermal plates with a notch and projection on the posterodorsal margin (revised autapomorphy from [22]); (vii) cervical neural spines are positioned over the anterior half of the centrum and become progressively more anteriorly positioned passing posteriorly on the cervical series; (viii) cervical transverse processes more than half the axial length of the centrum in all but the anteriormost cervical vertebrae; (ix) outline in lateral view of cervical prezygapophyses round posteriorly and straight anteriorly with an anterodorsal notch; (x) closed proximodorsal canal on the ribs of the first caudal vertebra; (xi) progressively more posteriorly inclined neural spines of anterior caudal vertebrae, inclined at less than 45º to the horizontal between Cd8 and Cd11; (xii) neural spine reduced to one fifth the height and width from the 10th to the 12th caudal vertebra, vestigial further posteriorly in the vertebral series; (xiii) presence of longitudinal cord-like ridges in the femur shaft, two posteriorly and one anterolaterally positioned, with distal bifurcation.

#### Referred specimens

MG 4863, a partially complete adult specimen; ML 433-A, a juvenile specimen consisting of two dorsal centra, three dorsal neural arches, both pubes and the left ilium [22].

#### Locality and horizon of MG 4863

Atouguia da Baleia, Portugal. Coordinates: 39°20'45"N, 9°18'58"W. Santa Rita Member (Bombarral beds), Lourinhã Formation. Tithonian, Late Jurassic.

### Description

#### Cranial skeleton

**Fig 6:**
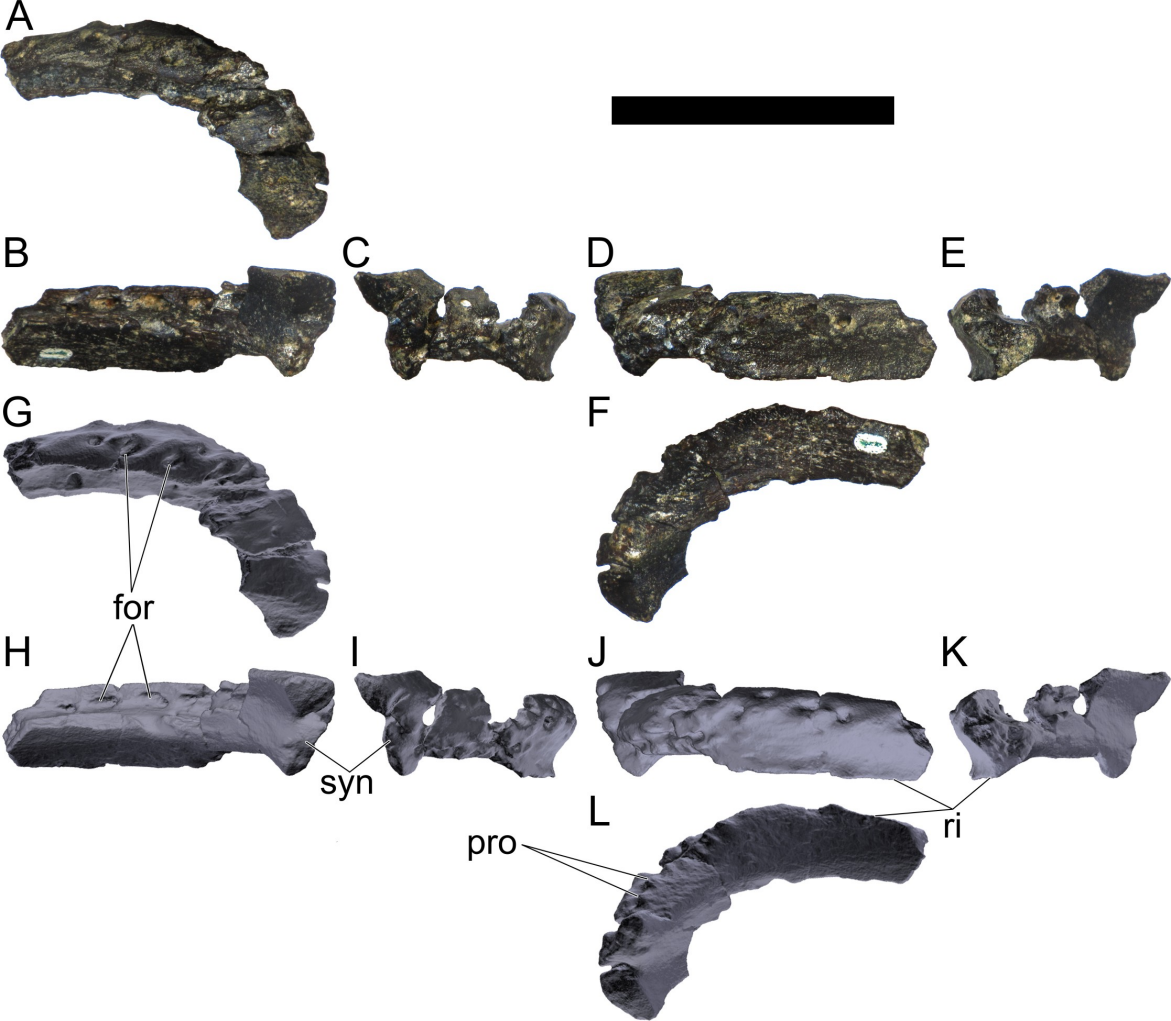
Dentary of *Miragaia longicollum* MG 4863. **A–F,** photographs, **G–L**, screenshots of 3D model in **A, G**, dorsal (or dorsolateral), **B, H**, medial (or dorsomedial), **C, I**, anterior, **D, J**, lateral (or ventrolateral), **E, K**, posterior, and **F, L**, ventral (or ventromedial) view. **For**, foramen, **pro**, process, **ri**, ridge, **syn**, symphysis. Scale bar equal to 5 cm. **G–L**, published under a CC BY license, with permission from Marco Marzola, original copyright 2017.

MG 4863–1 is most probably an anterior fragment of the left dentary (Fig 6; 3D model provided in S1 Fig.). It includes the symphysis surface and the ventral margin, but it is missing all the dorsal surface (apart for adjacent to the symphysis), retaining therefore no dental alveoli or teeth. Other smaller fragments (MG 4863–60) are possibly more posterior sections of the dentary, fragments of the predentary or other cranial bones, but these are too fragmentary and featureless to conclusively identify (at least without direct comparisons with the cranial bones of other stegosaurs). A series of small and round foraminae are present in the ventral side of the dentary (Fig 6A, 6B, 6G and 6H), presumably for nutrient vessels for each tooth [96,97]. These are more tightly packed close to the anterior end and often are separated by small processes in between (where the horny rhamphotheca would be covering; [1]; Fig 6F and 6L). While the dorsal section of the dentary is mostly absent, the remaining ventral section reveals the floor of a longitudinal channel (Fig 6B and 6H), apparently deep inside the dentary when complete to house the replacement teeth and the roots of the protruding teeth. The symphyseal facet is reniform, with the convex side facing laterally and clefted transversely in its middle (Fig 6B, 6C, 6H and 6I). The medial side of the dentary appears to be longitudinally concave. The ventral margin evidences a low but sharp longitudinal ridge (Fig 6D, 6E, 6J and 6K). The anterior end curves medially from the rest of the dentary.

**Fig 7:**
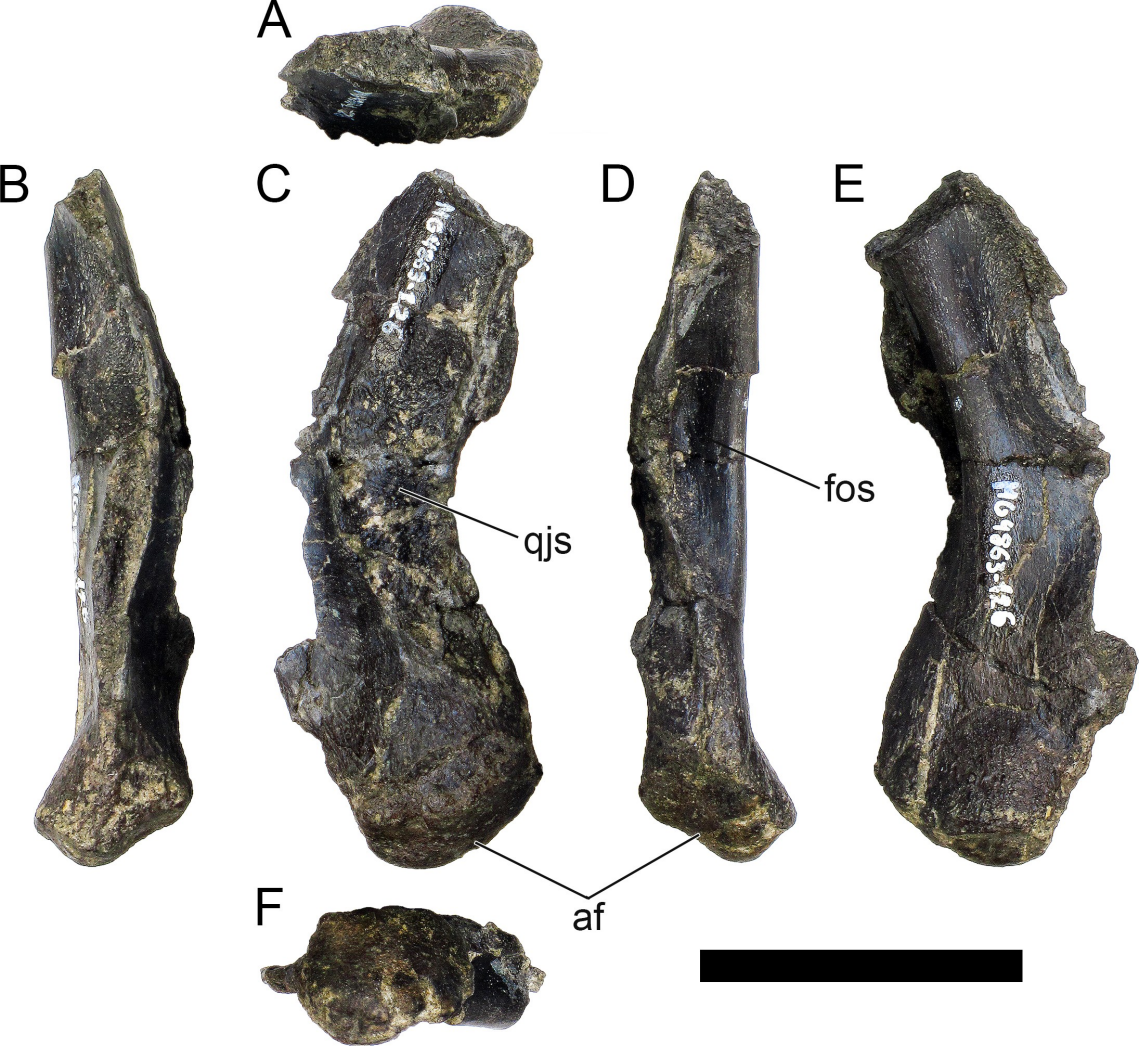
Quadrate of *Miragaia longicollum* MG 4863. **A**, dorsal, **B**, anterior, **C**, lateral, **D**, posterior, **E**, medial, and **F**, ventral view. **Af**, articular facet, **fos**, fossa, **qjs**, scar of the quadratojugal. Scale bar equal to 5 cm.

MG 4863–126 is the incomplete left quadrate (Fig 7). It is missing the dorsal end that would meet with the squamosal and paraoccipital (the “quadrate head”) and most of the anterior margin (*i*.*e*., the pterygoid flange). Part of the posterolateral margin that was covered by the quadratojugal is also missing (Fig 7C and 7D. The partial quadrate is approximately 110 mm tall, being probably about 150 mm when complete (as estimated by comparing its shape and position of the scar of the quadratojugal with *S*. *stenops*, *Kentrosaurus aethiopicus* Hennig, 1915 [98] and *Huayangosaurus taibaii* Dong *et al*., 1982 [99]; [33,97,100]). It is tall, relatively thick, more compressed transversely than axially, widest at the ventral articular face with the jaw. The scar surface for the quadratojugal is present, showing that the quadratojugal was positioned just dorsally to the articular facet of the quadrate (Fig 7C). The ventral articular facet is subrectangular in outline in ventral view (Fig 7F). In posterior view, the margin of the articular facet is obliquely oriented with the main shaft of the quadrate, angling ventromedially at approximately 35 degrees (*i*.*e*., the medial side of the articular face is the most ventral part of the quadrate; Fig 7B and 7D). The ventral articular facet is strongly convex medially and slightly convex laterally in axial view (Fig 7B and 7D). A pronounced longitudinal fossa is present in the posterior face of the quadrate (Fig 7D), about half as tall as the preserved quadrate–the posterolateral lamina besides it is thinner, sharper and extends further posteriorly than the posteromedial edge (Fig 7D). In lateral and medial view, the posterior margin gently curves posteriorly in a round curve (Fig 7C and 7E).

#### Cervical vertebrae and ribs

Ten semi-complete cervical vertebrae were found, including the axis and three vertebrae posteriorly articulated to it by matrix, two isolated mid cervical vertebrae and a sequence of four articulated mid-posterior cervical vertebrae. Some cervical fragments were also found, that can safely be ascribed to missing vertebrae. The relative positions of the cervical vertebrae were estimated by relative size and comparisons with ML 433 (considering that in ML 433 and other stegosaurs these get proportionately larger posteriorly along the neck; [22,19]). Despite this, the possibility that there was at least one more cervical vertebra not accounted for between Cv5 and Cv10 is not ruled out, which would move the posterior cervical vertebrae up one position. All cervical vertebrae suffered some considerable amount of deformation, as such all centra were somehow reshaped and many parts of the neural arches were broken. The description of the cervical vertebrae and cervical ribs are herein done together as these are fused (common state to dacentrurine stegosaurs), therefore should be treated, effectively, bas one osteological unit.

It is possible to observe that the centra are longer anteroposteriorly than transversely wide or tall dorsoventrally. The most anterior centra are about as tall as wide, becoming wider progressing posteriorly. The central articulations are almost round in the anterior vertebrae and become more ellipsoid passing posteriorly. The centra are amphicoelous and usually more excavated on the posterior articulation. The centrum wall is smooth at mid length but shows rugosities in both posterior and anterior rims. A ventral keel in the centra as described in other stegosaurs [19] is not visible, instead the centra evidence a flattened base with a pair of symmetrical tuberosities on the ventral side of the centrum rims, each positioned lateroventrally. The anterior pair of tuberosities is more developed and enlarged. The centra have markedly concave sides in lateral and ventral views, which give them an hourglass outline in these views. There are no foramina or pleurocoels visible.

The diapophyses, parapophyses and ribs form a closed canal that runs sagittally and parallel to the neural canal. While the cervical ribs are always seamlessly fused to the vertebrae through the parapophyses, a contact suture or partial fusion may be evident between the most anterior diapophyses and ribs. The diapophyses and parapophyses have subround facets in the most anterior vertebrae, but become longer anteroposteriorly and less tall dorsoventrally passing posteriorly. As such, the transverse processes are subtriangular in dorsal view in the most anterior vertebra and sub-rectangular in the other cervical vertebrae. The parapophyses are placed on the anterior half of the centrum, mostly retain a mid-height position from the centrum and project laterally. The body of the transverse processes projects ventrolaterally and beyond the lateral margins of the centrum. The transverse processes are long, proximally they are about double the length of the prezygapophyses, extending anteroposteriorly over all the anterior half of the centrum–that is, from mid-length of the prezygapophyses and confluent with the anterior margins of the pedicels, to beyond the posterior margin of the neural spine–and their bases sprout level with the ventral margin of the prezygapophyses. Both the anterior and posterior margins of the transverse processes evidence a series of varied and irregular processes projecting, respectively, anteriorly and posteriorly.

The neurocanal is taller than wide, about twice as tall (absolute measurements are not possible since all neurocanals are somewhat crushed), with a round top and flat horizontal floor. The pedicels are thin, a few millimeters thick, and insert in almost all length of the centrum, particularly anteriorly. There is no distinct neurocentral suture in any cervical vertebra.

The prezygapophyses project anterodorsally and beyond the anterior face of the centrum. The prezygapophyseal articular facets face dorsomedially. The outline in lateral view of the prezygapophyses is equal to what is observable in the cervical prezygapophyses of ML 433 [22]: the posterodorsal margin is strongly curved, while the anterior margin is almost straight, facing anterodorsally and bearing a slight notch, so the anteriormost projection of the prezygapophysis is the most ventral part. The posterior edges of the prezygapophyses overlap or reach as far as the anterior edge of the neural spines in lateral view in mid and posterior cervical vertebrae. A wide interprezygapophyseal shelf, that roofs the neural canal, separates the bases of the prezygapophyses.

The postzygapophyses are tongue-shaped and project posteriorly beyond the posterior centrum face, as far as the postzygapophyseal facets fully surpassing it in mid and posterior vertebrae. The postzygapophyses are bridged by an interpostzygapophyseal lamina and separated posteriorly by a round concavity in dorsal view, that becomes less pronounced progressing posteriorly. The postzygapophyseal articular surfaces face ventrolaterally and are oval, longer than wide. The postzygapophyseal facets form between them a “V” shaped angle in posterior view, becoming more vertical progressing posteriorly. Epipophyses are present in the postzygapophyses, and a spinopostzygapophyseal lamina extends anteriorly from the epipophyses, passing laterally on the neural spine and culminating on the anterior projection on the base of the neural spine. This projection is the anteriormost point of the neural spine, it is present on mid and posterior cervical vertebrae, as well as a notch ventrally to it. Medial to the spinopostzygapophyseal laminae is a pair of parallel accessory crests or low laminae that also pass laterally on to the sides of the neural spines, and medial to these a deep fossa is present posteriorly to the neural spine (which becomes deeper passing posteriorly within the cervical series).

The neural spines are transversely narrow ridges on the anterior cervical vertebrae, with a round dorsal outline in lateral view, increasing in size passing posteriorly. The most anterior neural spines are positioned mid-length to the centrum and about equidistant to the pre- and postzygapophysis. In mid and posterior vertebrae, the neural spine becomes progressively wider, taller and more anteriorly positioned (i.e., closer to the prezygapophyses and the anterior edge of the centrum than to the postzygapophyses and the posterior centrum articulation).

The ribs project posteriorly and beyond the centrum. These are subtriangular in section at mid length, with longitudinal grooves medially and laterally, and become rounder passing posteriorly, tapering in a blunt end. The lateral groove is bordered by a lateral ridge which, combined, form a nest for the previous rib shaft to settle. The cervical ribs possess an additional anterior process from the capitulum that projects anteriorly beyond the anterior articular face of the centrum. A much smaller anterior process is also observable from the tuberculum.

Measurements of all cervical vertebrae are given in Table 2.

**Table 2:** Measurements of vertebral elements of *Miragaia longicollum* MG 4863. **Cd,** caudal vertebrae; **Cv**, cervical vertebrae; **D**, dorsal vertebrae. Values represent the maximum measurement. (), lower than real measurement (mostly due to broken ends). *, estimated measurement. Values in mm.

	Total height	Centrum Length	Anterior centrum height	Posterior centrum height	Posterior centrum width
Axis	96*	87*	-	50*	-
Cv3	101*	90	-	55*	-
Cv4	97	93	49*	54	62
Cv5	-	-	54	-	-
Cv6	-	-	-	-	-
Cv7		114	(48)	(52)	95*
Cv9	130*	126	71	74	88
Cv10	-	-	-	-	-
Cv11	(95)	126	(61)	-	106*
Cv12	(97)	128	(64)	(66)	118*
Cv13	(118)	138	(58)	(77)	106*
Cv14	(160)	133*	80*	92*	(95)
Cv17	-	-	-	-	-
D2	(230)	96	93*	99	102
D3	(240)	90	-	99*	104*
D5	360	75*	95	-	-
D6	(260)	70*	-	100*	105
D7	(280)	79	94*	99	100*
Cd1	(265)	68	85	95	110
Cd2	(200)	64	87	93	117
Cd3	(153)	70	(85)	(80)	105*
Cd4	-	-	-	-	-
Cd5	(210)	52	85	88	12
Cd7	(132)	69	92	98	106
Cd8	280	71	100*	-	100
Cd9	260*	73	-	95*	102
Cd10	(190)	76	95	92	98
Cd11	121	76	89	95	98
Cd12	153	76	85	80	91
Cd13	150*	73	-	86	83
Cd14	(112)	70	-	71	95
Cd15	120*	68	80	82	92
Cd16	(94)	70	83	83	93
Cd18	110	63	75	76	95
Cd19	103	63	74	74	93
Cd23	(67)	56	67	66	88
Cd24	88	53	66	64	83
Cd27	84*	51	63	64	74
Cd28	(64)	50	62	63	76
Cd30	75*	48	61	60	78
Cd32	(70)	45	57	56	72
Cd34	66	44	54	52	68
Cd36	(53)	41	53	47	64
Cd37	55*	40	47	44	60

**Fig 8:**
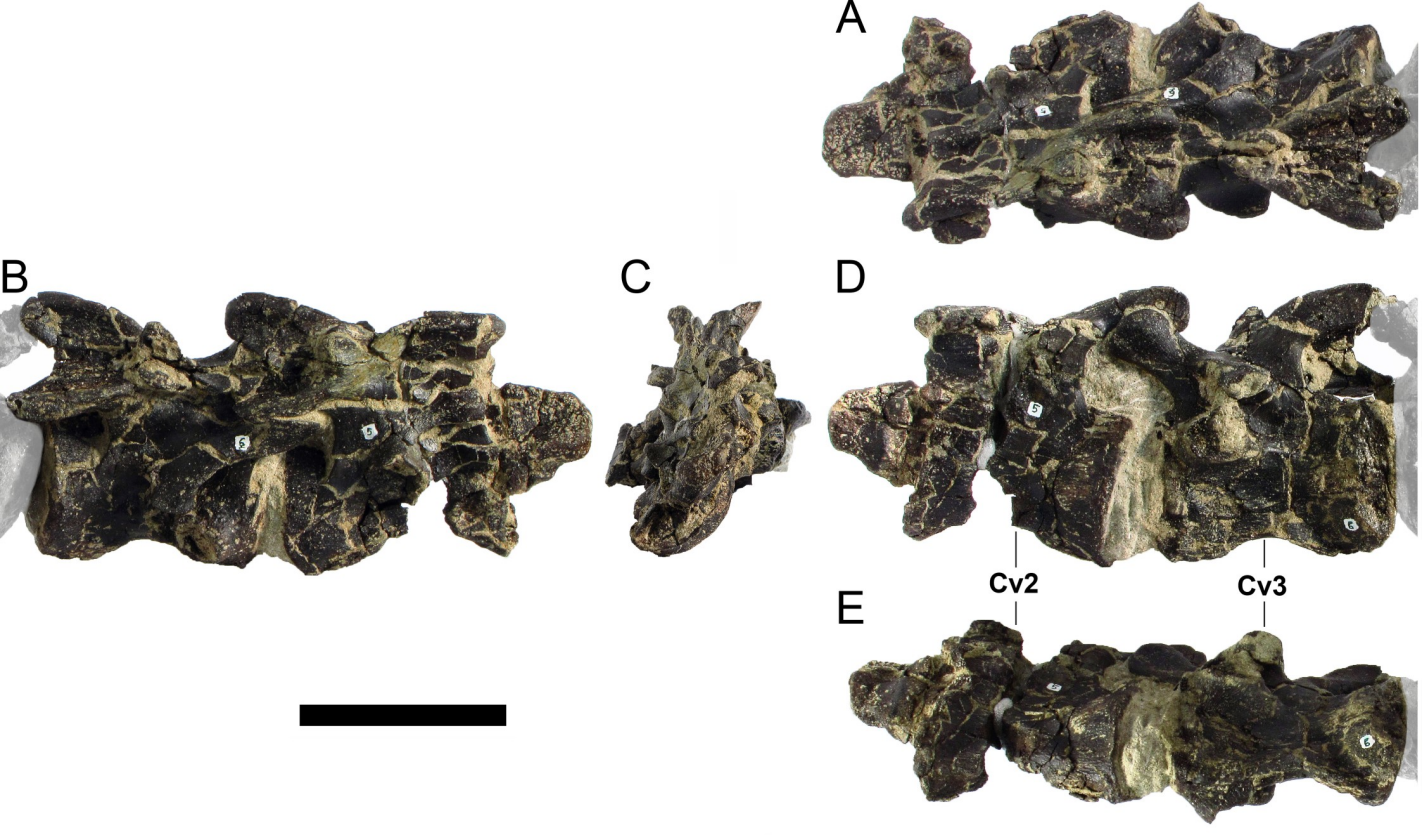
Cervical vertebrae two (axis) and three of *Miragaia longicollum* MG 4863 (photographs). **A**, right dorsolateral, **B,** right lateral, **C,** anterior, **D,** left lateral, and **E,** ventral view. **Cv2**, axis, **Cv3**, cervical vertebra three. Scale bar equal to 10 cm.

**Fig 9:**
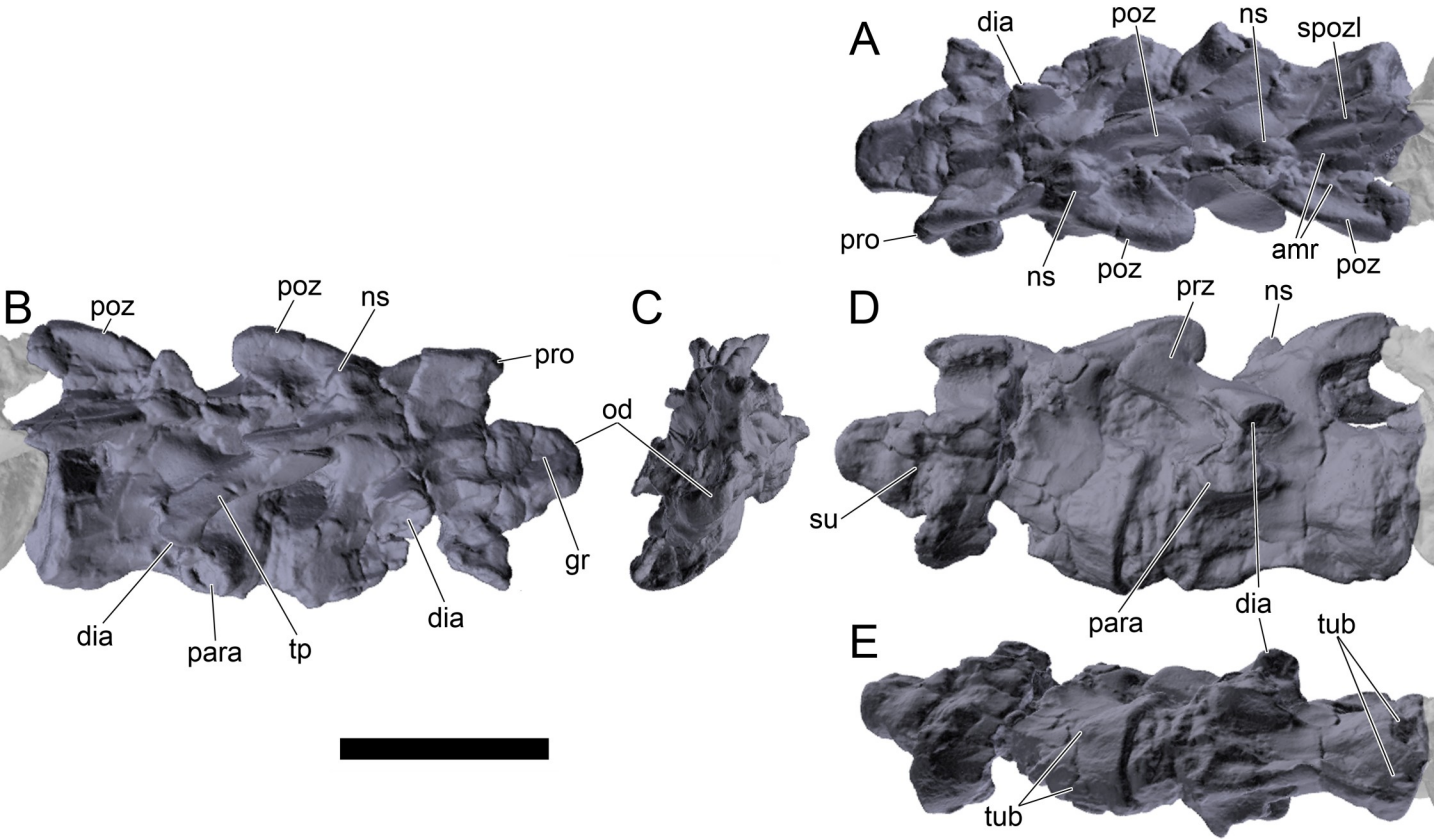
Cervical vertebrae two (axis) and three of *Miragaia longicollum* MG 4863 (screenshots of 3D model). **A**, right dorsolateral, **B**, right lateral, **C**, anterior, **D**, left lateral, and **E**, ventral view. **Amr**, assessory medial ridges of spinopostzygapophyseal laminae, **dia**, diapophysis, **gr**, groove, **ns**, neural spine, **od**, odontoid process, **para**, parapophysis, **poz**, postzygapophysis, **pro**, process, **spozl**, spinopostzygapophyseal lamina, **tub**, tuberosities. Scale bar equal to 10 cm. Published under a CC BY license, with permission from Marco Marzola, original copyright 2017.

Axis (MG 4863–5; Figs 8 and 9; 3D model provided in S2 Fig): The axis is mostly complete but severely compressed and flattened transversely, including several cracks and a complete rotation of the top of the neural arch to the right side, so some details may not be clearly observable. It is connected posteriorly with Cv3 in near articulation, having been compressed to the point that the postzygapophyses of the axis are positioned posteriorly beyond the prezygapophyses of Cv3. The odontoid process evidences a suture between it and the centrum, so it is not fully fused to the axis (Figs 8D and 9D). The odontoid process is positioned at mid height of the anterior central articular facet, round in outline in lateral view and about as tall dorsoventrally as it is long axially (Figs 8D, 9B and 9D). A broad and shallow anteroposterior groove is present on the dorsal side of the odontoid process, continuous with the floor of the neural canal (repositioned in the right side by deformation; Figs 8A, 8B and 9B). The ventral margin of the axis is concave in lateral view, as are the sides in ventral view (Figs 8D, 8E, 9D and 9E). A pair of ventroposterior tuberosities is visible in the ventral side of the centrum, alike those observed in more posterior cervical vertebrae (Figs 8D and 9E). The parapophyses are mostly broken, but the left one appears to be a low ridge, 30 mm long and 10 tall positioned laterally. The right diapophysis is short transversely and anteroposteriorly (the left one is broken), with a round facet facing lateroventrally (Figs 8A, 8B, 9A and 9B). The diapophysis is positioned in the lateral center of the pedicels. The neural arch appears to have a straight and almost horizontal dorsal margin, but this may have been exaggerated by deformation and could have been steeper in life (Fig 8B and 8C). A small and round anterior process projects just ventrally to the dorsal edge of the axis, slightly beyond the anterior margin of the centrum (Figs 8A, 8B and 9A). The neural spine is low and wide and positioned two thirds the length posteriorly of the centrum (Figs 8A, 8B, 9A and 9B). The postzygapophyses are in most respects similar to more posterior cervical postzygapophyses, but the spinopostzygapophyseal laminae are not clearly present and the epipophyses do not extend as much posteriorly and are lower (Figs 8A, 8B and 9A). The postzygapophyses appear to project quite more posteriorly and dorsally than the neural spine, but this may have been exaggerated by deformation.

**Fig 10:**
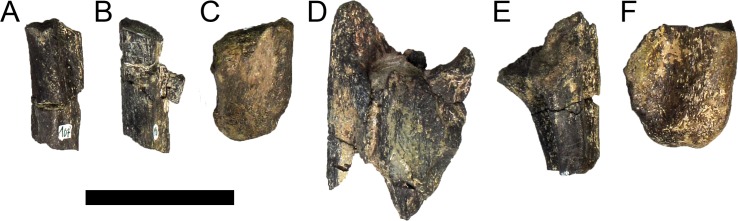
Cervical fragments of *Miragaia longicollum* MG 4863. **A**, MG 4863–107, **B**, MG 4863–110, **C**, MG 4863–108, **D**, MG 4863–104, **E**, MG 4863–105, **F**, MG 4863–106, **A–F** in dorsal view, anterior is upwards. Scale bar equal to 5 cm.

3rd cervical vertebra (MG 4863–6; Figs 8 and 9; 3D model provided in S2 Fig): The vertebra is missing both cervical ribs and was severely flattened and deformed laterally, including an almost full rotation of the top of the neural arch to the right side (Figs 8A, 8B and 9A). It is partially articulated anteriorly to the axis and posteriorly to Cv4. The centrum was flattened transversely so that some anatomical details are unclear, but it is possible to observe the same pair of ventroposterior tuberosities found in other cervical vertebrae (Fig 8). The diapophyses and the parapophyses appear to not have been completely fused to the missing ribs, unlike more posterior cervical vertebrae that evidence a seamless fusion between the parapophyses and the cervical rib (Figs 8B, 8D, 9B and 9D). The facets of the diapophyses and parapophyses are subround, only slightly longer than tall. The transverse processes extend from posteriorly to the prezygapophyses to level with the posterior margin of the neural spine, and since the diapophyseal facets are anteroposteriorly short, the transverse processes in dorsal view have a subtriangular shape (Figs 8A, 8B, 9A and 9B). The prezygapophyses extend anterodorsally over the centrum of the axis on long peduncles (Figs 8D and 9D). In lateral view the prezygapophyses have a similar morphology to more posterior prezygapophyses–round in the dorsal margin, with an anteroventral projection and a gentle notch at the anterodorsal margin. The neural spine is relatively sharp and taller than the one in Cv4, but this is probably exaggerated by deformation (Figs 8A, 8D, 9A and 9D). The postzygapophyses are long, have oval shaped facets and a round concavity in between separating them in dorsal view (Figs 8A, 8B, 9A and 9B). The spinopostzygapophyseal laminae are not easily noticeable, but are present, along with the accessory pair of smaller medial ridges (Figs 8A, 8B, 9A and 9B). The medial part of a right cervical rib (MG 4863–107; Fig 10A) probably belongs to this vertebra.

**Fig 11:**
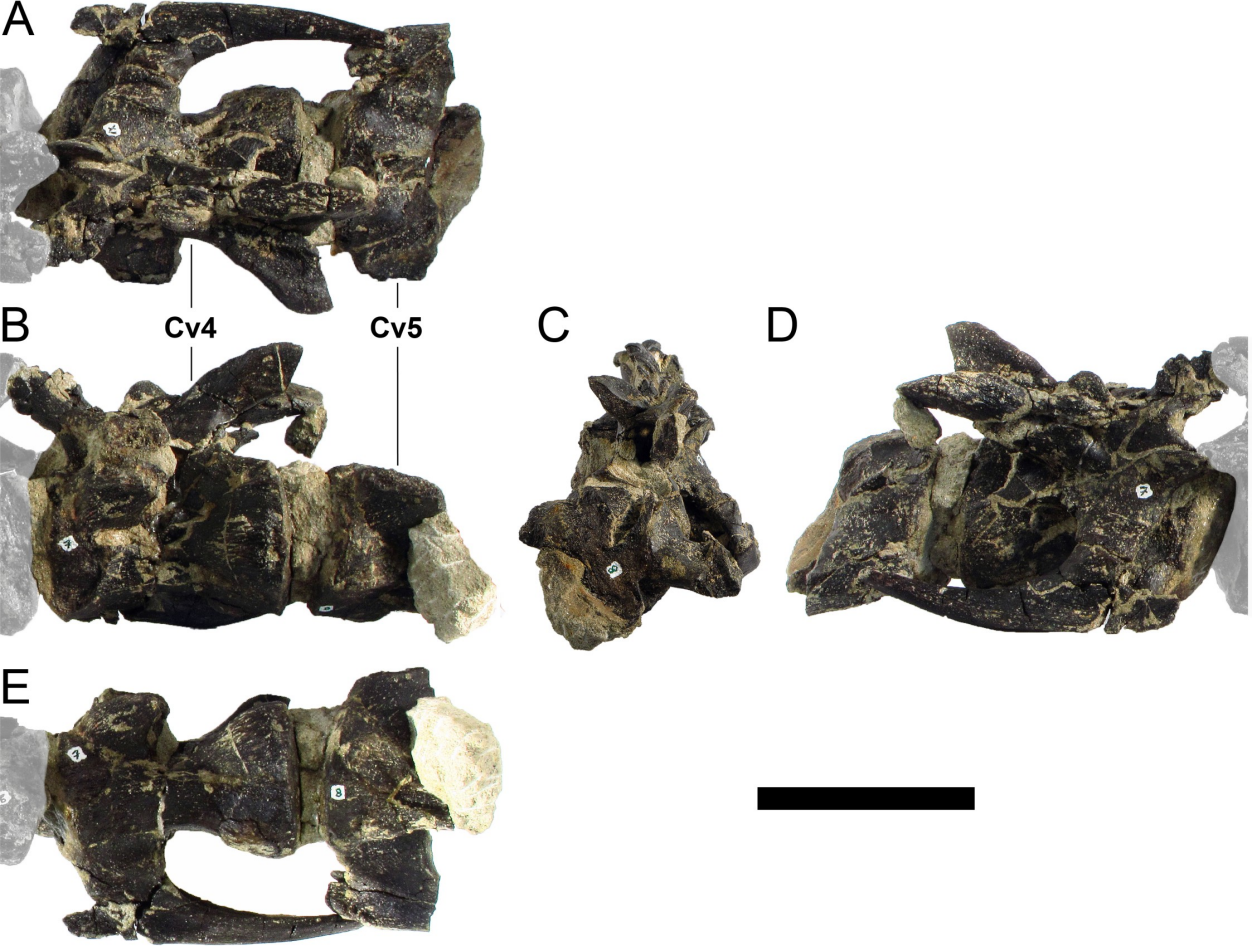
Cervical vertebrae four and five of *Miragaia longicollum* MG 4863 (photographs). **A,** dorsal, **B**, left lateral, **C**, posterior, **D**, right lateral, and **E**, ventral view. **Cv4**, cervical vertebra four, **Cv5**, cervical vertebrae five. Scale bar equal to 10 cm.

**Fig 12:**
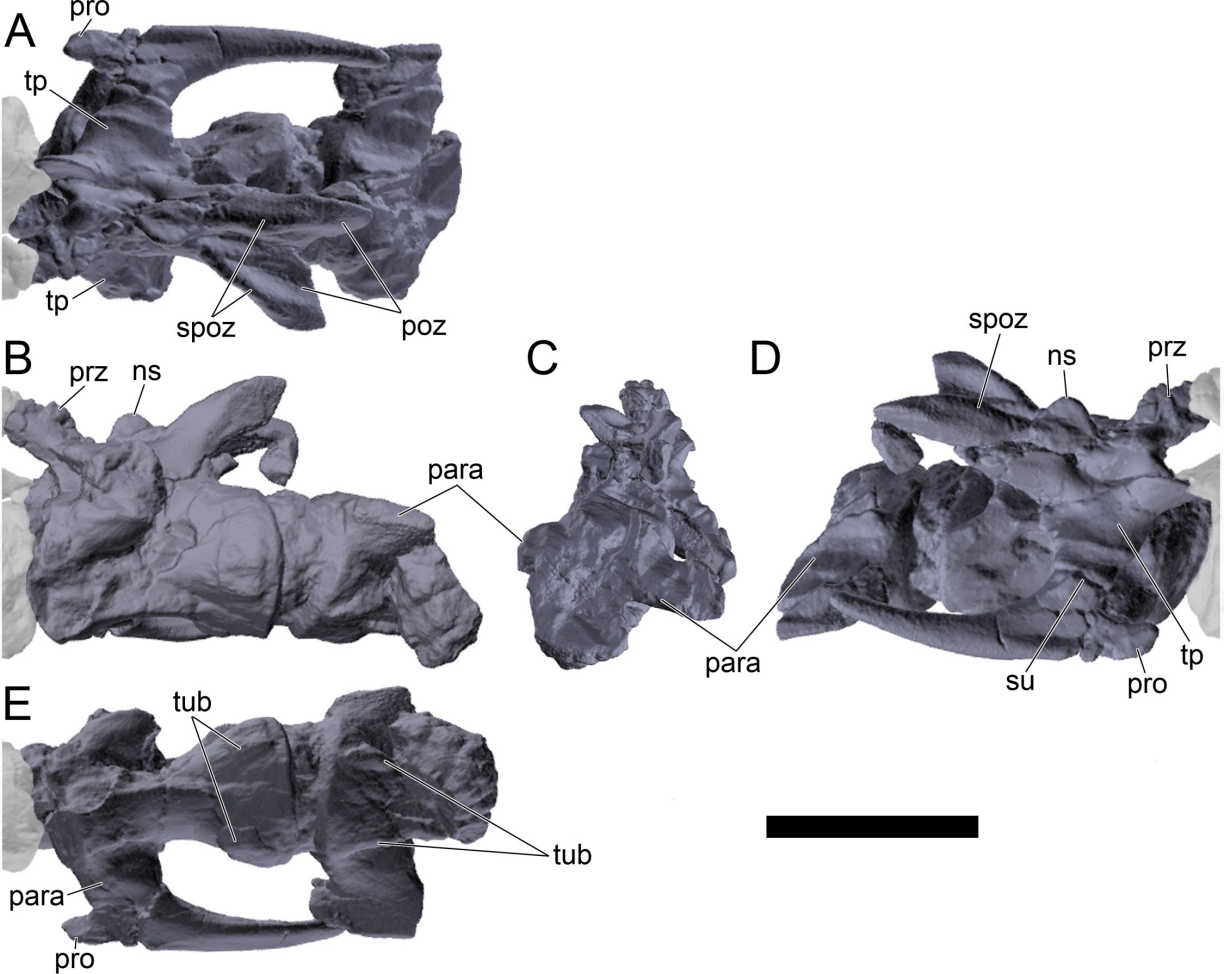
Cervical vertebrae four and five of *Miragaia longicollum* MG 4863 (screenshots of 3D model). **A**, dorsal, **B**, left lateral, **C**, posterior, **D**, right lateral, and **E**, ventral view. **Ns**, neural spine, **para**, parapophysis, **poz**, postzygapophysis, **prz**, prezygapophysis, **spozl**, spinopostzygapophyseal lamina, **su**, suture, **tp**, transverse process, **tub**, tuberosities. Scale bar equal to 10 cm. Published under a CC BY license, with permission from Marco Marzola, original copyright 2017.

4th cervical vertebra and ribs (MG 4863–7; Figs 11 and 12; 3D model provided in S2 Fig): Cv4 is missing the left cervical rib and is laterally deformed, but not as much as Cv3. It is articulated by matrix anteriorly with Cv3 and posteriorly with Cv5. The anterior half of the centrum is flattened transversely, but the posterior end is mostly unaltered, evidencing a round outline and a pair of ventroposterior symmetrical tuberosities (Figs 11D and 12E). The centrum walls are concave, evidencing excavation in ventral and lateral view (Figs 11B, 11E and 12B). The cervical ribs are fused seamlessly with the parapophyses while the diapophyses evidence fusion suture with the ribs (Figs 11D, 11E, 12I and 12E). The transverse processes are long anteroposteriorly, much longer than those in Cv3, extending from about mid-length of the prezygapophyses to level with the posterior margin of the neural spine (so more akin to mid and posterior cervical vertebrae than Cv3) and are trapezoidal in shape in dorsal view (Figs 11A, 11D, 12A and 12D). The transverse process is also longer than in Cv3, with the diapophyseal facet about twice as long than tall and not fully fused to the rib. The floor of the neural canal is flat and horizontal. The neural canal was constricted transversely, but was evidently large, about two thirds the height of the centrum and almost as wide. In lateral view the posterior margin of the pedicels form a round concavity (Figs 11B and 12B).

The prezygapophyses extend anterodorsally beyond the anterior facet of the centrum, but not as much as in Cv3 (Figs 11B, 11D, 12B and 12D). The neural spine is small but distinct, round in lateral view, bulging dorsally like a thick small coin (Figs 11B, 11D, 12B and 12D). The spinopostzygapophyseal laminae are more distinct than in preceding vertebrae and are particularly wide and thick on the sides of the neural spine (Figs 11A, 11D, 12A and 12B). The shaft of the cervical rib curves gently medially and dorsally, extends over the anterior process of the next cervical rib and has a well-developed and flat anterior process (Figs 11A, 11D, 12A and 12D). In all other visible respects, it is similar to Cv3.

5th cervical vertebra and ribs (MG 4863–8; Figs 11 and 12; 3D model provided in S2 Fig): This vertebra includes only the anterior half of the centrum and the tip of the right prezygapophysis. It is less deformed than preceding cervical vertebrae, with a round centrum and aligning parapophyses, but the remains of the neural canal evidence some deformation (Figs 11C and 12C). It has a clearly distinguishable pair of anterior tuberosities on the ventral side of the centrum and no keel (Figs 11E and 12E). In all other respects, it is similar to the preceding vertebrae and offers no additional anatomical details. The posterior breakage surface evidences an apparently fresh cut, so possibly more posterior material of this vertebra may be found in the future. The medial section of a right cervical rib (MG 4863–110; Fig 10B) may be part of this vertebra or of Cv6.

6th cervical vertebra (MG 4863–108; Fig 10C): Only the right postzygapophysis of this vertebra was found, and since it is much larger than the one in Cv4 and slightly smaller than the one in Cv7, it is identifiable as pertaining to the 6th cervical vertebra. Apart for the size, it is virtually identical to the one in Cv7.

**Fig 13:**
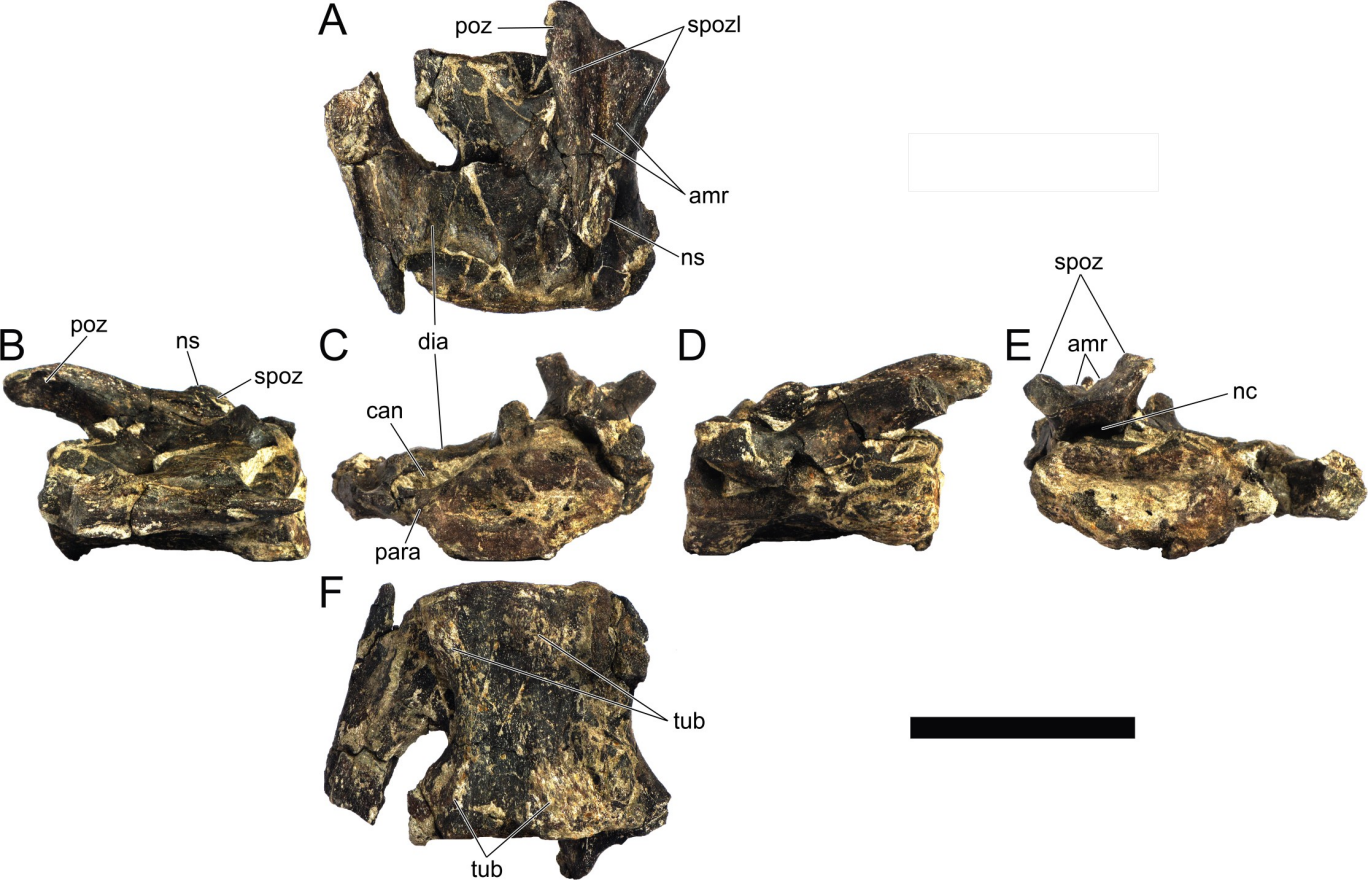
Cervical vertebra seven of *Miragaia longicollum* MG 4863. **A**, dorsal, **B**, right lateral, **C**, anterior, **D**, left lateral, **E**, posterior, and **F**, ventral view. **Amr**, assessory medial ridges of spinopostzygapophyseal laminae, **can**, canal, **dia**, diapophysis, **nc**, neural canal, **ns**, neural spine, **para**, parapophysis, **poz**, postzygapophysis, **spozl**, spinopostzygapophyseal lamina, **tub**, tuberosities. Scale bar equal to 10 cm.

7th cervical vertebra and ribs (MG 4863–9; Fig 13): Cv7 is missing the left cervical rib, the posterior half of the right cervical rib, the prezygapophysis and the tip of the left postzygapophysis. It is severely flattened dorsoventrally, with a flattened centrum, neural arch sheared to the left and neural canal collapsed against the centrum. The cervical rib posteriorly is abnormally large for the size of the vertebra, larger than all more posterior cervical ribs, and includes an irregular dorsal projection, probably due to a pathology.

The centrum is amphicoelous, twice as wide than tall, and 1.4 times longer than wide. The articular facets are oval-shaped with marked horizontal grooves in the center of both where the concavities converge (Fig 13C and 13E). The centrum wall is mostly smooth, apart for two pairs of symmetrical bulges with rugosities, one anteroventrally and one posteroventrally (Fig 13F). A cylindrical shape of the centrum is perceivable, with an hourglass outline in ventral view, but this may have been exaggerated by the deformation forces (Fig 13F). The parapophysis are placed on the anterior half of the centrum and at mid-height of the centrum, projecting laterally and fusing to the ribs (Fig 13C and 13F). The fusion sutures of the rib with the diapophyses and parapophyses are not visible (Fig 13A). The diapophyses, parapophyses and rib form a closed canal that runs horizontally parallel to the neural canal (Fig 13C). There are no foramina or pleurocoels visible. Although collapsed, it is possible to observe a flat floor and curved roof of the neural canal (Fig 13E). The pedicels are thin and insert in almost all length of the centrum (Fig 13B and 13D). The neural arch is about as tall as the centrum. The postzygapophyseal facet is round and projects halfway past the posterior articulation of the centrum (Fig 13A, 13B and 13F). The interprezygapophyseal lamina roofs the neural canal and does not extend anteriorly beyond the centrum facet (Fig 13A). The interpostzygapophyseal lamina extends beyond the centrum and forms a concave rim in dorsal view (Fig 13A). Both medial and lateral distal edges of the postzygapophyses are directed horizontally in lateral view (Fig 13B). The spinopostzygapophyseal laminae are thick and well visible and connected to the side margins of the neural spine, together with two accessory parallel adjacent crests that run posteriorly to the neural spine (Fig 13A, 13B and 13E). The tip of the neural spine is not expanded and has a gently curved outline in lateral view (Fig 13B and 13D). The neural spine is low, narrow and axially longer than wide or tall (Fig 13A and 13B). The presence of an anterior notch and tip in the neural spine as in other cervical vertebrae is not possible to determine since this area is broken and missing.

**Fig 14:**
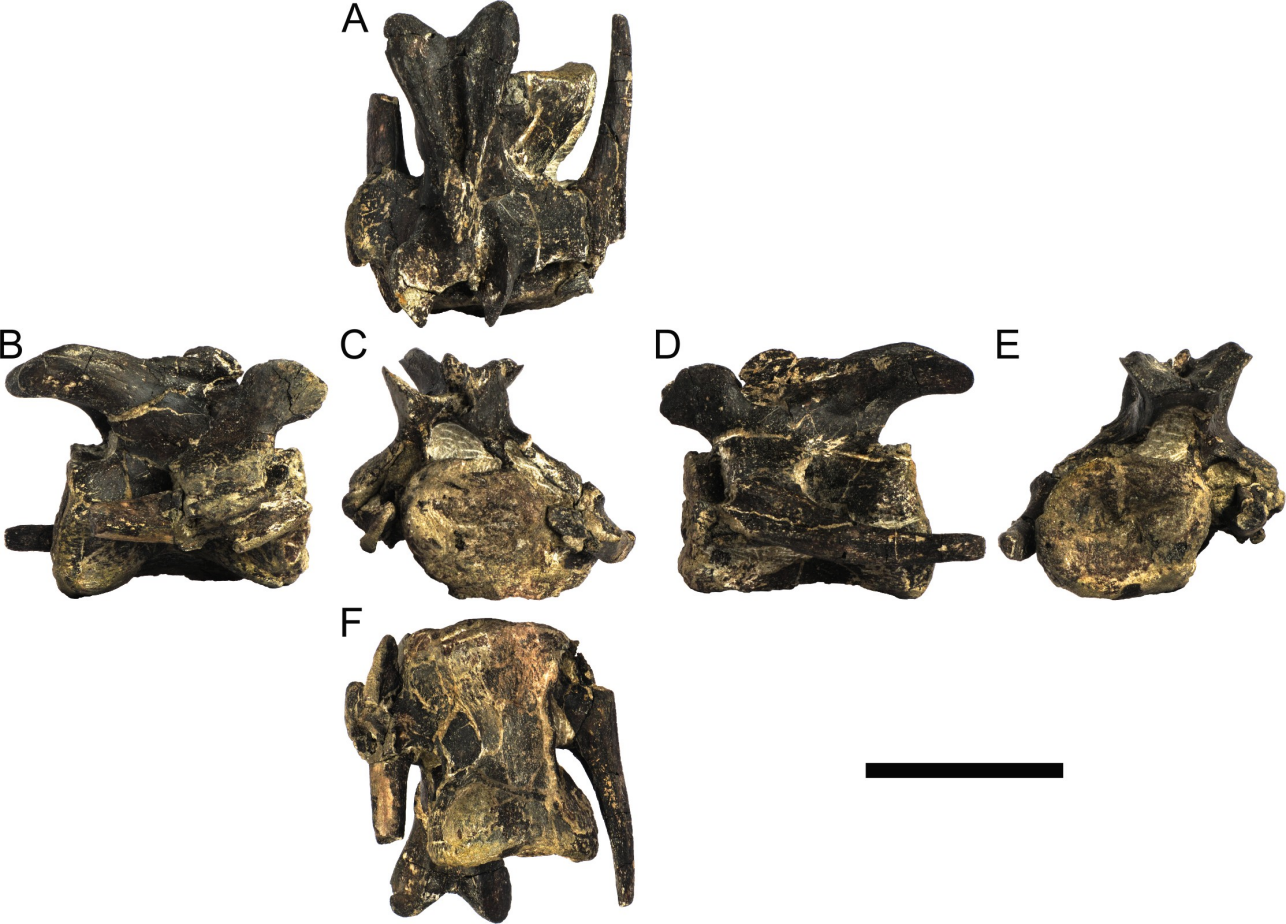
Cervical vertebra nine of *Miragaia longicollum* MG 4863 (photographs). **A**, dorsal, **B**, right lateral, **C**, anterior, **D**, left lateral, **E**, posterior, and **F**, ventral view. Scale bar equal to 10 cm.

**Fig 15:**
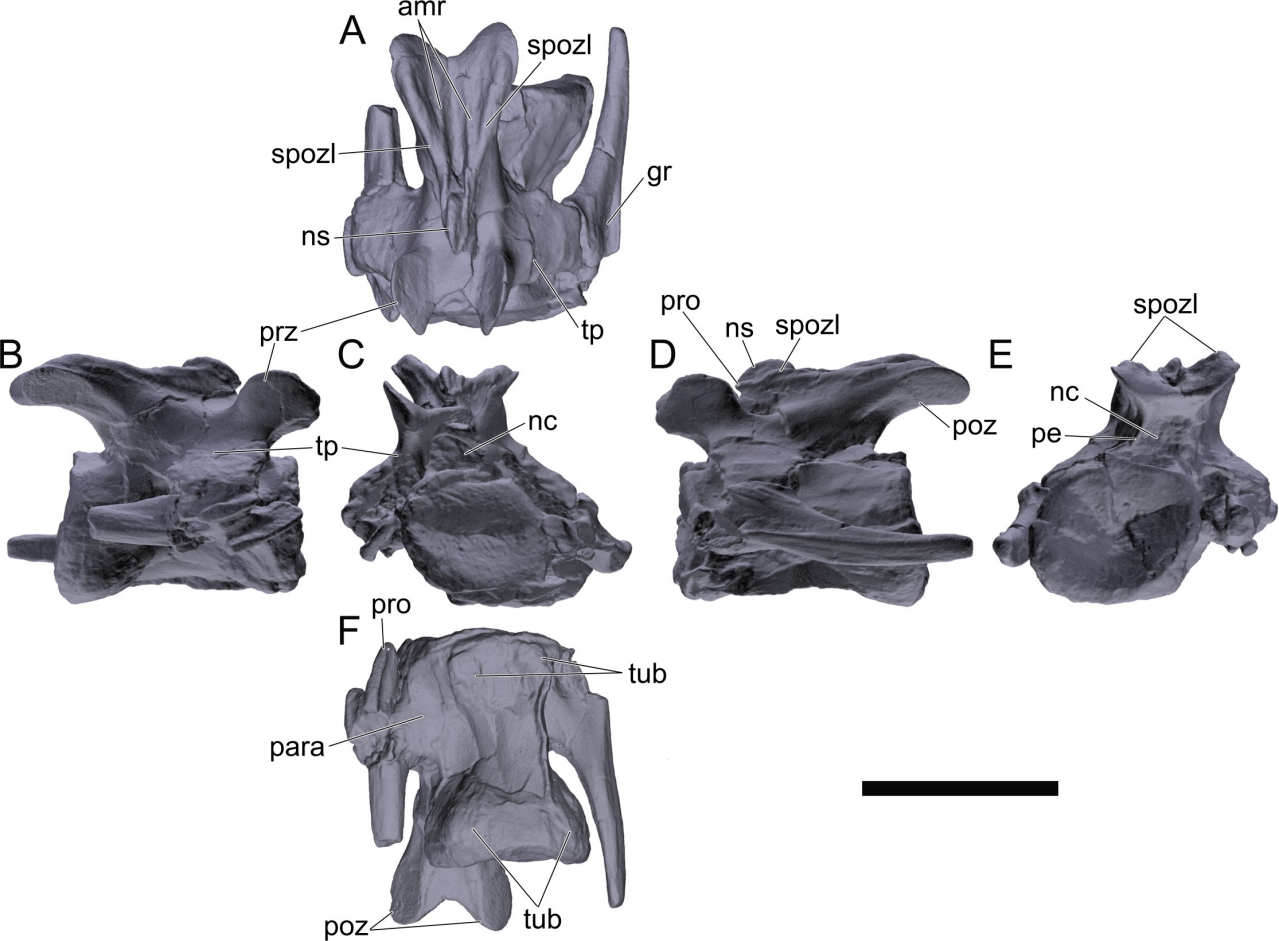
Cervical vertebra nine of *Miragaia longicollum* MG 4863 (screenshots of 3D model). **A**, dorsal, **B**, right lateral, **C**, anterior, **D**, left lateral, **E**, posterior, and **F**, ventral view. **Amr**, assessory medial ridges of spinopostzygapophyseal laminae, **gr**, groove, **nc**, neural canal, **ns**, neural spine, **para**, parapophysis, **pe**, pedicel, **poz**, postzygapophysis, **pro**, process, **prz**, prezygapophysis, **spozl**, spinopostzygapophyseal lamina, **tp**, transverse process, **tub**, tuberosities. Scale bar equal to 10 cm. Published under a CC BY license, with permission from Marco Marzola, original copyright 2017.

9th cervical vertebra and ribs (MG 4863–10; Figs 14 and 15; 3D model provided in S3 Fig): Complete vertebra with fused cervical ribs, deformed and sheared dorso-laterally, missing just the tip of the right cervical rib. This is the most complete and best preserved cervical vertebrae of MG 4863. The centrum body is considerably longer than tall or wide, 1.6 times longer than tall measured at the posterior end. The posterior articulation of the centrum is subcircular in outline (Figs 14E and 15E). The centrum is amphicoelous with well-defined concavities, which gives a funnel mouth appearance, mainly to the posterior articulation (Figs 14E and 15E). In the anterior articulation the cavity of the centrum converges in a horizontal line (Figs 14C and 15C). Both posterior and anterior rims of the centrum are rugose, bulging out in a pair of small hillocks or tuberosities in each centrum rim, positioned latero-ventrally (Figs 14F and 15F). The ventral aspect is damaged, but one can perceive an hourglass outline in ventral view, as well as deep depression in the ventral margin in lateral view (Figs 14D, 14F, 15D and 15F). The parapophyses are placed on the anterior half of the centrum and at mid-height of the centrum, projecting laterally and fused to the ribs (Figs 14C, 14F, 15C and 15F). The transverse processes project lateroventrally from the intersection of the prezygapophysis with the lateral sides of the pedicels, extending proximally over almost the complete anterior half of the centrum (Figs 14A, 14B, 14C, 15A and 15B). The transverse processes surpass the width of the centrum. The right diapophysis is fused to the rib, with a noticeable rugosity (Figs 14B, 15A and 15B). The suture contact between left diapophysis and rib is visible (Figs 14A and 15A). The neurocanal is taller than wide, with the floor horizontal and flattened, while the tall pedicels converge to a gently looped roof dorsally (Figs 14C, 14E, 15C and 15E). Both pedicels are less than 10 millimeters thick, while the neurocanal is about thirty millimeters wide. The pedicels insert in almost all length of the centrum and the neural arch has about equal height to the centrum (Figs 14B, 14D and 15B). The prezygapophyses project anterodorsally and beyond the anterior centrum face (Figs 14B and 15B). The prezygapophyses are convex on the lateral surface and flat on the articular facets, which face dorsomedially (Figs 14A and 15A). In lateral view, the margins of the prezygapophyses are curved, but bear a gentle notch in the anterodorsally margin (Figs 14B, 14D, 15B and 15D). The interprezygapophyseal lamina is wide (but broken with each half overlapping, making it appear shorter). The postzygapophyses are tongue-shaped and project considerably beyond the centrum, with the articular facets surpassing the posterior articulation (Figs 14B, 14D, 14F, 15D and 15F). The postzygapophyses are bridged by an interpostzygapophyseal lamina, which extends beyond the centrum and forms a concave rim in dorsal view (Figs 14A and 15A). The postzygapophyseal facet is longer than wide, oval shaped (Figs 14B, 14F, 15B and 15F). The postzygapophyseal facets are inclined forming a "V" angle of about 80º in posterior view (Figs 14E and 15E). Both medial and lateral edges of postzygapophyses are directed horizontally in lateral view (Figs 14B, 14D and 15D). The spinopostzygapophyseal laminae are thick and well visible, originate posteriorly on thick epipophyses, connect with the sides of the neural spine and carry on anteriorly until its anterior projection, along with two parallel accessory crests medially (Figs 14A, 14D, 14E, 15A, 15D and 15E). The neural spine is not expanded distally, it is low, about 20 mm tall and 30 mm axially long (Figs 14A, 14D, 15A and 15D). The outline of the neural spine in lateral view is gently curved dorsally, with an anterior projection or process at its mid-height and a notch at the base (Figs 14B, 14D, 15B and 15D). It is positioned anteriorly, much closer to the prezygapophyses (nearly overlapping them in lateral view) than to the postzygapophyses. The cervical ribs are positioned lateral to the centrum and run parallel to it, both in lateral and ventral views (Figs 14A, 14F, 15A and 15F). The shaft of the rib is gently curved, bowing slightly dorso-medially (Figs 14D and 15D). The rib extends posteriorly beyond the centrum. Evident longitudinal grooves are present medially and laterally at mid-length, and the posterior tip is blunt and round (Figs 14A and 15A). The articulation facets of the capitulum and the tuberculum are longer than tall and extend for the entire length of the transverse processes. From the rib body there is an anterior process projecting anteriorly about level with the centrum anterior articular facet; smaller processes also project anteriorly from the tuberculum (Figs 14A, 14F, 15A and 15F).

10th cervical vertebra and ribs (MG 4863–104 and MG 4863–105; Fig 10D and 10E): Only the anterior part of the left cervical rib and left diapophysis (MG 4863–104; Fig 10D) and a proximal fragment of the shaft of the right cervical rib (MG 4863–105; Fig 10E) were found, but since these are slightly smaller than those in Cv11 and slightly larger than those in Cv9, they are identifiable as part of the 10th cervical vertebra and ribs. Apart for the differences in size, they are mostly identical to their counterparts in Cv9 and Cv11.

**Fig 16:**
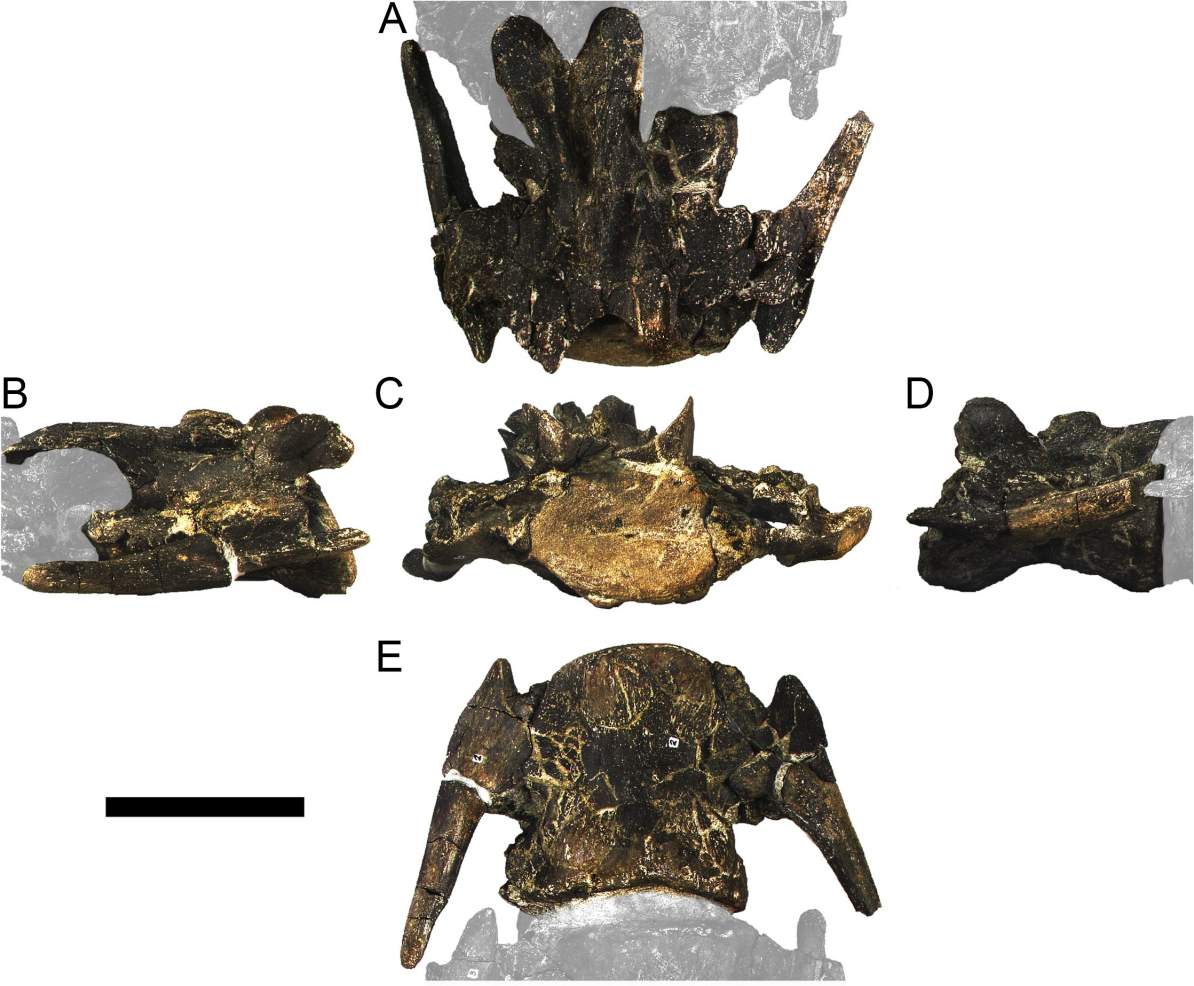
Cervical vertebra 11 of *Miragaia longicollum* MG 4863 (photographs). **A**, right dorsolateral, **B**, right lateral, **C**, anterior, **D**, left lateral, and **E**, ventral view. Scale bar equal to 10 cm.

**Fig 17:**
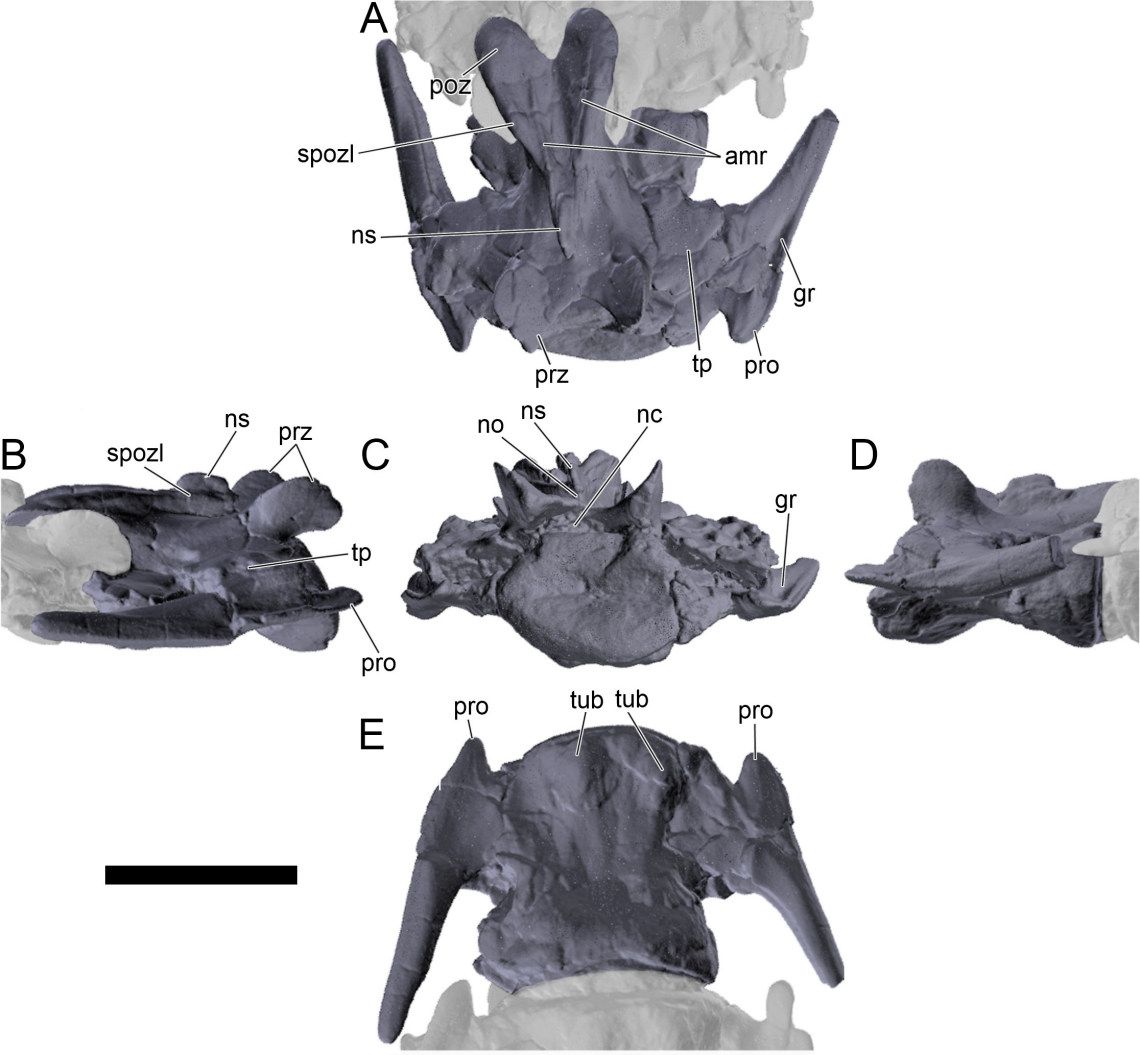
Cervical vertebra 11 of *Miragaia longicollum* MG 4863 (screenshots of 3D model). **A**, right dorsolateral, **B**, right lateral, **C**, anterior, **D**, left lateral, and **E**, ventral view. **Amr**, assessory medial ridges of spinopostzygapophyseal laminae, **gr**, groove, **nc**, neurocanal, **ns**, neural spine, **no**, notch, **poz**, postzygapophysis, **pro**, process, **prz**, prezygapophysis, **spozl**, spinopostzygapophyseal lamina, **tp**, transverse process. Scale bar equal to 10 cm. Published under a CC BY license, with permission from Marco Marzola, original copyright 2017.

11th cervical vertebra and ribs (MG 4863–2; Figs 16 and 17; 3D model provided in S4 Fig): The eleventh cervical vertebra is almost complete, only missing the tip of the left cervical rib. It is semi-articulated posteriorly to Cv12 (*i*.*e*., compressed axially), like Cv12, is severely flattened dorsoventrally, with a collapsed and closed neural canal (Figs 16C and 17C). The centrum seems much wider than long, with an elliptical outline in anterior view and concave in the anterior facet (Figs 16C and 17C). Despite the deformation, an hourglass outline of the centrum is perceptible, with depression ventrally and laterally. The two pairs of tuberosities in the ventral face are visible, and the anterior pair is particularly distinct (Figs 16E and 17E). A closed longitudinal canal is formed by the fusion of the diapophyses and parapophyses to the ribs (Figs 16C and 17C). The transverse processes are rectangular in dorsal view and long anteroposteriorly, extending from the base of the prezygapophyses to beyond the posterior margin of the neural spine (Figs 16A, 16B, 17A and 17B). The prezygapophyses extend half-length beyond the anterior centrum facet. In lateral view, the prezygapophyses are round dorsally in outline with a notch at the anterodorsal margin and an anteriormost projection ventrally positioned (Figs 16B and 17B). The postzygapophyses fully surpass the posterior margin of the centrum and the interpostzygapophyseal lamina fits in the notch ventral to the neural spine of Cv12 (Figs 16B and 17A). The spinopostzygapophyseal laminae and the pair of accessory medial ridges clearly extend from the postzygapophyses and pass on the sides of the neural spine, culminating in the anterior projection of the neural spine (Figs 16A, 16B, 17A and 17B). The neural spine is low and thin, round in outline dorsally and has a round notch under its anterior projection (Figs 16A–16C and 17A–17C). The neural spine is positioned double the distance from the posterior margin of the postzygapophyses than it is from the anterior margin of the prezygapophyses (Figs 16A and 17A). The cervical ribs have well developed anterior processes and deep grooves laterally (Figs 16A, 16B and 17). The ribs project posteriorly and slightly laterally and are subtriangular in cross section.

**Fig 18:**
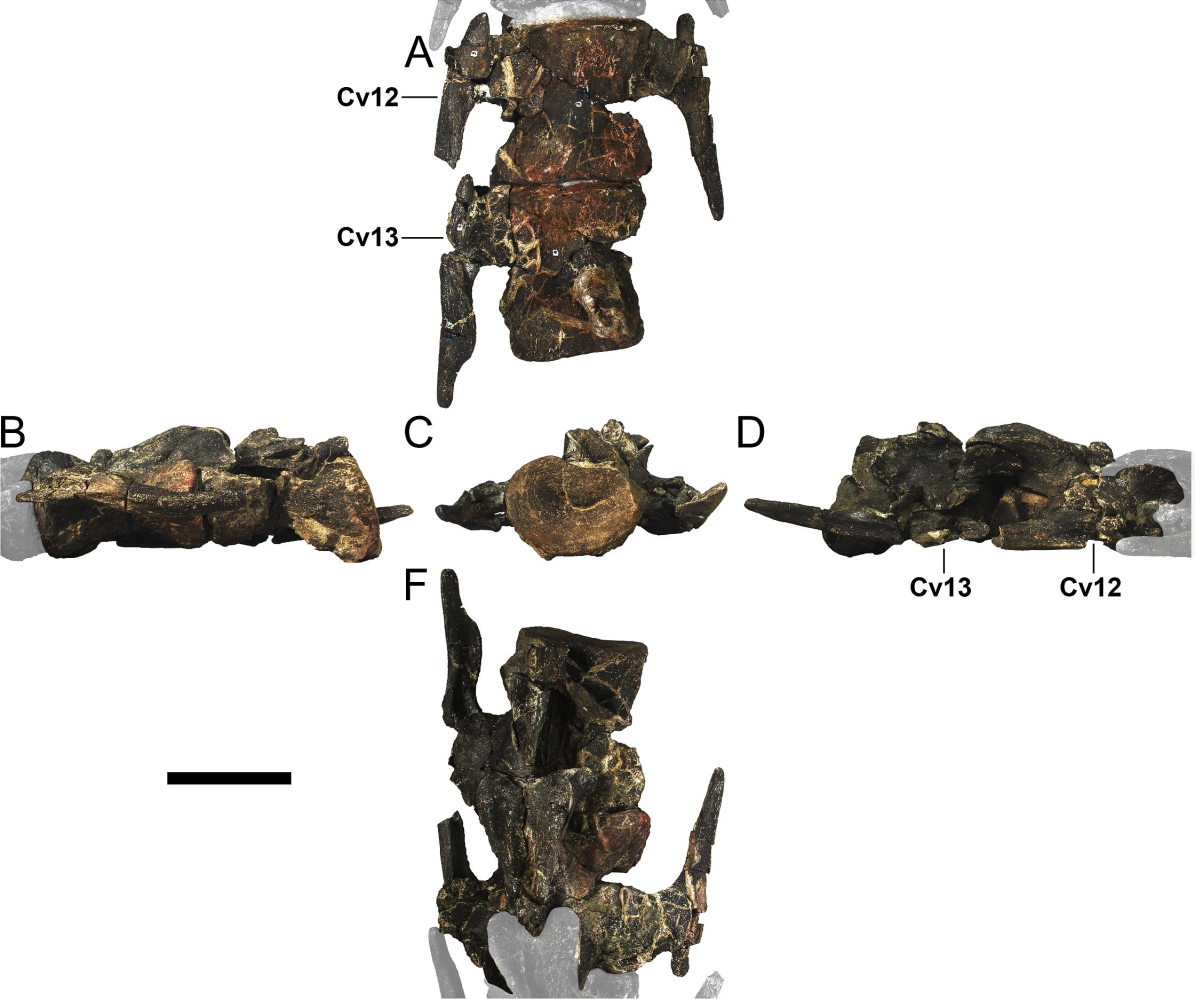
Cervical vertebrae 12 and 13 of *Miragaia longicollum* MG 4863 (photographs). **A**, dorsal, **B**, left lateral, **C**, posterior, **D**, right lateral, and **E**, ventral view. **Cv12**, cervical vertebra 12, **Cv13**, cervical vertebra 13. Scale bar equal to 10 cm.

**Fig 19:**
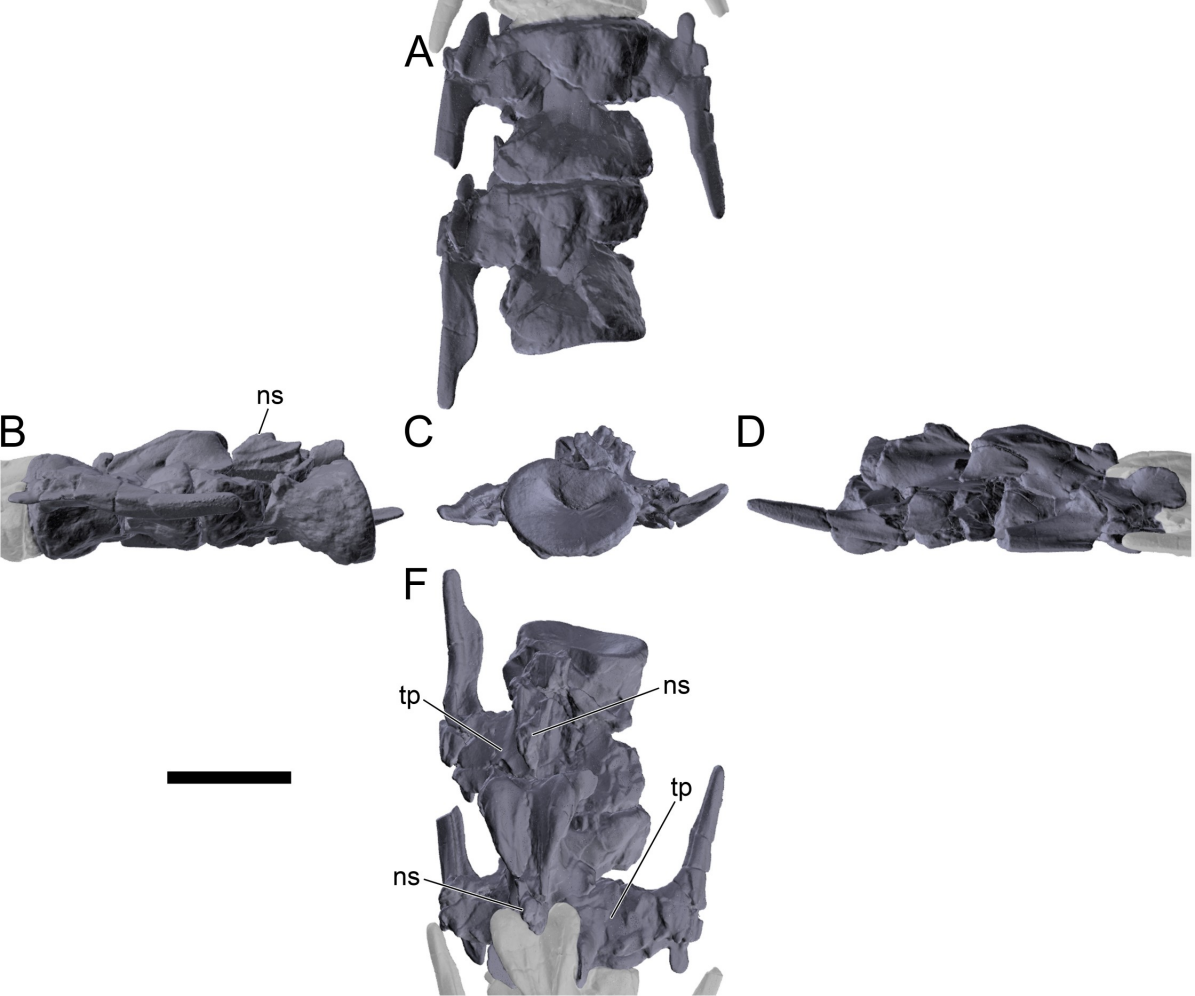
Cervical vertebrae 12 and 13 of *Miragaia longicollum* MG 4863 (screenshots of 3D model). **A**, dorsal, **B**, left lateral, **C**, posterior, **D**, right lateral, and **E**, ventral view. **Ns**, neural spine, **tp**, transverse process. Scale bar equal to 10 cm. Published under a CC BY license, with permission from Marco Marzola, original copyright 2017.

12th cervical vertebra and ribs (MG 4863–3; Figs 18 and 19; 3D model provided in S4 Fig): This vertebra is complete. It is preserved in near articulation as in life between Cv11 and Cv13, without intervertebral spacing probably due to anteroposterior compressive forces. Like the previous vertebra, Cv12 is strongly flattened dorsoventrally.

For what can be observed, Cv12 is anatomically almost identical to Cv11, except for: deeper lateral and ventral depressions enhancing the hourglass outline of the centrum in these views (Figs 18B, 18E, 19B and 19E), the transverse processes are slightly longer (Figs 18A and 19A), and the neural spine is thicker transversely (Figs 18A and 19A).

13th cervical vertebra and ribs (MG 4863–4; Figs 18 and 19; 3D model provided in S4 Fig): The 13th cervical vertebra is missing the postzygapophyses and most of the left side of the neural arch. It is semi-articulated anteriorly to Cv12; the anterior centrum facet of Cv14 was attached with the left posteroventral side of the centrum of Cv13. Like Cv11 and Cv12, it was flattened and deformed dorsoventrally, and in the observable details it is similar to these, except that the transverse process is relatively longer (Figs 18A and 19A) and the neural spine is thicker (Figs 18A, 18B, 19A and 19B). The major difference from Cv11 and Cv12 is the presence of a round shelf extending ventromedially from the ventromedial margin of the shaft of the cervical rib (it is projecting medially, but the rib has rotated, so it would project ventromedially; Figs 18A, 18C, 18E, 19A and 19C). The posterior facet of the centrum is concave converging in an almost horizontal concavity (Figs 18C and 19C). A right postzygapophysis (MG 4863–106; Fig 10F) probably belongs to this vertebra, but it is not possible to attach it to the existing deformed neural arch.

**Fig 20:**
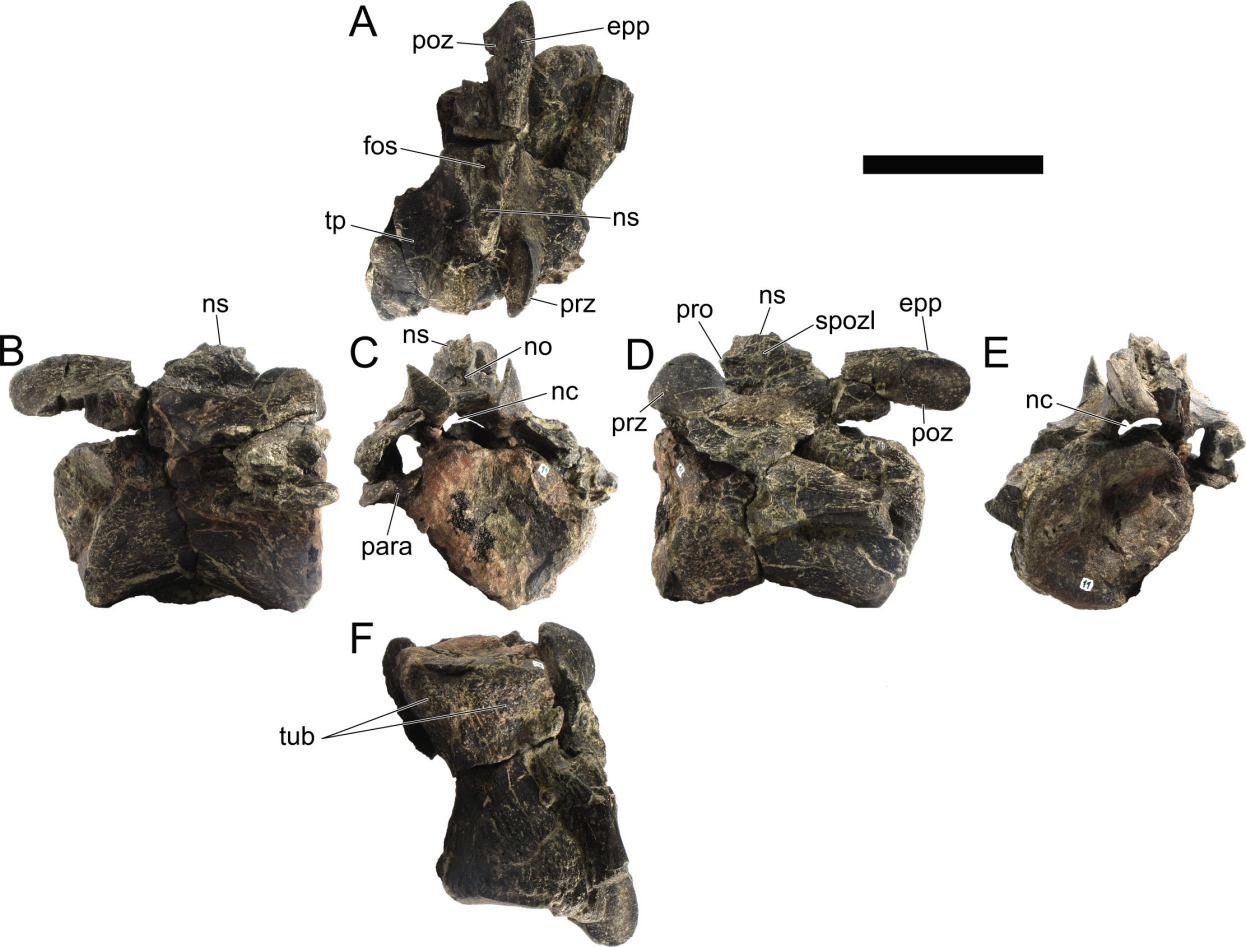
Cervical vertebra 14 of *Miragaia longicollum* MG 4863. **A**, dorsal, **B**, right lateral, **C**, anterior, **D**, left lateral, **E**, posterior, and **F**, ventral view. **Epp**, epipophysis, **fos**, fossa, **nc**, neural canal, **ns**, neural spine, **para**, parapophysis, **poz**, postzygapophysis, **pro**, process, **prz**, prezygapophysis, **spozl**, spinopostzygapophyseal lamina, **tp**, transverse process, **tub**, tuberosities. Scale bar equal to 10 cm.

14th cervical vertebra and ribs (MG 4863–11; Fig 20): Mostly complete cervical vertebra, missing parts of the ribs, right postzygapophysis, neural spine and other details. Dorso-laterally deformed and twisted transversely, with neural arch sheared to the right and centrum strongly deformed and crushed. It was moved to the left from its articulation with Cv13, connecting posterolaterally with the latter. The posterior centrum articulation is subcircular, strongly depressed and shows four fractures in a “X” shaped arrangement, remnants of strong compression forces (Fig 20E). The anterior articulation is partially obscured by bone remnants from Cv13, but it is possible to observe a slightly concave surface, so the centrum is amphicoelous (Fig 20C). The anterior articulation is heart-shaped as a result of the deformation. The centrum is about 1.5 times longer than wide or tall. The centrum wall shows rugosities on both posterior and anterior rims of the centrum, extending almost to mid-point axially (Fig 20F). The tuberosities situated ventrally on the centrum rims do not protrude as much as in other cervical vertebrae, particularly those of the posterior rim. An hourglass outline of the centrum is perceivable in lateral and ventral view, despite the strong deformation (Fig 20B, 20D and 20F). The parapophyses are positioned more dorsally than in more anterior cervical vertebrae, closer to the dorsal top of the centrum than to the ventral surface of this (Fig 20C). The bases of the parapophyses extend to all the anterior half of the centrum length and project laterally. The neurocanal appears to be wider than tall, but this was probably exaggerated by the deformation (Fig 20C and 20E). The neurocanal is about forty millimeters wide and the pedicels are relatively thick. The neurocanal floor is flat and horizontal while the roof is curved. The neural arch appears to be about half the height of the centrum. The pedicels insert in almost all length of the centrum. The postzygapophyseal facet is oval to subround. The postzygapophyses project beyond the centrum posterior articulation. The postzygapophyseal facet is close to vertical. An inter-postzygapophyseal lamina is present and extends beyond the centrum (Fig 20D). The left postzygapophysis is directed horizontally (Fig 20D). A thick epipophysis is present, arising dorsally about 20mm away from the posterior margin of the postzygapophysis (Fig 20A and 20D). This is thick posteriorly, about 10mm wide and 7mm tall, becoming a thinner ridge progressing anteriorly (*i*.*e*., the spinopostzygapophyseal lamina), until merging with the sides of the neural spine and culminating in an anterior projection at the base of it (Fig 20A, 20C and 20D). A deep fossa posteriorly to the neural spine is observable, a result of postzygapophyses much more vertical than in more anterior cervical vertebrae (Fig 20A). Although the neural spine is mostly broken off, it is observable in the sectioned base that it was much wider than more anterior cervical vertebrae and not narrowing dorsally like these (Fig 20A–20C and 20D), indicating a missing neural spine probably some centimeters tall (more like those found in ML 433 and NHMUK OR46013). The neural spine section is 18mm wide, 30mm long and extends dorsally until 10mm from the spinopostzygapophyseal lamina lateral to it (Fig 20A–20C and 20D). An anterior projection at the base of the neural spine–the anteriormost point of it–is present, as well as a notch ventrally to this, as observed in ML 433 (Fig 20C and 20D). The prezygapophyses are convex laterally and, in lateral view, rounded in outline, apart for a slight notch on the anterodorsal margin (Fig 20A, 20C and 20D). The prezygapophyses extend anteriorly beyond the centrum, while the interprezygapophyseal lamina extends about as far as the anterior articular centrum facet. The articulation facets of the prezygapophyses are flat. The transverse processes project lateroventrally between the prezygapophysis and the pedicels and surpass the centrum width (Fig 20A and 20C).

**Fig 21:**
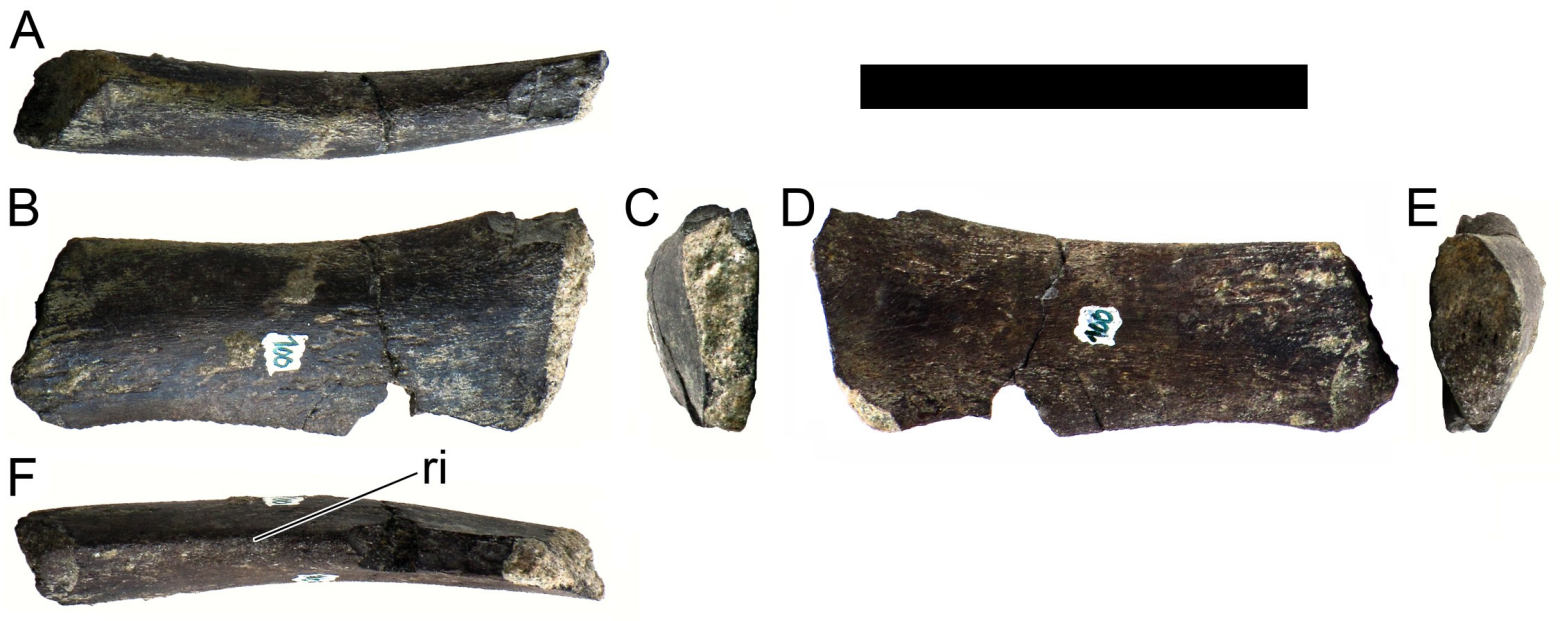
Cervical rib 17 of *Miragaia longicollum* MG 4863. **A**, dorsal, **B**, anterolateral, **C**, anteromedial, **D**, posteromedial, **E**, posterolateral, and **F**, ventral view. **Ri**, ridge. Scale bar equal to 5 cm.

17th cervical rib (MG 4863–100; Fig 21): Right partial cervical rib from the 17th cervical vertebra, section of the rib rod between the capitulum and distal tip. Transversely thin, it is three times taller than wide proximally (Fig 21C) and two times taller than wide distally (Fig 21E). It is slightly thicker near the dorsal side, giving it an oval cross section outline (apex pointing ventrally), so the dorsal margin is blunt and the ventral margin is a sharp longitudinal ridge (Fig 21F). It is thinnest dorsoventrally at mid-length, expanding slightly towards the distal and proximal ends (Fig 21B and 21D). It is gently curved medially (Fig 21A and 21F). No additional longitudinal ridges are present on the sides. Through first-hand observations and comparisons, its morphology is closely analogous only with the posteriormost cervical ribs of ML 433, especially the 17th cervical ribs (which, in *M*. *longicollum*, are peculiar for sharing morphological features with the dorsal ribs, effectively being a transitional form). It is distinguishable from more anterior cervical ribs because, while these have subtriangular or round cross sections, it is transversely flattened in all length. It is distinguishable from anterior dorsal vertebra for its size, with close distal and proximal expansions, and for the lack of longitudinal lateral or medial ridges.

#### Dorsal vertebrae

Five semi-complete dorsal vertebra were found (three semi-articulated and two isolated), as well as other fragments of dorsal transverse processes. All dorsal vertebrae are missing most of the top of the neural arch, except for MG 4863–17, which appears to be almost complete but is still mostly encased in matrix. Only two vertebrae (MG 4863–14 and MG 4863–13) could be fully prepared at the time of writing. All the other dorsal vertebral parts are almost completely encased in matrix, limiting the descriptions and comparisons of the dorsal vertebral series at this point. A more detailed description of the dorsal vertebrae, after their preparation can be completed, will be included in a future publication regarding the osteological reconstruction of *Miragaia* with the available fossil record.

**Fig 22:**
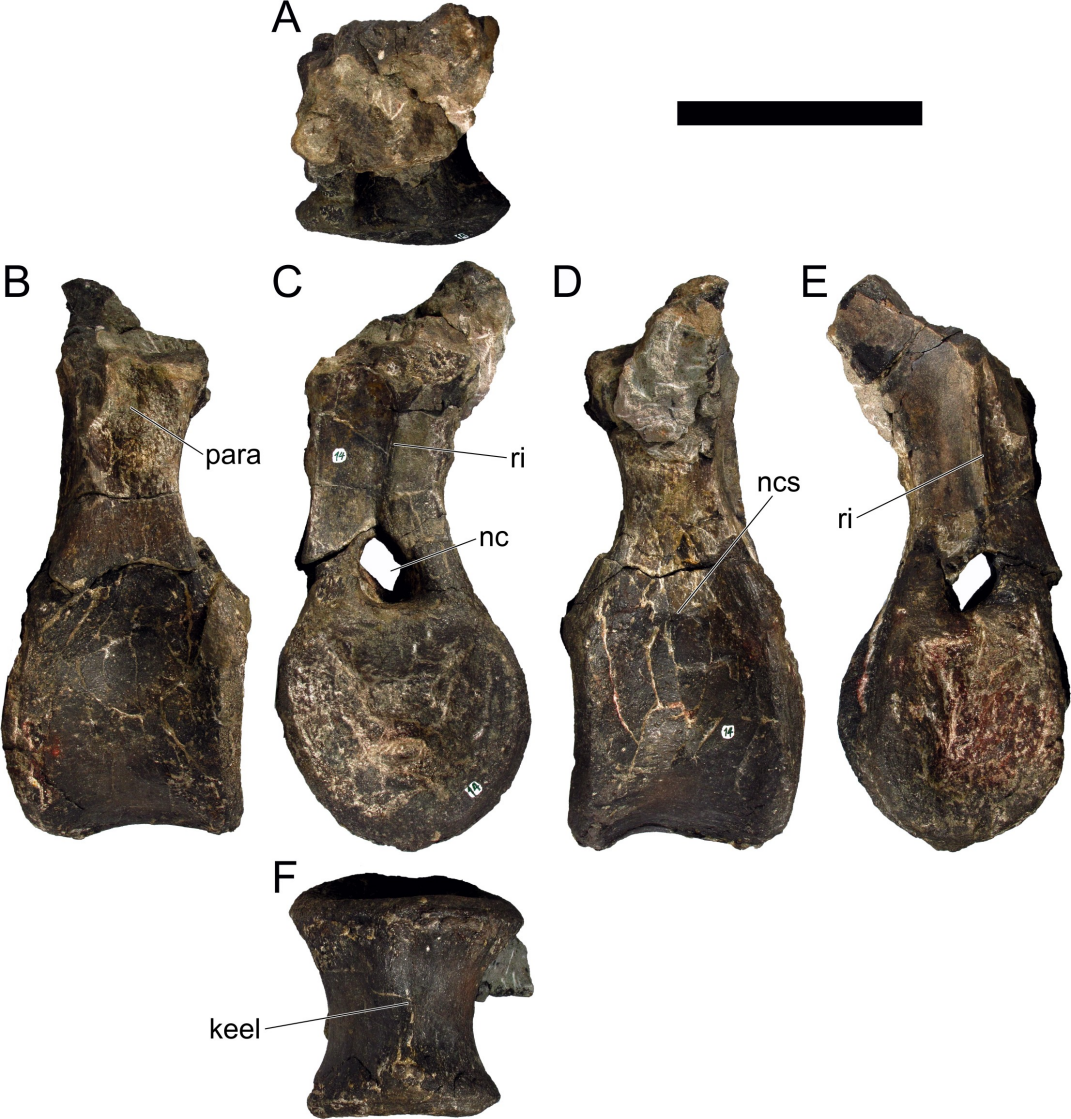
Dorsal vertebra two of *Miragaia longicollum* MG 4863. **A**, dorsal, **B**, right lateral **C**, anterior, **D**, left lateral, **E**, posterior, and **F**, ventral view. **Keel**, keel, **nc**, neurocanal, **ncs**, neurocentral suture, **para**, parapophysis, **ri**, ridge. Scale bar equal to 10 cm.

**Fig 23:**
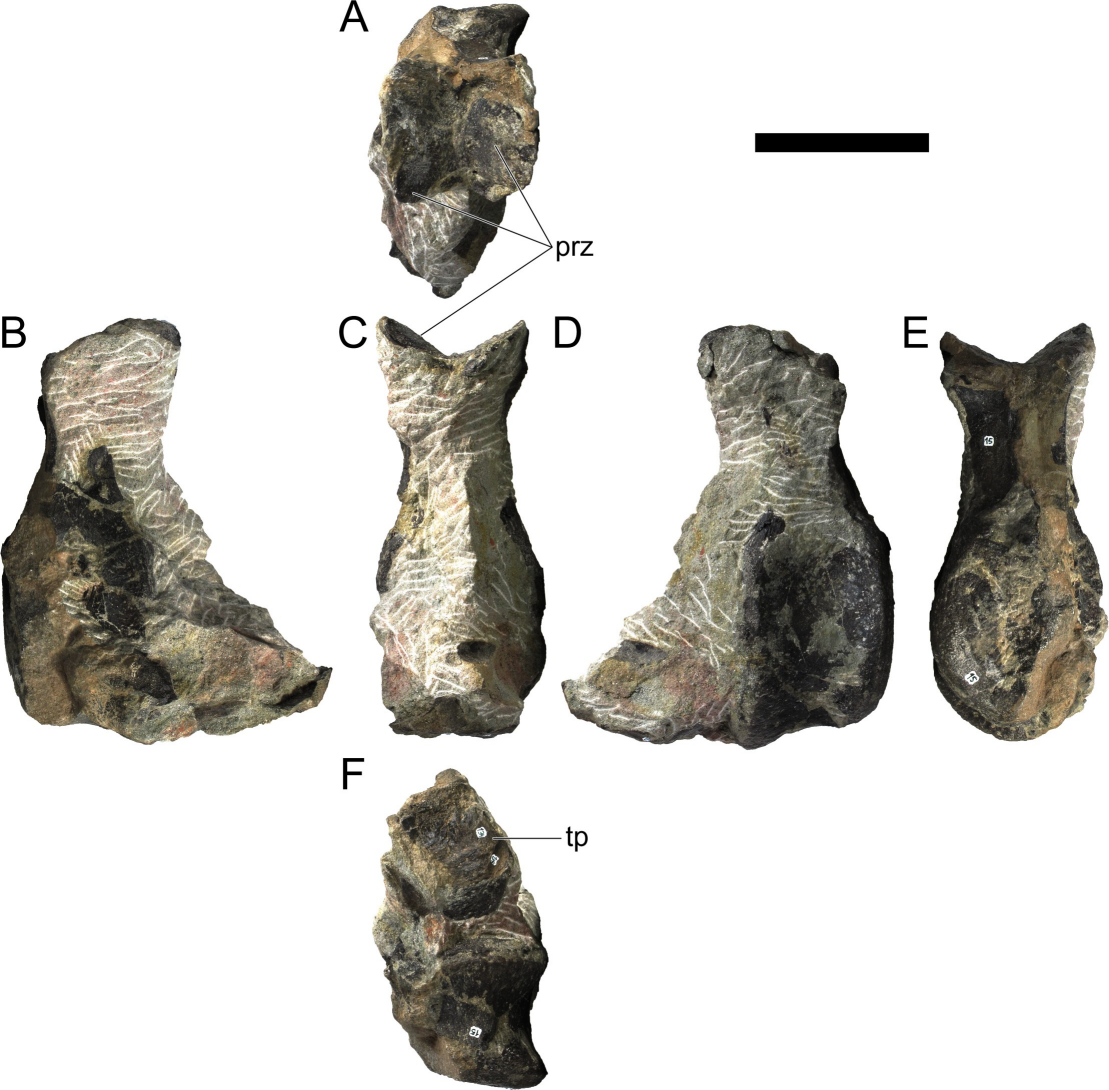
Dorsal vertebra three and unidentified dorsal transverse process of *Miragaia longicollum* MG 4863. **A**, dorsal, **B**, right lateral, **C**, anterior, **D**, left lateral, **E**, posterior, and **F**, ventral view. **Tp**, transverse process, **prz**, prezygapophysis. Scale bar equal to 10 cm.

**Fig 24:**
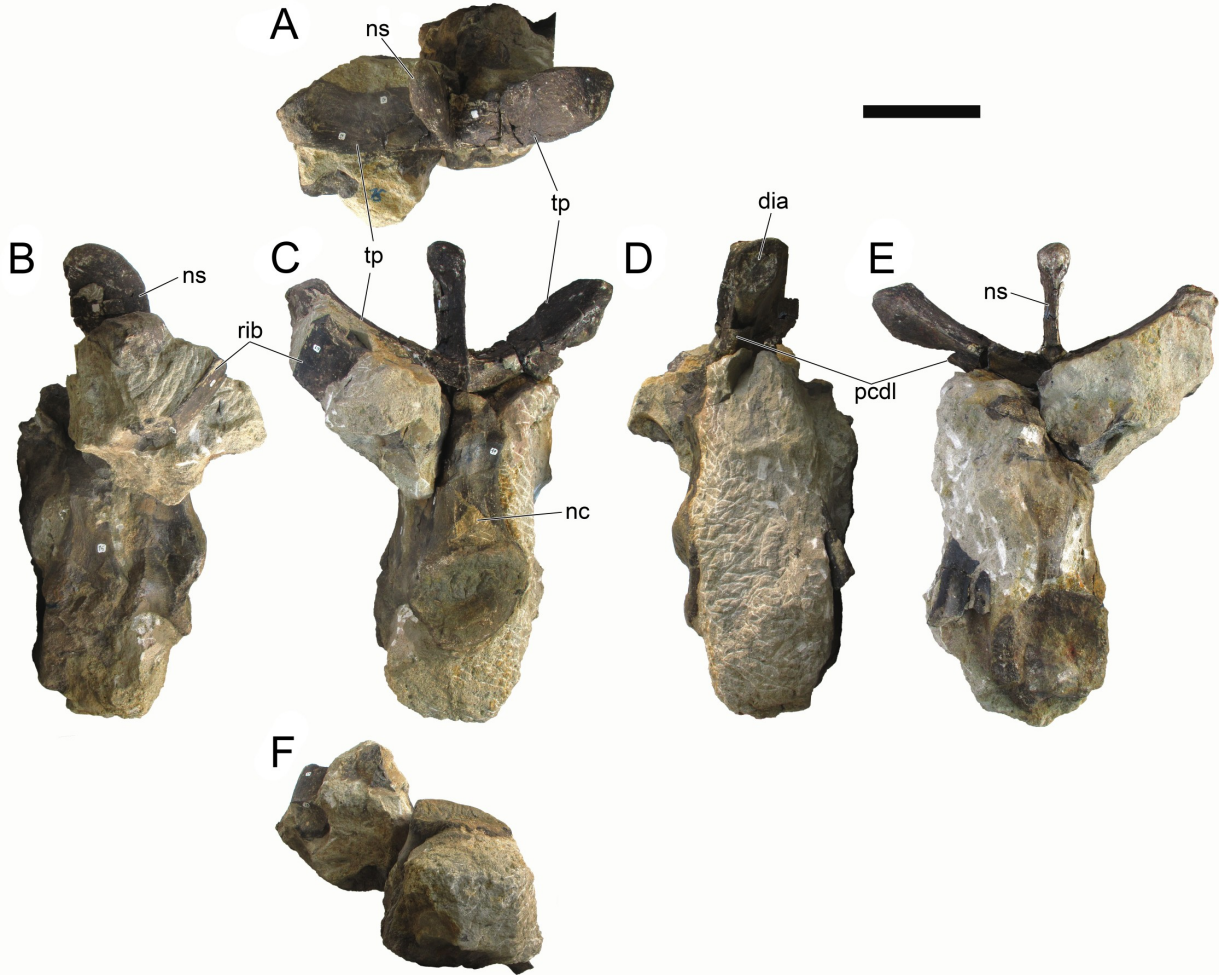
Dorsal vertebrae five, six and dorsal rib of *Miragaia longicollum* MG 4863. **A**, dorsal, **B**, right lateral, **C**, anterior, **D**, left lateral, **E**, posterior, and **F**, ventral view. **Dia**, diapophysis, **nc**, neurocanal, **ns**, neural spine, **pcdl**, posterior centrodiapophyseal lamina, **rib**, rib, **tp**, transverse process. Scale bar equal to 10 cm.

**Fig 25:**
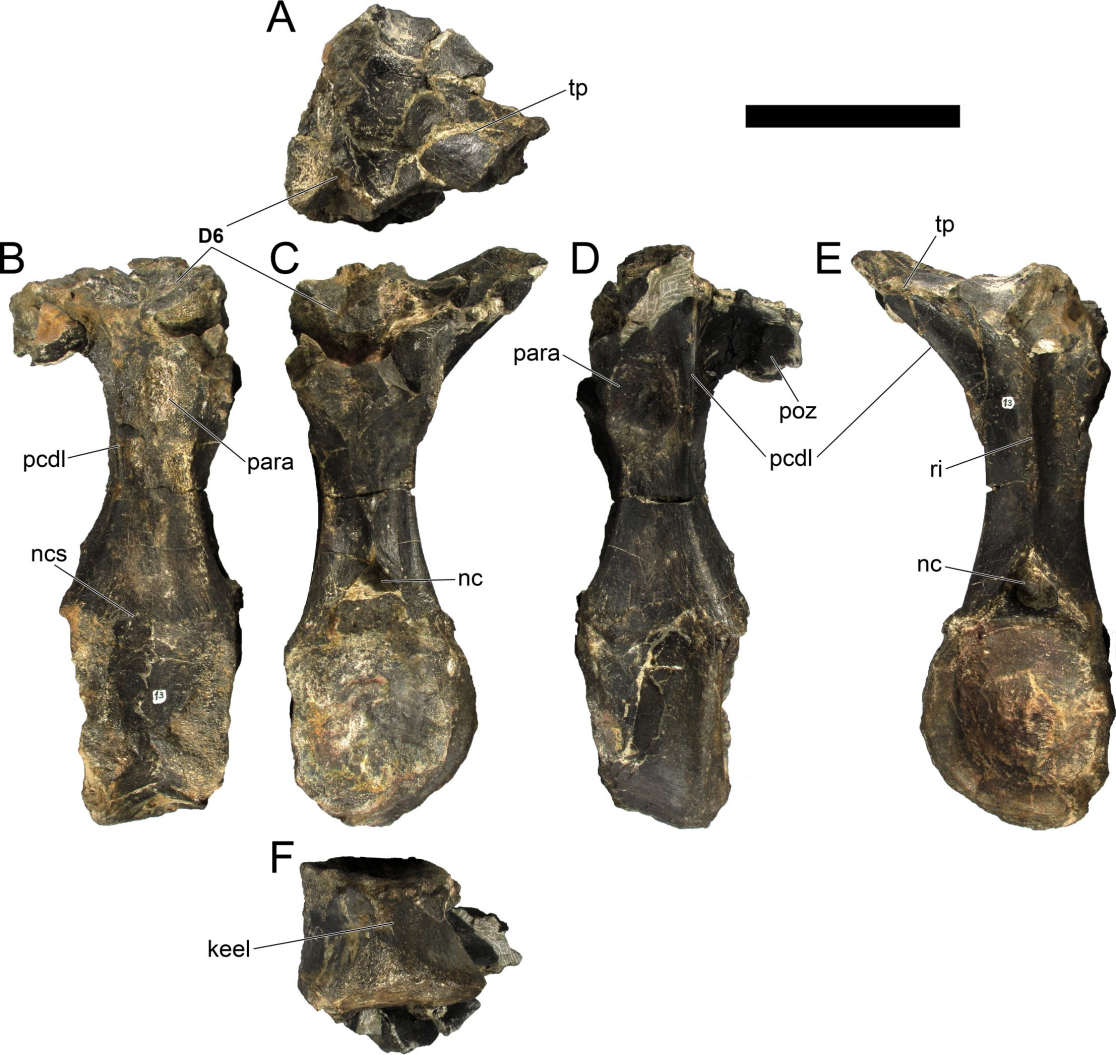
Dorsal vertebra seven and part of six of *Miragaia longicollum* MG 4863. **A**, dorsal, **B**, right lateral, **C**, anterior, **D**, left lateral, **E**, posterior, and **F**, ventral view. **D6**, postzygapophyses of dorsal vertebra six, **Keel**, keel, **nc**, neurocanal, **ncs**, neurocentral suture, **para**, parapophysis, **pcdl**, posterior centrodiapophyseal lamina, **poz**, postzygapophysis. Scale bar equal to 10 cm.

The number of dorsal vertebrae in *Miragaia longicollum* has been anticipated to be most likely between 8 and 10 [22], but no complete dorsal series has been found yet to confirm this. While only the two most anterior dorsal vertebrae from ML 433 were found, the *D*. *armatus* from Moçafaneira [41] reportedly has a complete dorsal vertebra, three dorsal centra and nine dorsal neural arches, but it is unclear if these represent a complete dorsal series and the total of dorsal vertebrae present. The collection of dorsal ribs of MG 4863 also does not conclusively indicate a total number of dorsal vertebrae present. With the currently available information, MG 4863–14 (Fig 22) and MG 4863–15 (Fig 23) are here identified as anterior dorsal vertebra (respectively, 2nd dorsal vertebra and 3rd dorsal vertebra) for their shorter pedicels and large neural canals, characteristic of anterior dorsal vertebrae [1]. Three dorsal vertebrae are articulated in near life position–namely, from the most anterior: MG 4863–17, -16 (Fig 24) and -13 (Fig 25)–and, due to their taller pedicels, smaller neural canals and more dorsally positioned parapophyses, were identified respectively as the 5th dorsal vertebra, 6th dorsal vertebra, and 7th dorsal vertebra. The order of the dorsal vertebrae is most probably bound to be reinterpreted after a more comprehensive preparation. D7 is the most complete of the two fully prepared dorsal vertebrae (the other being D2), but it is missing the neural spine, right transverse process, distal half of left transverse process, and the post- and prezygapophyses (Fig 25). The partial preparation of D5 revealed most of the transverse processes and the only complete dorsal neural spine, but the prezygapophyses are missing (Fig 24). D3 is the only dorsal vertebra that includes well preserved prezygapophyses, but these are partially encased in matrix and almost nothing else remains of the top of the neural arch (Fig 23). The postzygapophyses of D6 broke off from it and are articulated with D7 (Fig 25A–25C). Three partial dorsal transverse processes were found (MG 4863–63, -112 and -134) associated with the dorsal vertebrae, which appear to not fit with these, but it is unclear to what vertebrae they pertained. Since a full description of all properly identified dorsal vertebrae is not possible, general trends are here described.

The centra represent slightly less than one third of the total height of the vertebrae. The centra are amphicoelous and broader posteriorly than anteriorly. In axial view they are sub-round but slightly flattened dorsally which gives them a “heraldic shield” outline (Figs 22C, 22E and 24C). The neural canal is flattened ventrally but slightly acute dorsally, which gives it a pyriform to droplet-like outline (Figs 22C, 25C and 25E). The ventral surface of the centrum bears a subtle midline keel (Figs 22F and 25F). The centra are wider transversely than long and hollowed laterally in ventral view (Figs 22F and 23F). The fused neurocentral suture is subtle but visible (Figs 22D and 25B). The concave articular facet of the centrum is rather rugose and irregular (Figs 22C, 22E, 25C and 25E). The shape of the articular concavity is more funnel-like (towards one mid-point) rather than a continuous parabolic concavity. As in most stegosaurs, the pedicels are a tall and solid column, about slightly more than 1.5 times the height of the centrum. The pedicels in cross section are square to hexagonal, due to the posterior and anterior midline ridges. The anterior ridge projects more than the posterior ridge, and both extend ventrally from the base of the zygapophyses to merge with the top of the neural canal (Figs 22C, 22E and 25E). In the top of the pedicels and ventral to the transverse processes there are conspicuous parapophyses, elliptical in outline and with a markedly concave surface (Figs 25B and 25D). The posterior centrodiapophyseal lamina passes posteriorly to the parapophyses (Figs 25B, 25D and 25E). The prezygapophyses form, together with the interprezygapophyseal laminae, an anterior shelf, which is “V” shaped in anterior view and houses the postzygapophyses of the preceding vertebra (Fig 23A and 23C). The transverse processes are very conspicuous, projecting laterodorsally at an angle of about 30 degrees to the horizontal (Fig 24C and 24E). The lateral end of the diapophyses is sub-triangular in outline, with a flat dorsal surface and a ventral vertex, from which the posterior centrodiapophyseal lamina projects (Fig 24C, 24D and 24E). The articular surface of the diapophyses is conspicuous and broad (Fig 24D). In dorsal view the transverse processes have subparallel anterior and posterior margins and rounded lateral margins (Fig 24A). The neural spine projects dorsally and is slightly inclined posteriorly. The neural spine body is very compressed transversely and long anteroposteriorly, except at the apex, which is expanded transversely, forming a gently round dorsal table (Fig 24A and 24E). This expansion is gradual from the upper third of the neural spine in axial view and its dorsal surface faces anterodorsally, so its posterior end is the dorsalmost point of the dorsal vertebrae (Fig 24B and 24E). Measurements of all dorsal vertebrae are given in Table 2.

#### Caudal vertebrae

Twenty-five semi-complete caudal vertebrae and some vertebral caudal fragments were found. These represent the series from the first to the 37th caudal vertebra, missing eleven vertebrae amid the sequence. Only Cd7 to Cd16 (MG 4863–58 to MG 4863–30) were in a continuous articulated sequence. During preparation, the matrix connecting this sequence was mostly removed, but enough was left so that the articulation in death can be recovered, which evidences a markedly dorsal curvature from Cd9 to Cd14. All other caudal vertebrae were found non-articulated, in isolated blocks or in association with other bones. The relative position was estimated from the volume of the centra, considering that in other stegosaur specimens these become proportionately smaller progressing posteriorly [19], by osteological features reported in other stegosaurian caudal vertebrae (e.g.: [1,27]) and by comparison with the articulated sequence of Cd7 to Cd16. The estimated length of the tail in life position from Cd1 to Cd37 (including missing vertebrae, separated by about 10 mm as evidenced by the articulation of Cd7 to Cd16) is 230 cm. Roughly five to eight posteriormost caudal vertebrae are missing, for a total of approximately 45 caudal vertebrae (estimated considering a total of 35 to 42 in *Huayangosaurus*, 45 in *Hesperosaurus*, and 45 or 46 in *Stegosaurus*; [1,19]). It is possible that the second caudal vertebra is actualy the third, which would move all other posterior caudal vertebrae up one position, but it is here identified as more likely Cd2 (see bellow). The caudal vertebrae can be roughly separated in four morphotypes: the most anterior (from Cd1 to Cd6) have axially narrow centra, thick pedicels, wide caudal ribs, tall neural spines and are the only ones without chevron facets; the antero-medial (from Cd7 to Cd11) have reduced caudal ribs and still have tall neural spines; the medial (from Cd12 to Cd27) have vestigial or absent neural spines, longer centra, only reduced transverse processes remain of the caudal ribs, and the postzygapophyses are still bifurcated in dorsal view; the posterior (from Cd28 onwards) are the most compressed ventrodorsally and the postzygapophyses are not bifurcated in dorsal view. Measurements of all caudal vertebrae are given in Table 2.

All caudal centra are wider than long or tall, as well as taller than long. The centra are much wider transversely than long axially in the most anterior vertebrae (more than twice as wide than long), are longer axially in the medial vertebrae than in the anterior series (relatively and absolutely, approximately 1.5 times wider than long) and become relatively wider in the posterior caudal series (almost twice as wide as long). The centrum outline in axial view is round and slightly dorsoventrally flattened in anterior vertebrae, while in medial and posterior vertebrae it is round to apple-shaped–that is, the neural canal excavates a semi-circle shaped concavity in the dorsal face, the lateral sides are convexly curved and widest in the dorsal half, and the ventral base is flattened or concave between the chevron facets. The caudal centra appear to be amphicoelous (except for Cd1 and the posteriormost centra, which are, respectively, opisthocoelous and acoelous), with deeper depression in medial and posterior centra. In all but the most anterior caudal vertebrae, the centrum articular rims are notably expanded, giving a deeply concave outline to the centrum sides in ventral and lateral view. The posterior centrum rim projects ventrally more than the anterior in anterior caudal vertebrae–the most in Cd1, but levels out progressively with the anterior rim until Cd16. A horizontal lateral ridge is not present in any of the caudal centra as is in other stegosaurs (such as *S*. *stenops*; [19]), so the lateral faces are smoothly curved. The posterior chevron facets are well developed and easily distinguishable, while the anterior facets are much less developed or even unnoticeable (but in Cd1 to Cd5 both are absent). The chevron facets are always distinctly separated in transverse pairs, suggesting that all chevrons had an open haemal canal. The area between and including the chevron facets is flatter or more concave than the rest of the centrum wall. In most of the articular facets there are concentric ridges and grooves, variable in thickness, amount and occurring variably along the caudal series (some of the vertebrae lacking these may be due to poor preservation). These ridges may converge in a round or trapezoid-shaped swelling dorsally in the articular facet (just ventral to the neural canal, effectively elongating its floor) that in cases draws to a point beyond the margin of the centrum in dorsal view. Another process in the center of the centrum surfaces is variably present or not along the caudal series, with variable size and shape (often pointed). These central processes are more common in the more deeply concave articular surfaces. Most centrum facets evidence a texture of sub-millimetric and tightly packed pustules covering them (the ones that lack these are apparently due to poor preservation).

The neurocanal varies slightly in shape, but is generally teardrop-shaped, with the round base excavating a crescentic groove in axial view along the dorsal side of the medial and posterior caudal centra. The neurocanal is relatively larger and rounder in the most anterior caudal vertebrae. The pedicels are thin in medial and posterior vertebrae, but in the most anterior vertebrae are much thicker close to the anterior margin and have a subtriangular cross-section, where these merge with the deep bases of the transverse processes. In the anterior vertebrae the pedicels extend from the anterior to the posterior faces of the centrum, but extend less posteriorly in medial and posterior caudal vertebrae, (about two thirds the length of the centrum). The neurocanal and neural arch are much reduced in all but the most anterior vertebrae, about one third the width of the centrum (measured from the lateral margins of the pedicels) and about one third the height of the centrum (measured from the dorsal top of the centrum to the dorsal margin of the neural arch, dismissing the neural spine when present). There is no noticeable neurocanal suture in the caudal vertebrae.

The caudal ribs decrease in size progressing posteriorly: from elongate processes each about as wide as the centrum from Cd1 to Cd5, thin blades expanding just beyond the centrum lateral margins from Cd8 to Cd13, depressed and irregular stubs or rugosities from Cd14 to Cd36, and a vestigial ridge in Cd37. The caudal ribs are much taller dorsoventrally than long axially in the anterior vertebra, becoming narrower dorsoventrally and thicker anteroposteriorly passing posteriorly–by Cd5 and Cd7 they are about as tall as they are long, and along the medial caudals they are thin dorsoventrally and longer anteroposteriorly. In the anterior and medial caudal vertebrae they are laterally directed (with only a slight curvature ventrally in the distal tips of the anteriormost vertebrae). A proximodorsal projection is present in the ribs from Cd2 to Cd5 (possibly was present until Cd7, but is absent by Cd8). This process points dorsally in Cd2, but points more anteriorly in Cd3 and Cd5. Another, much smaller distoventral process is present in the ribs of Cd2 and Cd5, that while it points ventrally in Cd2, in Cd5 it points mainly posteriorly, suggesting that there is some rotational component in the observed anteroposterior thickening and dorsoventral narrowing of the caudal ribs passing posteriorly. The caudal ribs are placed on the dorsal half of the caudal centra, move more dorsally passing posteriorly on the caudal series and are either equidistant from the anterior and posterior margins or slightly more posteriorly positioned. In the medial and some of the anterior caudal vertebrae the contact of the transverse process with the centrum is marked dorsally by a low anteroposteriorly oriented rugose ridge (its rugosities are clearly oriented anteroposteriorly, distinct from the rugosities on the caudal ribs which are oriented transversely). This ridge in medial caudal vertebrae culminates in an anterior projection, overhanging the sides of the centrum, that becomes more developed and sharper passing posteriorly on the caudal series. Although the caudal ribs and transverse processes are fused in a single osteological unit, the contact suture between these can be perceived in some vertebrae (e.g., in Cd8, Cd10 and Cd11) while in others the ribs have broken off through this connection (e.g., in Cd9 and Cd12). The anatomy suggests that the ribs become vestigial between Cd12 and Cd18, so only the transverse processes are retained from there onto the most posterior vertebrae.

The neural spines from Cd1 to Cd11 are tall (somewhat taller than each centrum), straight in lateral view with an elliptical cross-section (except for Cd1, that has a gently posteriorly curved and transversely compressed spine) with moderately expanded and non-bifurcated apices. The neural spine is virtually vertical in Cd1, becoming more posteriorly inclined progressing posteriorly, pointing posterodorsally at Cd11. The neural spines decrease sharply in size passing posteriorly between Cd10 and Cd12 (in fact most likely between Cd11 and Cd12), from a spine taller than the centrum in Cd10 to a low transversely thin ridge in Cd12 (a decrease to about one fifth the height and width). The neural spine is absent by Cd18 on to the end of the caudal sequence. There are three parallel proximodistally oriented ridges in the anterior face of the anterior caudal neural spines–from mid length of the spine, the two lateral ridges culminate on the base of the prezygapophyses (the spinoprezygapophyseal laminae), while the central ridge (usually the most developed of the three) flattens just dorsally to the roof of the neural canal.

The prezygapophyses project anteriorly and extend beyond the anterior facet of the centrum, are lobate in shape with round facets in the anterior caudal vertebrae and finger-like with ellipse-shaped facets in medial and posterior caudal vertebrae, which face dorsomedially. The prezygapophyses are held apart from each other, with no lamina medially (but in the most anterior caudal vertebrae a low round interprezygapophyseal shelf roofs the neural canal). In medial and posterior caudal vertebrae, a longitudinal thick ridge extends on their lateral sides, giving the prezygapophyses a subtriangular cross-section. Pre- and postzygapophyses are positioned almost at the same horizontal level, in some instances the postzygapophyses are slightly more dorsally positioned. The postzygapophyses are small, borne on the base of the neural spine, and do not extend beyond the posterior facet of the centrum (barely expanding posteriorly beyond the neural spine posterior margin). The articular facets of the postzygapophyses are round in outline and face posterolateroventrally. The postzygapophysis are separated by a shallow posterior concavity in dorsal view until the most posterior vertebrae where they are fused with no evident bifurcation.

**Fig 26:**
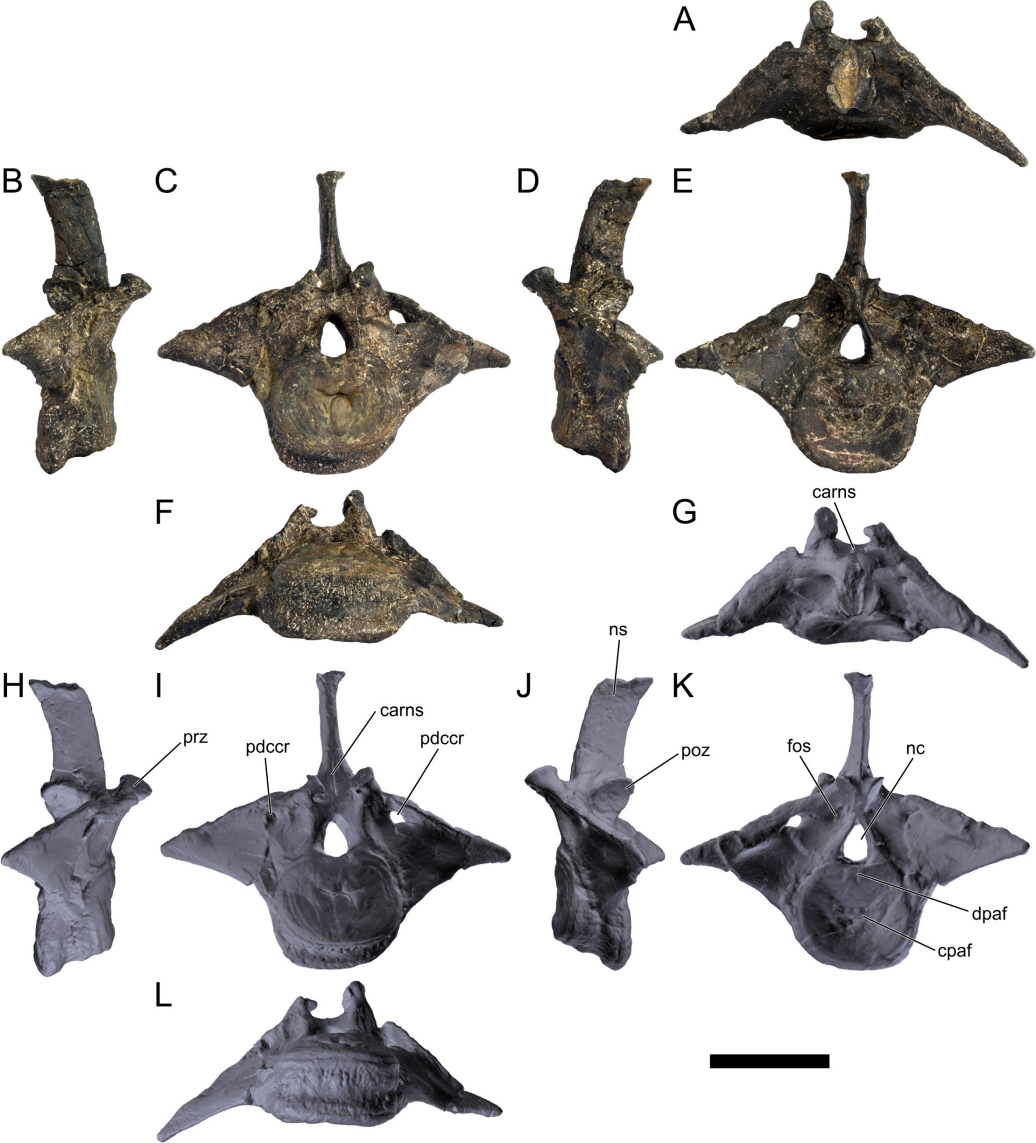
Caudal vertebra one of *Miragaia longicollum* MG 4863. **A–F,** photographs, **G–L,** screenshots of 3D model in **A, G**, dorsal, **B, H**, right lateral, **C, I**, anterior, **D, J**, left lateral, **E, K**, posterior, and **F, L**, ventral view. **Carns**, central anterior ridge of neural spine, **cpaf**, central process of articular facet, **dpaf**, dorsal process of articular facet, **fos**, fossa, **nc**, neural canal, **ns**, neural spine, **pdccr**, proximodorsal canal of caudal rib. Scale bar equal to 10 cm. **G–L**, published under a CC BY license, with permission from Marco Marzola, original copyright 2017.

1st caudal vertebra (MG 4863–34; Fig 26; 3D model provided in S5 Fig): The vertebra is essentially complete, except for the very apex of the neural spine. The right caudal rib was compressed transversely and the right prezygapophysis was deformed against a dorsal transverse process.

The first caudal vertebra bears the most distinct features not shared by others in the caudal series. The centrum is opisthocoelous. The posterior face is concave with two processes in the center of the concavity similar to those found in other articular facets of the series (Fig 26E and 26K). A projection ventral to the neural canal is also present in the posterior facet (Fig 26E and 26K). The anterior facet is mostly flat (but slightly convex), with a “𝜋” shaped groove in its center (Fig 26C and 26I). The centrum outline is sub-round in anterior view, slightly dorsoventrally compressed. The centrum is wedge-shaped in lateral view, thicker anteroposteriorly in the ventral side than in the dorsal side. The caudal ribs of Cd1 are the deepest dorsoventrally in all the caudal series, proximally extending from mid-height of the centrum sides to level laterally with the prezygapophyses. They are very thin anteroposteriorly, about 10 mm thick in most of their area. In axial view these are subtriangular in outline, with straight dorsal and ventral margins and tapering to a sharp tip distally. They are obliquely inclined, with the posterior surfaces facing posterodorsally (as the ventral margins root on the posterior centrum articular facet and the dorsal margin on the sides of the prezygapophyses). Instead of proximodorsal projections in the caudal ribs as in the other anterior caudal vertebrae, Cd1 has a closed canal near the dorsal margin (Fig 26C, 26E, 26I and 26K). The dorsal margin of the caudal ribs extends medially over the lateral canals until the base of the neural spine, as a thick rugose ridge, resulting in deep fossae laterally to each postzygapophysis (Fig 26E). The neural canal is oval-shaped in posterior view, with the apex pointing dorsally, but slightly rounder in anterior view (Fig 26C, 26E, 26I and 26K). The pedicels are completely encased in the expanded caudal ribs and their posterior margins merge with the postzygapophyseal ventral margins. The neural spine is long anteroposteriorly and constantly thin transversely–about three times longer than thick transversely–except in the very tip that, although broken off, evidences it was expanding transversely just proximally to the apex (Fig 26C, 26D and 26E). The neural spine points dorsally, but curves gently posteriorly (Fig 26D and 26J). A longitudinal and sharp ridge extends proximally on the anterior side of the neural spine (Fig 26C, 26G and 26I). The articular surfaces of the prezygapophyses face dorsomedially (and slightly anteriorly; Fig 26B, 26H and 26I) while the postzygapophyses face ventrolaterally (and slightly posteriorly; Fig 26D, 26E and 26J). The prezygapophyses are set in separated and anterodorsally projecting peduncles, with a short thick interprezygapophyseal shelf in between roofing the neural canal. The prezygapophyseal facets appear much smaller than the postzygapophyseal facets (which are large and round), but this may be mostly due to the compressive deformation suffered.

**Fig 27:**
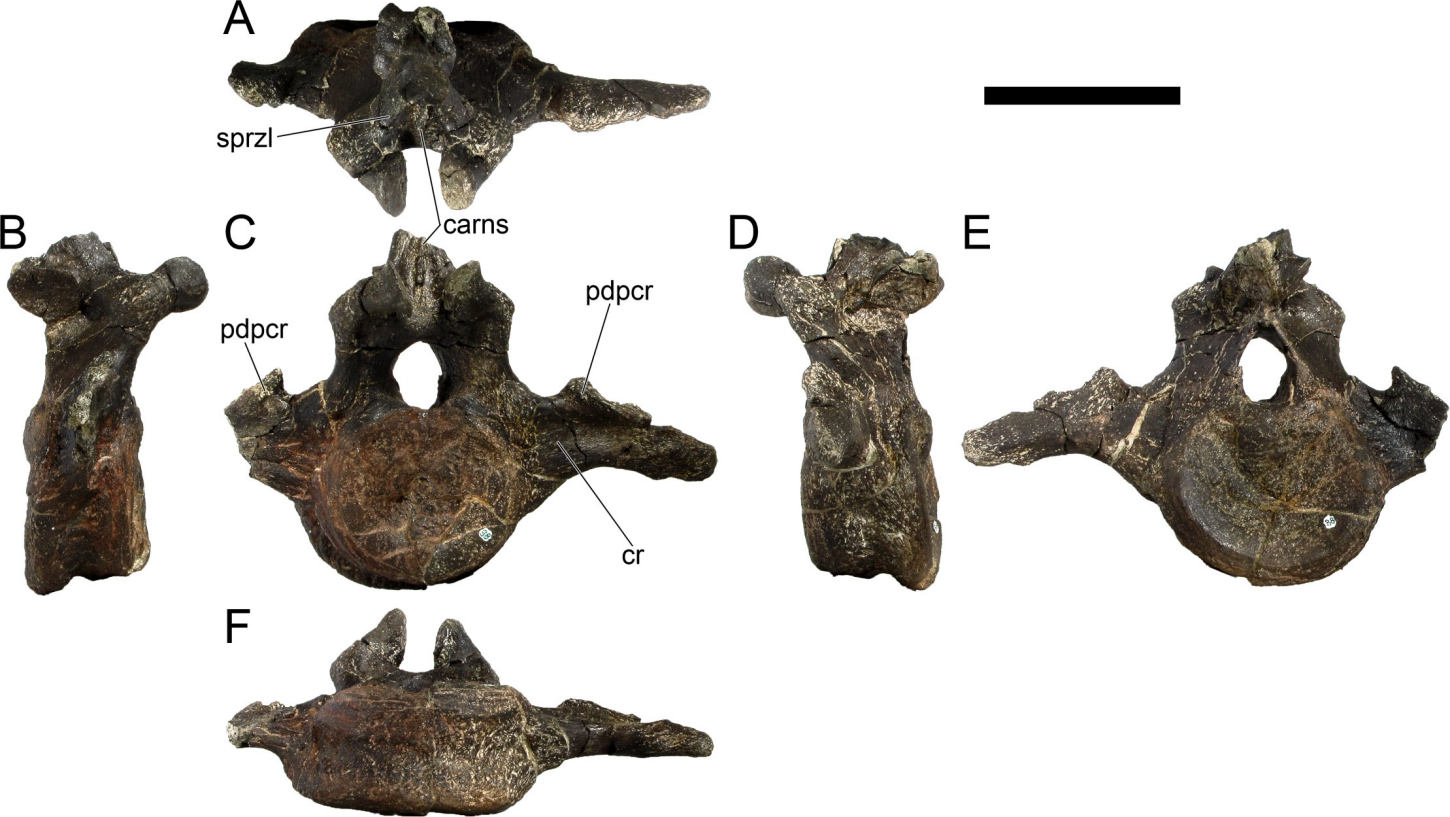
Caudal vertebra two of *Miragaia longicollum* MG 4863. **A**, dorsal, **B**, right lateral, **C**, anterior, **D**, left lateral, **E**, posterior, and **F**, ventral view. **Carns**, central anterior ridge of neural spine, **cr**, caudal rib, **pdpcr**, proximodorsal process of caudal rib, **sprzl**, spinoprezygapophyseal lamina. Scale bar equal to 10 cm.

2nd caudal vertebra (MG 4863–38; Fig 27): The vertebra is the second caudal vertebra, complete except for the neural spine and right rib. It is mostly comparable to the subsequent anterior caudal vertebrae, but shares many features with Cd1. Similarly to Cd1, it has deep caudal ribs, but their proximodorsal margins only extend dorsally until the base of the prezygapophyses (Fig 27C). The neural canal is also relatively large, oval in outline in posterior view but in anterior view it is round (Fig 27C and 27E). The postzygapophyses are also closely spaced together on the base of the neural spine, with large round to oval articular facets and a fossa laterally to each. For these features it shares with Cd1 (also considering that the prezygapophyses of Cd2 fit with the postzygapophyses of Cd1 and their facets are the same size), this vertebra is identified herein as more likely to be the second in the caudal series.

**Fig 28:**
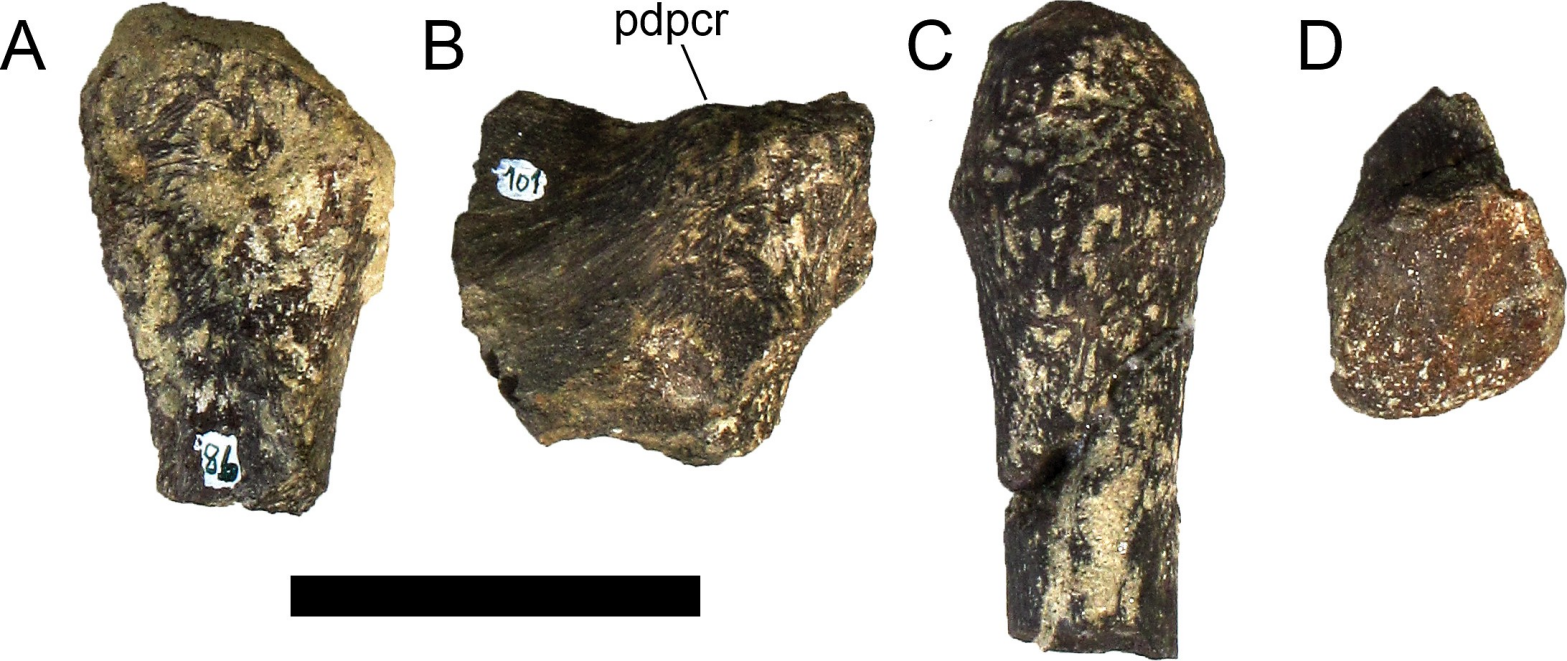
Caudal fragments of *Miragaia longicollum* MG 4863. **A,** MG 4863–98, **B**, MG 4863–101, **C**, MG 4863–103, **D**, MG4863–51 in **A–C**, anterior view, and **D**, left lateral. **Pdpcr**, proximodorsal process of caudal rib. Scale bar equal to 5 cm.

Alike to subsequent anterior caudal vertebrae, the ribs are much thicker anteroposteriorly and do not draw to a tip distally as in Cd1, with a well-developed proximodorsal process in each (Fig 27C and 27E). The caudal ribs are positioned obliquely to the centrum, with the posterior side facing slightly dorsally. The centrum is subround in outline, amphicoelous and consistently thick. The pedicels are wide in the anterior margin, giving them a thick sub-triangular cross section. The prezygapophyses are larger than those in Cd1, extending beyond the centrum facet and lobate in dorsal view. The prezygapophyseal articular facets are round, face dorsomedially and are closely together, almost facing each other. Spinoprezygapophyseal laminae are present, round and thick (Fig 27A). The bases of the prezygapophyses, where the spinoprezygapophyseal lamina and the proximodorsal margins of the caudal ribs converge, are noticeably swollen and rugose. The neural spine is wide, akin to more posterior caudal vertebrae instead of Cd1. The central ridge in the anterior face of the neural spine is expanded anteriorly more than in any other caudal vertebra (Fig 27A and 27C). The distal apex of an anterior caudal neural spine (MG 4863–98; Fig 28A) probably pertains to this vertebra or to Cd3.

**Fig 29:**
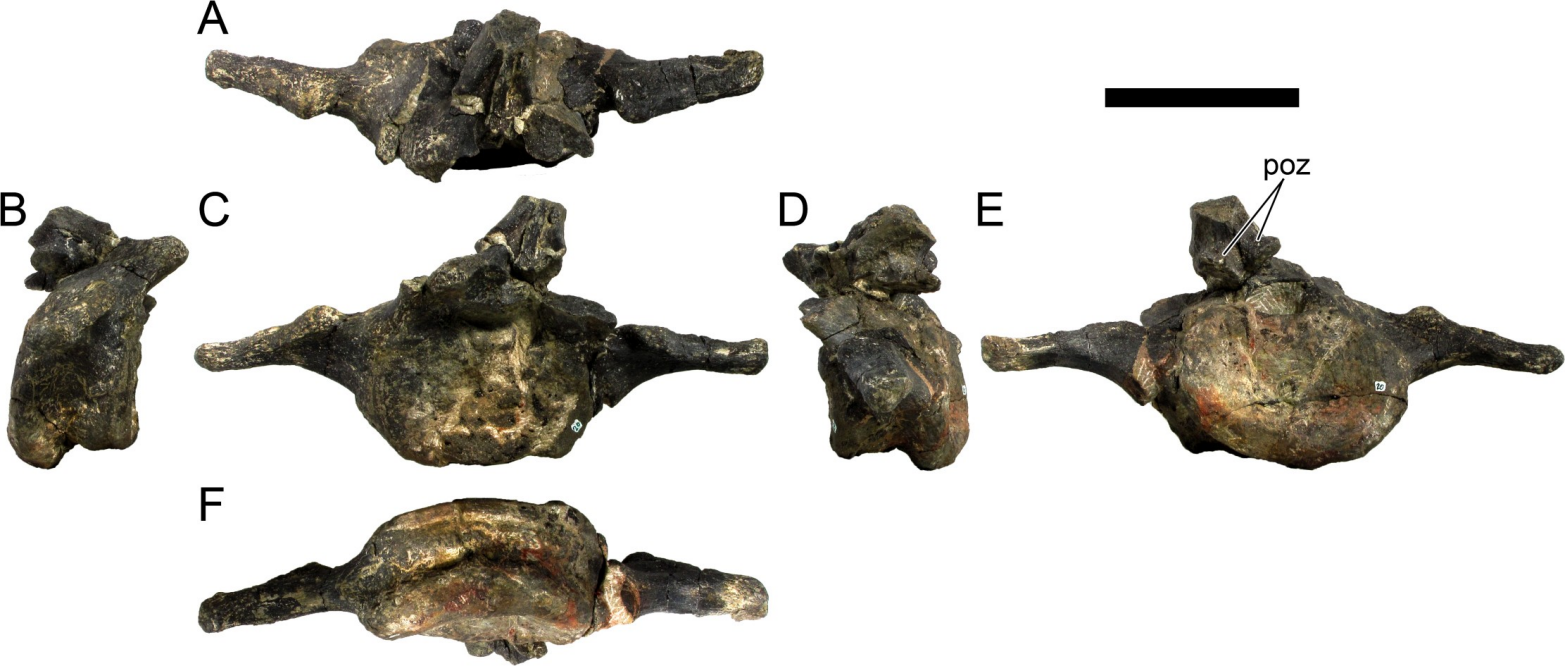
Caudal vertebra three of *Miragaia longicollum* MG 4863. **A**, dorsal, **B**, right lateral, **C**, anterior, **D**, left lateral, **E**, posterior, and **F**, ventral view. **Poz**, postzygapophyses. Scale bar equal to 10 cm.

3rd caudal vertebra (MG 4863–20; Fig 29): This is the third (or possibly fourth) caudal vertebra. It is mostly complete, missing only part of the neural spine and the prezygapophyses, but it is strongly deformed, having suffered high pressure forces dorsoventrally (it was found crushed against the right femur) that flattened the centrum, sheared and deformed the neural arch (Fig 29C). In most visible respects, it is like Cd2, except that the caudal ribs are relatively not as tall dorsoventrally. There appears to be no fossae laterally to the postzygapophyses as in Cd1 and Cd2 and the postzygapophyses are visibly more divided (with a more noticeable concavity between them in dorsal view, but this was probably exaggerated by deformation, as they evidence divergently different sizes).

4th caudal vertebra (MG 4863–101; Fig 28B): From this vertebra only the base of the caudal rib (including its proximodorsal process and part of the transverse process) was found. It is taller than the base of the caudal ribs of MG 4863–21 and not as tall as the ones in MG 4863–20, so it is from the fourth (or fifth) caudal vertebra.

**Fig 30:**
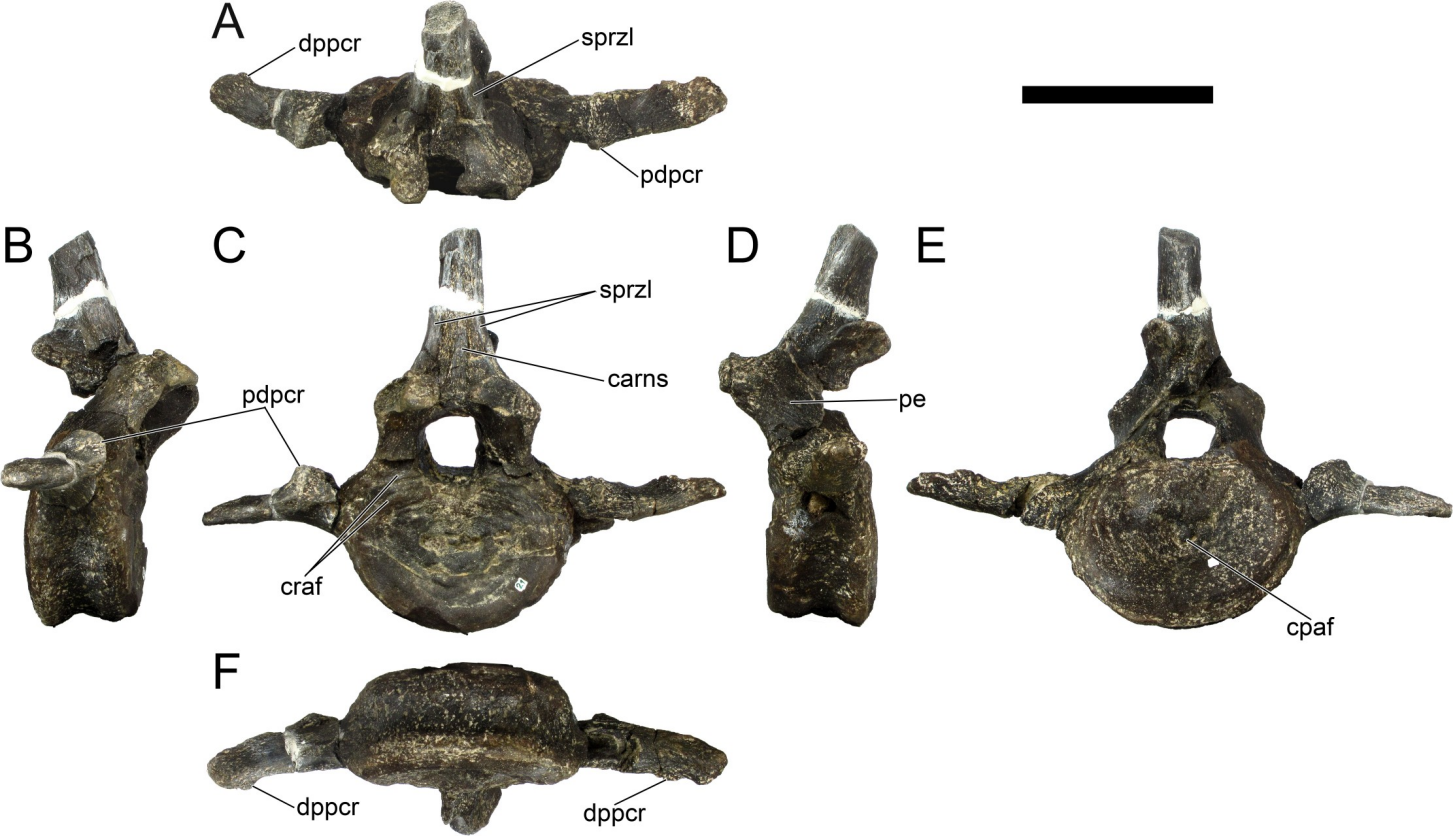
Caudal vertebra five of *Miragaia longicollum* MG 4863. **A**, dorsal, **B**, right lateral, **C**, anterior, **D**, left lateral, **E**, posterior, and **F**, ventral view. **Carns**, central anterior ridge of neural spine, **cpaf**, central process of articular facet, **craf**, concentric ridges of articular facet, **dppcr**, distaloposterior process of caudal ribs, **pe**, pedicel, **pdpcr**, proximodorsal process of caudal rib, **sprzl**, spinoprezygapophyseal lamina. Scale bar equal to 10 cm.

5th caudal vertebra (MG 4863–21; Fig 30): This is the fifth (or possibly sixth) caudal vertebra. Cd5 is almost complete, missing the tips of the prezygapophyses and the apex of the neural spine. The centrum is about twice as wide than long and about 1.5 times wider than tall, with a round but dorsoventrally compressed outline. The centrum is amphicoelous, more deeply excavated in the posterior articular facet. The posterior centrum rim projects ventrally slightly more than the anterior rim (Fig 30B and 30D) and the centrum wall has a gently concave outline in lateral and ventral view. The vertebra has no visible anterior or posterior chevron facets and the ventral side of the centrum is uniformly curved transversely, with no evidence of flattening as in more posterior caudal centra (or a keel as in other stegosaurs). Concentric ridges are present in both centrum facets outer margins, thicker in the anterior facet (Fig 30C and 30E). A round process is present in the center of both centrum faces, much thicker and noticeable in the posterior facet (Fig 30C and 30E). The neural canal is round and relatively large. The pedicels extend over the full length of the centrum and are gently curved anteriorly (Fig 30D). The pedicels are thick transversely, but not as much as in preceding caudal vertebrae–in part since the proximodorsal margins of the caudal ribs are not expanded dorsally over the pedicels as in preceding caudal vertebra, only marginally extending proximodorsally beyond the margin of the centrum. The caudal ribs are long and thin, each almost as wide as the centrum, and slightly longer anteroposteriorly than tall dorsoventrally. The caudal ribs are positioned mid-length of the centrum, project laterally and curve almost imperceptibly posteriorly. A low proximodorsal projection is present in the caudal ribs (Fig 30A–30C), as well as a much smaller distaloposterior projection (Fig 30A and 30F). The neural spine is thick, elliptical in cross section, straight, more posteriorly inclined than preceding caudal neural spines and, like the former, also evidences three proximal ridges on the anterior surface progressing distally (Fig 30A and 30C). If complete, the neural spine would be taller than the centrum. The bases of the prezygapophyses are noticeably swollen and rugose, similarly to what is observed in Cd2. The postzygapophyses are positioned in the base of the neural spine, separated by a concavity posteriorly in ventral view and have large and oval articular facets.

**Fig 31:**
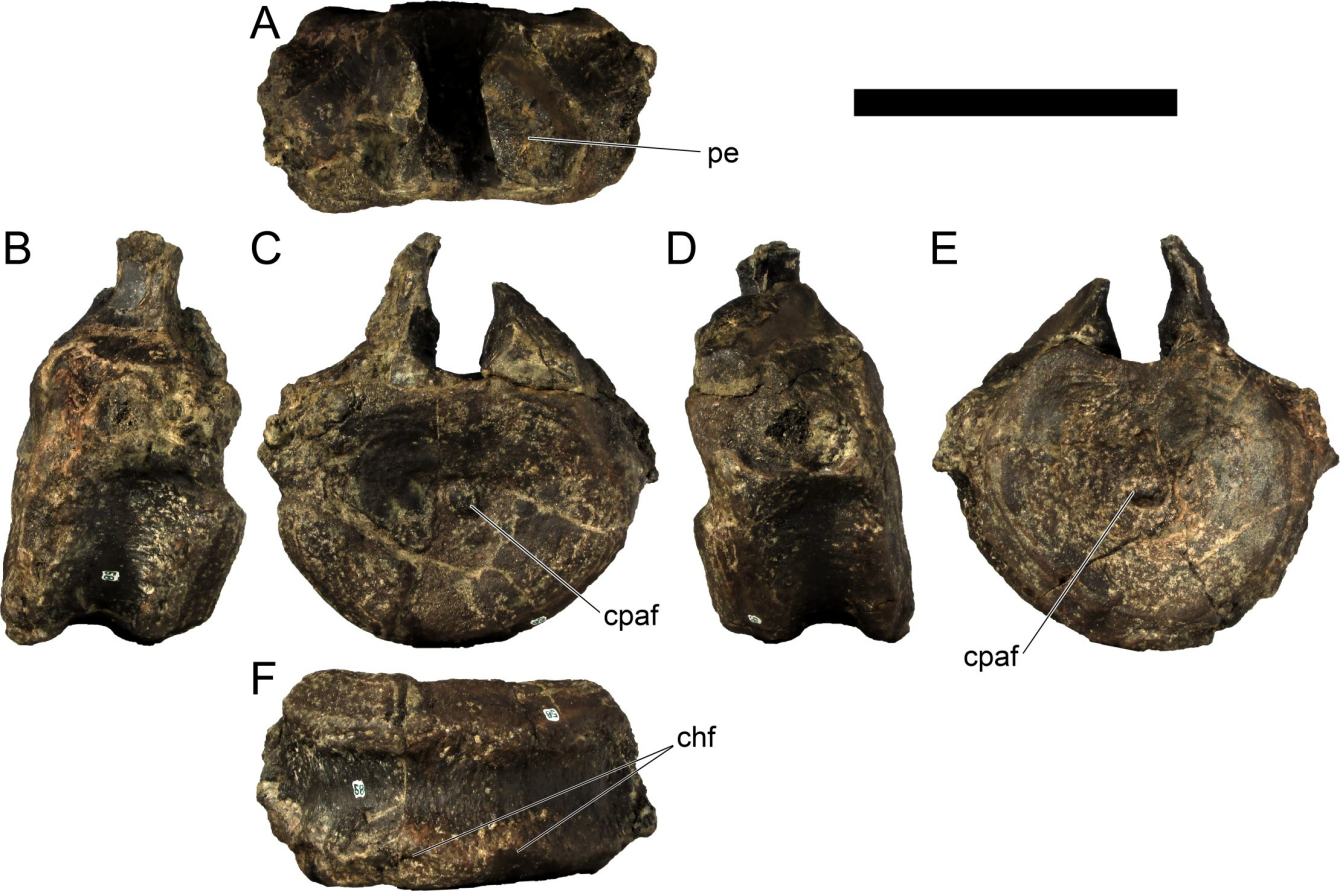
Caudal vertebra seven of *Miragaia longicollum* MG 4863. **A**, dorsal, **B**, right lateral, **C**, anterior, **D**, left lateral, **E**, posterior, and **F**, ventral view. **Chf**, chevron facets, **cpaf**, central process of articular facet, **pe**, pedicel. Scale bar equal to 10 cm.

7th caudal vertebra (MG 4863–58; Fig 31): Centrum with partial pedicels, was posteriorly articulated with Cd8. It is very similar to Cd5, except that the centrum is not as wide, is noticeably taller and longer, giving it a sub-round outline. The pedicels are not as thick transversely, but are still thicker close to the anterior margin, with a subtriangular cross section (Fig 31A). Well-developed processes are present in the center of each centrum facet (Fig 31C and 31E). Unlike preceding anterior caudal vertebrae, posterior chevron facets are present, low but recognizable (Fig 31F). The dorsolateral margins of the transverse processes are flat, not expanding over the pedicels as a lamina as in preceding caudal vertebrae.

The distal half of a neural spine with a transversely and anteroposteriorly expanded apex (MG 4863–103; Fig 28C) is from this or from the 6th caudal vertebra. The apex expands anteroposteriorly as much as transversely and the shaft is elliptical in cross section.

**Fig 32:**
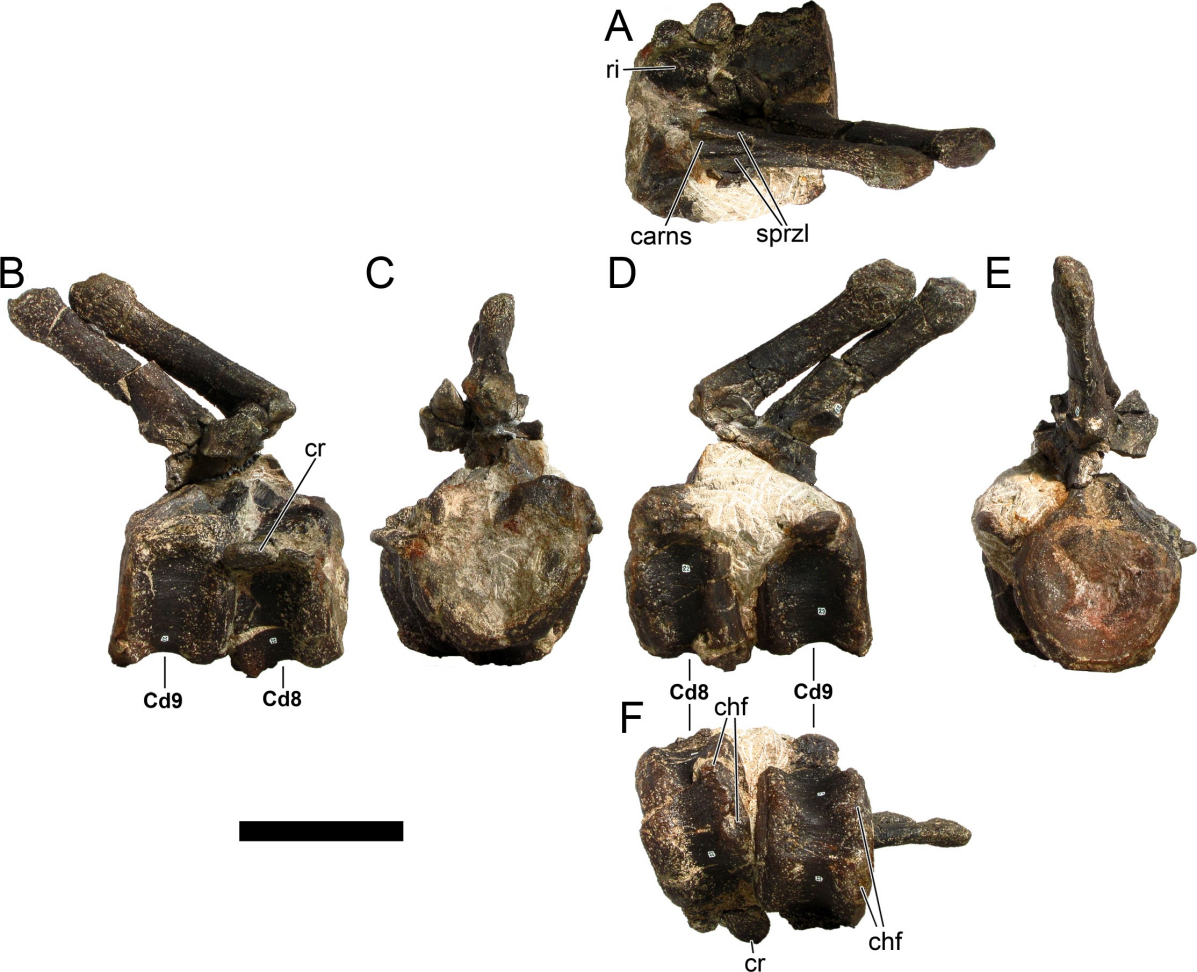
Caudal vertebrae eight and nine of *Miragaia longicollum* MG 4863. **A**, dorsal, **B**, right lateral, **C**, anterior, **D**, left lateral, **E**, posterior, and **F**, ventral view. **Cd8**, caudal vertebra eight, **Cd9**, caudal vertebra nine, **carns**, central anterior ridge of neural spine, **chf**, chevron, **ri**, ridge, **sprzl**, spinoprezygapophyseal lamina. Scale bar equal to 10 cm.

8th caudal vertebra (MG 4863–22; Fig 32): The dorsal portion of the vertebra is still mostly encased in matrix and it articulates posteriorly in full contact with Cd9 (Fig 32), but it is possible to perceive that the anterior surface of the centrum is concave and sub-round in outline. The caudal rib is a dorsoventrally thin and transversely short flap-shaped projection (Fig 32B and 32F). The neural spine is taller than the centrum and neurocanal combined, projects posterodorsally and its distal end is expanded (especially anterodorsally). A ridge with anteroposteriorly oriented rugosities is present dorsally on the base of both transverse processes (Fig 32A). Three ridges are present in the proximal half of the anterior face of the spine. The postzygapophyses are mostly borne on the base of the spine and have round articular facets.

9th caudal vertebra (MG 4863–23; Fig 32): Like Cd8, the dorsal portion of Cd9 is mostly encased in matrix and the anterior side is obstructed by Cd8 –so far as observable, the exposed vertebra appears to be complete. The posterior articulation of the centrum is concave, round and has almost imperceptible concentric ridges on the outer margins. In all other visible respects, it is like Cd8, except that the chevron facets are more evident (Fig 32F), the centrum is slightly longer anteroposteriorly and the neural spine is marginally shorter, thinner and less expanded distally. The prezygapophysis has an oval articular facet.

**Fig 33:**
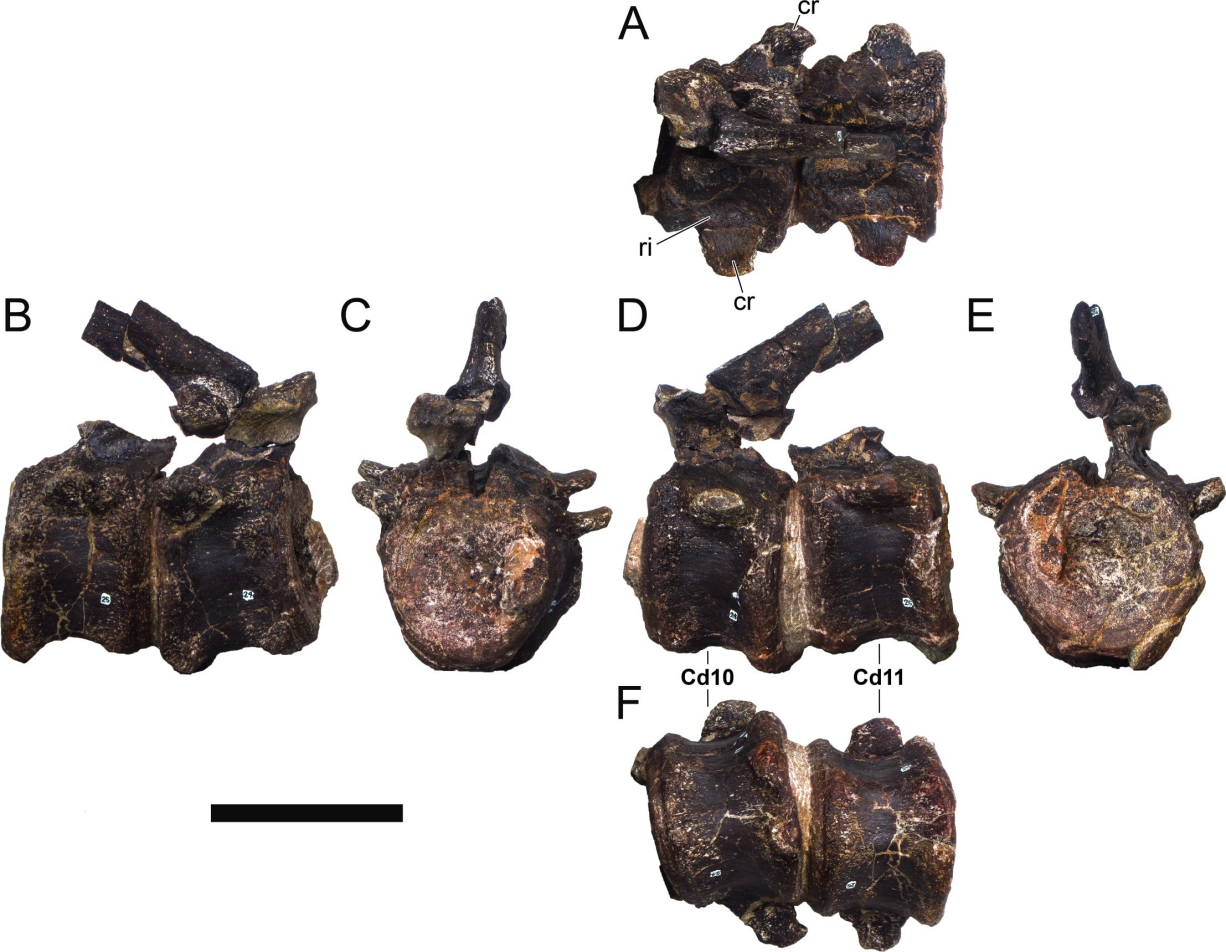
Caudal vertebrae 10 and 11 of *Miragaia longicollum* MG 4863. **A**, dorsal, **B**, right lateral, **C**, anterior, **D**, left lateral, **E**, posterior, and **F**, ventral view. **Cd10**, caudal vertebra 10, **Cd11**, caudal vertebra 11, **cr**, caudal rib, **ri**, ridge. Scale bar equal to 10 cm.

10th caudal vertebra (MG 4863–24; Fig 33): Mostly complete vertebra, but some elements (prezygapophyses and other parts of the neural arch) are possibly still encased in sediment. In articulation by matrix with Cd11 (Fig 33). The anterior articulation of the centrum is concave and evidences a low process in its center. In all other visible respects, it is mostly like Cd9 and Cd8, except that the caudal ribs project also slightly posteriorly (with a concavity in the posterior margin in dorsal view; Fig 33A) and the neural spine (although virtually identical morphologically to the ones in Cd9 and Cd8) is noticeably smaller, shorter, narrower and points slightly more posteriorly. A rugose ridge anteroposteriorly over the base of the transverse processes is observable (homologous to those observed in Cd8 and Cd10), with a noticeable process anteriorly on the right side (Fig 33A). The neural canal appears to be especially narrow and tall, but this is probably an abnormality due to deformation.

11th caudal vertebra (MG 4863–25; Fig 33): Centrum with caudal ribs and part of the pedicels, but the rest of the neural arch is still encased in matrix. Articulated anteriorly by matrix with Cd10. The posterior articulation of the centrum is strongly concave and subround with some depression dorsally by the neural canal. In all other aspects it is essentially identical to Cd10, except that the caudal ribs only extend laterally and the anterior end of the ridge over the right transverse process is even more developed into a distinct process. The anterior margins of the pedicels extend anterodorsally. Although at the time mostly surrounded by matrix, it is possible to observe that the neural spine is alike in morphology to the one in Cd10, but about half the size, while much larger than the one in Cd12.

**Fig 34:**
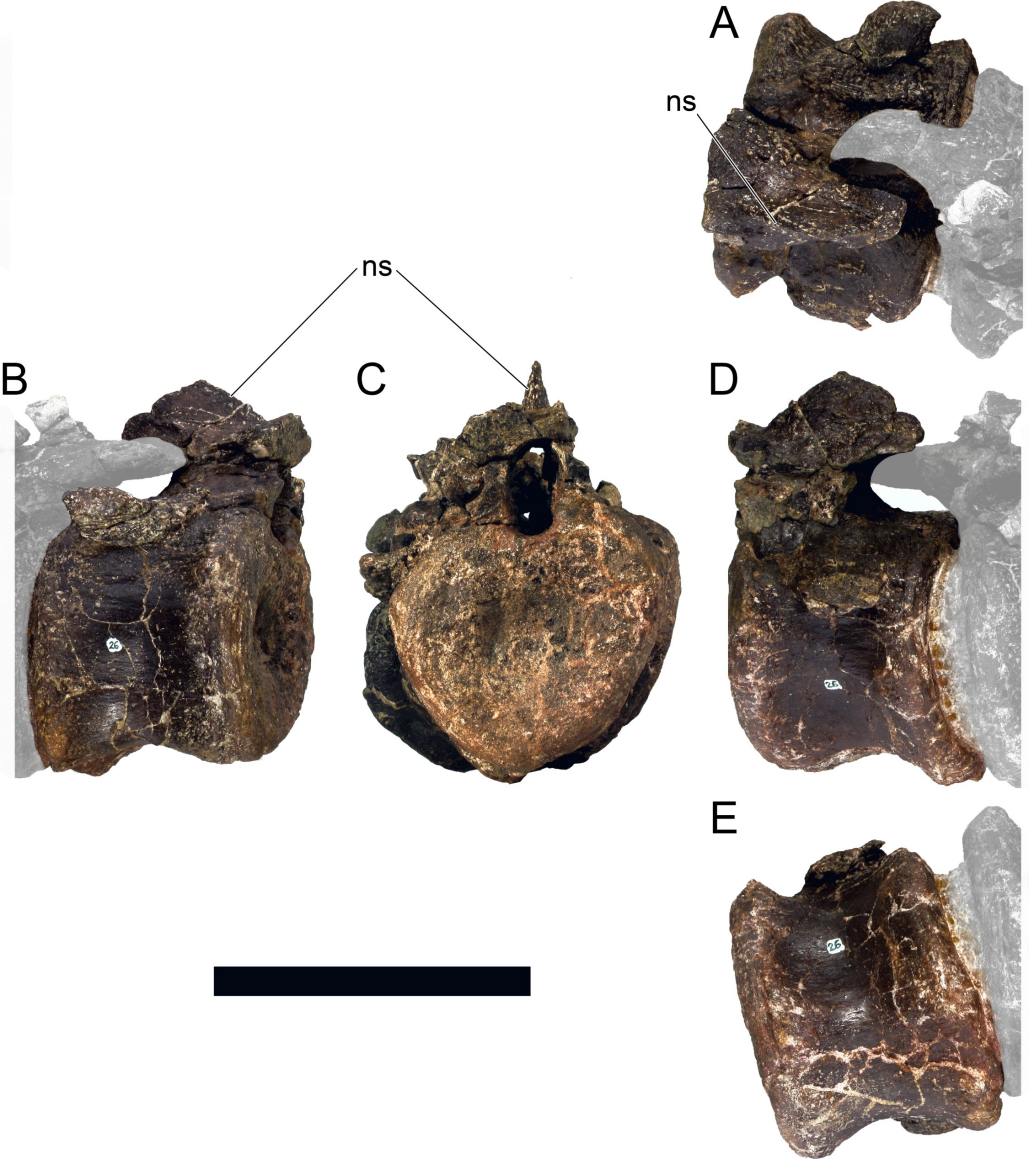
Caudal vertebra 12 of *Miragaia longicollum* MG 4863. **A**, dorsal, **B**, right lateral, **C**, anterior, **D**, left lateral, **E**, posterior, and **F**, ventral view. **Ns**, neural spine. Scale bar equal to 10 cm.

12th caudal vertebra (MG 4863–26; Fig 34): Vertebra missing prezygapophyses and left caudal rib. This vertebra is in most respects similar to Cd11 and Cd10, apart that the transverse processes are much shorter anteroposteriorly and the posterior chevron facets are gently concave. The right rib curves slightly posteriorly alike to Cd10 and an axial rugose ridge is visible dorsally on the transverse process with a well-developed anterior projection. The major difference from preceding caudal vertebrae is the vestigial neural spine–it is transversely thin, less than 9 mm thick in all of its extension, 20 mm tall, round in outline in lateral view, and not expanded in any way axially or transversely (Fig 34A–34C). The neural spine extends anteroposteriorly from the anterior margin of the centrum to the tips of the postzygapophyses. The postzygapophyseal facets are reniform and long.

**Fig 35:**
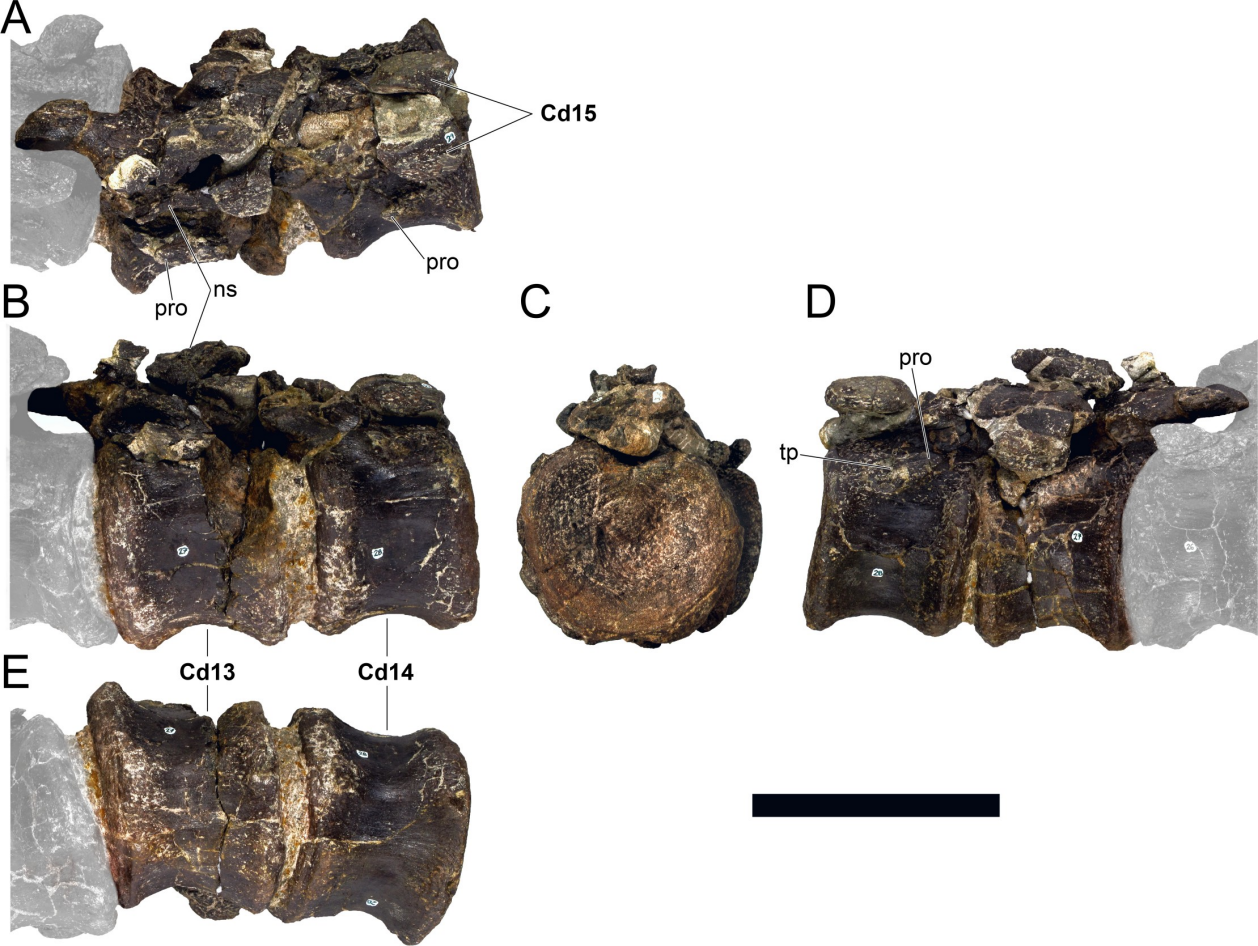
Caudal vertebrae 13, 14 and part of 15 of *Miragaia longicollum* MG 4863. **A**, dorsal, **B**, right lateral, **C**, anterior, **D**, left lateral, **E**, posterior, and **F**, ventral view. **Cd13**, caudal vertebra 13, **Cd14**, caudal vertebra 14, **Cd15**, prezygapophyses of caudal vertebra 15, **ns**, neural spine, **pro**, process, **tp**, transverse process. Scale bar equal to 10 cm.

13th caudal vertebra (MG 4863–27; Fig 35): Cd13 is covered anteriorly and posteriorly by matrix that connects it to Cd12 and Cd14, respectively. The elements of the neural arch are mostly broken and moved out of place. In most visible respects, it is identical to Cd12. The neural spine is broken at its base, but its section evidences it was thin and long as in Cd12 (Fig 35A and 35C). The caudal ribs are shorter anteroposteriorly and positioned more posteriorly. The prezygapophyses extend anteriorly over half the length of Cd12, evidence a rugose longitudinal ridge laterodorsally and have elliptical shaped facets.

14th caudal vertebra (MG 4863–28; Fig 35): This vertebra is articulated anteriorly with Cd13 and the prezygapophyses of Cd15 were preserved in a posterior articulation (Fig 35A and 35C). Cd14 is essentially identical to Cd13. The major difference being that the caudal ribs are vestigial, barely expanding laterally as in previous vertebrae, while the rugose ridge on the dorsal face of the transverse processes is even more noticeable and compact, and its anterior processes are even more expanded, into sharp points overhanging the walls of the centrum (Fig 35A and 35E). The posterior centrum facet is concave and has centrifugally positioned concentric ridges. The pedicels are oriented anterodorsally, so the anterior margins face anteroventrally.

**Fig 36:**
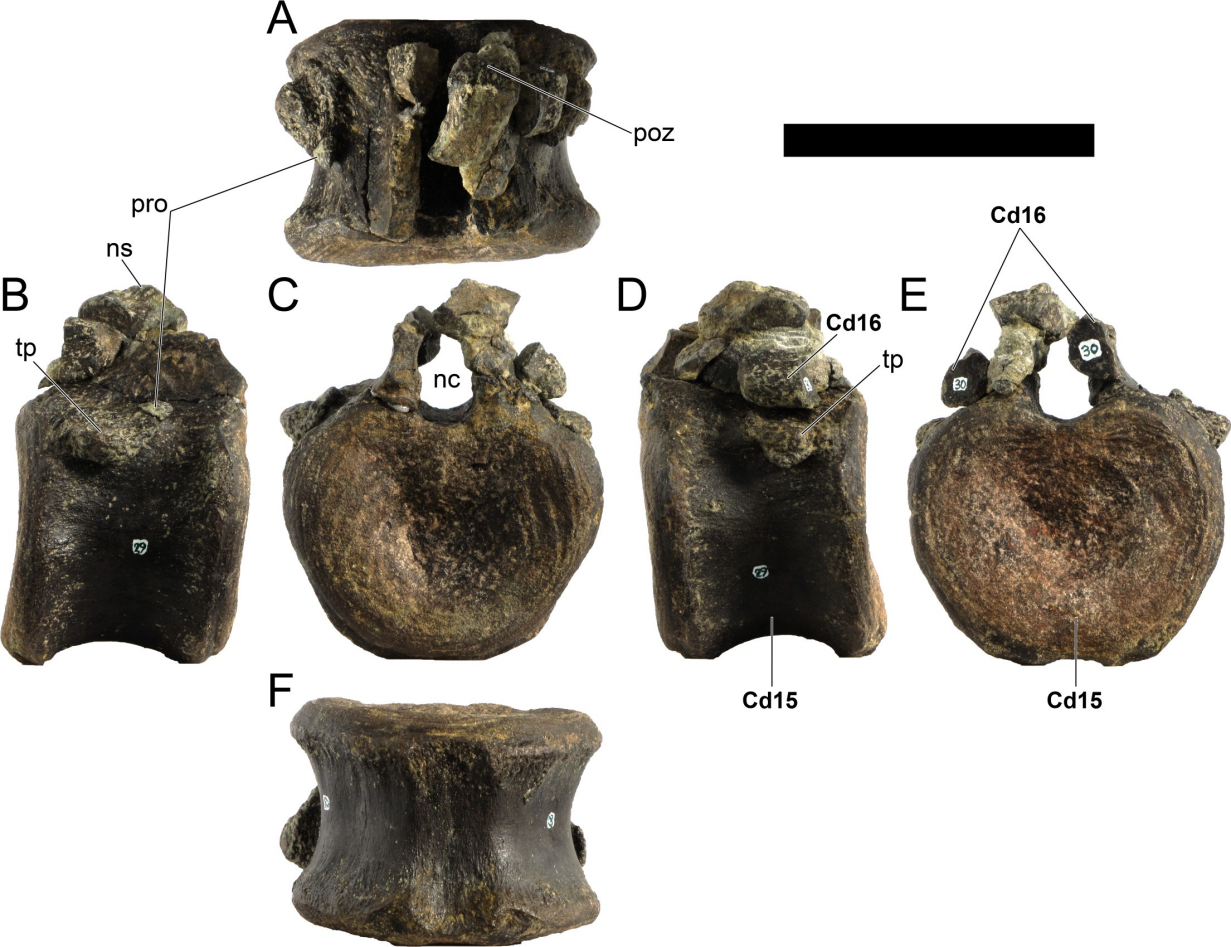
Caudal vertebra 15 and part of 16 of *Miragaia longicollum* MG 4863. **A**, dorsal, **B**, right lateral, **C**, anterior, **D**, left lateral, **E**, posterior, and **F**, ventral view. **Cd16**, prezygapophyses of caudal vertebra 16, **nc**, neural canal, **ns**, neural spine, **poz**, postzygapophyses, **tp**, transverse process. Scale bar equal to 10 cm.

15th caudal vertebra (MG 4863–29; Figs 35 and 36): Centrum with partial pedicels, postzygapophyses and prezygapophyses (the prezygapophyses are separated, articulated with Cd14; Fig 35A and 35C). The prezygapophyses of Cd16 were preserved in articulation with this vertebra (Fig 36D and 36E). It is mostly similar to Cd14. The centrum is amphicoelous, evidencing concentric ridges on the margins of its articular facets and no central process. In outline it is subround to apple-shaped in axial view. The body of the caudal ribs is vestigial and reworked with the transverse processes, which are bulbous, overhang the walls of the centrum (Fig 36A, 36B and 36D) and evidence anterior processes analogous to the ones in Cd10 to Cd14, well developed and pointed (Fig 36A and 36B). The neural canal is twice as tall than wide and elliptical in outline and the neural arch is one third the width of the centrum (Fig 36C and 36E). The postzygapophyseal facets are oval in shape and present almost no bifurcation in between in dorsal view (Fig 36A). A partial low ridge (about 3 mm tall) is present in the dorsal face of the neural arch (suggesting a similar but smaller neural spine to Cd12), but since this part of the vertebra is mostly missing, it is not clear if this is the full height of the neural spine in this vertebra (Fig 36B and 36D).

**Fig 37:**
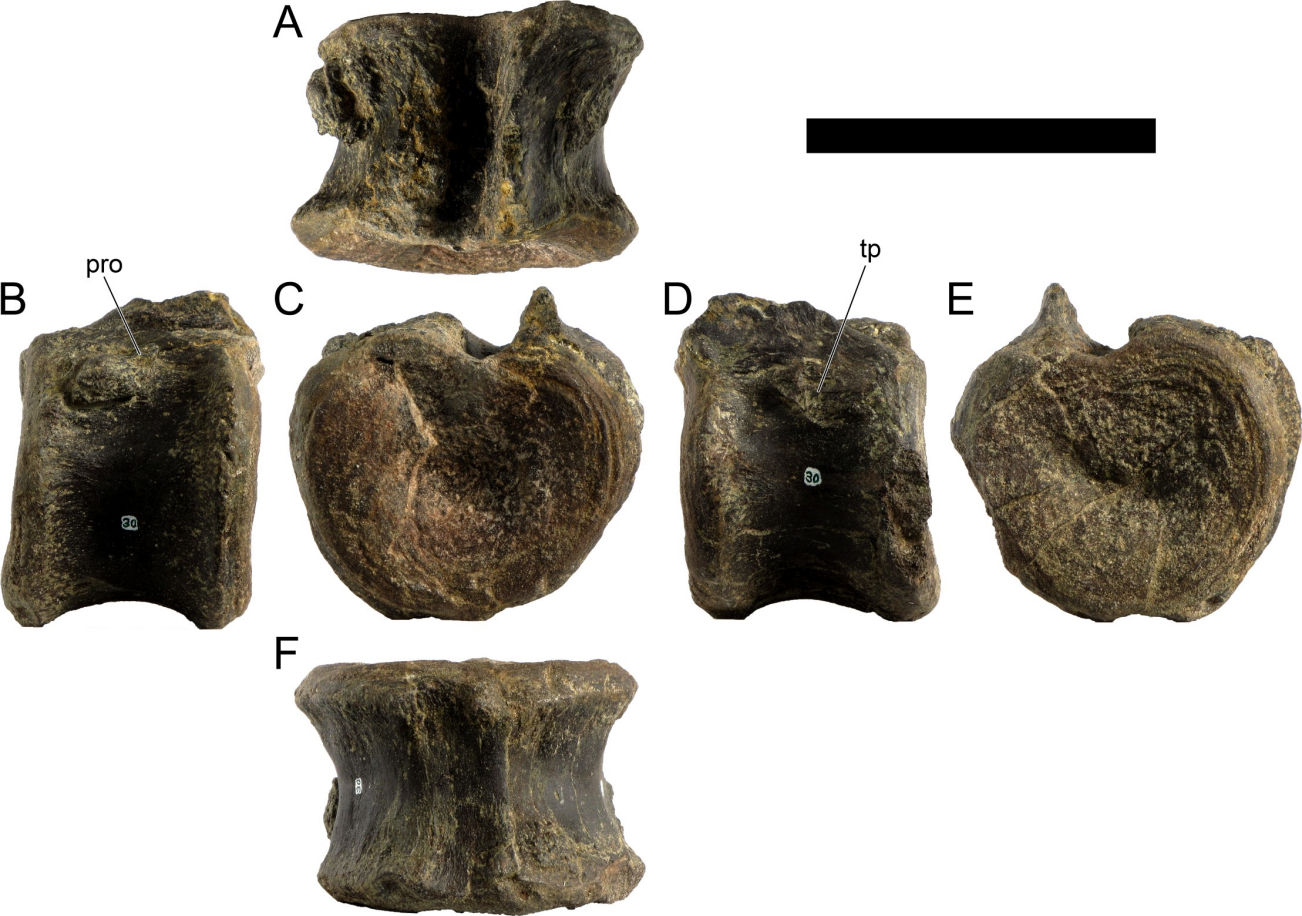
Caudal vertebra 16 of *Miragaia longicollum* MG 4863. **A**, dorsal, **B**, right lateral, **C**, anterior, **D**, left lateral, **E**, posterior, and **F**, ventral view. **Pro**, process, **tp**, transverse process. Scale bar equal to 10 cm.

16th caudal vertebra (MG 4863–30; Figs 36 and 37): Centrum with a partial pedicel and tips of prezygapophyses (the prezygapophyses are separated, articulated with Cd15; Fig 36D and 36E). It is mostly identical to Cd15, except only slightly smaller and one of the transverse processes has a concavity in its middle (at this stage in the caudal series, the body of the caudal ribs is essentially absent, and only the transverse processes remain on the sides of the vertebra; Fig 37D).

**Fig 38:**
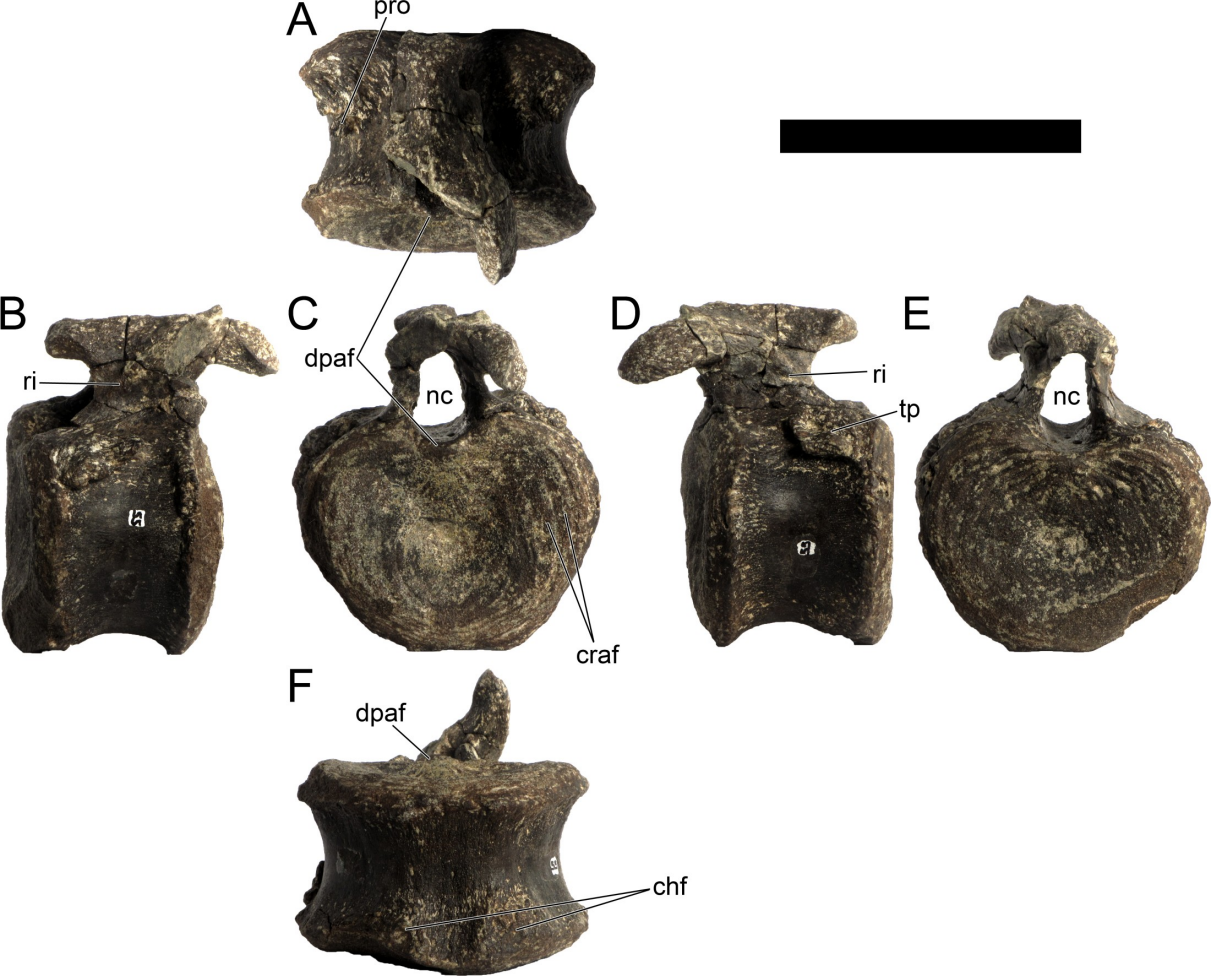
Caudal vertebra 18 of *Miragaia longicollum* MG 4863. **A**, dorsal, **B**, right lateral, **C**, anterior, **D**, left lateral, **E**, posterior, and **F**, ventral view. C**hf**, chevron facets, **craf**, concentric ridges of articular facet, **dpaf**, dorsal process of articular facet, **nc**, neurocanal, **pro**, process, **ri**, ridge. Scale bar equal to 10 cm.

18th caudal vertebra (MG 4863–55; Fig 38): Complete vertebra missing right prezygapophysis and broken right ventroposterior margin of the centrum. The centrum is amphicoelous, 1.3 times wider than tall and 1.6 times wider than long. Both centrum faces evidence concentric ridges, more noticeable on the outer margins and close to the process ventral to the neural canal (Fig 38C and 38E). Both neural canal processes draw to a point beyond the respective articular facet (Fig 38A and 38F). The quality of preservation enables the observation of a texture of sub-millimetric pustules in both articular facets. The centrum outline is round to heart-shaped. The centrum sides are concave all around in the axial plane. The posterior chevron facets are well developed and separated, gently concave in the middle of each (Fig 38F). The anterior chevron facets are subtle tips with a straight centrum ventral margin between them. Between the chevron facets, the centrum wall is flatter and shallow (Fig 38F). The neural canal is teardrop-shaped, with a rounder roof in anterior view (Fig 38C and 38E). The anterior margins of the pedicels are confluent with the anterior centrum face and extend posteriorly over two thirds of the centrum length (Fig 38D). In lateral view, the anterior margin of the pedicels projects anterodorsally and beyond the anterior centrum facet, while the posterior margins evidence a semi-circle concavity that ventrally merges with the dorsoposterior margin of the centrum. The neural canal is three times narrower transversely (from the lateral margins of the pedicels) than the centrum (Fig 38C and 38D). The transverse processes are irregular and asymmetrical–the right one has an anterior process similar to preceding caudal vertebrae and the left one is shallower and concave in the center (Fig 38A and 38D). They are positioned dorsolaterally and on the posterior half of the centrum, contacting the posterior centrum rim. A neural spine is not present, only a blunt ridge remains between the post- and prezygapophyses (Fig 38A and 38B). The prezygapophysis projects anteriorly (it appears to project slightly ventrally because it was broken and moved) and beyond the anterior centrum facet. It is subtriangular in axial cross section. The prezygapophyseal facet is elliptical and faces dorsomedially. A ridge projects ventroposteriorly on the lateral sides of the pedicels from the base of the prezygapophyses, flattening at the posterior margin of the pedicels (Fig 38B and 38D). The postzygapophyseal facets are oval-shaped, smaller than the prezygapophyseal facets and face posteroventrolaterally. The postzygapophyses are separated by a gentle concavity in dorsal view, effectively bifurcating the posterior end of the neural arch.

**Fig 39:**
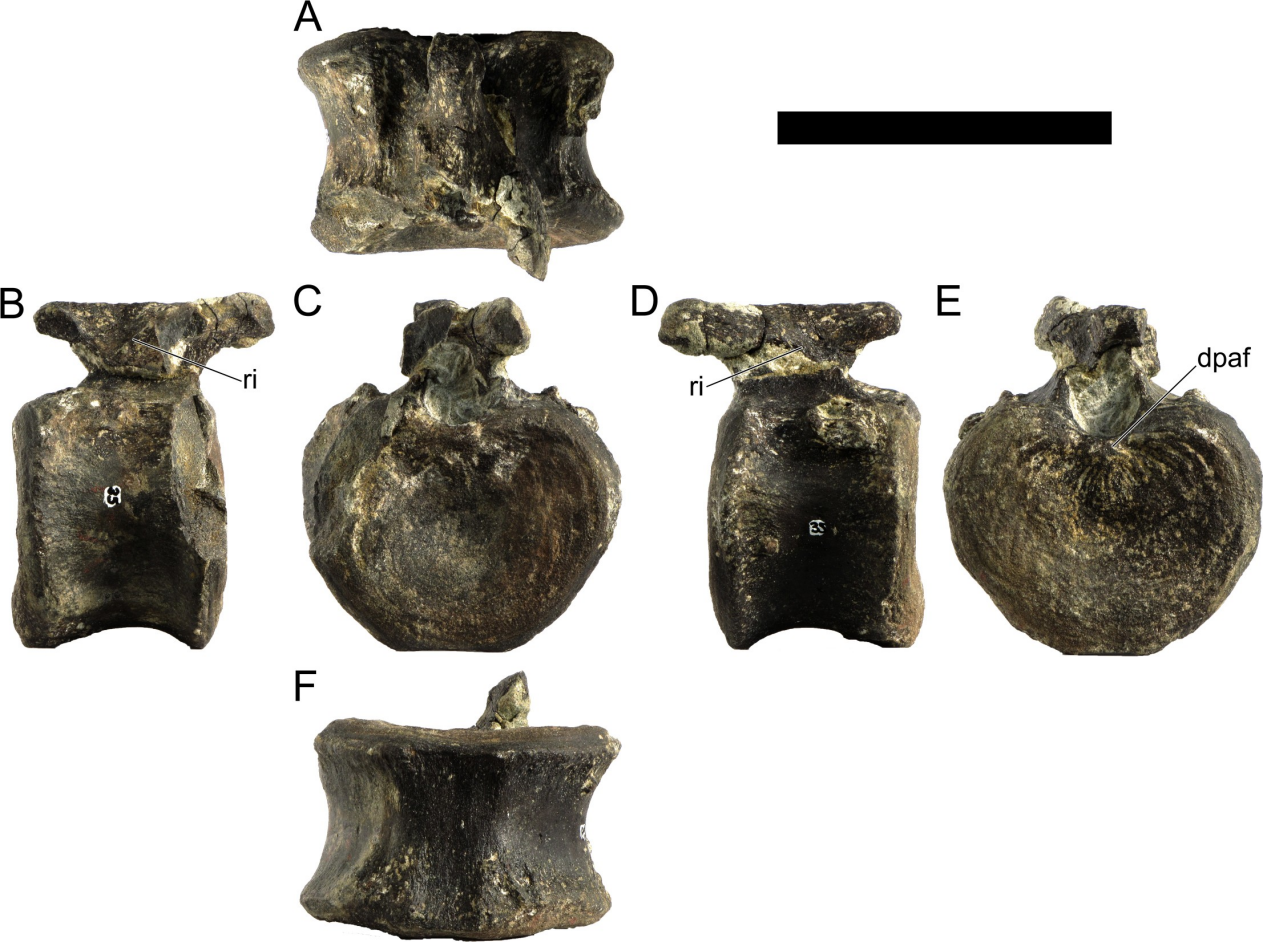
Caudal vertebra 19 of *Miragaia longicollum* MG 4863. **A**, dorsal, **B**, right lateral, **C**, anterior, **D**, left lateral, **E**, posterior, and **F**, ventral view. **Dpaf**, dorsal process of articular facet, **ri**, ridge. Scale bar equal to 10 cm.

19th caudal vertebra (MG 4863–35; Fig 39): Complete vertebra missing the right prezygapophysis. Cd19 is essentially identical to Cd18, except that it is slightly smaller, the transverse processes are not as well developed, the ridges between the prezygapophyses and the posterior margin of the neural canal are not as evident (Fig 39C and 39D), the postzygapophyses are relatively much smaller and the dorsal process in the posterior articular facet is more salient (Fig 39E).

**Fig 40:**
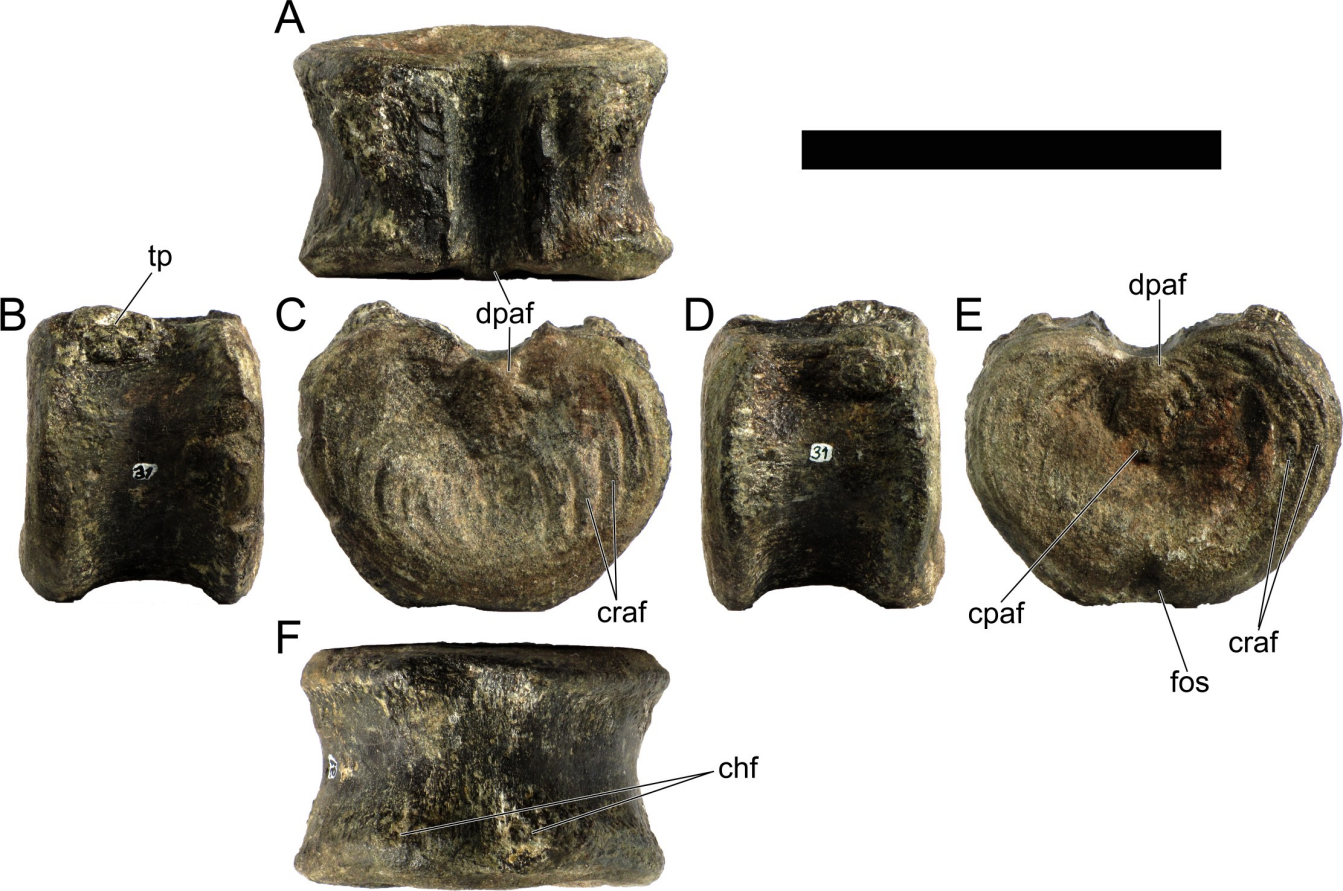
Caudal vertebra 23 of *Miragaia longicollum* MG 4863. **A**, dorsal, **B**, right lateral, **C**, anterior, **D**, left lateral, **E**, posterior, and **F**, ventral view. **Chf**, chevron facets, **cpaf**, central process of articular facet, **craf**, concentric ridges of articular facet, **dpaf**, dorsal process of articular facet, **tp**, transverse process. Scale bar equal to 10 cm.

23rd caudal vertebra (MG 4863–31; Fig 40): Centrum missing whole neural arch. Cd23 is very similar to Cd19, but much smaller and the centrum is slightly more compressed dorsoventrally. The transverse processes are just rugose nubbins that do not overhang or expand as much anteriorly as in Cd18 or Cd19 (Fig 40B and 40D). The concentric ridges on the centrum facets are thick and easily perceivable, and the processes where these converge dorsally in the articular facets are trapezoidal in shape and expand to a point (Fig 40A, 40C and 40E). A low process is present in the center of the posterior centrum face (Fig 40E). The posterior chevron facets are not concave as in more anterior caudal vertebrae (Fig 40F). A shallow fossa is present in the posteroventral margin of the centrum, close to the chevron facets (Fig 40E).

**Fig 41:**
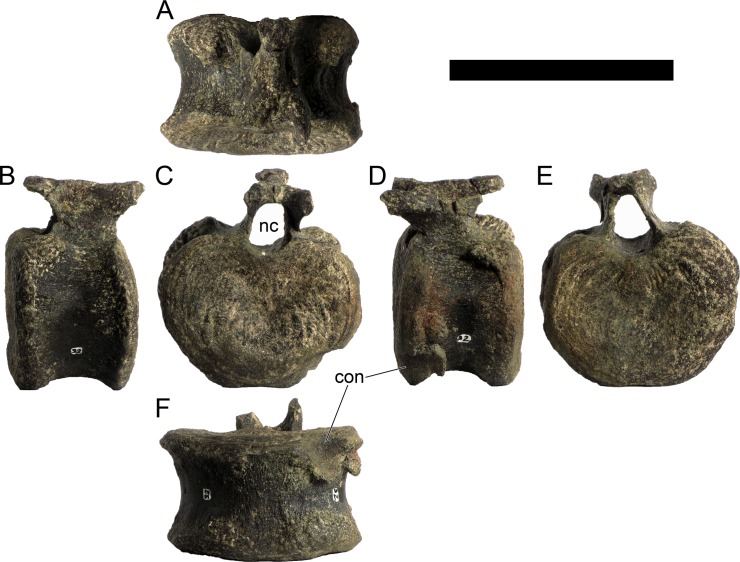
Caudal vertebra 24 of *Miragaia longicollum* MG 4863. **A**, dorsal, **B**, right lateral, **C**, anterior, **D**, left lateral, **E**, posterior, and **F**, ventral view. **Con**, concavity, **nc**, neural canal. Scale bar equal to 10 cm.

24th caudal vertebra (MG 4863–32; Fig 41): Complete vertebra except for the tips of the prezygapophyses that are partially broken. The centrum is amphicoelous, 1.3 times wider than tall and 1.8 times wider than long. The centrum outline is apple-shaped and slightly compressed dorsoventrally. Concentric ridges are present in both articular facets, but have a poor level of preservations and are badly noticeable. The centrum sides are concave in lateral and ventral view. The posterior chevron facets are visible with evident rugosities (Fig 41F). The anterior chevron facets are less conspicuous but are also marked by rugosities. The ventral wall area between the chevron facets is flatter than the rest of the centrum sides and not as concave in lateral view. A low process is present in the center of the posterior centrum facet. In the left anteroventral margin of the centrum a concavity is present, with two projections in the left wall associated, probably due to a physical impact trauma the vertebra suffered in life (Fig 41C–41F). The neural canal is teardrop-shaped, with a rounder roof in anterior view. The pedicels are very thin transversely, about two millimeters thick each. The anterior margins of the pedicels are confluent with the anterior articular facet of the centrum and extend posteriorly over two thirds of the centrum length. The neural arch is much reduced, three times narrower transversely than the centrum and makes up for only one fourth the total height of the vertebra (measured from the base of the pedicels to the dorsal margin). The transverse processes are smaller than in preceding caudal vertebrae, positioned dorsolaterally and posteriorly (over the posterior centrum rim). The neural spine is absent. The prezygapophyses project anteriorly and beyond the centrum articular facet. The prezygapophyseal facets appear elliptical. In dorsal view, the prezygapophyses appear to be separated by a round cleft and parallelly point in the anterior direction. The postzygapophyseal facets are mostly indistinct and only marginally separated in dorsal view.

**Fig 42:**
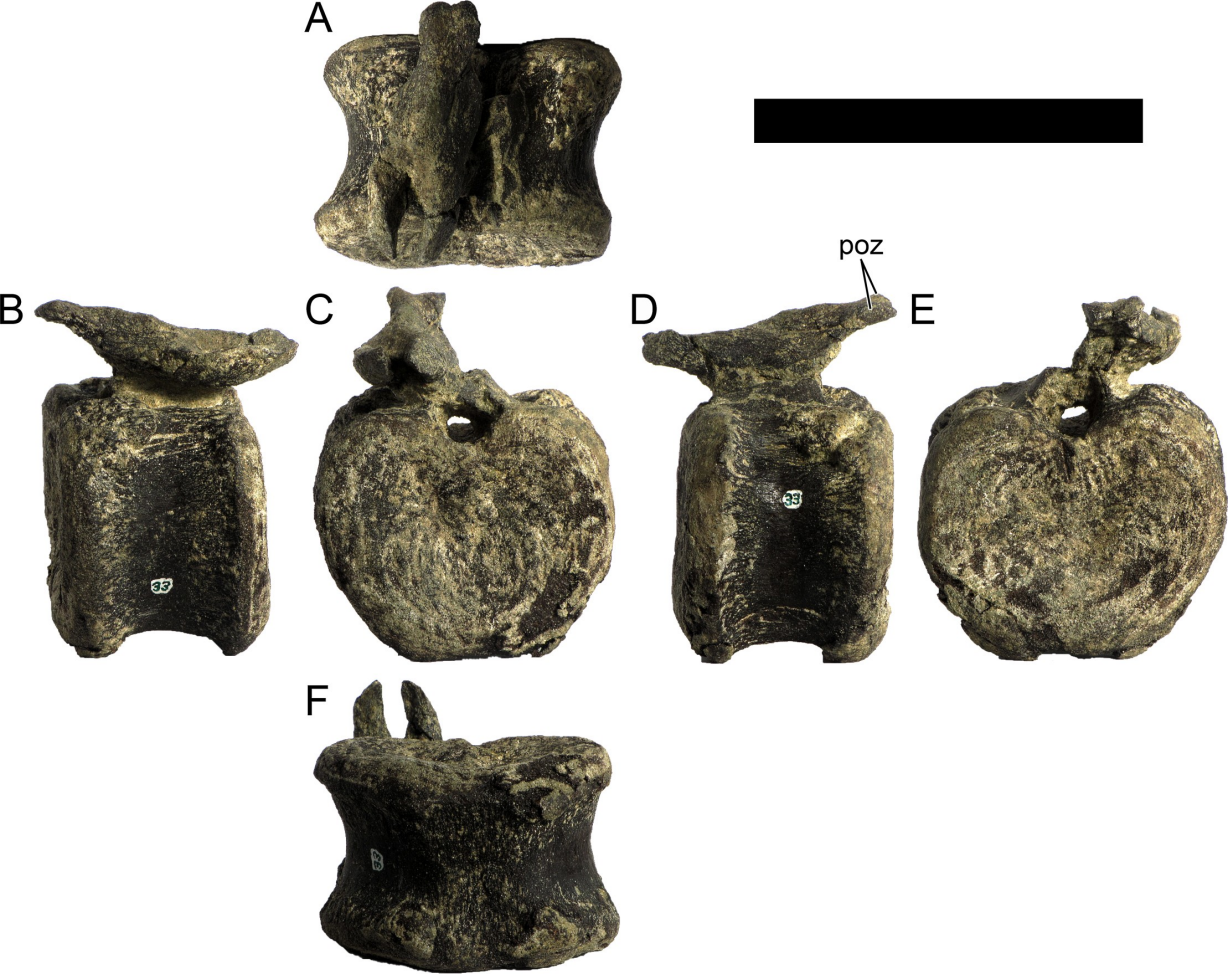
Caudal vertebra 27 of *Miragaia longicollum* MG 4863. **A**, dorsal, **B**, right lateral, **C**, anterior, **D**, left lateral, **E**, posterior, and **F**, ventral view. **Poz**, postzygapophyses. Scale bar equal to 10 cm.

27th caudal vertebra (MG 4863–33; Fig 42): Cd27 is a complete caudal vertebra, mostly identical to Cd24, apart that it is much smaller, the centrum is rounder and not as compressed dorsoventrally. The postzygapophyses appear to extend beyond the posterior centrum facet (Fig 42D) because the neural arch was broken off and moved.

**Fig 43:**
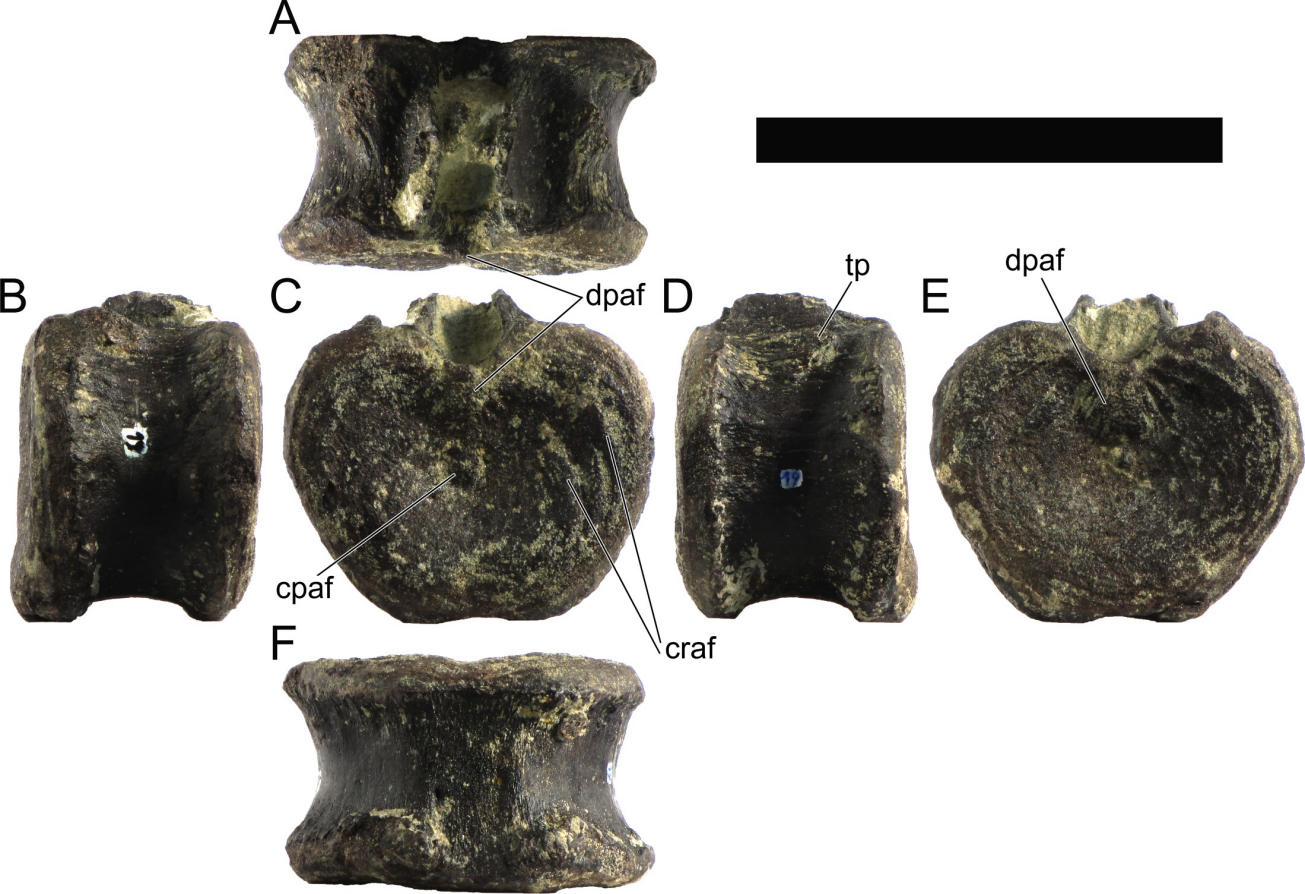
Caudal vertebra 28 of *Miragaia longicollum* MG 4863. **A**, dorsal, **B**, right lateral, **C**, anterior, **D**, left lateral, **E**, posterior, and **F**, ventral view. **Cpaf**, central process of articular facet, **craf**, concentric ridges of articular facet, **dpaf**, dorsal process of articular facet, **tp**, transverse process. Scale bar equal to 10 cm.

28th caudal vertebra (MG 4863–19; Fig 43): Centrum lacking most of the neural arch. It is alike the centrum from Cd27, except that: the transverse processes are positioned more anteriorly (Fig 43D) and are just low rugosities; the concentric ridges (Fig 43D) and neural canal process in the centrum faces (Fig 43A, 43C and 43E) are thicker and more visible; and a pointed process is present in the middle of both centrum surfaces (Fig 43C).

**Fig 44:**
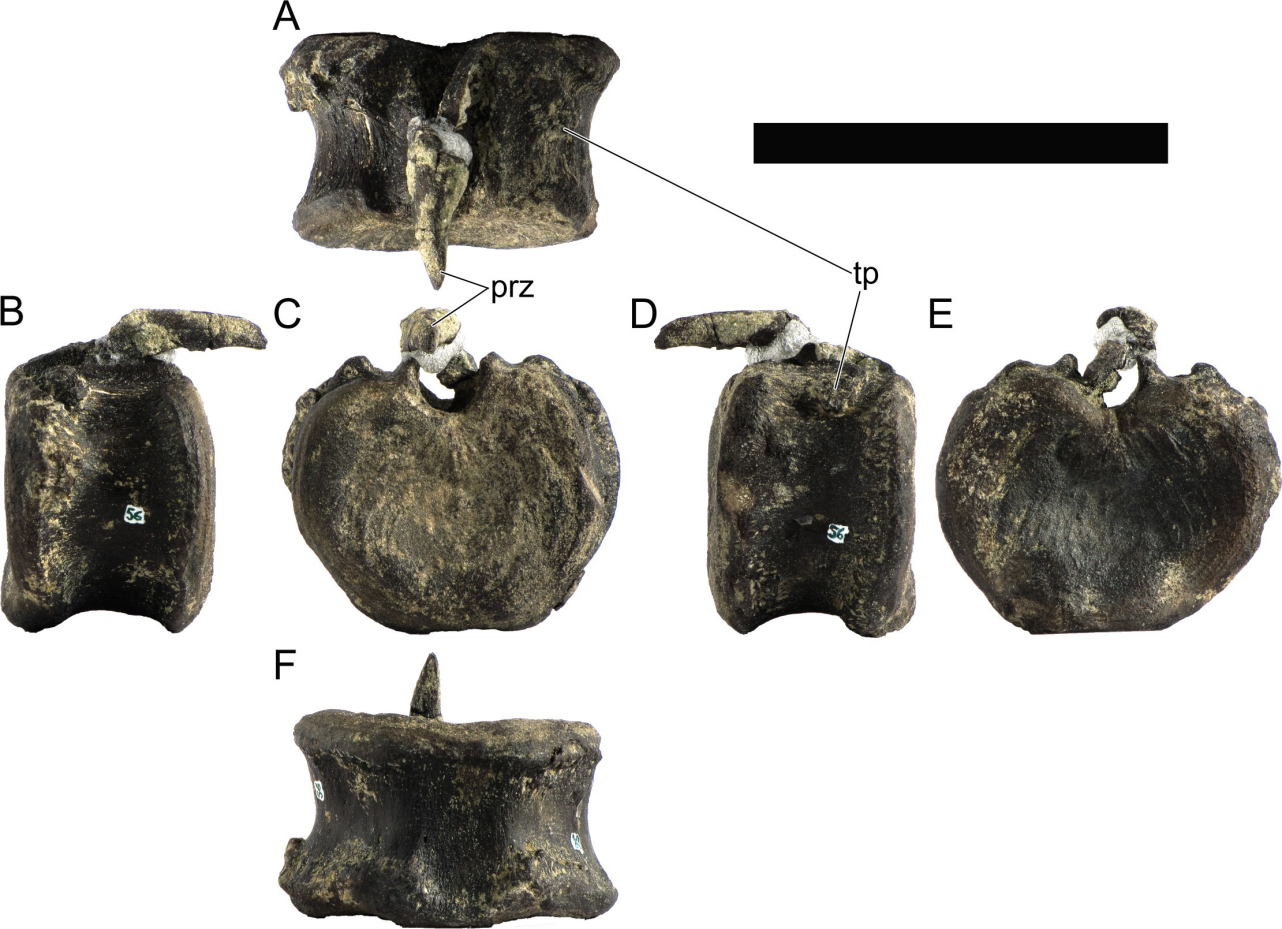
Caudal vertebra 30 of *Miragaia longicollum* MG 4863. **A**, dorsal, **B**, right lateral, **C**, anterior, **D**, left lateral, **E**, posterior, and **F**, ventral view. **Prz**, prezygapophysis, **tp**, transverse process. Scale bar equal to 10 cm.

30th caudal vertebra (MG 4863–56; Fig 44): Centrum with fragments of the neural canal. It is mostly identical to Cd28, but smaller, the transverse processes are rounder (Fig 44A and 44D) and has no process in the middle of the posterior articular facet. The right prezygapophysis (Fig 44A and 44C) is long and thin with no evident facet (it was found associated with Cd30 and later reconstructed with it, but it is possible it belongs to another caudal vertebra).

**Fig 45:**
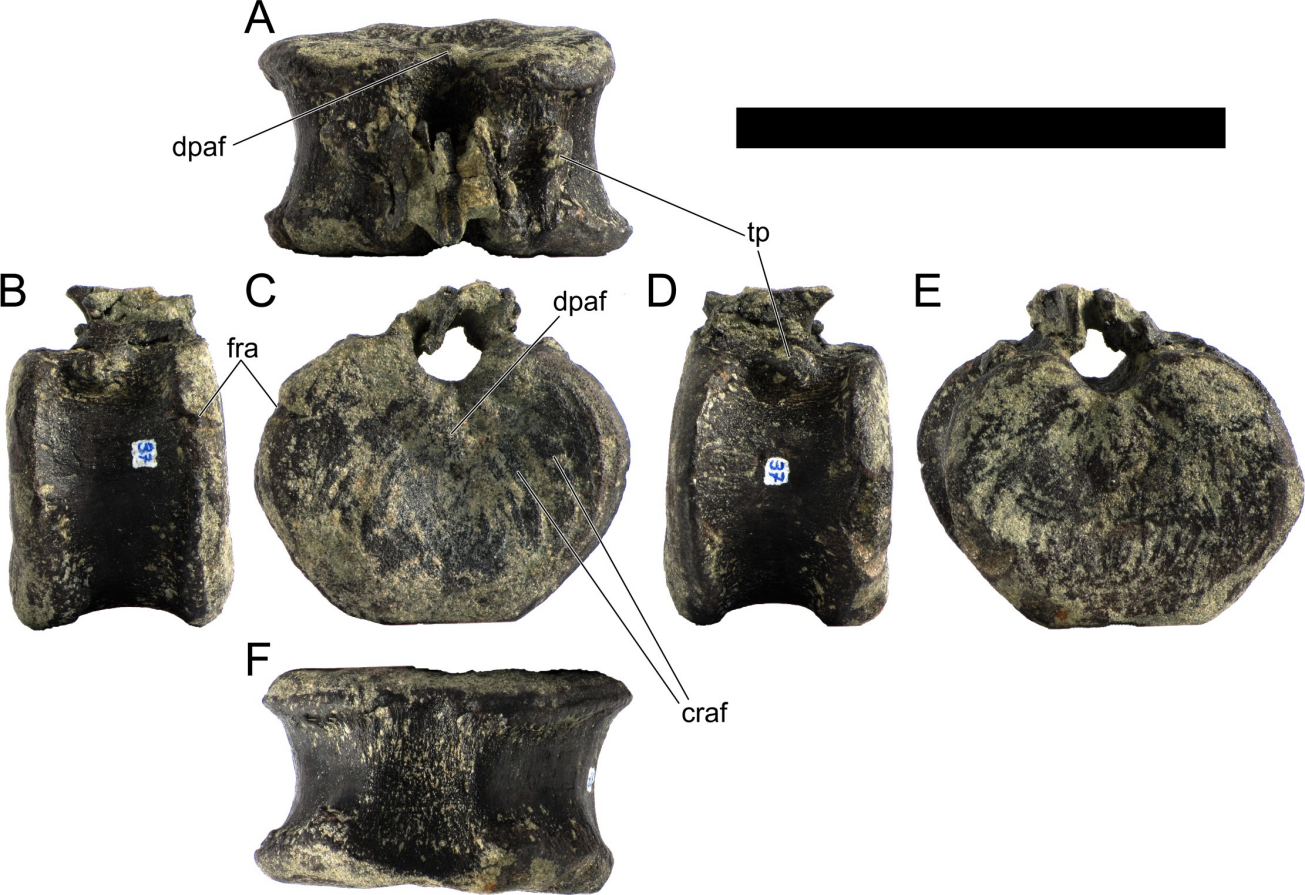
Caudal vertebra 32 of *Miragaia longicollum* MG 4863. **A**, dorsal, **B**, right lateral, **C**, anterior, **D**, left lateral, **E**, posterior, and **F**, ventral view. **Craf**, concentric ridges of articular facet, **dpaf**, dorsal process of articular facet, **fra**, fracture, **tp**, transverse process. Scale bar equal to 10 cm.

32nd caudal vertebra (MG 4863–37; Fig 45): Centrum with pedicels and transverse processes. Centrum is amphicoelous, 1.2 times wider than tall and twice as wide as long. In axial view it has an apple-like outline, with a flattened base and round neural canal that deepens in a semi-circle concavity dorsally. Concentric ridges, thick and well defined, are present in both centrum surfaces (Fig 45C), in each converging dorsally in a process ventral to the neural canal (Fig 45C). These anterodorsal processes in the articular facets are round in shape and, in dorsal view, project to a tip (Fig 45A). A texture of multiple miniscule pustules covers both articular surfaces. The centrum rims are well defined, giving a concave outline all around on the transverse plane. The lateral and ventrolateral surfaces are smooth, while the ventral surface between the chevron facets is slightly but noticeably more rugose and concave. The posterior chevron facets are only slightly differentiated from the posterior centrum rim. The transverse processes are ball-shaped on the surfaces of the centrum (Fig 45A and 45D), relatively rounder and smoother than present in other caudal vertebra, positioned dorso-laterally (close to dorsally) and equidistant anteroposteriorly. The neural canal has a curved floor and is about 13 mm wide. The pedicels are thin, about 3mm thick each, and insert from the anterior edge of the centrum to about two thirds the length posteriorly. The neural arch is one third the width of the centrum. A fracture in the right antero-lateral border (Fig 45B and 45C) is filled with either reconstructive bone or foreign (*i*.*e*., part of the tooth of a predator or scavenger).

**Fig 46:**
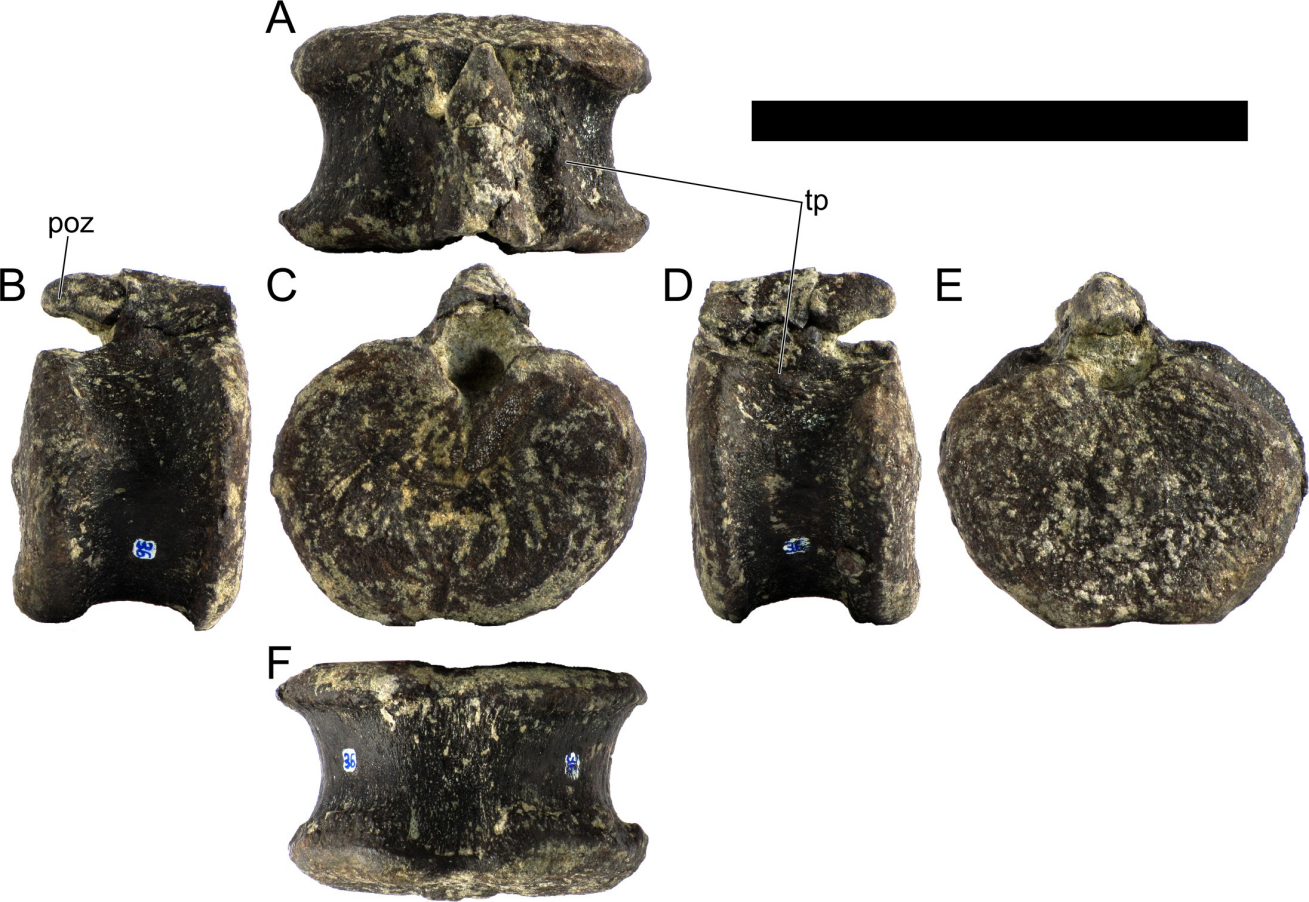
Caudal vertebra 34 of *Miragaia longicollum* MG 4863. **A**, dorsal, **B**, right lateral, **C**, anterior, **D**, left lateral, **E**, posterior, and **F**, ventral view. **Poz**, postzygapophyses, **tp**, transverse process. Scale bar equal to 10 cm.

34th caudal vertebra (MG 4863–36; Fig 46): Complete vertebra missing just the prezygapophyses. Cd34 is similar to Cd32, except slightly smaller, the transverse processes are almost imperceptible, refraining to low ridges extending posteriorly to mid length of the centrum (Fig 46A and 46D), the concentric ridges on the surfaces are not so visible or well preserved, the processes ventral to the neural canal are not present and the anterior centrum facet is flatter and gently convex (making the vertebra procoelous). The dorsal surface of the neural arch is a blunt longitudinal ridge. The postzygapophyses are indistinguishable, with no bifurcation between these, so the postzygapophyses are reduced to a small featureless posterior projection with a subtriangular cross-section (Fig 46B).

**Fig 47:**
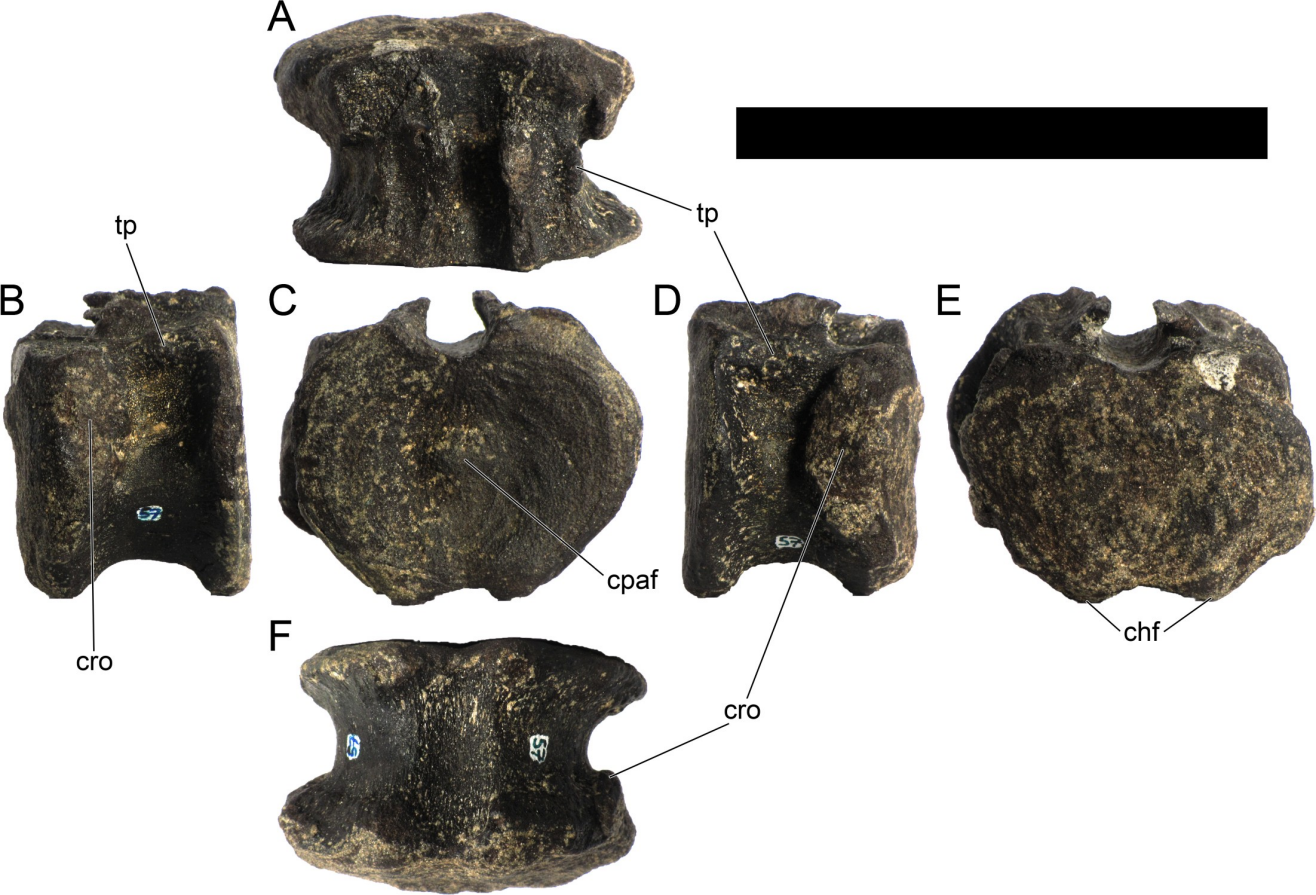
Caudal vertebra 36 of *Miragaia longicollum* MG 4863. **A**, dorsal, **B**, right lateral, **C**, anterior, **D**, left lateral, **E**, posterior, and **F**, ventral view. **Chf**, chevron facets, **cpaf**, central process of articular facet, **cro**, lateral centrum rim ossification, **tp**, transverse process. Scale bar equal to 10 cm.

36th caudal vertebra (MG 4863–57; Fig 47): Centrum retaining part of the pedicels. The anterior articular facet is concave, but much flatter than in the preceding caudal centra, while the posterior facet is slightly convex, as such the centrum is procoelous but closely acoelous. Subtle concentric ridges are present in the anterior centrum surface, while the posterior surface is irregular with poorer preservation, but concentric ridges are also faintly visible. A round process is present in the center of the anterior articular facet (Fig 47C), much more conspicuous than those in other centrum facets and is relatively and absolutely the largest present in all caudal series. The centrum outline in axial view is round and compressed dorsoventrally. The sides of the centrum are markedly concave in dorsal and ventral view, more than in preceding caudal centra. The posterior centrum rims are extraordinarily thick, much more than in any preceding centra or the anterior centrum rim. This is mostly visible on the dorsolateral sides of the rim, evidencing additional symmetrical ossification anteriorly, thus extending over almost all the posterior half of the centrum (Fig 47D and 47F). The area between the chevron facets is furthermore concave, resulting in dorsally arched anteroposterior ridges between each anterior and posterior facet. The neural canal is round in outline in axial view. The pedicels are low and their posterior margins curve sharply backwards, giving it a strongly concave outline in lateral view.

**Fig 48:**
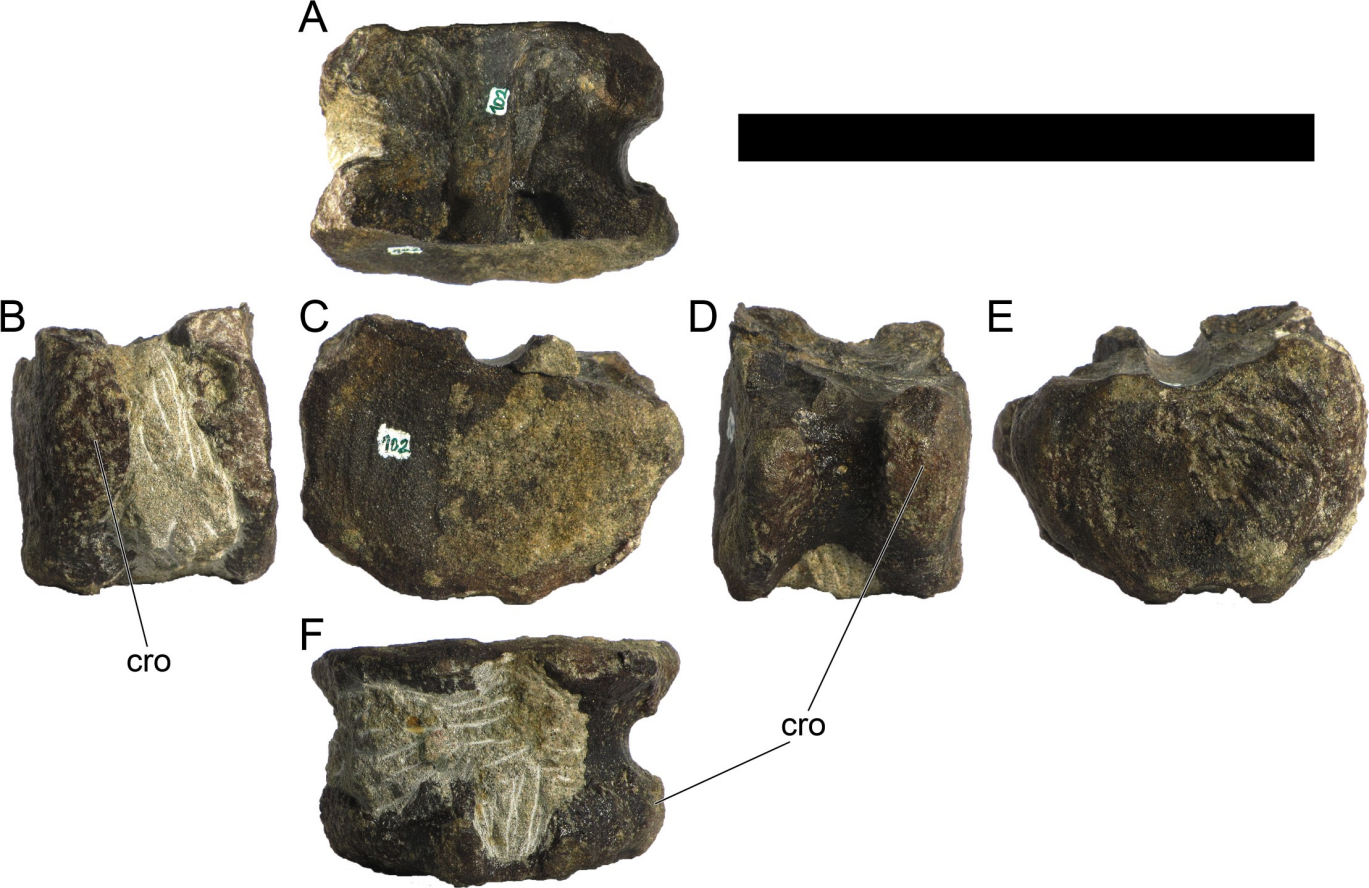
Centrum of caudal vertebra 37 of *Miragaia longicollum* MG 4863. **A**, dorsal, **B**, right lateral, **C**, anterior, **D**, left lateral, **E**, posterior, and **F**, ventral view. **Cro**, lateral centrum rim ossification. Scale bar equal to 10 cm.

37th caudal vertebra (MG 4863–102; Fig 48): Centrum with parts of the neural canal encased in matrix. The 37th caudal vertebra is almost identical to Cd36, differing mostly in the fact that the centrum is slightly more compressed dorsoventrally, the anterior articular facet is much flatter with no process in its center (so the centrum is acoelous) and the transverse processes are reduced to a vestigial ridge. It has extraordinary ossification on the centrum posterior rim (Fig 48B and 48D) much like Cd36, and the anterior rim is also additionally thick in a similar manner, although not as much as the posterior rim. The neural arch is mostly still encased in matrix, but it is possible to observe that the neural canal is round, slightly taller than wide, the prezygapophyses project beyond the anterior articular facet, the neural arch is one third the width of the centrum and the pedicels project posteriorly from adjacent to the anterior centrum facet to less than half the length of the centrum.

#### Dorsal ribs

**Fig 49:**
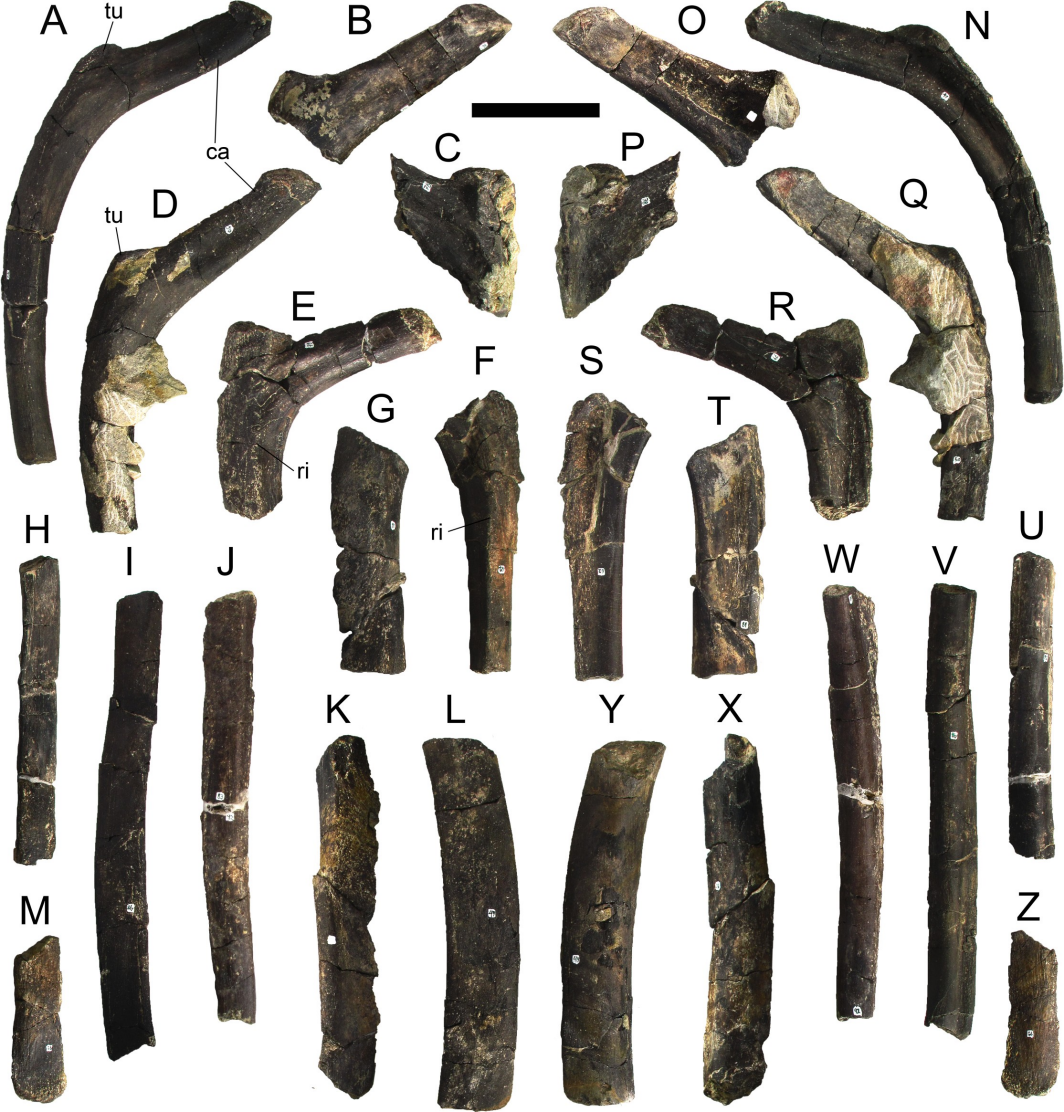
Dorsal ribs of *Miragaia longicollum* MG 4863. Only the most complete partial dorsal ribs are figured. **A, N**, MG 4863–47, **B, O**, MG 4863–75, **C, P**, MG 4863–78, **D, Q**, MG 4863–50, **E, R**, MG 4863–79, **F, S**, MG 4863–62, **G, T**, MG 4863–81, **H, U**, MG 4863–82, **I, V**, MG 4863–48, **J, W**, MG 4863–83, **K, X**, MG 4863–80, **L, Y**, MG 4863–49, **M, Z**, MG 4863–92, in **A–G**, anterior, **H–M**, lateral, **N–T**, posterior and **U–Z**, medial view. **Ca**, capitulum, **ri**, ridge, **tu**, tuberculum. Scale bar equal to 10 cm.

No complete dorsal rib was found, nor any dorsal rib articulated with a dorsal vertebra. Several partial dorsal ribs and fragments of dorsal ribs were found (including proximal, medial and distal parts, from both the left and right side), but even after reconstructive efforts few parts were conclusively fit together. As such, the ribcage is represented by an array of dispersedly arranged and not continuous fragments (Fig 49), and the total length and number of dorsal ribs is unclear.

In general, the dorsal ribs of MG 4863 are long and thin, with a short tubercular process and a long capitulum set at a gentle obtuse angle with the rib shaft (Fig 49A, 49D, 49N and 49Q). The section between the proximal end and the tuberculum is compressed anteroposteriorly, gently concave anteriorly and posteriorly (giving it a dumbbell-shaped cross section). The section adjacent ventrally to the capitulum is the widest part of the rib dorsoventrally and on its posterior face has the most deeply excavated concave area of the rib, while on the anterior face it is convex and often with a longitudinal medial ridge (Fig 49E and 49F). Progressing distally from the tuberculum, the concavity on the posterior side becomes less pronounced, until the rib has a round or semi-round cross section at mid-length, onwards becoming increasingly more compressed transversely (acquiring an elliptical cross section at two thirds the length) until ultimately becoming flat at the distal end (Fig 49M and 49Z). The angles of the capitula with the rib shafts vary slightly, but generally tend to have a 45 degrees with the rib shafts, gently curved on the dorsal and ventral margins, although some ribs (such as MG 4863–79; Fig 49E and 49R) have an angle closer to perpendicular. The tubercula generally are almost flat with the dorsal margins of the ribs, only marginally diverging from it, but some ribs evidence a more pronounced tuberculum (such as MG 4863–78 and MG 4863–79; Fig 49C, 49E, 49P and 49R). The articular surface of the tuberculum faces ventroposteriorly, is elliptical in shape and gently concave (Fig 49N), while the articular surface of the capitulum is slightly expanded from the rest of the body of the capitulum (Fig 49A and 49D).

#### Chevrons

**Fig 50:**
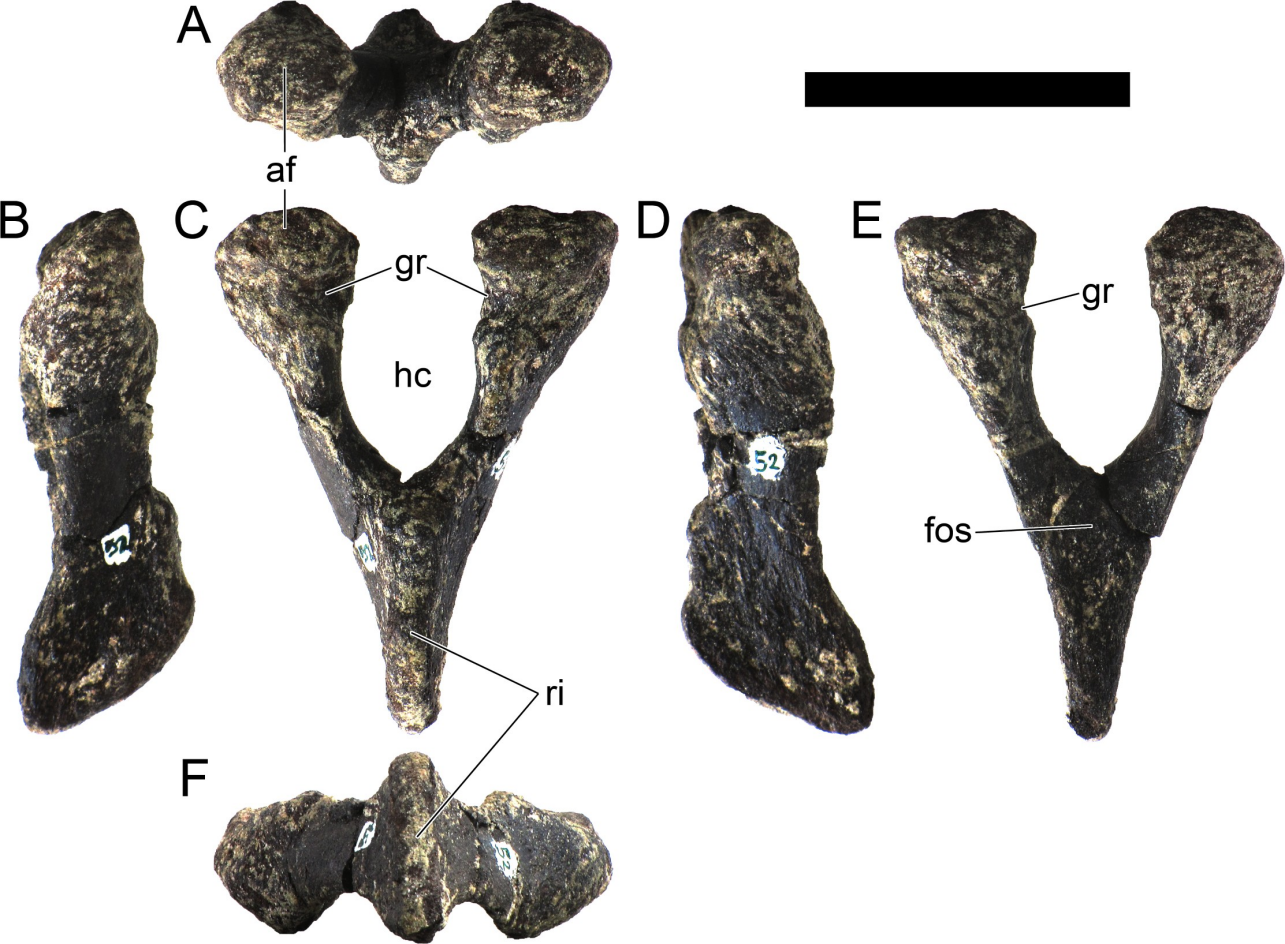
Chevron 17 of *Miragaia longicollum* MG 4863. **A**, dorsal, **B**, right lateral, **C**, anterior, **D**, left lateral, **E**, posterior, and **F**, ventral view. **Af**, articular facet, **fos**, fossa, **gr**, groove, **hc**, haemal canal, **ri**, ridge. Scale bar equal to 5 cm.

Only one complete chevron was found (MG 4863–52; Fig 50), associated with Cd24, Cd27 and Cd34, but, by direct comparison with chevron facets of the caudal vertebrae, it probably articulated posteriorly with Cd17. It is Y-shaped in axial view, with expanded articular surfaces, but not united by a medial bridge enclosing the haemal canal as in other stegosaurian chevrons [19,33]. The proximal articular surfaces are widely spaced and the chevron is short in length, giving it a stout appearance (Fig 50A and 50C). The haemal canal is elliptical in shape and relatively wide and deep, as wide as each articular facet and half the total depth of the chevron (Fig 50C and 50E). Each articular facet is wider than long in proximal view (Fig 50A). The distal half of the chevron (the chevron blade) is keel-shaped, transversely thin and expanded posteriorly (but not as much as in *Kentrosaurus*; [1]). The posterior side of the chevron blade has a fossa ventrally to the haemal canal (Fig 50E) and is wider than the anterior side. The distal surface is an axial ridge that faces antero-ventrally (Fig 50C and 50F). A pair of secondary axial grooves, facing antero-medially, is present just ventrally to the articular facets (Fig 50C). Another chevron blade (MG 4863–51; Fig 28D), much longer axially and shorter dorsoventrally, was found isolated and clean of matrix, probably belonging to the holotype of *D*. *zbyszewskii*, but a clear assignment is inconclusive currently. The haemal canal in all chevrons of this animal would be open, since all caudal vertebrae present chevron facets that are always separated transversely in a distinct pair.

#### Appendicular skeleton

**Fig 51:**
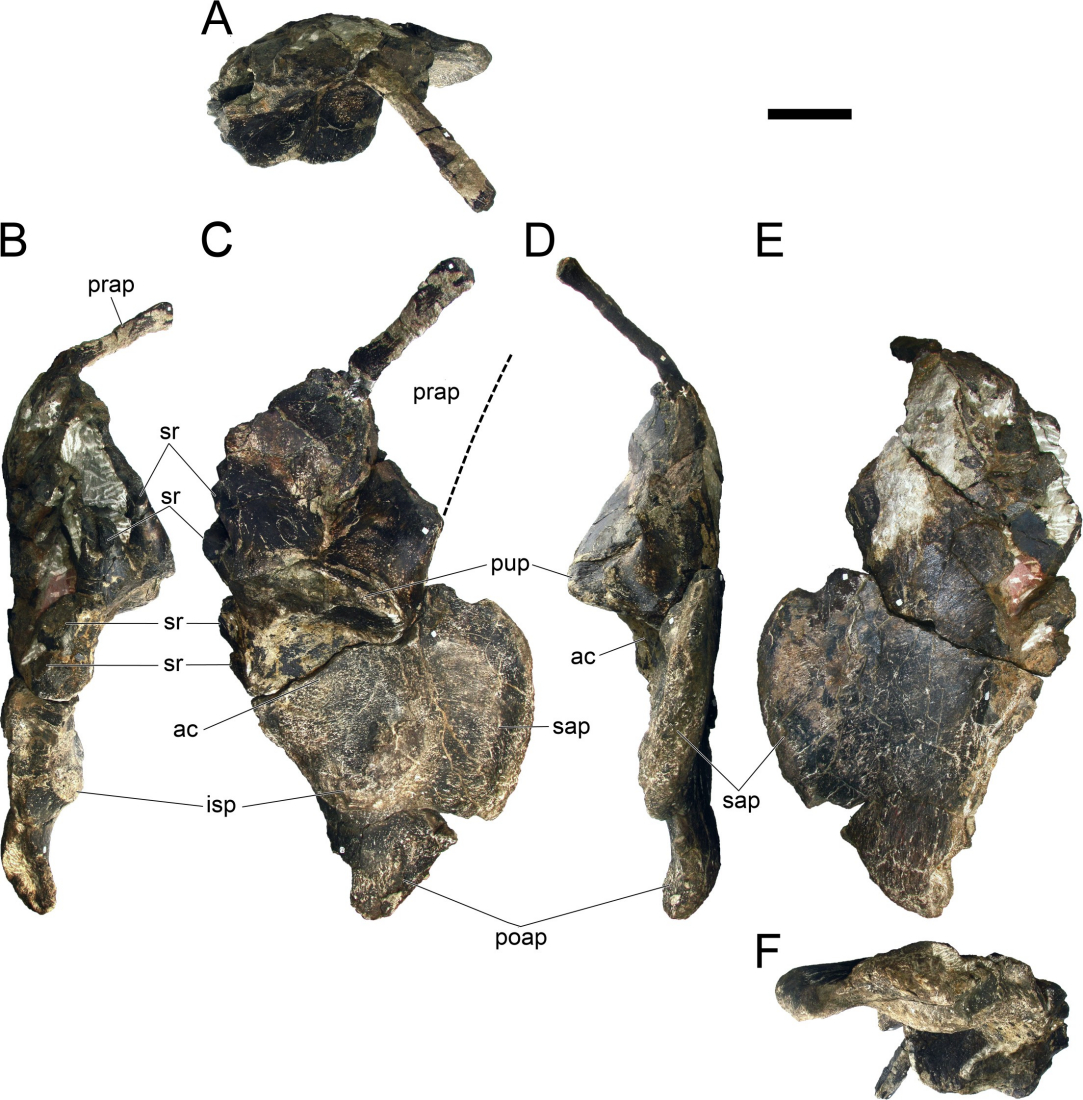
Left ilium and sacral ribs of *Miragaia longicollum* MG 4863. A, anterior, B, medial, C, ventral, D, lateral, E, dorsal, and F, posterior view. Ac, acetabulum, isp, ischial peduncle, poap, postacetabular process, prap, preacetabular process, pup, pubic peduncle, sap, supracetabular process, sr, sacral rib. Scale bar equal to 10 cm.

Left ilium and sacral ribs: The left ilium (MG 4863–59; Fig 51) is almost complete (only a proximal section of the medial margin remains of the preacetabular process). The posteromedial margin of the ilium appears to have been slightly eroded. The preacetabular process broke off at its base (Fig 51D), but the matrix adjacent to its ventral face retains the mold of the bone, so it is possible to observe that the preacetabular process was wide (Fig 51C), its ventral side was gently concave and faced ventroposteromedially. The lateral margin of the preacetabular process was thicker than the medial margin, which curves slightly medially (Fig 51C). Nor the medial margin or the outline in the matrix evidence conclusively the total length of the preacetabular process, only evidencing that it was longer than the anterior extremity of both. It is also not possible to observe if the preacetabular process tapers or expands distally. In ventral view, the medial margin of the preacetabular process sets at an angle of around 45 degrees with the axial plane. The orientation of the preacetabular process was probably exaggerated by posteriorly oriented compressive forces on the anterior side of the pelvic girdle (as is also suggested by the compressive deformation of the anterior tip of the pubis and the tightly stacked sacral ribs), pointing more ventrally than natural. The pubic peduncle is prominent, thin axially and projects ventrally (Fig 51C and 51D). The ischial peduncle is not expanded, defined by low rugosities and concavities that mirror the articular iliac facet of the left ischium (Fig 51B and 51C). The lateral edge of the acetabulum is marked anterolaterally by a low ridge and posterolaterally by a rugose low elevation (Fig 51C). The supracetabular process is large and round in ventral view, expanding anteriorly and posteriorly beyond, respectively, the anterior and posterior margins of the acetabulum (Fig 51C). In lateral view the supracetabular process curves slightly ventrally in the lateral margin (Fig 51D). The postacetabular process is continuous with the supracetabular process and, like it, is mostly flat dorsally and gently concaves ventrally (Fig 51C and 51D). The postacetabular process is small and subtriangular in outline.

**Fig 52:**
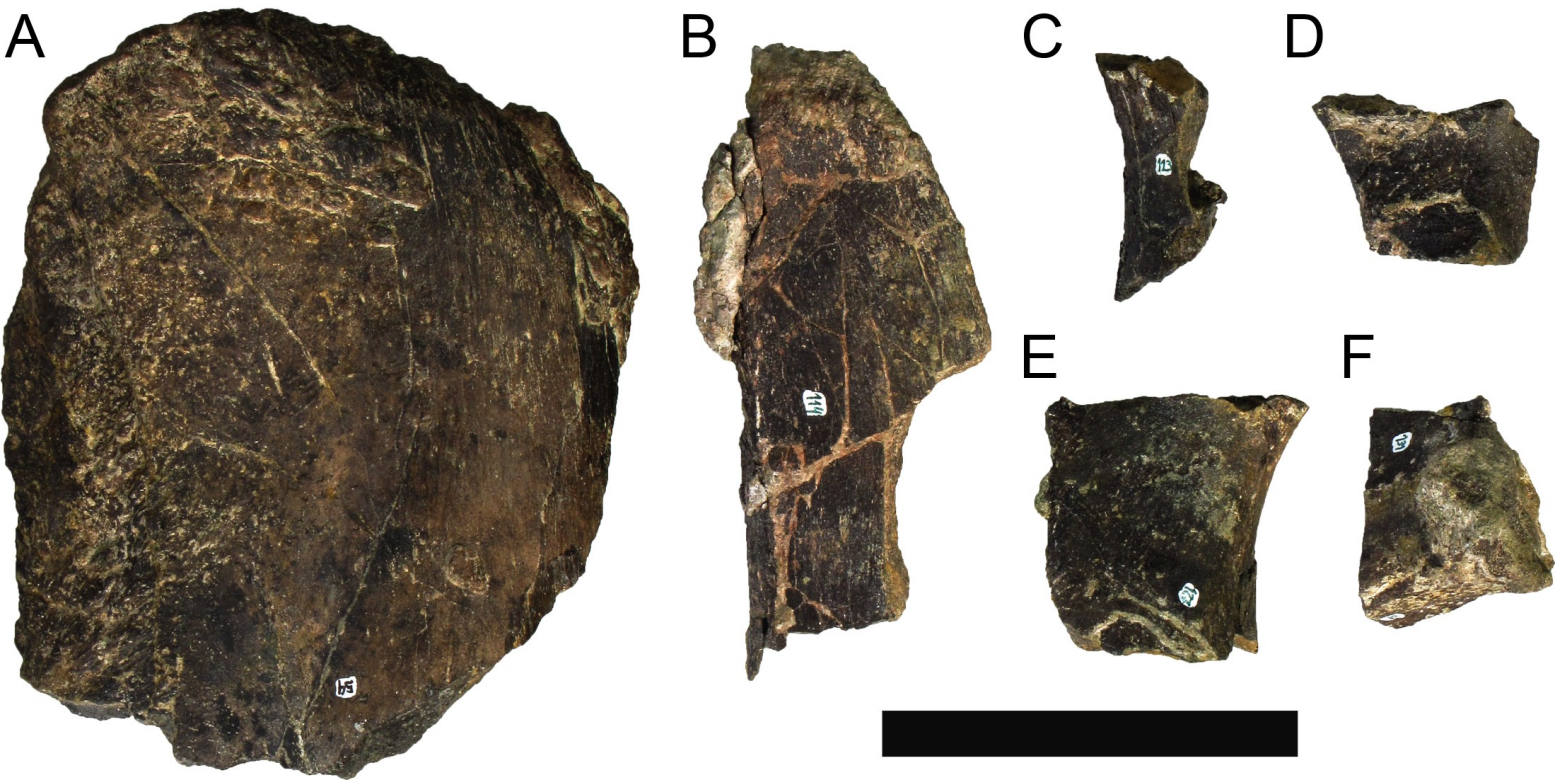
Possible pelvic fragments of *Miragaia longicollum* MG 4863. **A,** MG 4863–54, **B**, MG4863–114, **C**, MG 4863–123, **D**, MG4863–125, **E**, MG 4863–127, **F**, MG4863–131 in lateral or medial view.

A plate-like fragment (MG 4863–54; Fig 52A) may the anterior end of one of the ilia, but its identification is inconclusive. It is relatively thin, with a flat surface on one side and a convex surface on the other, with a round and wide preserved margin. If it is indeed from one of the ilia, it would evidence the preacetabular process has a wide proximal end, not tapering in a sharp point.

The lateral ends of four sacral ribs are preserved (Fig 51B and 51C), they appear to be fused to the medial side of the ilium and are continuous with it dorsally. The sacral ribs are still partially encased in matrix. The first and second sacral ribs are much deeper and narrower than the third and fourth, which appear to be thicker and rounder ventrally. At least the most anterior intervertebral fenestra appears to be closed; the other sacral ribs are partially encased in matrix dorsally, but it appears that the fenestra between them were not closed, so a fully solid dorsal shield was likely not present. The surface of the first sacral rib faces anteroventrally and projects laterally from the ilium. Although slightly eroded, it is unlikely that a fifth sacral rib is present posteriorly. There is no evidence of an additional free rib anteriorly to the first sacral vertebra as in other stegosaurs [1].

**Fig 53:**
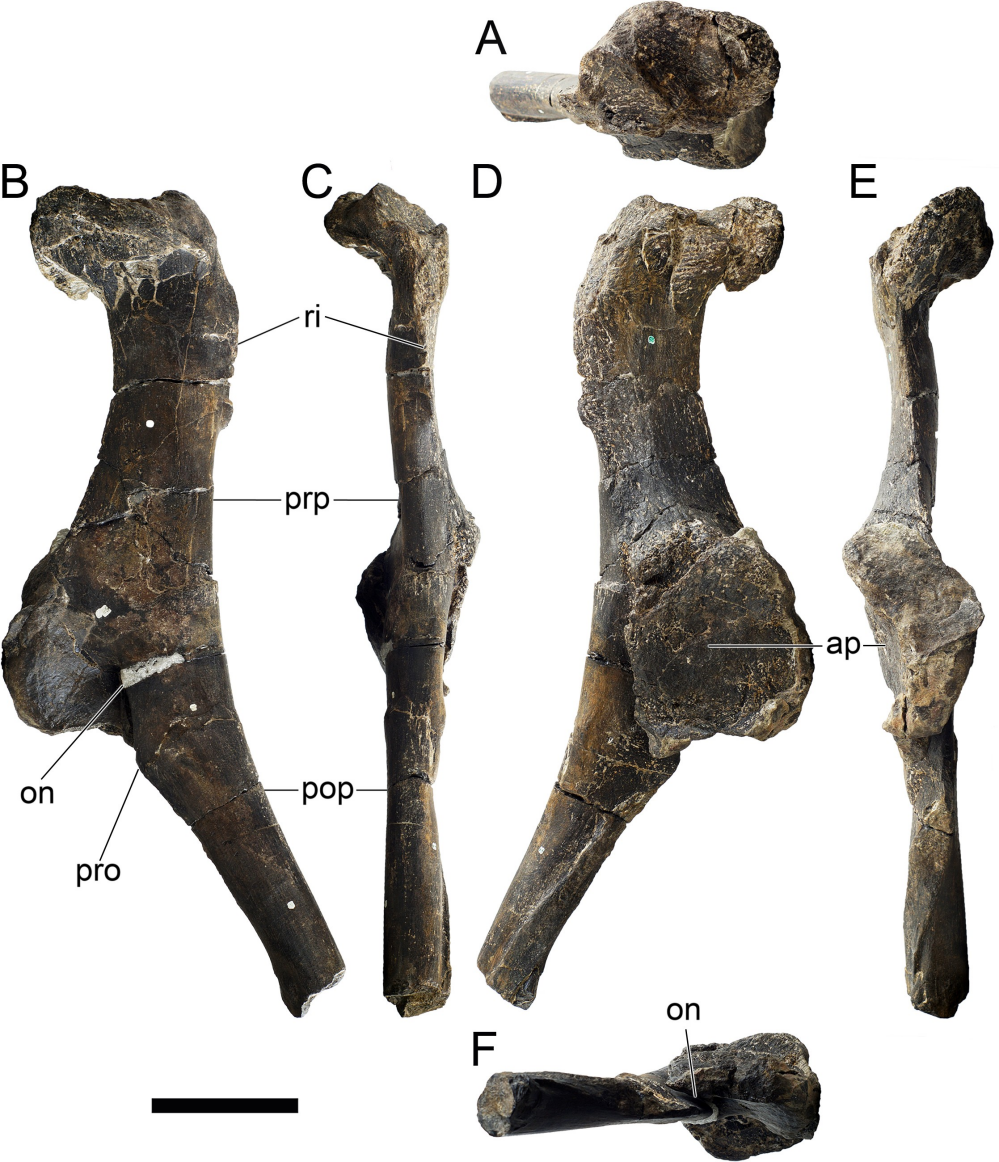
Left pubis of *Miragaia longicollum* MG 4863. **A**, anterior, **B**, medial, **C**, ventral, **D**, lateral, **E**, dorsal, **F**, posterior view. **Ap**, acetabular process, **on**, obturator notch, **pop**, postpubis, **pro**, process, **prp**, prepubis, **ri**, ridge. Scale bar equal to 10 cm.

Left pubis: The left pubis (MG 4863–53; Fig 53) is mostly complete, missing the distal tip of the postpubis and part of the posterior margin of the acetabular process. It was articulated in near life position with the left ilium, as it appears the prepubis has rotated slightly laterally from its original position. The anterior end was severely crushed anteroposteriorly, fracturing in several fragments that were displaced medially, but it is still possible to infer—from these fragments and by how both the ventral and dorsal margins of the prepubis gently curve dorsally—that the anterior end was expanded dorsally but not ventrally (Fig 53B and 53D). The prepubis and proximal postpubis are about equally thick transversely and deep dorsoventrally (Fig 53B and 53C). The prepubis is rugose on the lateral surface and smooth on the medial surface (Fig 53B and 53D). A protruding rugose ridge is present crossing the anterior half of the dorsal margin of the prepubis (Fig 53B and 53C). The proximal section of the postpubis is only marginally shorter dorsoventrally and transversely than the prepubis, while the mid postpubis is thicker transversely and rounder in outline, with a longitudinal ridge laterally (Fig 53B and 53D). The pubis is straight in ventral view (Fig 53C). The acetabular process is large, sub-circular in outline in lateral view (Fig 53D) and thickened dorsally in the articulation surface with the ilium (Fig 53E). The anteroventral margin of the acetabular process merges in a gentle dorsal curve with the prepubis (Fig 53B). The obturator notch is opened posteriorly in posterior view (Fig 53F), but it is backed laterally by the deep postpubis, so in medial view it is closed (Fig 53B)–this observable state has been exhagerated some deformation, as the acetabular process was compressed and displaced in the ventral direction slightly, but it is observable that even if this deformation did not occur, the obturator notch would still be closed in transverse view by overlap. The ventral process just posteriorly to the obturator notch is low and thin transversely (Fig 53B and 53F).

**Fig 54:**
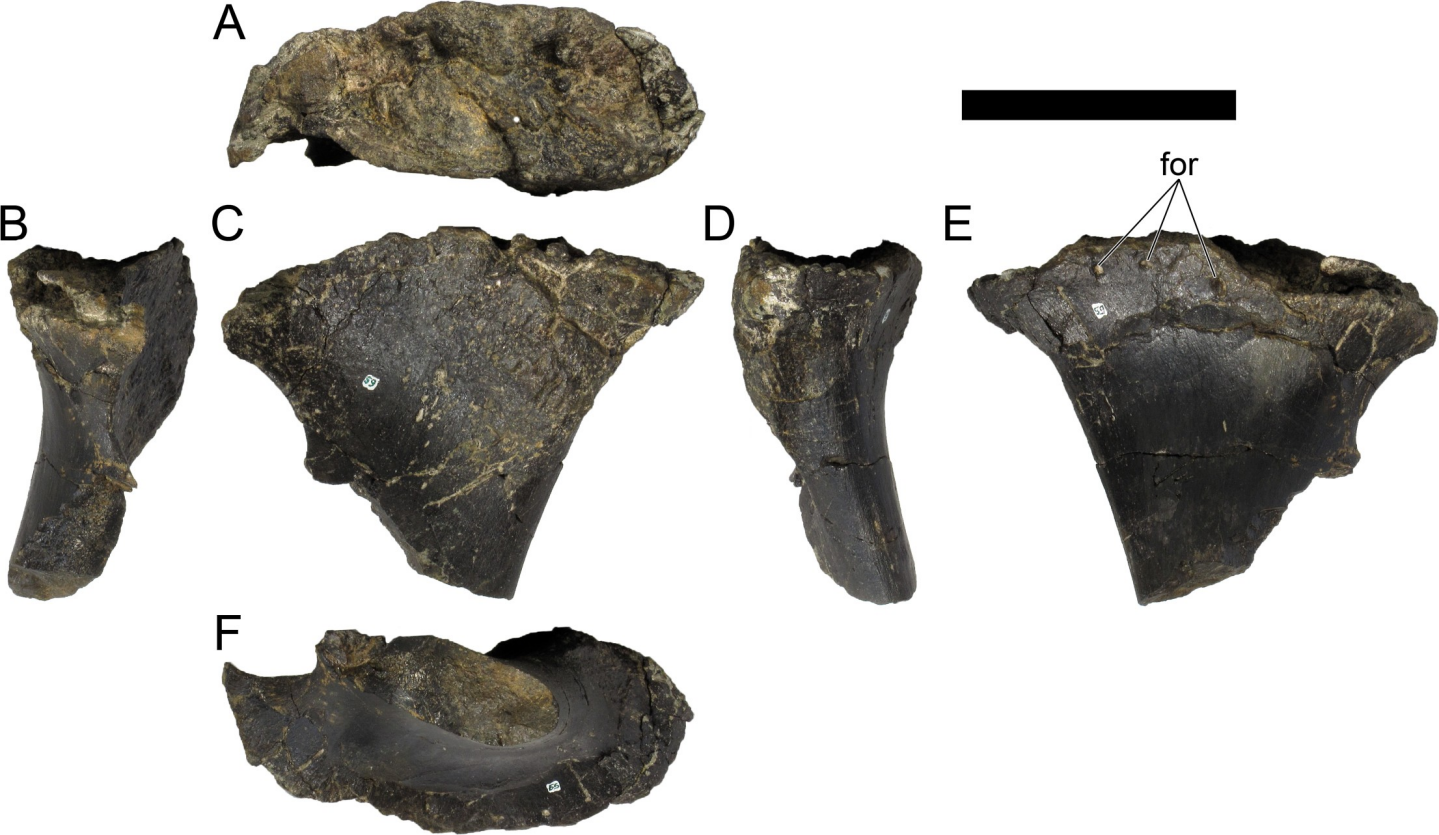
Left ischium of *Miragaia longicollum* MG 4863. **A**, dorsal, **B**, anteromedial, **C**, anterolateral, **D**, posterolateral, **E**, posteromedial, and **F**, ventral view. **For**, foramina. Scale bar equal to 10 cm.

Left ischium: Only the iliac peduncle of the left ischium (MG 4863–65; Fig 54) has been unequivocally identified as part of the ischia of MG 4863. Other fragments (MG 4863–114, -123, -125 and -127; Fig 54B–54E) may be part of this or the right ischium, but are too fragmentary to properly identify or fit with other parts. MG 4863–65 was found isolated (unlike the left pubis, which was articulated with the left ilium). The anterolateral face of the iliac peduncle (i.e., the acetabular side) is concave with marked rugosities, particularly in the dorsal margin (Fig 54C); the posteromedial side is concave, has a smooth surface and evidences some foraminae near the dorsal margin (Fig 54E). The articular facet is lenticular in outline in dorsal view, slightly wider posteriorly and with a sharp curve laterally on the anterior end (Fig 54A). The articular facet has a bulbous and irregular surface, perfectly mirroring the opposing texture of the ischial peduncle in the left ilium, resulting in an exact articulation. When articulated the acetabular side faces anterolaterally and slightly ventrally; passing ventrally from the articular facet, it twists, with the ventral section aligned more anteroposteriorly (Fig 54F). The anteromedial margin of the iliac peduncle (i.e., the acetabular margin) curves sharply in a round and short concavity in lateral view (Fig 54C and 54E), while the posterior margin also curves but more gently (Fig 54C and 54E).

MG 4863–114 may be the posterior end of one of the ischia (Fig 52B). It is a flat and narrow straight blade with some slight expansion in the distal margin. It was found against the proximal articular facet of the left tibia. If it is indeed one of the ischial posterior ends, it does not evidence rugosities in any of the sides that would mark the presence of the ischial symphysis (where the ischia ends would contact posteromedially). It is also straight in both margins, tapering gently to the distal end. Some other fragments (MG 4863–123, -125, -127, -131; Fig 52C–52F) probably are also be part of one of the ischia or of the pubes, but their identification is inconclusive.

**Fig 55:**
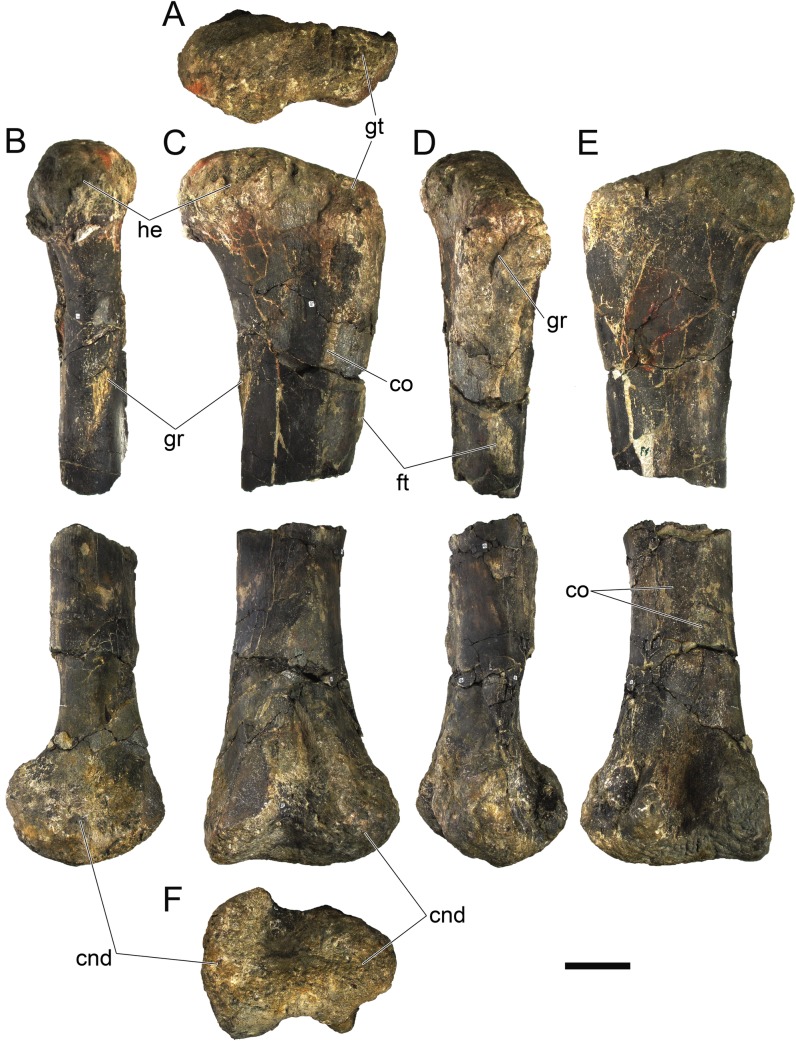
Left femur of *Miragaia longicollum* MG 4863. **A**, dorsal, **B**, medial, **C**, anterior, **D**, lateral, **E**, posterior, and **F**, ventral view. **Cnd**, condyle, **co**, cord, **ft**, fourth trochanter, **gr**, groove, **gt**, greater trochanter, **he**, femur head. Scale bar equal to 10 cm.

**Fig 56:**
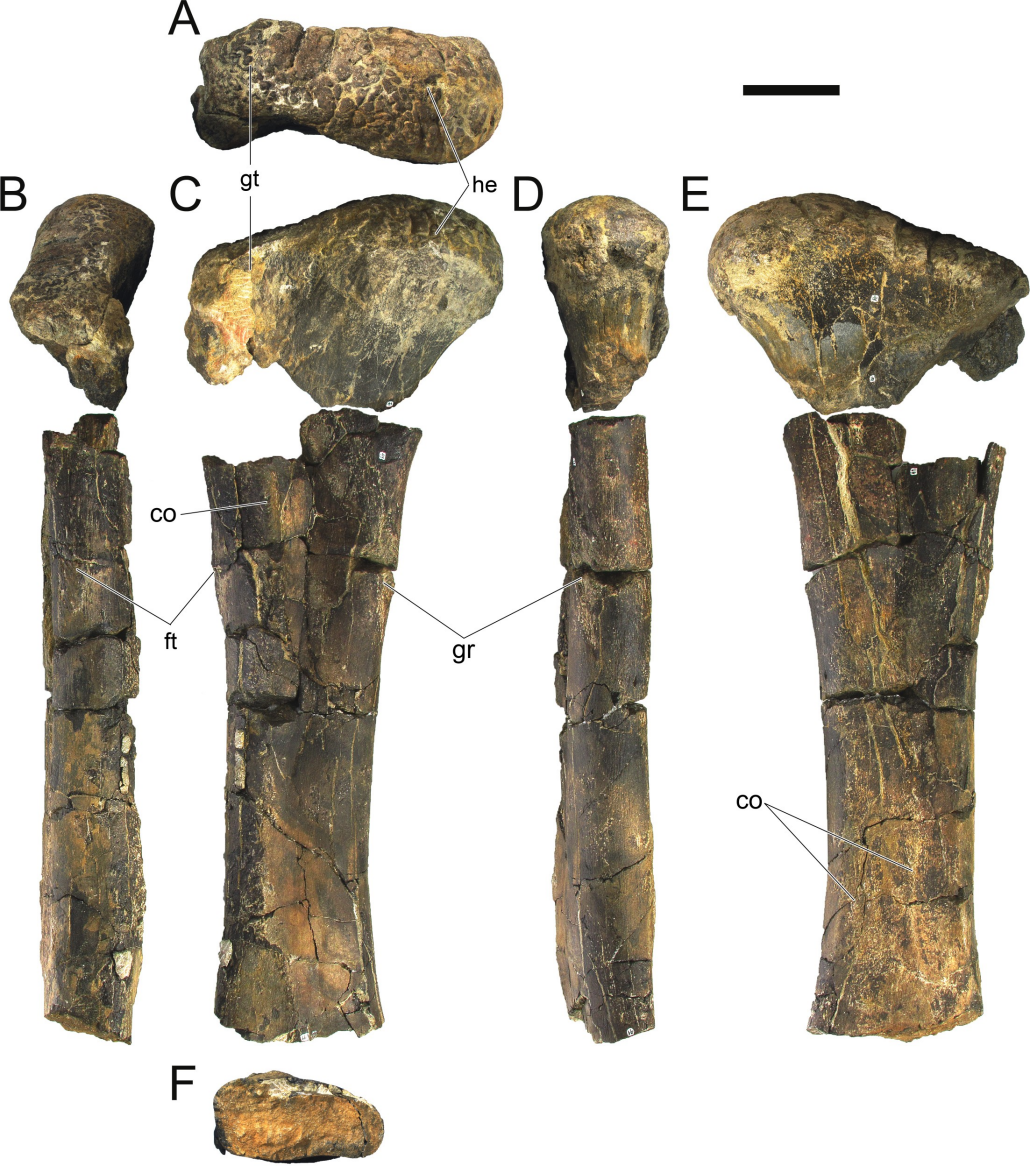
Right femur of *Miragaia longicollum* MG 4863. **A**, dorsal, **B**, lateral, **C**, anterior, **D**, medial, **E**, posterior, and **F**, ventral view. **Co**, cord, **ft**, fourth trochanter, **gr**, groove, **gt**, greater trochanter, **he**, femur head. Scale bar equal to 10 cm.

Femora: Both femora are present but are not complete. The left femur (MG 4863–40; Fig 55) is broken up in a proximal and a distal half, but these do not connect as a mid-section of about one tenth the length of the femur is missing; the right femur (MG 4863–41; Fig 56) is composed of an almost complete shaft (it is missing the distal condyles, but evidences the transverse expansion just proximally to the distal end) and a broken off proximal end (which fits with the top of the shaft, but in a much reduced area). Although neither is complete, they can compose a complete femur, and through measured comparisons between these (and with the *Dacentrurus* sp. femora displayed in MG), a total length of approximately 100 cm for complete femora is probable (see Table 3 for measurements of limb elements). Both femora appear to have suffered anteroposterior crushing, particularly in the proximal portions of the shafts. The lateroproximal end of the right femur was found pressed against the distal condyles of the left femur, which may have deformed both. In most respects, these are similar to the femora of other stegosaur species [1]. The femora are straight and columnar in anterior and lateral views, with transversely and anteroposteriorly expanded ends and a shaft somewhat compressed anteroposteriorly. The femoral head is not distinct from the shaft, evidencing a smooth transition (except in the posterior side of the left femur head, which evidences a constriction probably by deformation; Fig 55C, 55E, 56C and 56E). The femoral heads are convex and round and project medially and slightly proximally, with the dorsal margin sloping in a flat surface to the dorsal margin of the greater trochanter, setting the dorsal margin of the femora at an angle of about 110 degrees with the lateral margin of the femora in axial view (Fig 55C, 55E, 56C and 56E). A smooth and round curve is present, in axial view, between the ventral margin of the femoral heads and the medial margin of the shafts (Fig 55C, 55E, 56C and 56E). In proximal view the femora have a sub-dumbbell outline (but the femoral head and greater trochanter are barely distinguishable), wide transversely and longer anterodorsally in the femoral head (Fig 55A and 56A). The proximal articular surfaces have a markedly bulbous texture (Fig 55A). The lesser trochanter is fused to the adjacent surface of the greater trochanter, not distinctly protruding in the left femur, but a variety of low ridges and marked rugosities in the proximoanterolateral area and a lateral groove mark its position (Fig 55C and 55D). A shallow groove is present proximally at the lateral side of the left femur, extending longitudinally (Fig 55D). The shaft of the left femur has a thick sub-elliptical to sub-dumbbell cross section, while the right femur has a much more rectangular outline (with rounded edges). The fourth trochanters are distinguishable as low rugose ridges positioned laterally about one third the length of the femur passing distally, but do not form a distinct process and are hardly visible in anterior view (Figs 55C, 55D, 56B and 56C). A rugose groove is positioned anteromedially in both femora (approximately opposed to the fourth trochanters and about the same size), extending longitudinally but with the distal end inclined slightly medially (Figs 55B, 55C, 56B and 56D). A longitudinal cord-like ridge extends over the lateral half of the anterior face of the femoral shaft of each femur, bifurcating and merging with the shaft close to the distal end (Figs 55C and 56C). These are most distinct proximally, evidencing a texture of chevron-shaped rugosities with the apex pointing distally. A pair of similar parallel ridges is observable over the posterior surface of each femoral shaft, but is not as developed (Figs 55E and 56E). Shallow concavities in the anterior surface are present distally and proximally, but these may have been exaggerated (or shaped) by the compressive deforming forces. A deep semi-circle shaped intercondylar groove separates the condyles and extends dorsally in a shallow concavity (Fig 55C and 55F). In distal view, the left femur has a semi-dumbbell outline, longer anteroposteriorly and narrower transversely by the medial condyle and shorter and wider by the lateral condyle (Fig 55F). In posterior view, the medial condyle is about twice as wide than the lateral condyle, both have an elliptical outline and the former distinctly projects from the rest of the femur (Figs 55A and 56B). In medial view, the medial condyle is distinctly round in outline and expanded about twice as long anteroposteriorly than the femoral shaft (Fig 55B).

**Table 3:** Limb elements measurements. *, estimated measurement. Values represent the maximum measurement. Values in mm.

	Length	Proximal width	Distal width	Midshaft width
Femur, left	1000*	290	250	165
Femur, right	1000*	290	-	160
Tibia, left	57	28	23	12
Tibia, right	-	18	23	11
Fibula, left	53*	10	10	5
Fibula, right	-	-	-	5
Metatarsal 2, left	-	-	67	42
Metatarsal 3, left	117	(68)	74	44
Metatarsal 4, left	124	83	79	45
Metatarsal 3, right	-	-	76	43
Metacarpal 1	100	63*	72	51
Metacarpal 2	113*	66	57*	38
Metacarpal 3	93*	87*	78	49

**Fig 57:**
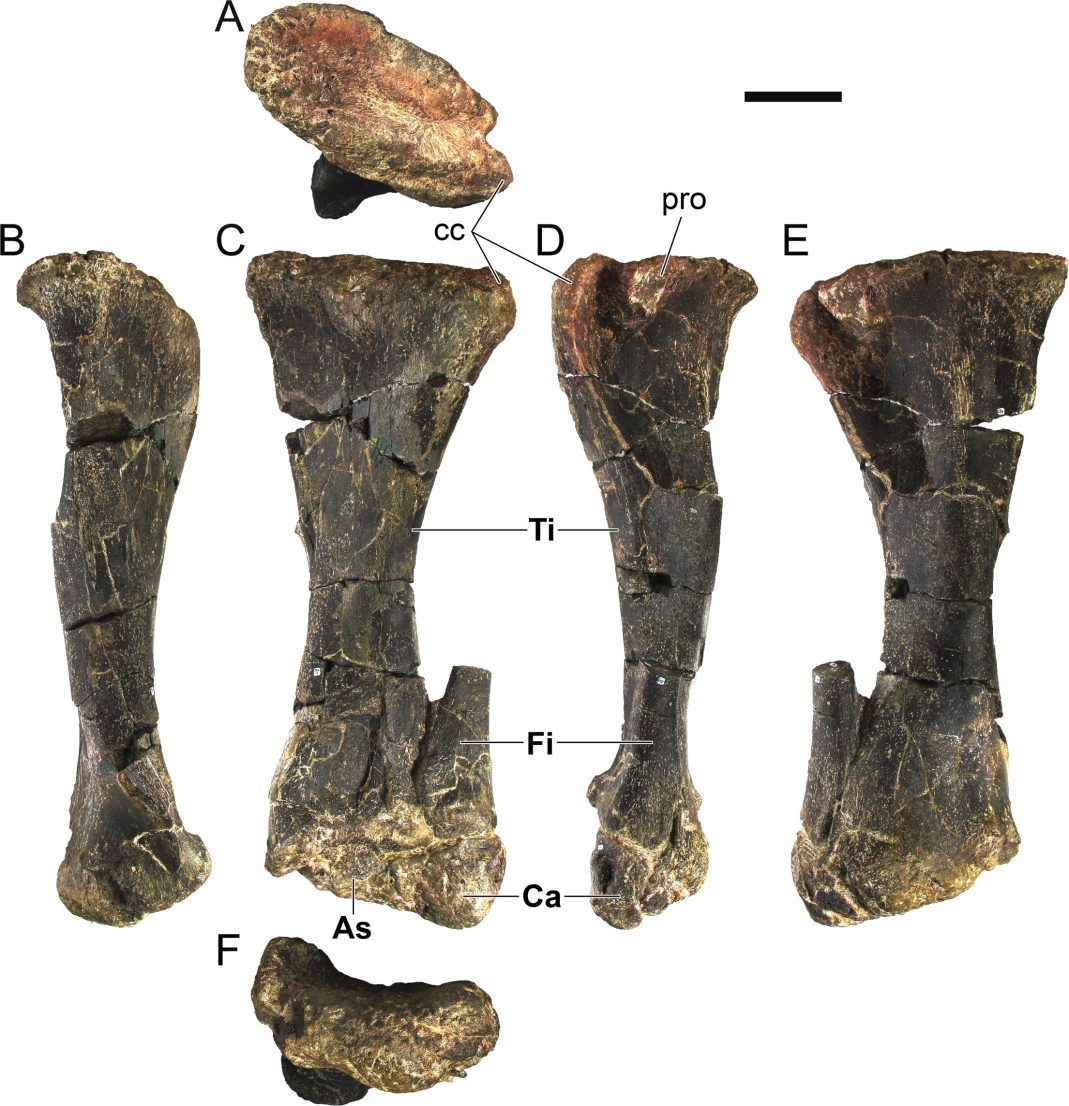
Left tibia, tarsus and distal fibula of *Miragaia longicollum* MG 4863. **A**, dorsal, **B**, medial, **C**, anterior, **D**, lateral, **E**, posterior, and **F**, ventral view. **As**, astragalus, **Ca**, calcaneum, **cc**, cnemial crest, **Fi**, distal fibula, **pro**, process. Scale bar equal to 10 cm.

**Fig 58:**
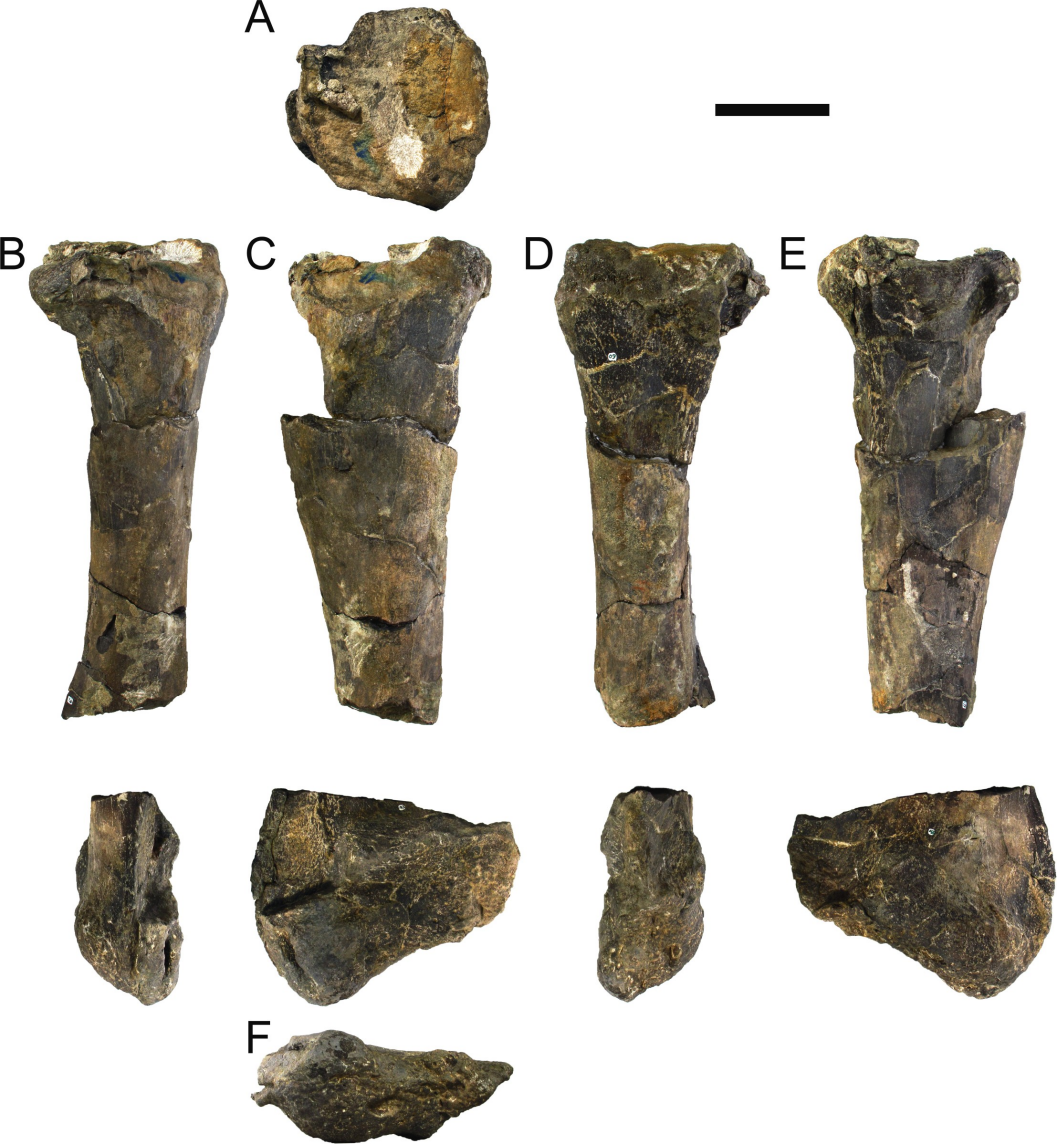
Right tibia and tarsus of *Miragaia longicollum* MG 4863. **A**, dorsal, **B**, lateral, **C**, anterior, **D**, medial, **E**, posterior, and **F**, ventral view. Scale bar equal to 10 cm.

Tibiae and tarsus: The left tibia, left astragalus and left calcaneum (MG 4863–42; Fig 57) are complete and fused. The right tibia, right astragalus and right calcaneum (MG 4863–43; Fig 58) are also fused, but the distal end is broken off from the rest of the proximal two thirds of the tibia, which is severely deformed. Both tibiae appear partially fused distally with the respective fibula, and the proximal left fibula articulated with the left tibia.

The left tibia evidences expanded ends with the longer transverse axes set at an angle of about 45 degrees (Fig 57A). This is probably due more to rotative deformation than anatomical adaptation, as the non-aligning medial broken ends of the left fibula seem to suggest, and in other stegosaurs the widest diameters of the expanded ends of the tibiae occupy the same plane [33]. In lateral view the anterior surface evidences a sigmoid curve in outline (convex in the anterior half and concave in the distal half) while the posterior side evidences more of a low “W” shaped outline (concave in the distal and proximal ends and convex in the middle; Fig 57B and 57D). The proximal articular surface of the left tibia is concave and elliptical in proximal view (Fig 57A). The cnemial crest projects laterally and is thin anteroposteriorly (but this orientation was most likely deformed to point in this direction instead of more anteriorly as in other stegosaurs [33]; Fig 57A, 57C and 57D). Posteriorly to the cnemial crest there is a low process where the fibula attached, and in proximal view a marked notch is present between these (Fig 57A, 57D and 57E). The shaft of the tibia is more slender than the expanded ends, sub-sound in outline at mid-length (Fig 57C and 57E). The distal end is convex posteriorly and flat to gently convex anteriorly (Fig 57C and 57E) The proximal two-thirds of the right tibia, since extensively crushed and deformed (to a degree that it does not fit with the distal end) offers no further useful anatomical details than the left tibia. The left tibia is much shorter than the femora, with the femora 159% the length of the tibia (see Table 3 for measurements of limb elements).

Both astragali and calcanea are fused to the respective tibial distal ends, with almost indistinct sutures (Fig 57C and 57D). The tarsals are widely different between one side and the other of the animal, but it is possible to observe that the astragali are bigger than the calcanea. It is not possible to observe if the distal end of the tibia is hollowed out to receive the large proximal projection of the astragalus since these are fused in both cases.

**Fig 59:**
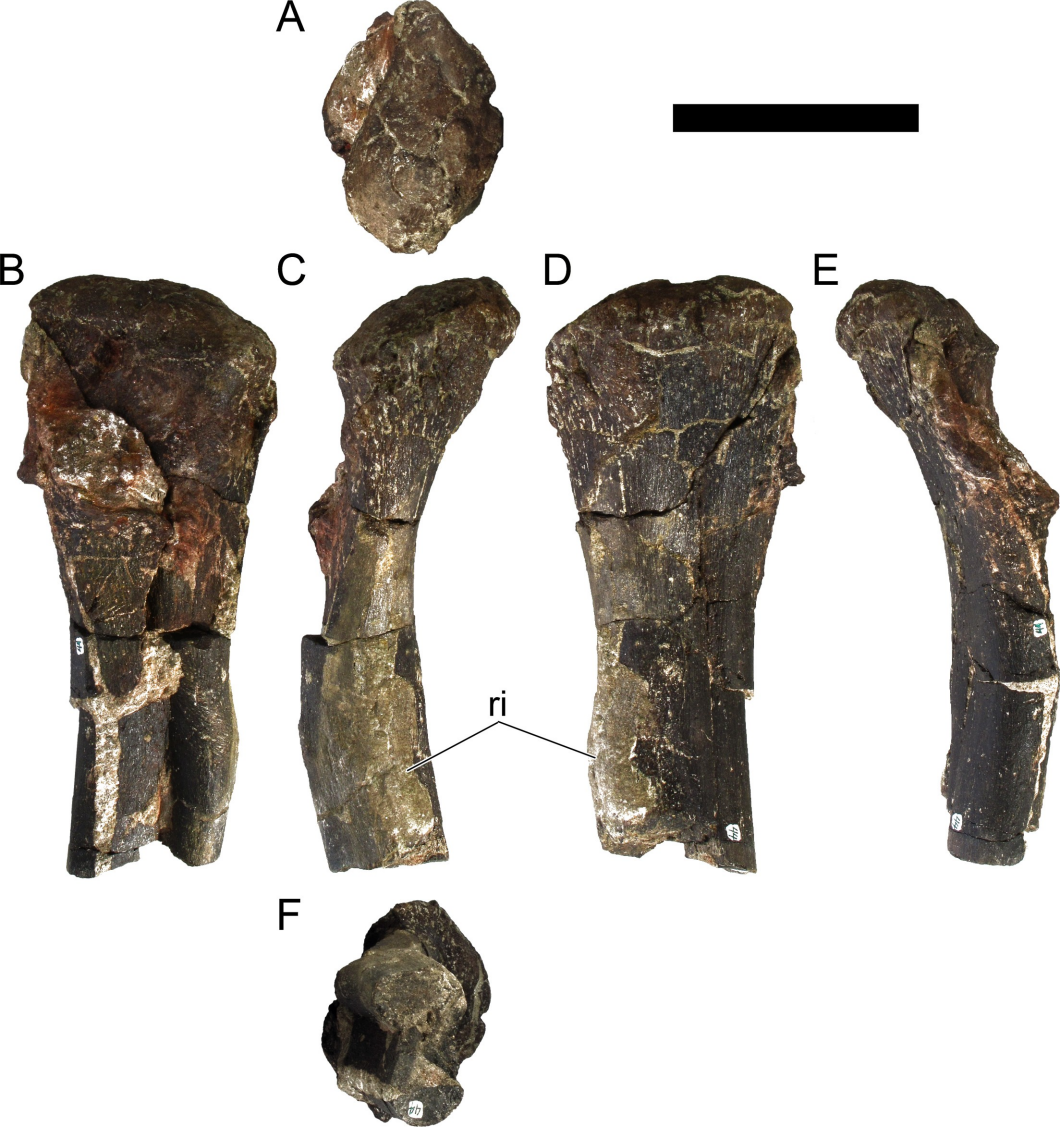
Left proximal fibula of *Miragaia longicollum* MG 4863. **A**, dorsal, **B**, medial, **C**, anterior, **D**, lateral, **E**, posterior, and **F**, ventral view. **Ri**, ridge. Scale bar equal to 10 cm.

**Fig 60:**
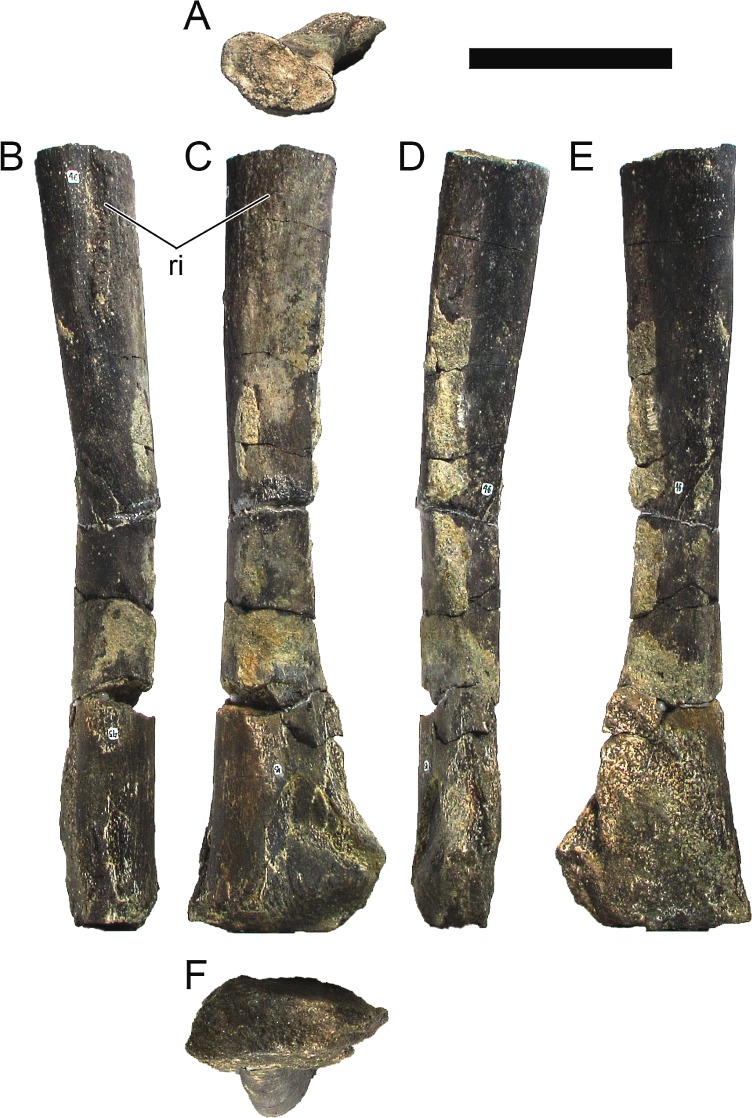
Right fibula of *Miragaia longicollum* MG 4863. **A**, dorsal, **B**, lateral, **C**, anterior, **D**, medial, **E**, posterior, and **F**, ventral view. **Ri**, ridge. Scale bar equal to 10 cm.

Fibulae: The left fibula (MG 4863–44; Fig 57 and Fig 59) is missing its mid-section while the right fibula (MG 4863–45; Fig 60) is missing its proximal end. Both fibulae are fused distally to the respective tibia (but the right fibula has broken off distally from the right tibia) and the left fibula was articulated proximally to the lateral side of the left tibia.

The fibulae are slender and slightly expanded at both ends. The fibulae are rod-like and straight (Fig 60B and 60C), except at the proximal end that curves gently laterally (Fig 59C and 59E). The proximal end is compressed transversely and the distal end is compressed anteroposteriorly (Figs 59A–59C, 60B, 60C and 60F). The distal end of the right fibula is gently concave or flatter in the articulating surface with the tibia (Fig 60E and 60F). A thick obtuse rugose ridge passes diagonally across the medial surface, just distally to the proximal articulation (Figs 59C, 59D, 60B and 60C). In the preserved articulation of the left tibia and fibula, the proximal margin of the fibula projects proximally beyond the proximal margin of the fibula. The fibulae are contiguous and contact ventrally the dorsal side of the calcanea (Fig 57C).

Metatarsals: The three left metatarsals and the third right metatarsal were found, all isolated and not articulating. The metatarsals are short, with expanded ends and stout shafts.

**Fig 61:**
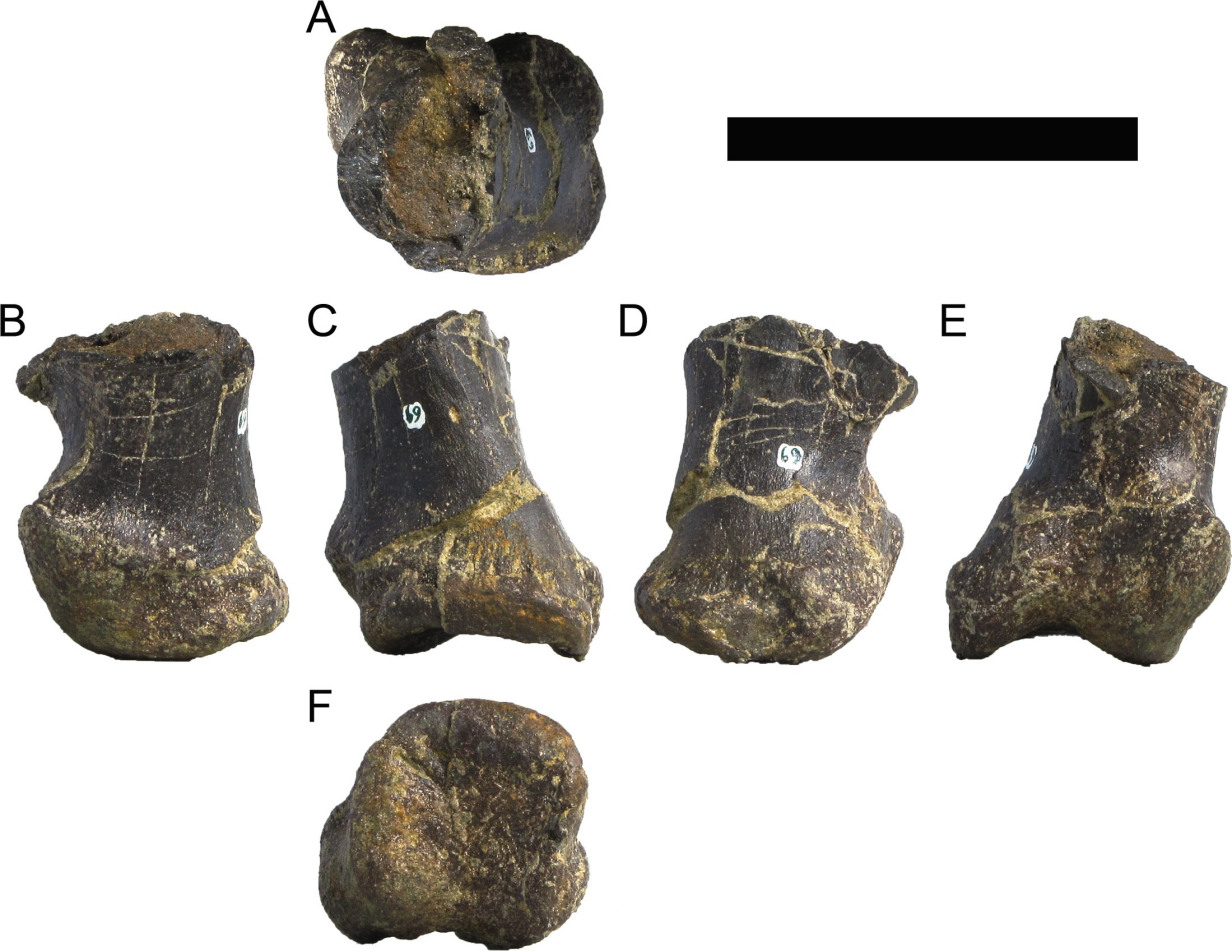
Left metatarsal two of *Miragaia longicollum* MG 4863. **A**, dorsal, **B**, medial, **C**, anterior, **D**, lateral, **E**, posterior, and **F**, ventral view. Scale bar equal to 10 cm.

Only the distal half of the second left metatarsal (MG 4863–69; Fig 61) was found. The shaft is deformed, curving laterally (Fig 61C and 61E). The shaft is about as thick as the shafts of the third and fourth metatarsals. The distal articulation is the narrowest transversely among the metatarsals, about as long anteroposteriorly as it is wide with a sub-square outline (Fig 61F). In distal view, the distal articulation is concave distally and posterodistally and has a short and wide extension posterolaterally (Fig 61F).

**Fig 62:**
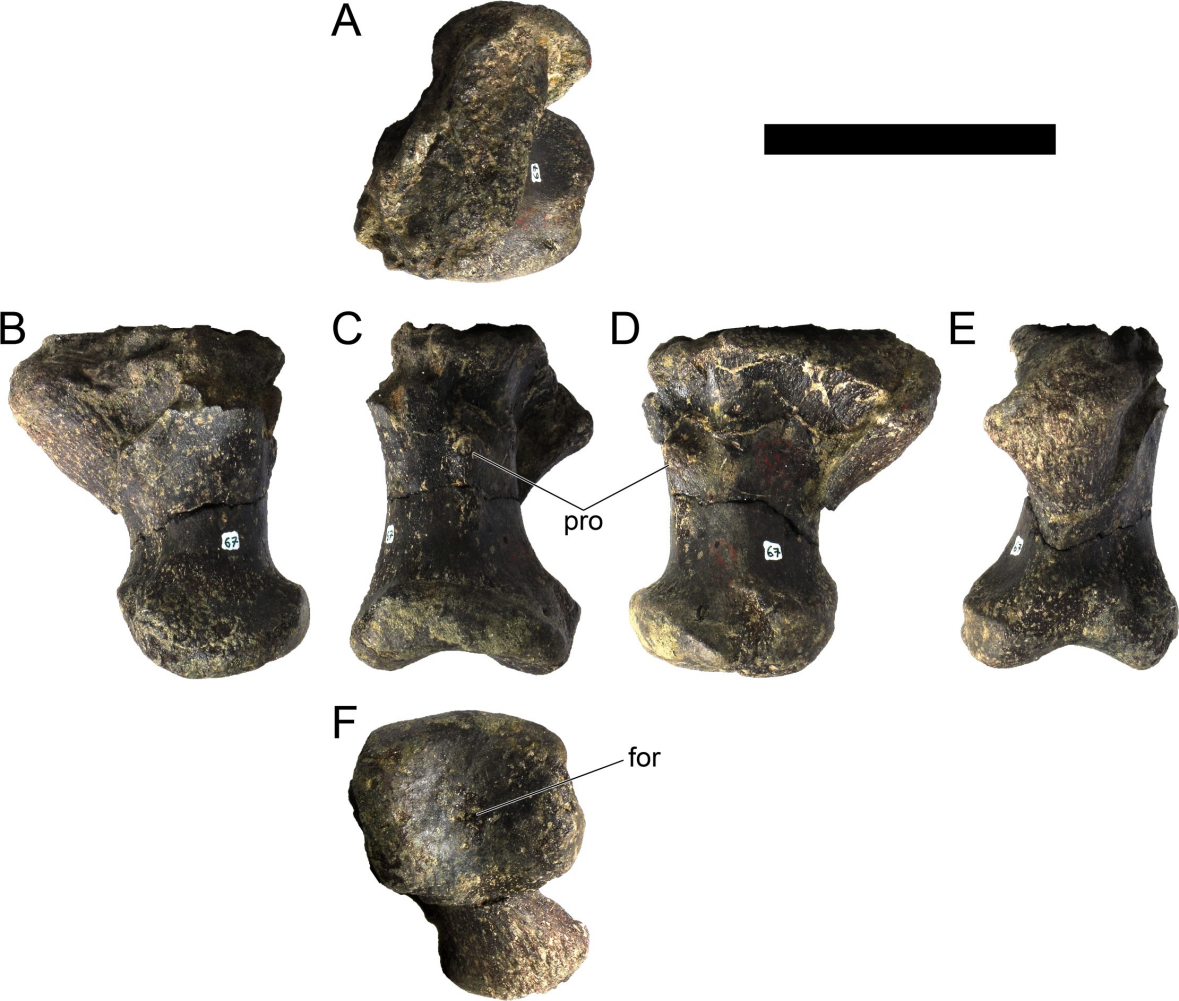
Left metatarsal three of *Miragaia longicollum* MG 4863. **A**, dorsal, **B**, medial, **C**, anterior, **D**, lateral, **E**, posterior, and **F**, ventral view. **For**, foramen, **pro**, process. Scale bar equal to 10 cm.

The third left metatarsal (MG 4863–67; Fig 62) is complete, but its proximal articulation was crushed transversely. Although likely exaggerated by the deformation, it is possible to observe that the proximal articulation was longer anteroposteriorly than transversely (Fig 62A). The posterior margin curves sharply posteriorly in transverse view, while the anterior margin is mostly straight and vertical (Fig 62B and 62D). A process is present approximately in the center of the anterior face (slightly more proximolaterally positioned than the center), projecting dorsally (Fig 62C and 62D). The distal articulation is wider than long anteroposteriorly and has a larger area than the distal articulations of the 2nd and 3rd metatarsals (Fig 62F). The distal articulation is concave distally and posterodistally (Fig 62E and 62F). In the center of the distal articulation there is a small foramen (Fig 62F).

**Fig 63:**
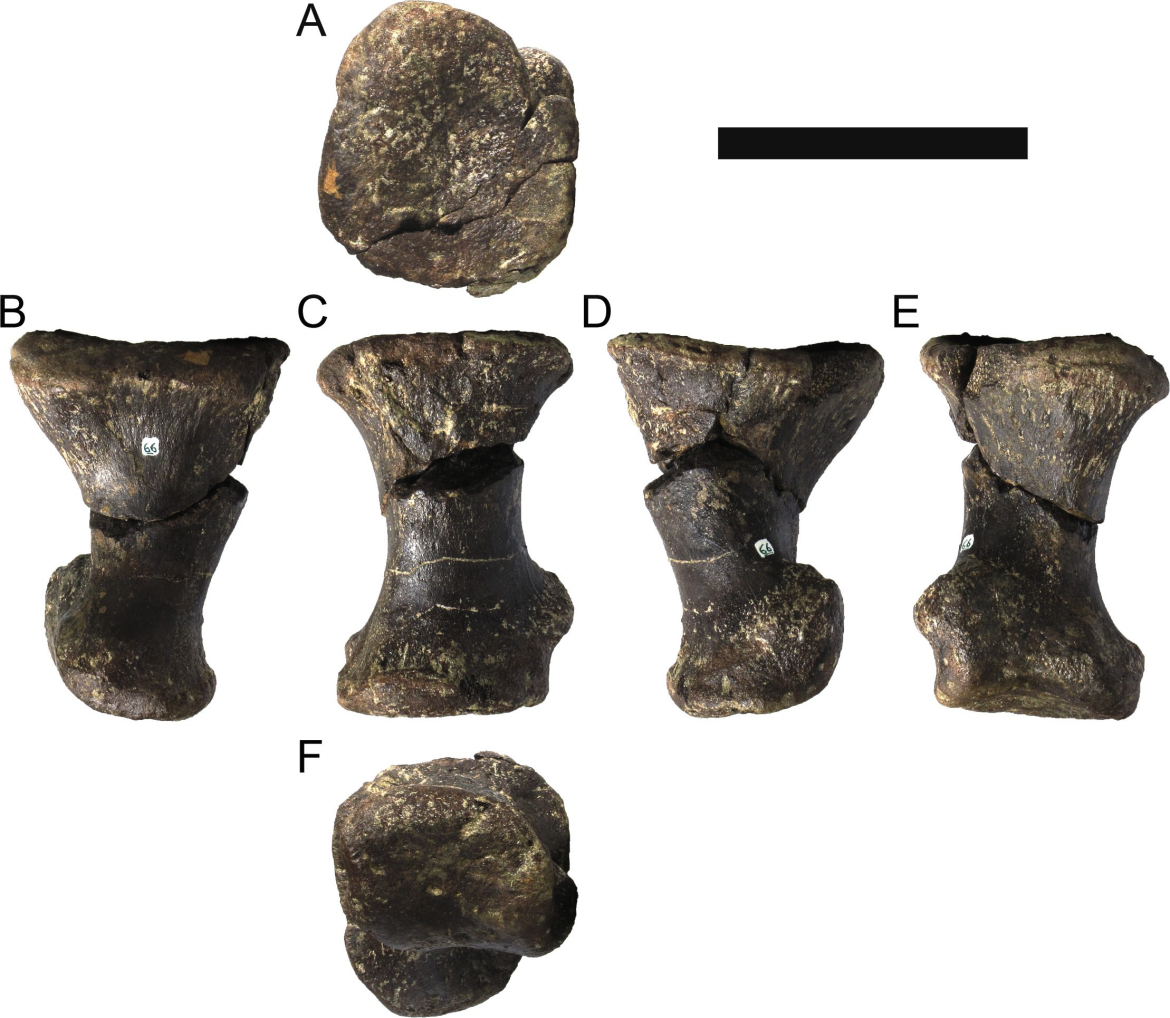
Left metatarsal four of *Miragaia longicollum* MG 4863. **A**, dorsal, **B**, medial, **C**, anterior, **D**, lateral, **E**, posterior, and **F**, ventral view. Scale bar equal to 10 cm.

The fourth left metatarsal (MG 4863–66; Fig 63) is the largest metatarsal. It has a large expanded proximal end, almost as wide as long anteroposteriorly, sub-round in outline and gently concave (Fig 63 and 63D). The shaft is thick and the posterior margin is concave in outline in transverse view (Fig 63B and 63D). The proximal articulation is about double the length anteroposteriorly of the distal articulation in transverse view (Fig 63B and 63D). The fourth metatarsal is slightly longer longitudinally than the 3rd metatarsal. The distal articulation is wider transversely and shorter anteroposteriorly than the distal articulations of the 2nd and 3rd metatarsals and has a prominent projection posterolaterally in distal view (Fig 63F).

**Fig 64:**
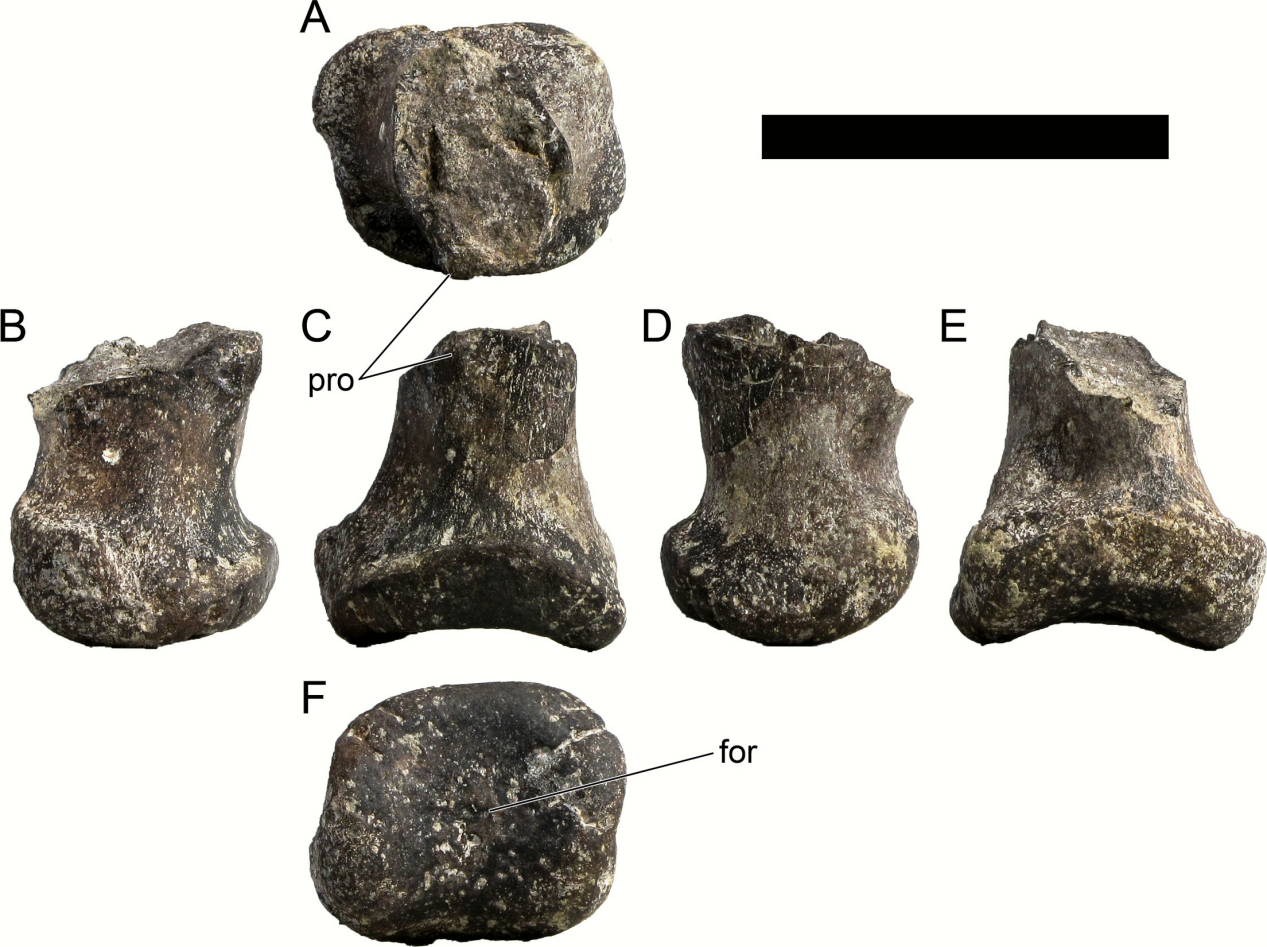
Right metatarsal three of *Miragaia longicollum* MG 4863. **A**, dorsal, **B**, lateral, **C**, anterior, **D**, medial, **E**, posterior, and **F**, ventral view. **For**, foramen, **pro**, process. Scale bar equal to 10 cm.

Only the distal half of the third right metatarsal (MG 4863–68; Fig 64) was found. It is virtually identical to the distal half of the 3rd left metatarsal, but mirrored, including a similar foramen in the center of the distal articular facet (Fig 64F) and a symmetrical process in the center of the anterior face (Fig 64A and 64C).

**Fig 65:**
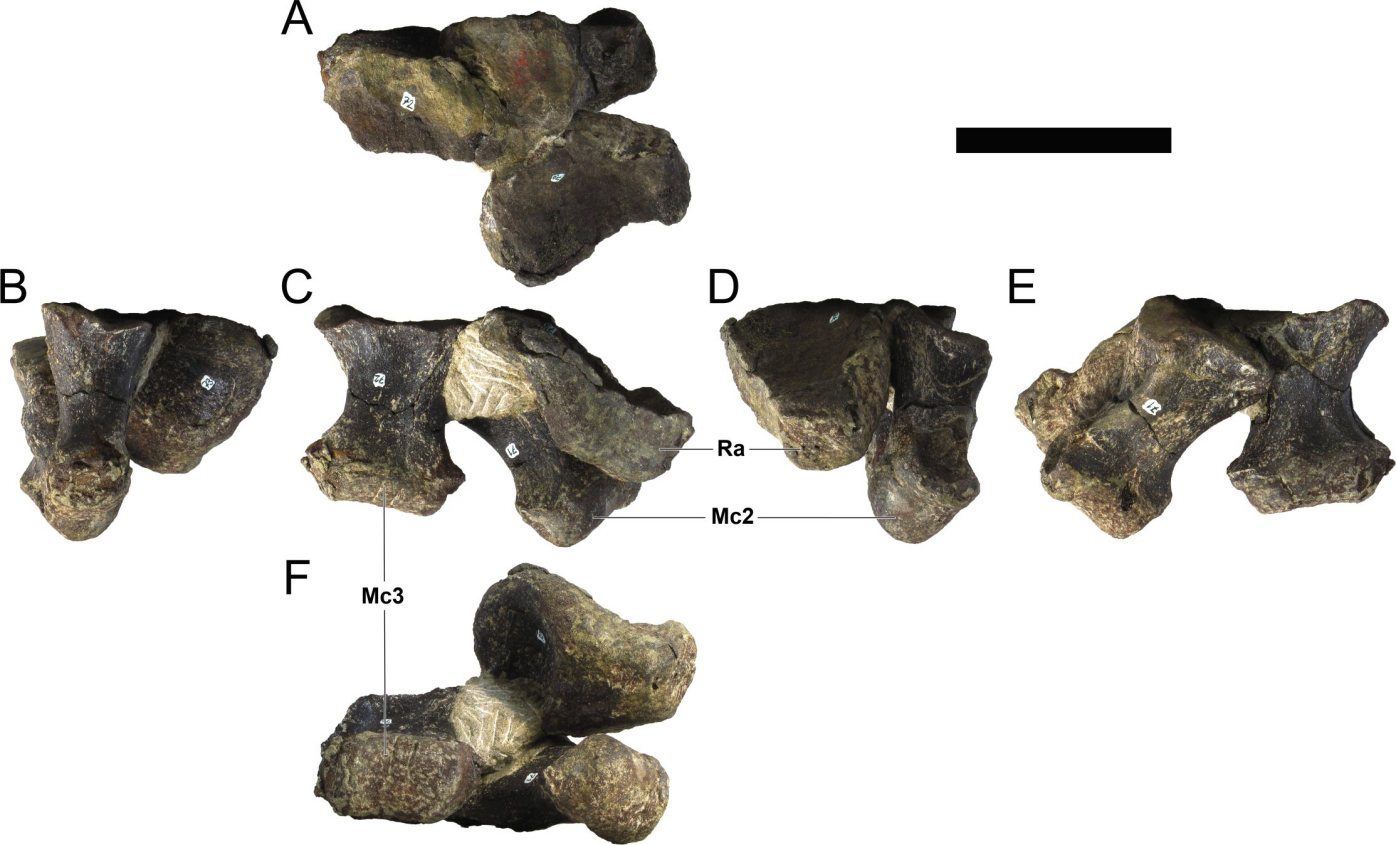
Left metacarpals two, three, and radiale of *Miragaia longicollum* MG 4863. **Mc2**, second left metacarpal in **A**, dorsolateral, **B**, dorsomedial, **C**, anterior, **D**, ventrolateral, **E**, posterior, and **F**, ventromedial view. **Mc3**, third left metacarpal in **A**, dorsal, **B**, medial, **C**, anterior, **D**, lateral, **E**, posterior, and **F**, ventral view. **Ra**, left radiale in **A**, dorsal, **B**, anterior, **C,** medial, **D**, posterior, **E**, lateral, and **F**, ventral view. Scale bar equal to 10 cm.

**Fig 66:**
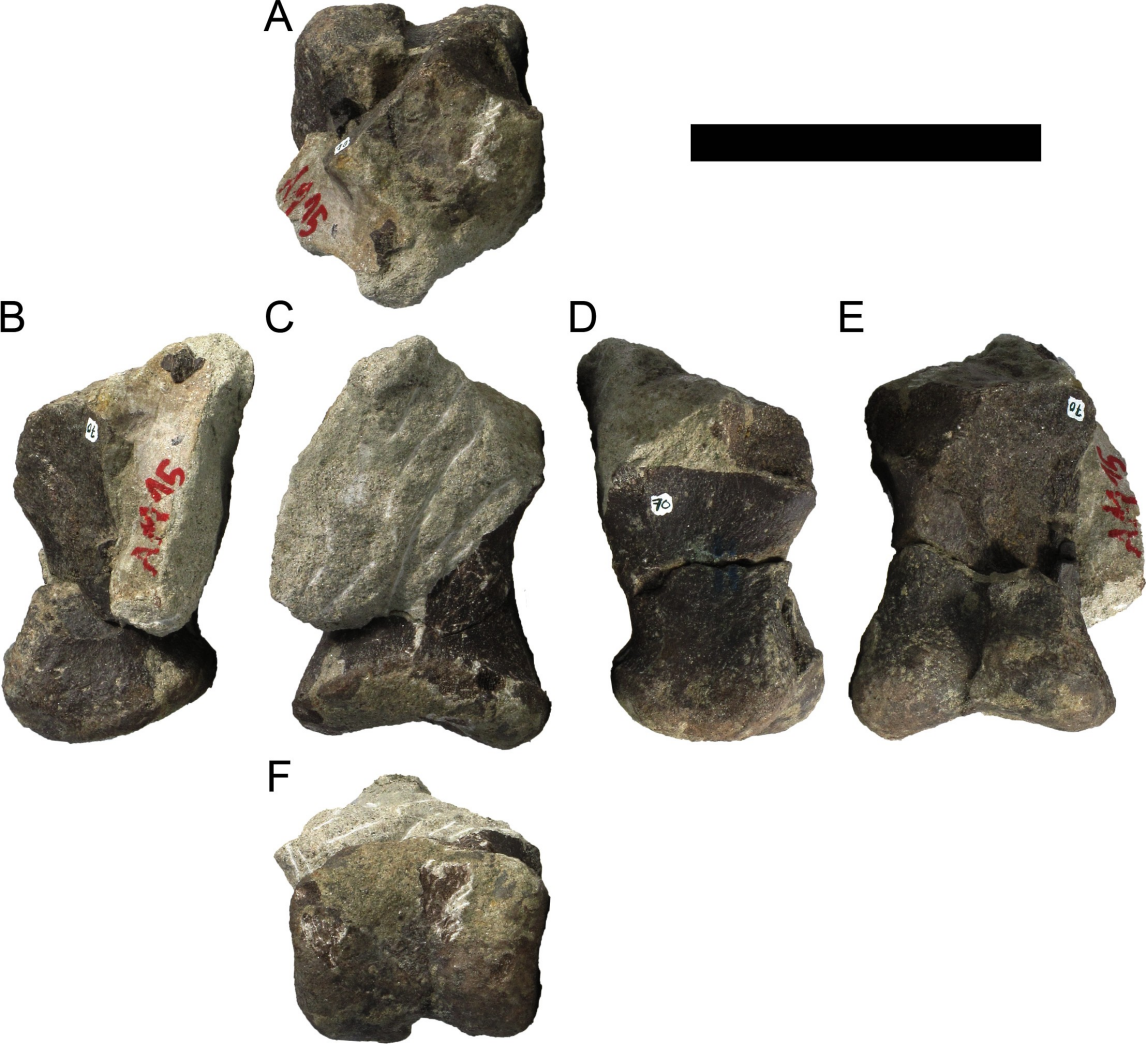
Left metacarpal one of *Miragaia longicollum* MG 4863. **A**, dorsal, **B**, medial, **C**, anterior, **D**, lateral, **E**, posterior, and **F**, ventral view. Scale bar equal to 10 cm.

Metacarpals: Two metacarpals were found semi-articulated with the anterior part of a radiale, while another was found isolated. The metacarpals and radiale are still partially unprepared and encased in matrix, so their identification is unclear at this point. These are possibly the second left metacarpal (MG 4863–71; Fig 65), third left metacarpal (MG 4863–72; Fig 65), left radiale (MG 4863–73; Fig 65) and left first metacarpal (MG 4863–70; Fig 66). All metacarpals are almost complete, but are missing their proximal articulations. The metacarpals are about equally long. They are shorter longitudinally, more slender and proportionately wider than the metatarsals found. The radiale evidences no fusion suture and is markedly concave anteriorly.

#### Osteoderms

**Fig 67:**
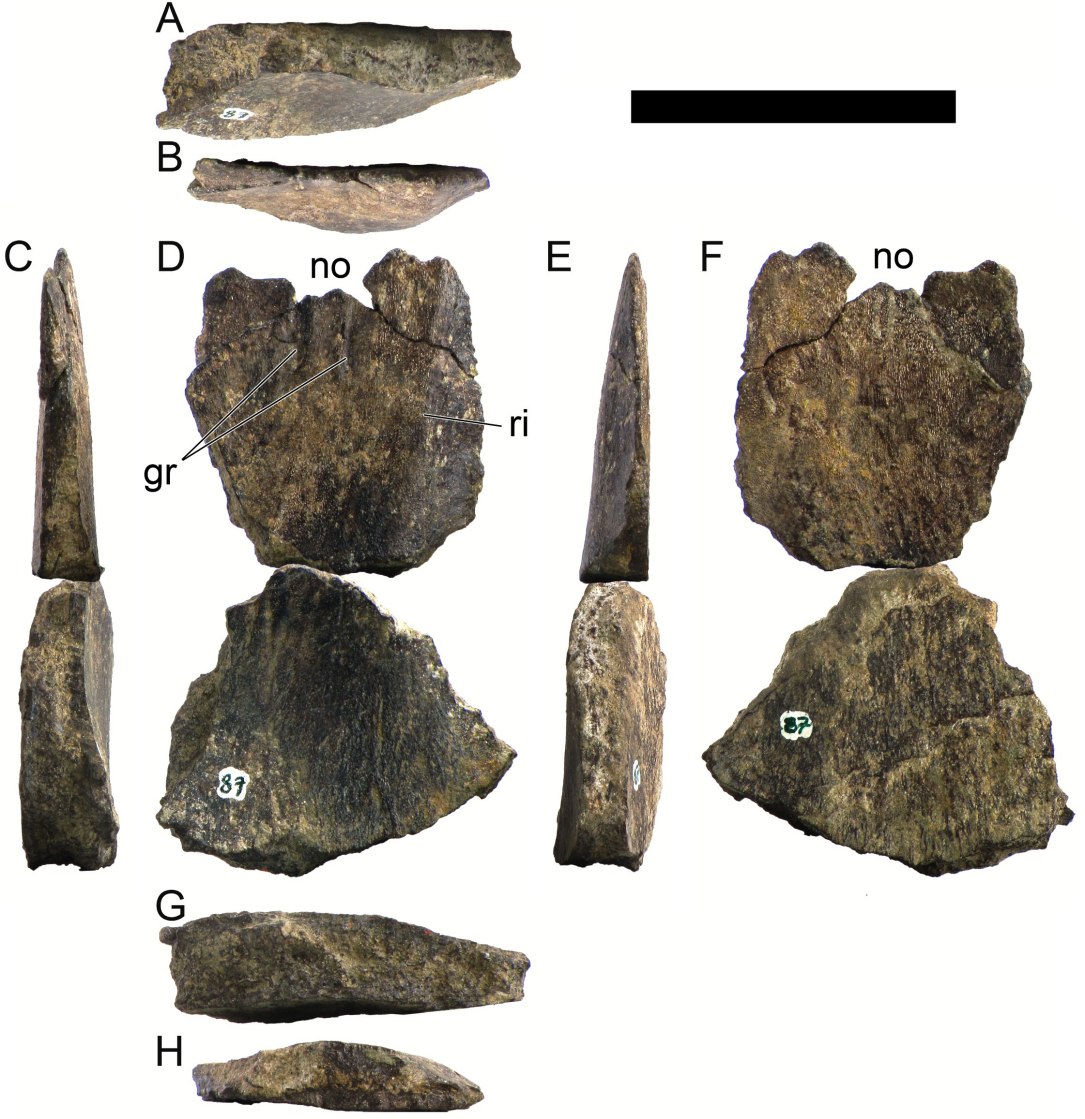
Dermal plates of *Miragaia longicollum* MG 4863. **A, C–F** (lower part), **G,** MG 4863–87, **B, C–F** (upper part)**, H,** MG 4863–46 in **A, B**, dorsal, **C**, anterior, **D**, ventral, **E**, posterior, **F**, lateral, and **G, H**, ventral view. Plates arranged how they probably fit together in **C–F**. **Gr**, groove, **no**, notch, **ri**, ridge. Scale bar equal to 5 cm.

Plates: The only unambiguous dermal plates of MG 4863 fragments present are the posterodorsal corner of a right cervical plate and a posteroventral fragment, possibly of the same plate (though the broken contiguous margins do not unequivocally fit together; Fig 67), largely similar to the most anterior cervical plates of ML 433, which allows their identification. Other fragments are probably of dermal plates (see Table 1), but are too diminutive or inconclusive to provide further useful information. The notch in the cervical plates of ML 433 was interpreted as anterodorsal by [22], but has since been reviewed as being posterodorsal, so this state is updated accordingly herein (see Discussion).

The posterodorsal fragment (MG 4863–46) is transversely thin, gently convex medially and mostly flat laterally (Fig 67E), with a scalloped dorsal edge and straight posterior edge (Fig 67D and 67F). The dorsal and posterior edges are preserved, but the ventral and anterior margins are sectioned (Fig 67B, 67C, 67E and 67H). The dorsal margin of this fragment bears in transverse view a clearly preserved notch and a projection posteriorly to it (Fig 67D and 67F). From the most posterodorsal projection, a low thicker ridge extends ventrally on the medial surface (Fig 67D). The medial surface has two short vascular grooves extending ventrally from the dorsal edge of the posterodorsal notch (Fig 67D). The medial and lateral surfaces have a fibrous texture, with low rugosities oriented ventrodorsally on the lateral surface (Fig 67D and 67F).

The posteroventral fragment (MG 4863–87) is just marginally thicker than the ventral edge of the anterodorsal fragment (Fig 67A–67C), has a similar texture (but with rougher rugosities; Fig 67D and 67F) and shows possible continuation of the thickened ridge observed in the former. It has no preserved edge. It is also mostly flat medially and convex laterally, but evidences some medial expansion at its ventral edge.

**Fig 68:**
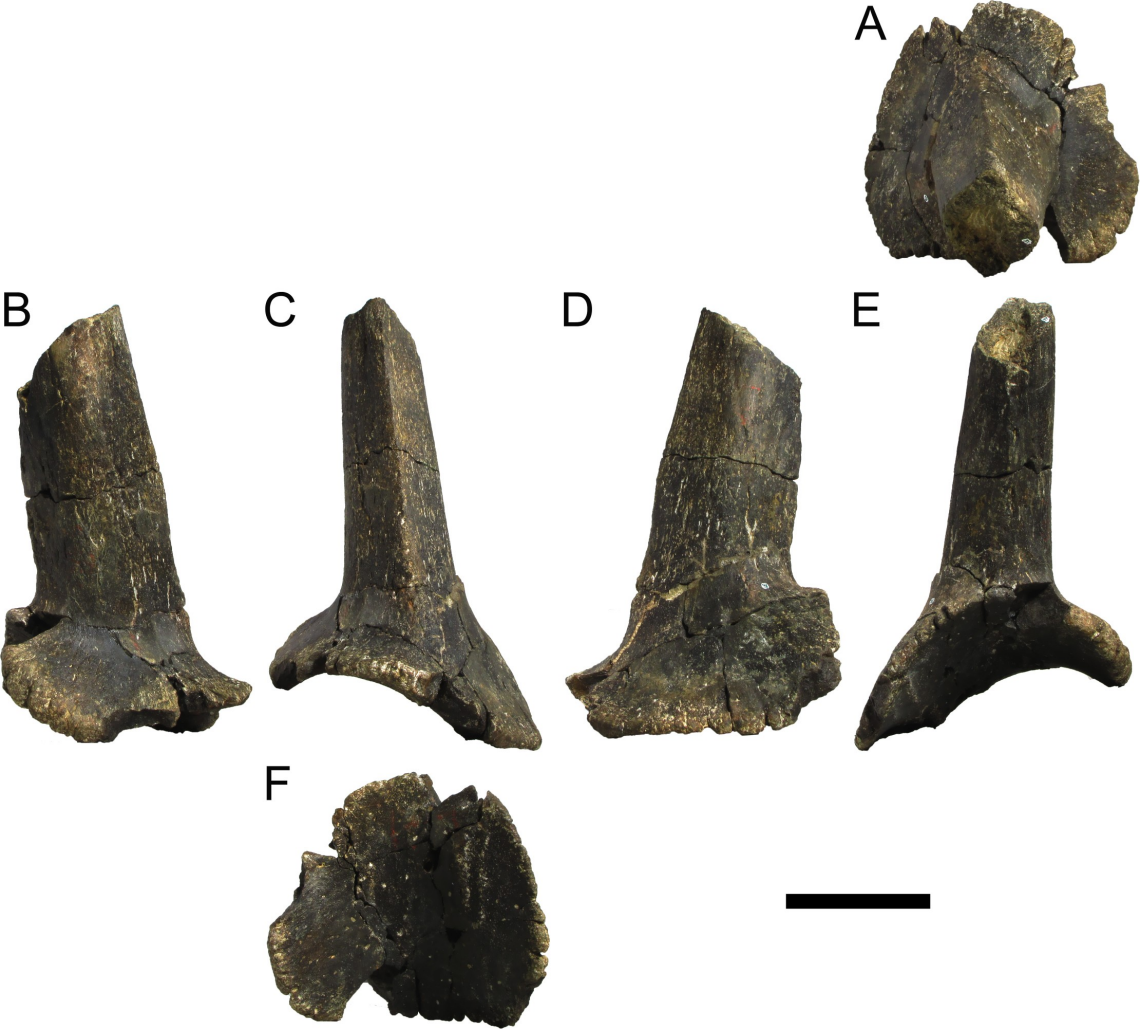
Dermal spine of *Miragaia longicollum* MG 4863 (photographs). **A**, dorsal, **B**, right lateral, **C**, anterior, **D**, left lateral, **E**, posterior, and **F**, ventral view. Scale bar equal to 10 cm.

**Fig 69:**
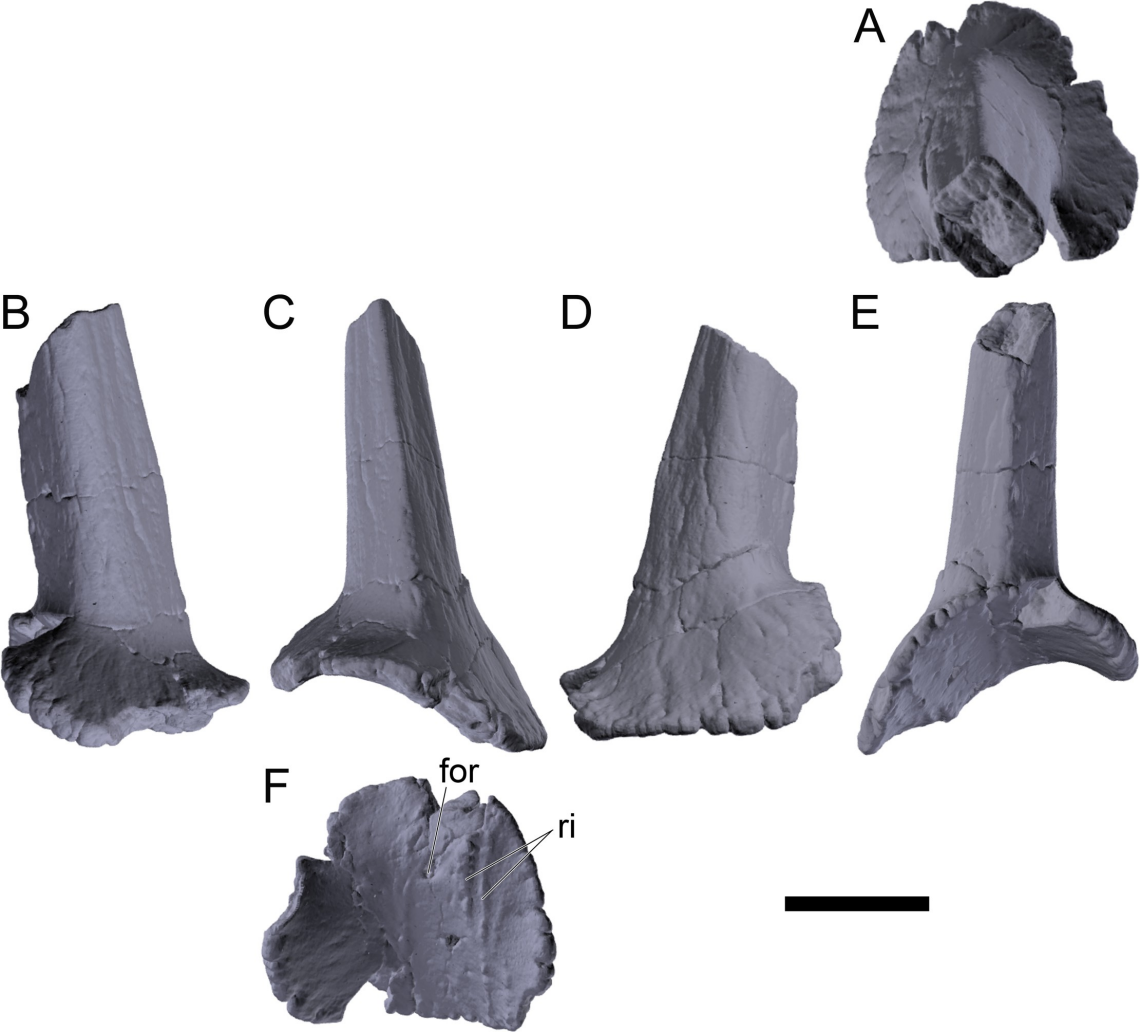
Dermal spine of *Miragaia longicollum* MG 4863 (screenshots of 3D model). **A**, dorsal, **B**, right lateral, **C**, anterior, **D**, left lateral, **E**, posterior, and **F**, ventral view. **For**, foramen, **ri**, ridge. Scale bar equal to 10 cm. Published under a CC BY license, with permission from Marco Marzola, original copyright 2017.

Spine: MG 4863–39 (Fig 68 and 69; 3D model provided in S6 Fig) is the left spine from the mid or anterior section of the tail. It is missing its distal half prior to fossilization. It is a large stocky spine with a greatly expanded base. The widest diameter of the base is more than twice the widest diameter of the proximal body of the shaft (Fig 68C). The base of the spine has a rough indented edge (Fig 68D and 68F), with a deeply anteroposteriorly concave and mostly smooth ventral surface–except for some low parallel ridges that cross it anteroposteriorly, with a most noticeable pair close to the lateral edge (Fig 68F and 69F). In ventral view, the base of the spine has a round outline with a gently concavely truncated posterior margin (Fig 68F and 69F). The median margin of the base does not have an evident surface of symphysis with the symmetrical right spine (but it is also partially broken; Fig 68B and 69B). The body of the spine is obliquely inclined from the ventral side of the base, so if the concavity in the ventral surface aligned approximately anteroposteriorly with the tail, the spine pointed posterolaterodorsally. In axial view, the spine is asymmetrical, namely the base extends more on the lateral side than on the medial side (Fig 68C and 68E). The shaft of the spine has sharp anterior and posterior edges (Fig 68C, 68E, 69C and 69E) and gently convex lateral sides, which give it a lenticular cross section (Fig 68A and 69A). In transverse and axial view, the shaft evidences gradual and gentle tapering, indicating that the spine would be elongate when complete (Fig 68B, 68C, 68D and 68E). The external surfaces of the spine are clearly covered in longitudinal vascular impressions, with a similar form to those in dermal plates of other stegosaurs [1]. The ventral surface is covered by small foramina, scattered and randomly arranged, and one large foramen close to the anterior edge of the spine (Fig 68F and 69F).

#### Unidentified material

Among the unidentified fossilized remains, some possible ossified tendons were found associated with the ninth cervical vertebra (in the same isolated block but distanced from the vertebra). These are small rods (it is unclear if there are more than one, or one that broke in separate parts), straight and with a circular section about 3 mm in diameter, with constant thickness. Ossified tendons were found associated with the dorsal neural spines of *Hesperosaurus* [1,101], which may be the case of these remains. These were not prepared at this point due to their delicate nature, hindering a complete identification and comparison currently. Other unidentified material may be cranial fragments (MG 4863–109) or parts of carpals (MG 4863–132) but are too fragmentary or not fully prepared to conclusively identify. See Table 1 for complete list of unidentified skeletal remains.

#### Ontogeny, sex and death

In thyreophorans, it has been shown that processes of increased ankylosing can be used as indicators to determine comparatively the ontogenetic stage of different specimens [1,102]. In the case of MG 4863, the lack of visible neurocentral suture in the cervical and caudal vertebrae indicates an older animal that had finished fusing the centra to the neural arches (the neurocentral suture is visible in the dorsal vertebrae, but is fully fused). A smooth curve between the ventral margin of the femoral head and the medial margin of the femora (in contrast to a sharp right-angled bend in the largest stegosaurian femora) indicates (according to [102]) that this animal had not reached its maximum growth. The lesser trochanter has been reworked and fused to the greater trochanter of the femur, as occurs in older stegosaurs [102]. Both tibiae are fused to the respective astragali and calcanea and the fibulae are fused distally to the tibiae. The occurrence of some cervical vertebrae with only one of the diapophyses fused to the respective cervical rib suggests that this specimen had not finished fusing. These observations are suggestive that MG 4863 was a young adult (probably sexually mature) but not fully grown.

The pelvis of MG 4863, although incomplete, clearly evidences it had only four pairs of sacral ribs. Galton suggests [29,102,103] that the presence of four pairs of sacral ribs in individuals of *Kentrosaurus*, *Dacentrurus*, *Stegosaurus* and *Chungkingosaurus jiangbeiensis* Dong *et al*., 1983 [104] is indicative of males (while specimens of these species with five pairs were likely females). However [19,101] find that the number of vertebrae fused together to form the sacral rod is variable within genera of Stegosauria, so the sex of MG 4863 can be suggested as male but not clearly determined.

The cause of death of MG 4863 is unknown, since there are no visible tooth marks or puncture wounds that would indicate predation or scavenging (apart for a questionable possibility in Cd32). The possibility of predation and scavenging can not be ruled out, since there could be evidences of this in the missing bone elements–the absence of which in itself may be suggestive of that. After death, the animal suffered some medium level of disarticulation, as observable by the variable instances of isolated, semi-articulated or articulated bones (though only observable locally for each block). The level of disarticulation, the fine sedimentology of the matrix with mud clasts and the lack of more movable skeletal elements (those with higher surface area per volume, namely plates and cranial elements) suggest that the animal suffered some considerable hydraulic action before deposition (i.e., died in a body of water with low to medium energy of deposition), which moved some elements and likely removed some of the missing ones. The presence of broken bones, of which the separated parts suffered distinct types of deformation (such as the right tibia and the left femur) is also indicative that these may have been moved and fractured prior to deposition.

### Comparisons

#### Cranial skeleton

The left predentary was identified based mainly on the isolated dentaries of *Kentrosaurus aethiopicus* and *Stegosaurus* sp. as illustrated, respectively, in [105] and [33]. There is the possibility that it is actually another cranial element, such as a predentary, but due to how incomplete and partial it is, interpretations and comparisons are limited. The orientation of the dentary is not clear. In ornithischians, the predentary is between the two dentaries and typically embraces the symphyses of the two dentaries, separating them by a median prong [96]. Since the predentary is missing, it is not possible to observe how the dentaries would articulate with it, but it is reasonable that the symphyseal facets of the dentaries would face medially or dorsomedially when articulating with the predentary. If positioned this way, the anterior end curves ventrodorsally, which means that in lateral view the dentaries would anteriorly evidence a convex ventral outline (in contrast to a concave outline as in other species such as *Stegosaurus* and *Huayangosaurus*; [1,33]). Most likely only more extensive comparisons of MG 4863–1 –either by direct comparisons with more complete dentaries and other cranial bones or resorting to more comprehensive descriptions of stegosaurian cranial skeletons—will result in conclusive interpretations of this bone and confirm if it is indeed a dentary or other cranial bone.

The quadrate of MG 4863 is more similar to that of *Stegosaurus stenops* than other stegosaurs (USNM 6645; [33]: Fig 6). A shallow vertical depression is not evident in the separation of the shaft and the pterygoid ramus of the quadrate, as in *S*. *stenops* and *H*. *taibaii* [100]. Since the anterior margin of MG 4863–126 is poorly preserved, it is not possible to determine if it had a quadrate foramen like *H*. *taibaii* and *K*. *aethiopicus*, or if it lacked the foramen like *S*. *stenops* [97,100]. The posterior depression is not as pronounced and extensive ventrodorsally in MG 4863–126 as in *K*. *aethiopicus* [97]. The ventral articular facet of MG 4863–126 is concave medially and slightly convex laterally in axial view, opposite of what was observed in *K*. *aethiopicus* [97].

#### Cervical vertebrae and ribs

The cervical vertebrae of MG 4863 are virtually identical to the cervical vertebrae of ML 433, evidencing or connoting compatibility with the diagnostic characters of *M*. *longicollum* defined on the latter. MG 4863 evidences a basal anterior notch and a projection dorsal to it in mid-cervical neural spines, as in ML 433 and diagnostic of *Miragaia longicollum*. Another diagnostic character of *M*. *longicollum* is the presence of at least 17 cervical vertebrae, but only a total of 12 cervical vertebrae, identified from the 2nd to the 14th cervical vertebra, were found in MG 4863. It is although clear that more posterior cervical vertebrae were present, since the most posterior cervical vertebrae found (such as Cv14) do not evidence the transitional morphology between cervical and dorsal vertebrae characteristic of the posteriormost pair of cervical vertebra, as observed for example in ML 433 ([22]; pers. obs.), NHMUK PV R36730 [19], and HMNH 001 (Hayashibara Museum of Natural History, Okayama, Japan; [1,20]), namely: neural arches much taller than the respective centrum and preceding neural arches, parapophyses more dorsally positioned than in preceding cervical vertebrae, transverse processes oriented more dorsally than in preceding cervical vertebrae (often horizontal or dorsally more, as opposed to ventrolaterally oriented), and the presence of a posterior centrodiapophyseal lamina (analogous to the paradiapophyseal lamina present in dorsal vertebrae). As such, it is anticipated that MG 4863 is missing at least two posterior cervical vertebrae, in a total of at least 16 cervical vertebrae, which is more akin and compatible to ML 433 and its 17 cervical vertebrae than to the stegosaurs with no more than 13 of cervical vertebrae–namely *S*. *stenops* and *H*. *mjosi* [19,22]. The presence of a short and transversely flat cervical rib analogous in morphology and size to the 17th cervical ribs of ML 433 (distinctive of anterior and mid cervical ribs of MG 4863 and ML 433, which bear a thick ridge and subtriangular cross section, and of the dorsal ribs, which are much longer) suggests the presence of a 17th cervical vertebra in MG 4863. It is not possible to determine the total number of cervical vertebrae in NHMUK OR46013, but considering the relatively constant total of 23 to 26–27 presacral vertebral from early thyreophorans to the most derived stegosaurs (see [22]) and the total of 14 [27] or 16 [9] dorsal vertebrae in NHMUK OR46013, it is more likely that NHMUK OR46013 would have had a total of 11 to 13 cervical vertebrae than 17 as in *Miragaia*. The most noticeable difference between the cervical vertebrae of MG 4863 and ML 433 is that the neural spines in MG 4863 appear to be less expanded transversely or dorsally (e.g. Cv14 of MG 4863 appears to have had a neural spine more equivalent in size and shape to that of Cv12 of ML 433; pers. obs.). Both specimens evidence similarly shaped and sized anterior cervical neural spines, that in both become progressively more expanded (transversely and dorsally) passing posteriorly on the cervical series, therefore larger posterior cervical neural spines, more analogous to those in ML 433, most likely occurred in the missing posterior cervical vertebrae of MG 4863. The smaller size of the neural spines also supports the previous claim that more posterior cervical vertebrae from MG 4863 are missing (as a marked increase in size of the posterior cervical neural spines is characteristic, not only of ML 433, but also of other stegosaurs such as *S*. *stenops*; [19]).

The cervical centra of MG 4863 are, like ML 433 [22], amphicoelous, anteroposteriorly longer than transversely wide, are wider than tall, but become larger and relatively less elongate through the vertebral series. One cervical centrum of NHMUK OR46013 has a convex anterior articular facet [27], a reversal of the observed in MG 4863 and ML 433. The cervical centra of *S*. *stenops* are taller than wide, except for the most posterior that appear to be as wide as tall [19]. A ventral keel in the centra as observed in other stegosaurs [19] is not evident in MG 4863. In ML 433 this ventral keel is present [22] but is only distinct in the posterior centra (pers. obs.), which are mainly missing in MG 4863. The anterior and medial centra of MG 4863 and ML 433 evidence a flatter ventral surface with a pair of rugose tuberosities in each centrum rim that protrude latero-ventrally (resembling to some level the chevron facets of the caudal vertebrae in shape and position). The anterior pair of tuberosities is more developed and swollen than the posterior, and similar processes appear to have been present in the holotype of *O*. *lennieri* [32] and anteriorly in some of the cervical centra of NHMUK PV R36730 (*S*. *stenops*; [19]).

In lateral view, the ventral margins of the centra of MG 4863 are markedly concave dorsally, like ML 433 and *H*. *mjosi*, while *Stegosaurus*, *Loricatosaurus* and *Kentrosaurus* present mostly straight ventral margins [19]. [101] claims this upwardly concave ventral margin of the axis to be diagnostic of *H*. *mjosi*. The posterior cervical centra of NHMUK OR46013 and the holotype of *O*. *lennieri* also have a straight ventral margin in lateral view [27,29].

The cervical diapophyses, parapophyses and ribs form an anterolaterally positioned fused and closed canal in MG 4863 (except in the axis) that runs horizontally and parallel to the neural canal, as in ML 433 [22]. This fused closed canal is a synapomorphy for Dacentrurinae according to [22], but [17] (supplementary information in [17]) consider it an autapomorphy of *M*. *longicollum* as it could not be found to occur in the holotype *of D*. *armatus*. On closer inspection in both MG 4863 and ML 433, the fusion is always seamless through the parapophyses, except in Cv3 where a fusion suture may be evident; the diapophyses appear to not be fused to the rib in Cv3, and a contact suture is present in some other anteriormost cervical diapophyses, indicating that they are not fully fused to the rib; in ML 433, the two posteriormost cervical vertebrae (Cv16 and Cv17) do not appear to have fused contacts to the ribs. The holotype of *O*. *lennieri* (currently *Dacentrurus* sp. according to [9]) also had cervical ribs fused to the 3rd to 10th cervical vertebrae, but not on the 11th and 12^th^ [29]. The two posterior cervical centra of NHMUK OR46013 bear unfused and similar parapophyses, with visible articular facets in one of them (according to [27]), but it is unclear what exact position these occupied in the cervical series and if the absence of fusion is due to occupying the posteriormost position in the cervical series (as in Cv16 and Cv17 of ML 433).

The transverse processes are long anteroposteriorly, about double the length of the prezygapophyses, extending anteroposteriorly over all the anterior half of the centrum–that is, from mid-length of the prezygapophyses to beyond the posterior margin of the neural spine, as in the cervical vertebrae of ML 433 (pers. obs.). Also, the bases of the diapophyses of MG 4863 and ML 433 sprout ventrally to the prezygapophyses. By contrast, in NHMUK OR46013 and the holotype of *O*. *lennieri*, the transverse processes are borne on the sides of the prezygapophyses and are as long as these (according to [27]), projecting anteriorly closely adjacent to the anterior edge of the prezygapophyses and reaching posteriorly only as far as the posterior margin of these and level with the anterior margin of the neural spine. *H*. *mjosi* HMNH 001 and *S*. *stenops* NHMUK PV R36730 also appear to have transverse processes positioned ventroposteriorly to the prezygapophyses as in MG 4863 and 433, but are not as long anteroposteriorly.

The outline in lateral view of the prezygapophyses of MG 4863 is round on the posterodorsal margin and straightens anterodorsally, with a slight but noticeable notch on the anterodorsal margin and an anteriormost projection ventrally, just as is observable and described in ML 433 [22]. By contrast, the prezygapophyses in NHMUK OR46013 are round in outline anteriorly and straighten on the posterior margin. The posterior margin of the prezygapophyses of MG 4863 and ML 433 are divergent from the roof of the neural arch by a sharp concave curve (more pronounced in posterior cervical vertebrae, to a degree that the posterior margin of the prezygapophyses is almost vertical), while in the posterior cervical neural arch of NHMUK OR46013 the posterior margin of the prezygapophyses is close to horizontal and merges in a gentle curve with the top of the neural arch.

The postzygapophyses of MG 4863 project beyond the posterior centrum face, as in ML 433, *Stegosaurus* and *Loricatosaurus*, which is a synapomorphy of Stegosauridae [19,22]. A pair of spinopostzygapophyseal laminae are present in both MG 4863 and ML 433 ([22]; pers. obs.). The spinopostzygapophyseal laminae extend anteriorly and almost horizontally from the epipophyses (which are present dorsally on the postzygapophyses) passing laterally on the sides of the neural spine and culminating on the anteriormost projection on the base of the neural spine (the presence of this anteriormost projection of the neural spine, and the notch ventrally to it, is one of the diagnostic characters of *M*. *longicollum*; [22]). ML 433 also shares with MG 4863 the same pair of parallel accessory lower crests, that in most instances also pass laterally on the sides of the neural spines, and the central deep fossa medial to these and posterior to the neural spine (which also becomes deeper passing posteriorly on the cervical series). As such, both MG 5863 and ML 433 always have two ridges (or, less often, one ridge) in each lateral side of the cervical neural spines. In *D*. *armatus* (NHMUK OR46013), *S*. *stenops* (NHMUK PV R36730) and *H*. *mjosi* (HMNH 001) a pair of anteroposterior ridges over the postzygapophyses may be present in some vertebrae, but these, when observable, culminate on the posterior margin of the neural spine (that is, do not carry on laterally on its sides or until its anterior margin), are much lower and less distinct than those observed in MG 4863 and ML 433, face posterodorsally in lateral view, are not as extensive posteriorly, are not accompanied by a pair of medial parallel lower ridges and the sides of the neural spines are smooth and without ridges present.

The neural spines of MG 4863 are transversely narrow ridges in anterior cervical vertebrae, positioned mid-length to the centrum and about equidistant to pre- and postzygapophysis in the axis and Cv3. Passing posteriorly, these progressively become wider transversely, taller dorsoventrally and positioned more anteriorly (i.e., closer to the prezygapophyses and anterior face of the centrum than to the postzygapophyses and posterior face of the centrum). This also occurs in ML 433 (from Cv3 to Cv17) while the reverse is observed in other stegosaurs as the neural arches are positioned progressively more posteriorly passing posteriorly [1,19]. In the posterior neural arch of NHMUK OR46013 the neural arch is positioned about equidistant from the pre- and postzygapophysis according to [27]. The anteriorly positioned neural spines of MG 4863 and ML 433 also mean the posterior edges of the prezygapophyses overlap or reach as far as the anterior edge of the neural spines in lateral view in mid and posterior cervical vertebrae, while in the posterior cervical vertebra of NHMUK OR46013 these margins are far apart ([27]: Fig 3O, 3P, 7K and 7L). In *H*. *mjosi* (HMNH 001) and *S*. *stenops* (NHMUK PV R36730) the prezygapophyses and neural spines overlap similarly in lateral view, but mostly because the base of the neural spines increases in length passing posteriorly–while in MG 4863 and ML 433 the base of the neural spine retains a mostly constant anteroposterior length along the cervical series, as it is the apices which chiefly expand axially.

The apex of the posterior cervical neural spine of NHMUK OR46013 expands greatly posteriorly (and the anterior cervical neural spine also appears to evidence posterior expansion of the apex), which is not observable in any of the cervical neural spines of ML 433 or MG 4863, that evidence continuous and vertical posterior margins of the neural spines. A marked posterior expansion can not be observed in any of the cervical neural spines of *Stegosaurus* or *Hesperosaurus* [19,20].

The cervical ribs of MG 4863 also project posteriorly beyond the posterior centrum surface and have an anterior projection from the capitulum that projects anteriorly to a point level or beyond the anterior centrum face, as in ML 433 [22].

A cervical vertebra from Barrihonda–El Humero site, Teruel (CPT-1330; Fundación Conjunto Paleontológico de Teruel Museum, Spain) is much like those of ML 433 and MG 4863, with an amphicoelous centrum longer than wide and wider than tall, a marked concave ventral margin of the centrum in lateral view, a pair of anterior tuberosities in the ventral surface and an anterior projection from the fused cervical ribs.

The odontoid process of the axis of MG 4863 evidences a suture between it and the centrum, so it is not fully fused to the axis. The odontoid process is also unfused in *H*. *mjosi* [19] but is ankylosed to the centrum of the axis in *Huayangosaurus*, *Kentrosaurus*, *Stegosaurus* and *Loricatosaurus* [1,19]. The ventral margin of the axis is concave in lateral view as in *H*. *mjosi*, differing from the flat ventral margins of *Stegosaurus*, *Loricatosaurus* and *Kentrosaurus* [19]. The odontoid process is positioned at mid-height of the anterior face of the centrum, more akin to *H*. *mjosi* than *S*. *stenops*, *Loricatosaurus* or *Kentrosaurus*, which have more dorsally positioned odontoid processes [20,19]. The dorsal margin of the neural arch of the axis of MG 4863 in lateral view is almost parallel to the neural canal, but this is most probably an artifact of the deformation it suffered, as in other stegosaurs (such as *Hesperosaurus*, *Stegosaurus* and *Loricatosaurus*) it is much steeper, facing anterodorsally and giving the neural arch a sub-triangular outline in lateral view ([20]: Fig 3.7; [19]).

#### Dorsal vertebrae

As is typical of stegosaurs, the pedicels of the dorsal vertebrae of MG 4863 are quite tall, about 1.5 times the height of the centrum, as in *Huayangosaurus*, *Dacentrurus*, and *Hesperosaurus* [1]. Although the mid-dorsal vertebrae of stegosaurs typically have strongly upturned diapophysis at an angle between 50 and 60 degrees to the horizontal [1], the dorsal diapophyses of MG 4863 only project at an angle of about 30 degrees to the horizontal, more similar to ML 433 and NHMUK OR46013 [27].

All the dorsal centra of MG 4863, like the two anterior dorsal vertebrae of ML 433 and the posterior two-thirds of the dorsal series of *Dacentrurus* [1], are wider than long, which is a synapomorphy of Dacentrurinae [22]. The dorsal centra of MG 4863 also appear to become slightly wider passing posteriorly, as also occurs in *D*. *armatus* [1]. In other stegosaurs the reverse occurs, as they become more elongate passing posteriorly [1,19]. This state can not be clearly compared currently though, considering the currently incomplete preparation of the dorsal vertebrae of MG 4863, their preliminary identification that the two most anterior vertebrae appear to have been slightly deformed transversely by compressive forces.

[22] notice that the transverse expansion of the apices of the anterior dorsal neural spines of ML 433 can be diagnostic of *M*. *longicollum*. By contrast, the first and second dorsal neural spines of *S*. *stenops* are thin and uniformly thick, with no transverse expansion distally ([19]: Figs 18 and 19), but the apices of the neural spines become progressively more transversely expanded passing posteriorly on the dorsal series [19]. The only preserved dorsal neural spine of MG 4863 is that of the purportedly 5th dorsal vertebra, which evidences clear expansion of the apex from the upper third of the neural spine, wider than what is observed in mid dorsal neural spines of *S*. *stenops* ([19]: Figs 20–23). This may suggest that MG 4863 could have had anterior dorsal neural spines also with wider distal expansions than the equivalent, at least, in *S*. *stenops* [19], but this can not be confirmed currently. The third or fourth dorsal vertebra of NHMUK OR46013 (Fig 1XII and 1XIII; [12]: plate XII and XIII; [27]) has the best preserved dorsal neural spine of the animal, with straight and vertical sides in all height that suggest it had no transverse expansion distally, but the apex is partially broken so this can not be fully demonstrated.

#### Caudal vertebrae

All caudal centra of MG 4863 are wider than long or tall, as well as taller than long, alike to the posterior caudal vertebrae of *Alcovasaurus* [18]. The caudal vertebrae of NHMUK OR46013 are also wider and taller than long, but it is unclear if they are wider than tall as in MG 4863 [27]. All the caudal vertebrae of *S*. *stenops* are longer than wide and taller than wide [19], differing from MG 4863. According to [18], the posterior caudal vertebrae of all other known stegosaurs are transversely compressed and the length exceeds the height (including *H*. *mjosi*, *H*. *taibaii*, *L*. *priscus*, *C*. *jiangbeiensis* and *T*. *multispinus*), differing from MG 4863.

In anterior vertebrae of MG4863 the centrum outline in axial view is round and slightly dorsoventrally flattened. In medial and posterior caudal vertebrae the axial outline of the centrum is round to apple-shaped, with the neural canal excavating a semi-circle shaped concavity in the dorsal surface and a flattened base between the chevron facets, similar to *Alcovasaurus* [18] and differing from the sub-pentagonal to rhombic outline observed in *Stegosaurus* [19] and *Loricatosaurus* [27]. This difference in outline is also due to the lack of a horizontal lateral ridge in any of the caudal centra of MG 4863 (present in other stegosaurs such as NHMUK PV R36730; [19])–instead, in all but the most anterior caudal vertebrae, the centrum articular rims are notably expanded transversely and ventrally, giving a deeply concave outline to the centrum sides in dorsoventral and lateral view (as was also observed in *Alcovasaurus*; [18]). In mid and posterior caudal vertebrae of MG 4863, the ventral area between and including the chevron facets is flat or more concave than the rest of the centrum wall in axial view, differing from the central keel present in *S*. *stenops* [19]. The anterior caudal centra of NHMUK OR46013 resemble MG 4863 in outline, but the mid and posterior caudal centra have a round outline, not evidencing the same depression of the neural canal or flattening of the ventral surface as in MG 4863 ([27]: Figs 2O, 8M, 8N and 12U–12X).

The caudal vertebrae of MG 4863 do not evidence any ossified processes for the haemal canal as observed in the chevron facets of NHMUK OR46013 [27] or *Stegosaurus* ([106]: p288-291).

The processes just ventral to the neural canal and concentric ridges (that converge dorsally in the aforementioned processes) that occur in some of the articular caudal facets of MG 4863 were not found to occur in NHMUK OR46013 or other stegosaurs (such as NHMUK PV R36730; [19]), which instead evidence uniformly concave centrum surfaces with irregular or smooth articular facets. No processes in the center of the caudal articular facets were observed or found either to be reported in NHMUK OR46013 or other stegosaurs [19,27], alike those that occur often along the caudal series of MG 4863.

The neurocanal and neural arch are in all (but the anteriormost) caudal vertebrae of MG 4863 much reduced, about one third or less the width and the height of the centrum (dismissing the neural spine and zygapophyses), unlike *S*. *stenops* where the neural arch is tall and mostly as wide as the centrum [19] but similarly to *Alcovasaurus* ([18]: Fig 4). The anteriormost caudal vertebrae of NHMUK OR46013, similarly to MG 4863, have wide neural arches almost as wide as the respective centrum ([27]: Fig 8)–but the mid caudal vertebrae clearly do not have reduced neural arches, being more than two thirds the width and height of the centrum, while the neural canals are half the centra width ([27]: Figs 2O, 8M, 8N and 12U–12X), differing from those in MG 4863. Due to the larger neural arch, the mid caudal prezygapophyses in NHMUK OR46013 are also much more widely spaced transversely than those in MG 4863 ([27]: Fig 12U–12X).

The most anterior caudal ribs of MG 4863, analogous to other stegosaurs, are laterally prominent and decrease in size passing posteriorly [1]. In MG 4863, these are elongate and about as wide as the centrum from Cd1 to Cd5, dorsoventrally thin blades from Cd8 to Cd13, depressed stubs or rugosities from Cd14 to Cd36 and are reduced to a vestigial ridge in Cd37. This is most similar to *Alcovasaurus*, that retains vestigial transverse processes in posterior caudal vertebrae (presumably caudals ca. 40 to 49; [18]), it is also similar to *Hesperosaurus*, in which the transverse processes are nubbins on Cd20 and disappear at Cd33 [1,20], and to *Kentrosaurus*, where small swellings are present to at least Cd29 [19], but unlike *S*. *stenops*, *H*. *taibaii*, *L*. *priscus*, *C*.*jiangbeiensis* and *T*. *multispinus*, that have no caudal ribs or transverse processes by Cd17 or Cd18 [18]. The mid caudal vertebrae of NHMUK OR46013 evidence reduced transverse processes ([27]: Figs 2O, 8M, 8N and 12U–12X) but, unlike MG 4863, the posterior caudal vertebrae do not have transverse processes [27]. The distal continuation of the transverse processes in MG 4863 should allow for greater lateral tail muscles for enhanced effectiveness of the tail spines as weapons, as suggested by [18] for *Alcovasaurus*.

The axial rugose ridges and anterior projections present proximodorsally in mid caudal transverse processes were not observed or found to be reported in NHMUK OR46013 or any other stegosaur.

The ribs of the anterior caudal vertebra of MG 4863 project laterally and almost horizontally, only marginally curving ventrally (so more akin to *Huayangosaurus*, *Dacentrurus*, *Kentrosaurus*, and *Wuerhosaurus* than to *Chungkingosaurus*, *Hesperosaurus*, *Loricatosaurus* and *Stegosaurus*, which project ventrolaterally; [1]). The anterior caudal ribs of NHMUK OR46013 are more ventrally oriented than those in MG 4863 ([27]: Fig 8), with a convex dorsal margin in axial view (while MG 4863 evidences straight or slightly concave dorsal margins) and a concave and ventrally curved ventral margin (while MG 4863 evidences mostly straight horizontal margin of the dorsal ribs).

The anterior caudal neural spines of MG 4863 are tall, straight and have moderately expanded and non-bifurcated apices (similarly to NHMUK OR46013 and other stegosaurs, while in *Stegosaurus* the apices also bifurcate; [1,19]), but the apices of MG 4863 are clearly more expanded anteroposteriorly than transversely–the opposite was a synapomorphy for Stegosauria according to [1] and a synapomorphy for Stegosauridae according to [9]. As a result, in dorsal view the expanded apices of the anterior caudal neural spines of MG 4863 are wider anteroposteriorly than transversely, reversed of what is observable in NHMUK OR46013 ([27]: Fig 8) and *Stegosaurus* [33]. The shaft of the anterior caudal spine of MG 4863 is also much more compressed transversely than the equivalent in NHMUK OR46013 ([27]: Fig 8). Like MG 4863, *L*. *priscus* also has anterior caudal apices more expanded transversely than axially (NHMUK OR3167; [27]: Fig 17).

The neural spines in MG 4863 become increasingly more posteriorly oriented passing posteriorly in the caudal series, while these retain a mostly constant dorsal or posterodorsal orientation in most stegosaurs [1], including in NHMUK OR46013 [27], *S*. *stenops* (NHMUK PV R36730; [19]) and *L*. *priscus* [27], while in *K*. *aethiopicus* these become more anteriorly oriented [1,107]. The neural spines of caudals 8 to 11 of MG 4863 are oriented posteriorly at less than 45 degrees to the horizontal, so strongly inclined posteriorly that their apices reach posteriorly beyond the posterior vertebra. In *L*. *priscus* (NHMUK OR3167; [27]: Fig 17) the apices of the anterior caudal neural spines also reach beyond the posterior vertebra, but mainly because the neural spines are extraordinarily long (more than twice the height of the centrum), and these do not become more posteriorly oriented as in MG 4863, retaining an orientation of approximately 45 degrees within the series.

The sharp decrease in size of the caudal neural spines of MG 4863 between Cd10 and Cd12 (from a spine taller than the centrum in Cd10 to a low and transversely thin ridge in Cd12) was not found to occur in any other stegosaur, where they progressively decrease in size passing posteriorly on the caudal series [1,19]. By Cd18 the neural spine is absent in MG 4863, while it only disappears entirely by Cd35 in *S*. *stenops* and by Cd33 in *Hesperosaurus* [19,20]. At least one mid caudal vertebra of NHMUK OR46013 evidences a large and tall neural spine ([27]: vertebra identified as P in Figs 4A, 5A and 6A) while in another ([27]: Q in Figs 4A, 5A and 12W) it is unclear if the neural spine (which is partially encased in matrix) is smaller or thinner than those from more anterior caudal vertebrae of NHMUK OR46013 and MG 4863.

The caudal prezygapophyses of MG 4863 project anteriorly and extend beyond the anterior facet of the centrum, similarly to *Stegosaurus*, *Gigantspinosaurus* Ouyang, 1992 [108], *Huayangosaurus* and *Kentrosaurus*, but unlike *Loricatosaurus* [19]. The postzygapophyses are small, borne on the base of the neural spine, and do not extend beyond the posterior facet of the centrum, akin to *S*. *stenops* and differing from *Loricatosaurus* and *Kentrosaurus* [19].

A small proximodorsal process, like those occurring in the anterior caudal ribs of *Dacentrurus*, *Kentrosaurus*, and *Wuerhosaurus* [1] is present in the ribs of Cd2 to at least Cd5 of MG 4863. The first caudal vertebra of NHMUK OR46013 also evidences this prominent anterodorsal projection ([27]: Figs 6H and 12V), but the first caudal vertebra of MG 4863 has instead a closed proximodorsal canal in each rib. This canal seems to have a similar ontogenetic origin to the anterodorsal processes in other stegosaurs, but in the case of MG 4863 it develops medially until it meets the base of the neural spine and closes in the aforementioned canal. These closed proximodorsal canals were not found to occur in the ribs of the first or other caudal vertebra of other stegosaurs, including *Stegosaurus* ([106]: p293) and *Loricatosaurus* ([27]: Fig 17T). The dorsally expanded proximodorsal projections in the ribs of an anterior caudal vertebra from the Barrihonda–El Humero site, Riodeva, Spain (CPT-1434; [56]) form an open arch (which becomes less noticeable in other anterior caudal vertebrae from the site; [56]), but it is not fully closed as observed in MG 4963.

The most posterior caudal vertebra of *A*. *longispinus* ([33]: Fig 67) evidences what appears to be analogous lateral ossification and thickening of the centrum rims as observed in Cd36 and Cd37 of MG 4863 (Figs 47B, 47D, 48B and 48D). This ossification may be loosely suggestive that these vertebrae bore over them one of the pairs of spines from the thagomizer (ankylosing and fusion observed in some posteriormost caudal vertebrae of *Kentrosaurus* may have been for this purpose; [107]: Fig 5).

#### Dorsal ribs

The capitula of most of the dorsal ribs of MG 4863 are set at angles of about 45 degrees with the rib shafts, whereas in most stegosaurs (according to [1]) the capitula are perpendicular to the shafts, giving them a deep and narrow rib cage, meaning that MG 4863 could have had a wider rib cage than other stegosaurs. A similar range in variation of the angles of the capitula is observable in the ribs of more complete specimens (e.g., NHMUK PV R36730; [19]) depending on the axial position of the ribs, suggesting the variation in MG 4863 could be for the same reason and not an anatomical adaptation. The cross sections of the proximal halves of the ribs of other stegosaurs are T-shaped, with the flat surfaces on the outside [1], while in MG 4863 it is concave medially and convex laterally, evidencing more of a C-shaped cross section. This cross section is also visible in the dorsal ribs of ML 433 (pers. obs.).

#### Chevrons

The chevron MG 4863–52 is Y-shaped in axial view, with expanded articular surfaces, similar to the chevrons of other stegosaurs. The articular ends are not united by a medial bridge enclosing the haemal canal as occurs in chevrons of other stegosaurs (e.g., [19,33]). However, the haemal canal was found to be variably open or closed in the same individual of *Stegosaurus* [1,33]. A pair of secondary grooves, present just ventrally to the articular facets in MG 4863–52, are not observable in a chevron from NHMUK OR46013, but similar grooves appear to be present in *Stegosaurus* (YPM 1853, the holotype of *S*. *ungulatus*; [106]). A preserved chevron of ML 433 has an open haemal canal and a blade relatively longer and not as deep as MG 4863–52 (more akin to MG 4863–51) and does not appear to evidence grooves ventrally to the articular facets (pers. obs.).

#### Pelvic Girdle

The preacetabular process of the left ilium of MG 4863 is almost completely missing, but it is possible to observe that it was wide at the base. Nor the preserved medial margin or ventral mold conclusively indicate its total length, only evidencing that it was longer than the anterior margin of both. As such, it is not possible to observe if the anterior process was long or widened anteriorly as in NHMUK OR46013 and the stegosaurs from MG [27,28] or if it was particularly short–a feature that has been proposed as diagnostic of *Dacentrurus armatus* [30]. [30] also propose that a smooth curvature between the anterior margin of the sacral plate and the medial margin of the preacetabular process may be diagnostic of *D*. *armatus*, but other stegosaur species also evidence a similar state (such as *Hesperosaurus mjosi*; [20]: Fig 3.10 and 3.11). In most other respects, the ilium of MG 4863 is mainly similar to the ilia of the stegosaurs at MG (from Atalaia, Murteiras and Pedras Muitas) and NHMUK OR46013, including a wide and long supracetabular process that extends anteriorly beyond the anterior edge of the acetabulum [30].

In *Dacentrurus*, *Hesperosaurus*, *Stegosaurus*, and *Wuerhosaurus* the prepubis is deeper than the postpubis [1], and this is particularly evident in *Dacentrurus* as the prepubis is about twice as deep than the proximal postpubis [27]. However, in MG 4863 both are deep, with the proximal postpubis only marginally less deep than the prepubis. As a result of this and the large and round acetabular process, the obturator notch is closed in lateral view (as the margins of the postpubis and acetabular process clearly overlap each other medially), a condition that is observable in no other stegosaur species besides *Huayangosaurus* [109]. The posterior edge of the acetabular process of the pubis of NHMUK OR46013 is broken [27] and appears to have been reconstructed since with plaster, but at least anteriorly the obturator notch would still be open, and it is unlikely it overlapped posteriorly the postpubis. The pubes of the juvenile associated with the holotype of *M*. *longicollum* (ML 433-A) also evidence postpubes as deep as the prepubes, but the acetabular process is relatively not as enlarged (pers. obs.). The anterior end of the prepubis of MG 4863 is only expanded dorsally and the ventral margin curves dorsally, as is also observed in ML 433-A, while the anterior end of the prepubis of NHMUK OR46013 expands both dorsally and ventrally [1]. The pubis of the stegosaur from Pedras Muitas (MG 4850) has an evidently (despite partially broken) large acetabular process, much akin to the one in MG 4863, but the anterior end of the prepubis is expanded both dorsally and ventrally, the prepubis is much deeper than the postpubis and the obturator notch is not closed in lateral view, which is much more alike the pubis of NHMUK OR46013. However, various authors have pointed out that the pubes of stegosaurs may have some individual variation, as variation of this bone is observable among specimens of some stegosaur species and other dinosaurs [19,27,101].

Of the ischia of MG 4863, only the left iliac peduncle unequivocally remains (MG 4863–65), while NHMUK OR46013 has almost complete ischia, but the iliac peduncle of the left ischium is broken and badly preserved, while the iliac peduncle of the right ischium is mostly encased in matrix in the main block of the specimen, so comparisons are very limited between these bones. The dorsal articular facet of the iliac peduncle of MG 4863 appears, though, to be more expanded longitudinally relative to its shaft than the equivalent in NHMUK OR46013, *S*. *stenops* or *H*. *mjosi* [1,19,27]. The strongly curved anterior edge of MG 4863–65 may also be indicative that the shaft of the iliac peduncle was much shorter ventrally than that of NHMUK OR46013 [27]. The posterior margin of MG 4863–65 is, in the visible respects, similar to those of NHMUK OR46013, with similar gentle curvature and rugose dorsal expansion of the articular facet. A straight dorsal surface of the ischium is diagnostic of *Dacentrurus armatus* (according to [9]). MG 4863–114, which is possibly a fragment of the distal end of one of the ischia, is straight at both margins, but it is too short and inconclusively identified to be representative of the margins of the ischium. The ischium of the stegosaur from Pedras Muitas (MG 4852) has a similar but deformed iliac peduncle to MG 4863–60 (including the same shaped concavities anteriorly and posteriorly and same rugosities dorsally) and has a straight dorsal surface like the ischium of NHMUK OR46013.

#### Femora and Tibiae

The left tibiae are much shorter than the femora, with the femora 159% the length of the tibia (akin to *Loricatosaurus* and *Stegosaurus* with a femur-to-tibia ratio of, respectively, 150% and 168%-185%; [1]). The fourth trochanter is almost indistinct, as is typical of stegosaurs, represented in MG 4863 only by a slight but distinct swelling as in *Dacentrurus* [1,27].

The same type of longitudinal rugose ridges that cross both anterior and posterior sides of the femora of MG 4863 also occur in the femora of *Dacentrurus* sp. from Pedras Muitas, Murteiras and Atalaia at MG. [29] describes these in the femur from Pedras Muitas (MG 4935) as “bony longitudinal cords” and “narrow raised, rugose and gently sinuous” which (just like in MG 4863) [29] notes one progresses distally in the anterior side of the femoral shaft from where the lesser trochanter would be, while two other parallel ones occur on the posterior side. Also like in MG 4863, the anterior ridge in MG 4863 bifurcates distally in MG 4935. The femur from Atalaia (MG 4969) evidences two longitudinal ridges on the posterior side and a less clear ridge on the anterolateral side, matching those in the femora of MG 4863. The femur from Murteiras (MG 4877) also evidences an anterolateral ridge with distal bifurcation as in the femora of MG 4863 and from Pedras Muitas, but posteriorly other ridges are present but less clear. These ridges may be correlated with the ontogeny and exclusive to adults, as they are less distinct or do not occur in juvenile specimens [29], such as the small femur from Alfeizerão (MG 4971) that does not evidence clear ridges. These cords are not present in the femur of NHMUK OR46013 (but the anterior side of the femur of this specimen is mostly encased in matrix; [27]) and were not found arranged in the same pattern in other stegosaur, apart for in a left femur from the Barrihonda–El Humero site, Teruel (CPT-1304: [56]: Fig 4) that appears to evidence similarly shaped and positioned bony cords as in the femora of MG 4863 (including the same bifurcation distally of the anterior ridge). Another femur (NHMUK R1989, part of an indeterminate stegosaur from the Callovian of UK; [9]) bears several ossified cords [27], but apparently more abundant than in MG 4863 and of variable sizes, as well as crossing each other (instead of mainly parallel as in MG 4863).

The fact that the longer axes of the ends of the left tibia of MG 4863 are set at an angle of about 45 degrees (as opposed to in the same plane, as occurs in other stegosaurs; [33]) is probably due mostly to deformation but, if it is to some extent intrinsic of the species, it would be an adaptation closer to what is observed in ornithopods and ceratopsians [33]. The tibia of NHMUK OR46013 has a slender shaft and distal end that strongly expands laterally and only scarcely expands medially [27] while the left tibia of MG 4863 has a much thicker shaft and a distal end that expands almost equally laterally and medially.

#### Metatarsals

The metatarsals are almost identical to the metatarsals of YPM 4836 (S*tegosaurus* sp.; [106]: p306-311), which are similar as well to the metatarsals of *Dacentrurus* and *Loricatosaurus* [1]. The right fourth metatarsal of MG 4863 (MG 4863–66) resembles more the equivalent from YPM 4836 than NHMUK OR46013 (which is, for example, much less expanded posteriorly than the counterparts from MG 4863 and YPM 4836, most noticeably proximally; [27]: Fig 11G and 11H). MG 4863–66 also has a proximal articulation rounder in outline and more perpendicular to its shaft than the fourth metatarsals of NHMUK OR46013 and YPM 4836 (which have subtriangular proximal articulations that articulate obliquely with the shaft; [106]: p339; [27]). A dorsally projecting process present approximately in the center of the anterior face of both third metatarsals is not observable in the equivalents of other stegosaurs ([27]: Fig 11G; [106]: p306-311).

#### Osteoderms

MG 4863–39 is strongly similar to the spines of NHMUK OR46320 (the holotype of *Omosaurus hastiger*; [27,31]; Fig 1XXII and 1XXIV) and FUB A (a spine from between Porto Dinheiro and Porto das Barcas; Freie Universitat, Berlin, Germany; [29]). This spine–with its greatly expanded base, smooth ventral surface and sharp anterior and posterior edges–is markedly different from the spine from NHMUK OR46013, that has a slightly expanded base, strongly sculpted ventral surface and rounded cross-section (Fig 1XXI). MG 4863–39 has two low parallel ridges ventrally that cross it anteroposteriorly. One similar ridge is also present in NHMUK OR46320, two in the base of a spine from Porto Dinheiro (identified as MSGP J in [29]) but not in FUB A [29]. Although similar, a notable difference between MG 4863–39 and the spines of NHMUK OR46320 (considered Stegosauria indet. according to [110]) is that the latter have a mostly convex ventral margin, while the former has a markedly concave ventral margin. According to [27,29], stocky spines like MG 4863–39 probably had a position in the tail more anteriorly than in the distal end. Taxonomic interpretations based on spines of stegosaurs should be reserved though, as their morphology is likely to be extremely variable depending on age, size, and the sex of the animal that bore them [9], and in some examples the range of different morphologies can be observed in a single individual [27].

The hypothesized life position of the spine MG 4863–39 in the anterior half of the tail, considering its wide base, indicates that this animal probably had spines in all the length of its tail, more akin to *Kentrosaurus* than to *Stegosaurus*.

The dermal plates from MG 4863 are almost identical to the anterior cervical dermal plates of ML 433, sharing features such as the posterodorsal notch, being transversely thin and convex medially [22]. The dermal plates of *Hesperosaurus*, *Loricatosaurus*, *Stegosaurus*, and *Wuerhosaurus* are also transversely thin, but are not expanded transverselly, while the plates in *Hesperosaurus* are expanded at the base, but only laterally [1]. *D*. *armatus* has dorsal plates with a thicker central portion, like a modified spine [22].

### Discussion

For a comprehensive discussion of the classification of MG 4863, an updated phylogenetical analysis of Stegosauria including this specimen is fundamental–ideally, based on the most up-to-date phylogenetic analysis available (currently [17]) and coding other stegosaurs in the characters newly described herein. However, this was beyond the scope and capabilities of this study and could not be achieved at this point, so an analysis with all the taxa and characters named here will be presented in a future phylogenetical study of Stegosauria.

As in this study the focus was on the relation of *M*. *longicollum* with *D*. *armatus*, the most relevant specimens and characters for that study were summarized in the form of a character matrix (Table 4), on which the discussion herein is mostly based. In this table, coding of the most relevant species for this discussion was restricted to their holotypes (namely, *Miragaia longicollum* ML 433, *Dacentrurus armatus* NHMUK OR46013, and *Alcovasaurus longispinus* UW 20503), and their published diagnostic characters are included [9,18,22,30], as well as characters not necessarily diagnostic (some newly described herein and some previously published; e.g., [1,9]). The codification of other stegosaur species was based on possible comparisons where found (not necessarily restricted to holotypes; see text for details) or publications with widely inclusive descriptions and comparisons of Stegosauria (e.g., [1,19,18]).

**Table 4:** Distribution of anatomical characters in MG 4863 with other stegosaurs. *, Not confirmed but suggested by the morphology of the present bones. The description of some characters described previously may have been modified as to better express the features observed.

#	Anatomical character	MG 4863	ML 433	NHMUK OR46013	UW 20503	Otherstegosaur spp.
**Autapomorphies for *M*. *longicollum* according to [22]**						
**1**	Anterior tip of the premaxilla in dorsal view: concave or flat (0); drawn to a point (1)	?	1	?	?	0
**2**	Anterolateral rim of the premaxilla: does not project ventrally (0); projects ventrally (1)	?	1	?	?	0
**3**	Cervical vertebrae total: 14 or less (0); 17 or more (1)	1	1	0*	?	0
**4**	Anterior edge of base of mid-cervical neural spines: vertical and straight (0); with an anterior projection and notch (1)	1	1	0	?	0
**5**	Apices of mid and posterior cervical and anterior dorsal neural spines: not transversely expanded (0); transversely expanded (1)	1*	1	0	?	0
**6**	Cervical dermal plates: alternated, flat and without notches (0); paired, slightly inwardly convex, triangular in shape with a notch and projection on the posterodorsal margin (1)	1*	1	?	?	0
**Autapomorphy for *M*. *Longicollum* according to [17] and synapomorphy for Dacentrurinae according to [22]**						
**7**	Cervical ribs contact with para- and diapophyses of cervical vertebrae: no fusion present (0); fused from Cv3 to the third to last cervical vertebra (1)	1	1	0	?	0
**Synapomorphies for Dacentrurinae according to [22]**						
**8**	Centra of dorsal vertebrae: longer than wide (0); wider than long (1)	1	1	1	?	0
**9**	Olecranon horn on ulna: absent (0); present (1)	?	1	1	?	0
**10**	Anterior end of prepubis: not expanded dorsally (0); expanded dorsally (1)	1	?	1	?	0
**Autapomorphies for *D*. *armatus* according to [22] and [30]**						
**11**	Dorsal surface of shaft of Ischium: has a distinct angle at approximately mid-length (0); is straight (1)	?	?	1	0	0
**12**	Curvature between the anterior margin of the sacral plate and the medial margin of the preacetabular process: sharp and perpendicular (0); smoothly curved (1)	?	?	1	?	0,1
**13**	Preacetabular process of the ilium: long and thin (0); anteriorly short and broad (1)	?	?	1	?	0
**Possible diagnostic feature of *D*. *armatus* according to [30]**						
**14**	Anterior margin of the supracetabular process of the ilium: posterior to the anterior edge of the acetabulum (0); extends anteriorly beyond the anterior edge of the acetabulum (1)	1	?	1	?	0
**Autapomorphies and other diagnostic features of *A*. *longispinus* according to [18]**						
**15**	Mid and posterior caudal centra: taller than wide (0); wider than tall (1)	1	?	1	1	0
**16**	Outline of mid and posterior caudal vertebrae: round (0); sub-pentagonal (1); apple-shaped with deep excavation of the neural canal (2)	2	?	0	2	0,1
**17**	Caudal transverse processes: not present in all caudal vertebrae (0); present in all caudal vertebrae (1)	1	?	0	1	0
**18**	Lateral sides of mid and posterior caudal centra: shallowly concave often with a longitudinal ridge at mid-height (0); deeply concave with no longitudinal ridges (1)	1	?	1*	1	0
**19**	Mid and posterior caudal centra: longer than tall (0); taller than long (1)	1	?	1	1	0
**20**	Femoral condylar articular surface: present in the posterior and/or anterior surface (0); confined almost exclusively to the distal surface (1)	0	?	0	1	0
**21**	Two pairs of distal dermal spines ~90% the length of the femur, with subequal bases and slender shafts: absent (0); present (1)	?	?	?	1	0
**22**	Posterior pair of distal dermal spines: widest in the base (0); widest at ~25% the length (1)	?	?	?	1	0
**Other characters studied herein**						
**23**	Spinopostzygapophyseal laminae of cervical vertebra: absent (0), low and culminates posteriorly on the neural spine (1); culminates on the anteriormost projection on the base of the neural spine (2)	2	2	1	?	0,1
**24**	Axial position of posterior cervical neural spines: in the posterior half of the vertebra (0); at mid-length (1); in the anterior half of the vertebra (2)	2	2	1	?	0,1
**25**	Axial position of cervical neural spines passing posteriorly: become more posteriorly positioned (0); become more anteriorly positioned (1)	1	1	?	?	0
**26**	Mid and posterior cervical transverse processes axial length: less than half the length of the centrum (0); more than half the length of the centrum (1)	1	1	0	?	0
**27**	Outline in lateral view of cervical prezygapophyses: sub-round (0); straight posteriorly and round anteriorly (1); round posteriorly and straight anteriorly with an anterodorsal notch (2)	2	2	1	?	0,1
**28**	Ventral margin of cervical centra in lateral view: straight (0); markedly concave (1)	1	1	0	?	0,1
**29**	Cervical transverse processes position on the neural arch: borne on the sides of the prezygapophyses, at mid height of these (0); project ventral to or level to the ventral margin of the prezygapophyses (1)	1	1	0	?	1
**30**	Posterior margin of posterior cervical neural spines: straight and vertical (0); apex expanded posteriorly (1)	?	0	1	?	0
**31**	Ribs of first caudal vertebra: without proximodorsal process (0); with proximodorsal process not forming a canal (1); with closed proximodorsal canal (2)	2	?	1	?	0,1
**32**	Apices of anterior caudal neural spines: more expanded transversely (0); more expanded axially (1)	1	?	0	?	0,1
**33**	Anterior caudal ribs: curved in a ventrolateral orientation (0); straight and mainly laterally oriented (1)	1	?	0	?	0,1
**34**	Orientation of anterior caudal neural spines passing posteriorly: constant (0); progressively more anteriorly oriented (1); markedly more posteriorly oriented (2)	2	?	0	?	0,1
**35**	Neural spines of caudal vertebrae decrease in size passing posteriorly: constant along the caudal series (0); decrease to one fifth the height and width from Cd10 to Cd12, vestigial further posteriorly (1)	1	?	0	?	0
**36**	Neural arch of mid and posterior caudal vertebrae: half or more the height and width of the centrum (0); one third or less the height and width of the centrum (1)	1	?	0	1	0
**37**	Expanded ossification of the posterolateral rim of posteriormost caudal centra: absent (0); present (1)	1	?	?	1	0
**38**	Dorsal side of anterior and mid caudal transverse processes: smooth (0); with an anteroposteriorly oriented rugose ridge that projects anteriorly into a process (1)	1	?	0	?	0
**39**	Round or trapezoid shaped process ventral to the neural canal and concentric ridges culminating in it in the caudal articular facets: absent (0); present (1)	1	?	0	?	0
**40**	Point shaped process in the center of the caudal centrum articular facets: absent (0); present (1)	1	?	0	?	0
**41**	Ossified processes in chevron facets of caudal vertebrae: absent (0); present (1)	0	?	1	0	0,1
**42**	Proximal postpubis and obturator notch of pubis: about half as deep as the prepubis, obturator notch open (0); about equally as deep as the prepubis, obturator notch closed in transverse view (1)	1	?	0	?	0,1
**43**	Ventral margin of anterior end of prepubis: straight (0); curves dorsally (1); expanded ventrally (2)	1	?	2	?	0,1
**44**	Longitudinal cord-like ridges in the femur shaft: absent (0); present one anterolaterally that bifurcates distally and two posteriorly (1)	1	?	0	0	0
**45**	Tibia: slender shaft and distal end expanded mostly medially (0); thick shaft and distal end expanded almost equally laterally and medially (1)	1	?	0	?	0,1
**46**	Spine: slightly expanded base, strongly sculpted ventral surface and rounded cross-section (0); greatly expanded base, smooth concave ventral side and sharp anterior and posterior edges (1); reduced base, sculpted ventral surface and sharp anterior and posterior edges (2)	1	?	0	2	0,1

#### Classification of MG 4863

The specimen MG 4863 can be clearly ascribed to a stegosaur by the presence of the diagnostic osteodermic plates and spines, and the vestigial fourth trochanter of the femur [9], and since its anatomy is favorably compared to other stegosaurs, sharing other features such as dorsal vertebrae with elongated neural arches with upturned transverse processes, wide ilio-sacral block, short metacarpals and metatarsals, and long columnar forelimbs with long femora [1]. The stegosaur taxa known from the Upper Jurassic rocks of Europe are: *Dacentrurus armatus*, *Miragaia longicollum*, and *Stegosaurus* sp. In Portugal, the taxa known are *Miragaia longicollum*, *Dacentrurus* sp., and *Stegosaurus* sp.

The specimen MG 4863 shares three of the seven autapomorphies for *Miragaia longicollum* (according to [17] and [22]; Table 4: characters 4, 5, and 7). Two extra characters are not confirmed but very likely to exist in MG 4863: the cervical dermal plates are also slightly inwardly convex, with a notch and projection on the posterodorsal margin, but it is not possible to verify if these were arranged in pairs as only one cervical plate was collected; 16 cervical vertebrae are confirmed in MG 4863 but likely 17 cervical vertebrae were present. Cv14 is the most posterior cervical vertebra found, but the morphology of it and of posterior cervical vertebrae of other stegosaurs indicate that at least two more posterior cervical vertebrae were present, while a total of 17 is suggested by the presence of cervical rib purportedly from CV17 (albeit, even if the total is effectively of 16, it is certainly closer to the definition of *M*. *longicollum* than to the 13–14 of *S*. *stenops*, the next stegosaur with the most cervical vertebrae; [22]). The two remaining autapomorphies for *M*. *longicollum*, defined on the premaxilla (Table 4: characters 1 and 2) can not be verified since this bone is missing in MG 4863.

Five more new features were shared only by MG 4863 and ML 433 –characters 23 to 27 (character 23 is a revised description with new aspects of character 4; Table 4). Characters 28 and 29 occur in ML 433, MG 4863 and in other stegosaurs (such as *H*. *mjosi*), but not in NHMUK OR46013. Our studied specimen MG 4863 can be further distinguished from NHMUK OR46013 for 17 additional features: characters 16, 17, 31 to 36, and 38 to 46 (Table 4). No feature was found that occurs in MG 4863 and NHMUK OR46013 but is distinctive of ML 433.

Since MG 4863 evidences most of the autapomorphies of *Miragaia longicollum*, shares more characters with its holotype than with any other stegosaur, and is easily distinguishable from *Dacentrurus armatus*, it can therefore be safely ascribed to *Miragaia longicollum*.

#### *Miragaia* and *Dacentrurus*

The specimen MG 4863 and the holotype of *M*. *longicollum* clearly differ from *D*. *armatus* as, in total: 10 features were found that occur in ML 433 and MG 4863 and are different from NHMUK OR46013 (characters 3 to 5, 7, 23, 24 and 26 to 29); 27 characters can be used to separate MG 4863 and NHMUK OR46013; and 11 characters distinguish ML 433 from NHMUK OR46013 (in a combined total of 29 that distinguish either ML 433 or MG 4863 from NHMUK OR46013). Therefore, *M*. *longicollum* and *D*. *armatus* bear enough anatomical differences to be considered different species, not supporting the synonymization previously hypothesized [56]. Of the 17 additional characters that distinguish MG 4863 from NHMUK OR46013, four were found to be distinguishable from all other stegosaur species as well, so these can, by extension, be considered diagnostic of *M*. *longicollum* (although these can not be confirmed at this point in ML 433 since the concerned skeletal elements are unknown in the specimen), namely: characters 31, 34, 35, and 44 (Table 4). Four further characters (characters 38, 39, 40, and 42) also differentiate MG 4863 from NHMUK OR46013 and other stegosaur species, but these may be unknown in other specimens solely for the lack of their description (due to the level of detail these pertain or bad preservation), so these are possibly not robust diagnostic features of *M*. *longicollum* (at least until firsthand confirmation of their absence primarily in NHMUK OR46013).

The three autapomorphies for *D*. *armatus* provided by [9] and [30] (see Table 4: characters 11 to 13) are not comparable in MG 4863 as the concerned skeletal elements are missing. However, three other features occur only in NHMUK OR46013, not in ML 433, MG 4863, or any other stegosaur species (Table 4: characters 29, 30 and 43), so are possibly diagnostic of *D*. *armatus*.

Three of the four characters that support the clade Dacentrurinae (according to [22]) were found to occur in MG 4863 (characters 7, 8 and 10; character 9 can not be verified since the ulna is missing in MG 4863), supporting the classification of MG 4863 as Dacentrurinae and the affinity of *M*. *longicollum* with *D*. *armatus*. The anteriorly large supracetabular process of the ilium (Character 14) was found to also be shared by MG 4863 and NHMUK OR46013 but not in other species of stegosaurs, and since it occurs as well in ML 433-A and in the iliosacral blocks from MG, it can probably be considered an additional synapomorphy of Dacentrurinae rather than diagnostic of *D*. *armatus* [30]. The fusion of the cervical ribs to the vertebrae was reinterpreted by [17] as an autapomorphy for *M*. *longicollum*, noting that it only occurs in this taxon, as the two posterior cervical centra of NHMUK OR46013 evidence that the parapophyses were not fused to the ribs ([27]: Fig 2B and 2D). However, ML 433 shows that the two most posterior cervical vertebrae are exceptionally not fused to their ribs, so in fact this fusion can be present in a specimen/species, but not necessarily occur in all cervical vertebrae (most likely, therefore, not on the most posterior ones). Since this could be the case of the cervical centra of NHMUK OR46013 (although their exact position is unclear) and since only other specimens with affinity to or referred to *Dacentrurus* sp. evidence some fusion of the cervical ribs (e.g., the holotype of *O*. *lennieri*, the Barrihonda-El Humero and the Cerrito del Olmo specimens; [9,56,57]), this feature likely can be recovered as a synapomorphy for Dacentrurinae as previously proposed by [22]. The mid and posterior caudal vertebrae of MG 4863 and NHMUK OR46013 being wider than tall, taller than long and with concave sides may be also diagnostic of Dacentrurinae, but the available description and figures of the mid caudal and the two posterior caudal vertebrae of NHMUK OR46013 does not clearly confirm this (these features are diagnostic of *Alcovasaurus longispinus* according to [18]). Raven and Maidment [17] state that the olecranon horn is not a diagnostic character of Dacentrurinae, as the development of the olecranon process can be observed to be ontogenetic in *Stegosaurus* [17,19,102] (purportedly continuing to grow to form an olecranon horn). However, since the formation of an olecranon horn has not yet been observed in any stegosaur specimen besides ML 433 and NHMUK OR46013, it is here still considered unique and likely diagnostic of Dacentrurinae (at least until it is reported in other species analogous to those in dacentrurines).

#### Comparisons with other stegosaurs

**Fig 70:**
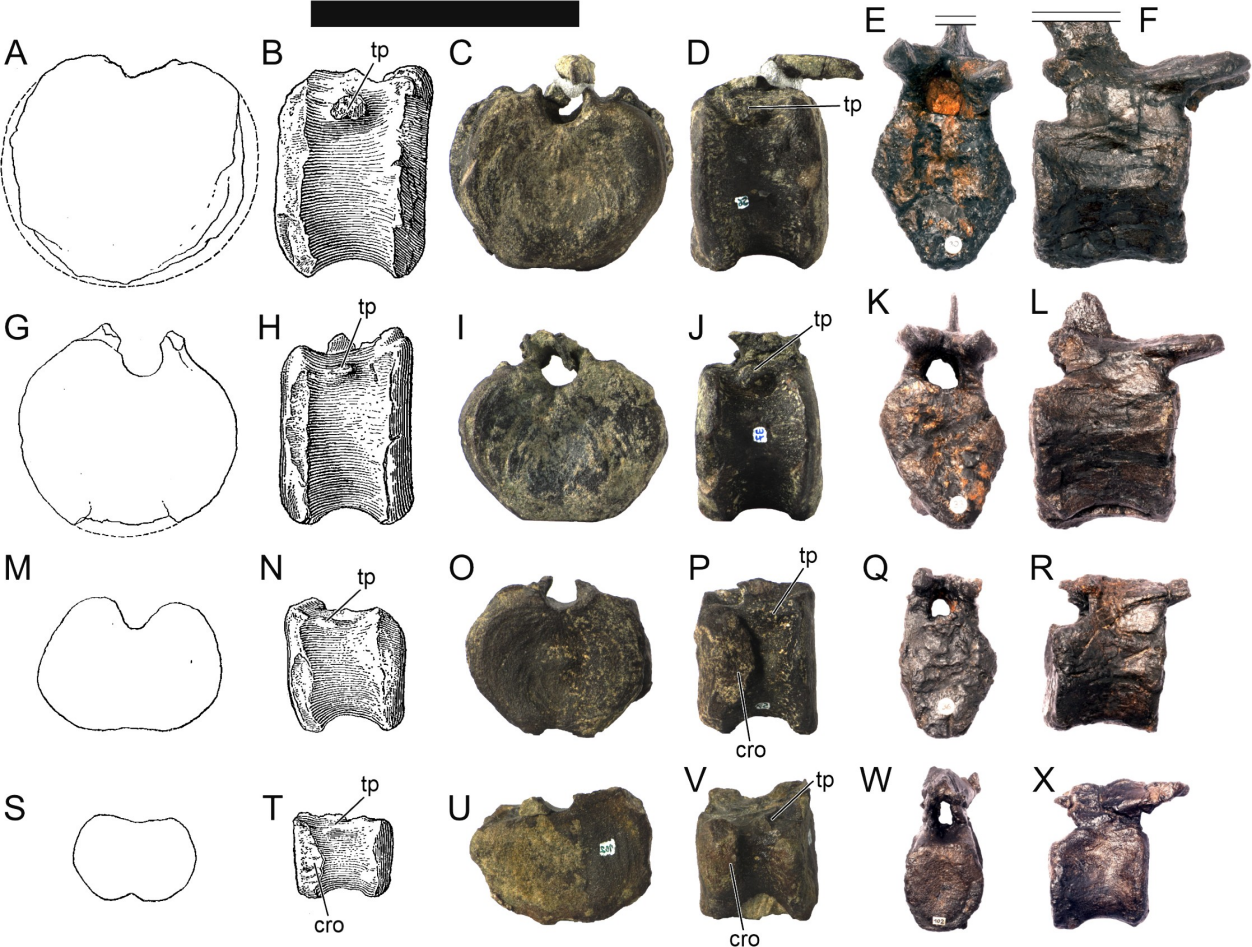
Compared posteriormost caudal vertebrae of *Miragaia longicollum* MG 4863. **A, B, G, H, M, N, S, T,** posteriormost caudal vertebrae of *A*. *longispinus* UW 20503 (modified from [33]), **C, D, I, J, O, P, U, V,** MG 4863 (mirrored for improved exhibition of the features discussed), **E, F, K, L, Q, R, W, X,** *S*. *stenops* NHMUK PV R36730 (from [19]), **C, D,** Cd30, **E, F,** Cd29, **I–L**, Cd32, **Q, R**, Cd35, **O, P**, Cd36, **U–X**, Cd37 in **A, C, E, G, I, K, M, O, P, R, S, U, W**, anterior view and **B, D, F, H, J, L, N, P, R, T, V, X**, right lateral view. The neural spines of **E** and **F** were cut for better visualization. **Cro,** lateral centrum rim ossification, **tp**, transverse process. Scale bars equal to 10 cm.

Gilmore [33] found that *A*. *longispinus* (Fig 70A–70H) could be easily distinguished from other species of *Stegosaurus* (Fig 70Q–70X) because the posterior caudal vertebrae were vertically compressed, rounded in axial view, had transverse processes until the most posterior caudal vertebrae, the sides were deeply concave, the centra were “mushroom headed” and axially shortened, so the length exceeded the height (as opposed to centra transversely compressed, pentagonal axial outline, sides moderately concave, transverse processes absent by Cd17, central rims not expanded and length exceeding the height). Galton and Carpenter [18] found that the posterior caudal vertebrae of *A*. *longispinus* UW 20503 “also differed in these characters from those of all other stegosaurs where known”, including *Hesperosaurus mjosi*, *Huayangosaurus taibaii*, *Loricatosaurus priscus*, *Chungkingosaurus jiangbeiensis* and *Tuojiangosaurus multispinus*. However, all of these diagnostic features of the posterior caudal vertebrae of *A*. *longispinus* also occur in the equivalent in MG 4863 (Fig 70I–70P). The most posterior caudal vertebra figured of UW 20503 (Fig 70A–70D; [33]: Fig 67) share even more similarities with the most posterior caudal vertebrae of MG 4863 (Cd36 and Cd37; Fig 70I–70L): a markedly more dorsoventrally compressed outline than preceding caudal vertebrae, transverse processes reduced to vestigial ridges and alike presence of lateral thickening of the margins of the posterior centrum rim. The strong similarities of the caudal vertebrae of *M*. *longicollum* MG 4863 and *A*. *longispinus* UW 20503 suggest a close affinity of these two species. NHMUK OR46013 apparently also shares three characters with UW 20503 that they do not share with other stegosaurs besides *Miragaia* (Table 4: characters 15, 18 and 19).

The character distribution with many similarities found between *Alcovasaurus longispinus* UW 20503 and MG 4863 (and some with *Dacentrurus*) that differ from *Stegosaurus* and all other stegosaurs suggests that *Alcovasaurus longispinus* is closer to *Dacentrurus* than to *Stegosaurus*, therefore can be diagnosed as Dacentrurinae, which would be the first occurrence of this taxon in North America.

Furthermore, *Alcovasaurus* seems to have a closer affinity to *Miragaia* than to *Dacentrurus*: since the presence of transverse processes in all caudal vertebrae and posterior caudal centra wider than tall and shorter than tall (which were considered diagnostic of *A*. *longispinus*; [18]) as well as reduced neural arches to one third the size of the centrum in mid and posterior caudal vertebrae occur also in MG 4863 *M*. *longicollum*, these may be diagnostic of the purposed clade *Miragaia*+*Alcovasaurus*. Other features shared by MG 4863 and *Alcovasaurus* that are distinct from *Stegosaurus* and *Loricatosaurus* (see [19,27]) are: caudal vertebrae lack horizontal lateral ridges; lack of central ventral keel (instead a shallow or deeply concave ventral margin occurs); neural canal deeply excavating a semi-circle shaped concavity in the dorsal surface of the centrum in axial view (which gives the apple-shaped outline to the centrum); ball shaped transverse processes anteroposteriorly equidistant. Comparisons of UW 20503 with ML 433 were and still are virtually not possible, as these two specimens have almost no equivalent skeletal elements (or, at least, comprehensively described overlapping elements), so this affinity was only evidenced by MG 4863.

Greatly elongated spines (~90% the length of the femora), with a narrow base and widest area at ~25% the length were also considered diagnostic features of *A*. *longispinus* by [18]. This form of spines is not currently known from either ML 433 or MG 4863, but various exceptionally elongated spines with two longitudinal ridges at opposite sides that approximate this description are known from the Lourinhã Formation, such as: ML 2043–1 is a 80 cm long spine that would be around 1.10 meters long (as long as the largest femurs known from the formation) if the distal tip and the whole base were preserved, that also has a narrow base and thickens at ~25% of its length; ML 436 is approximately the same size as ML 2043–1, but does not evidence thickening at ~25% of the length, while it has a narrow and oblique base; ML 812 and at least one pair of spines from SHN (Sociedade de História Natural, Torres Vedras, Portugal) are not as complete longitudinally as the former spines, but analogously have narrow and oblique bases, while also are articulated with a set of posteriormost caudal vertebrae, evidencing that these spines would have been part of the thagomizer at the posterior end of the tail (pers. obs.). This form of spines considered by [18] as diagnostic of *A*. *longispinus* is, therefore, not exclusive to UW 20503 or USA, but so far are only known as well from the Late Jurassic of the Lourinhã Formation, suggesting that these may actually be characteristic of dacentrurine stegosaurs or, more likely, the genus *Miragaia*. Concurrently, an undescribed tail spine recently found in the U.S.A., with strong similarities to dacentrurine spines from Europe with wide bases, also supports the presence of dacentrurine stegosaurs in America (pers. obs.; forthcoming).

A recent phylogenetical analysis using a super-matrix of Thyreophora [24] found that *A*. *longispinus* was the sister taxon to *Tuojiangosaurus*, but since only a communication of these results has been published so far, the shared characters found that support these results can not currently be compared with. Previous publications of these species, such as the matrix of the most up-to-date phylogenetical analysis available of Stegosauria (supplementary information in [17]), do not point any characters that could be unique to these species (thus, evidencing their grouping found later by [24]). The affinity found between the stegosaur *A*. *longispinus* and in particular *Tuojiangosaurus* is more likely a result of how missing data affects topology (just as it resulted in the anomalous placement of *A*. *longispinus* outside Eurypoda by [17]), so in this case it only proves that *A*. *longispinus* is a stegosaur as expected [17], while future phylogenetical analyses with additional characters and OTUs (for example, the characters described herein and MG 4863) will most likely prove a close affinity to *M*. *longicollum* and other Dacentrurines.

Raven and Maidment [17] suggest that *H*. *mjosi* was closer to *M*. *longicollum* than either was to *Stegosaurus* or *Dacentrurus*. Some features were found in this study to be shared by MG 4863 and *Hesperosaurus* and not by other stegosaurids, for example: deeply concave ventral margin of axis and other anterior cervical vertebrae in lateral view (as opposed to straight or shallowly concave); odontoid process not fully fused to centrum of the axis (in contrast to fully ankylosed to the centrum); odontoid process of the axis is positioned at mid height of the centrum (as opposed to positioned more dorsally); anteroposteriorly long cervical transverse processes (but not more than half the length of the centrum as in MG 4863 and ML 433); transverse processes present until the posterior caudal vertebrae (as opposed to absent by the anterior caudal vertebrae in other stegosaurids); plates thin transversely with expanded bases (as opposed to no expansion of the bases); anterior caudal vertebrae have large and well developed proximodorsal projections that almost form a closed canal (a state in approximation to the closed proximodorsal canals in Cd1 of MG 4863). These support that *H*. *mjosi* was closer to *M*. *longicollum* than to *S*. *stenops*, as previously stated [17]. However, most of these features (particularly those referring to the axis and anterior cervical vertebrae) are not possible to verify in NHMUK OR46013. [101] consider that the upwardly concave margin of the axis is an autapomorphy of *M*. *mjosi*, but since this was found to occur in MG 4863 as well, this diagnostic character should be reviewed and likely removed.

The four *Dacentrurus* sp. specimens from Museu Geológico (*sensu* [9]), although thoroughly described and studied previously [29,35], consist, like the holotype of *D*. *armatus*, mainly of posterior skeletons, so comparisons with the holotype of *M*. *longicollum* have so far been limited. Some features were found to be shared by *Miragaia* MG 4863 and these specimens, but not by *Dacentrurus* NHMUK OR46013, for example: the femora from Pedras Muitas (MG 4935), Atalaia (MG 4969), and Murteiras (MG 4877) have the same type of longitudinal ossified rugose cords (two posteriorly and one anterolaterally with distal bifurcation) that both femora of MG 4863 have (but which are absent in NHMUK 46013). However, some features were found to be shared between the MG stegosaurs and either MG 4863 or NHMUK OR46013, such as: the ischium of the stegosaur from Pedras Muitas has a similar iliac peduncle to MG 4863–60, but it has a straight dorsal margin similarly to NHMUK OR46013 (which is diagnostic of *D*. *armatus*); the anterior end of the pubis from Pedras Muitas (MG 4850) is expanded both dorsally and ventrally, the prepubis is much deeper than the postpubis and the obturator notch is not closed in lateral view. Although the peculiar ossified cords are suggestive of a close affinity of these stegosaurs with MG 4863, and the disparate pubes may be in part so due to intraspecific variation [27], it is unclear if these specimens can be safely assigned to either species at this point.

Some features were also found to be shared by MG 4863 and some of the stegosaurs from the Barrihonda-El Humero site (Teruel, Spain), but for the most part not by NHMUK OR46013, such as: a cervical vertebra (CPT-1330) also has an amphicoelous centrum, longer than wide and wider than tall, with a marked concave ventral margin of the centrum in lateral view, a pair of anterior tuberosities on the ventral surface and an anterior projection from the fused cervical ribs (which is analogous to, as well, ML 433); an anterior caudal vertebra (CPT-1434) has large and well developed proximodorsal projections that almost form a closed canal (like in the first caudal vertebra of MG 4863); a femur (CPT-1304) evidences similarly shaped and positioned longitudinal ossified rugose cords (two posteriorly and one anterolaterally with distal bifurcation). These newly found shared features with *Miragaia* may represent taxonomical affinities.

#### Reconstruction and paleobiology of MG 4863

Mateus *et al*. [22] oriented the cervical plates of ML 433 so their notches point anterodorsally and the convex sides of the plates face laterally. Since, however, this reconstruction was reviewed by turning each plate 180º degrees transversely, reinterpreting the notch as posterodorsal. In this reconstruction, each pair is in close contact with the zygapophyses of each cervical vertebra, with each neural spine medially to each pair, so positioned obliquely in an anteriorly pointing “V” in dorsal view, with the anterior ends closely meeting medially while the posterior ends of the previous pair overlap them laterally, with the convex sides facing medially (Jim Kirkland and Oliver Demuth, pers. comm.; pers. obs.). This close arrangement of the plates over the vertebrae supports the symmetrical arrangement of the osteoderms of *Miragaia* as previously found [22].

The large tail spine with a wide base herein described (MG 4863–39) can more plausibly be placed over the anterior section of the tail of the specimen (with its symmetrical pair, these would closely follow the dorsal half of the silhouette of the anterior tail). This spine is too large to have been placed in the posterior end of the tail and the base is too curved to have been placed over flatter surfaces, such as the scapulae, dorso or pelvis (where large spines have been placed; [1]). Considering this spine and other osteoderms known of dacentrurines and from the Lourinhã Formation, the dorsal covering of osteoderms of *Miragaia* (and to some extent other dacentrurines too) can be reconstructed as follows: transversely thin plates, convex medially with a posterodorsal notch over the neck (as shown by ML 433 and MG 4863); probably thicker and taller dorsal plates, with a thick central portion (a mid-morph between plates and spines) over the dorso (as the plate found of NHMUK OR46013 and some isolated plates in the Lourinhã Formation); large spines with a wide and curved base over the anterior tail, pointing posterolaterodorsally (as MG 4863–39, FUB-A and NHMUK OR46320); smaller spines over the posterior section of the tail (more approximate to the spine found of NHMUK OR46013; Fig 1XXI); at the end of the tail (part of the thagomizer) greatly elongated spines (up to 1 meter long) with a narrow base and sharp double edges (like the spines of *A*. *longispinus*, and evidenced from the Lourinhã Formation by the similar spines such as ML 2043–1, ML 436 and ML 812). All these osteoderms occurred in symmetrical pairs, as evidenced by ML 433, and MG 4863. No large spine with a wide and flat base, that would approximate the description of the parascapular spines known in some stegosaur species (e.g., [1,107,108]) is known in any dacentrurine or from the Lourinhã Formation, so *Miragaia* in life most likely lacked parascapular spines.

MG 4863 does not preserve any of the ulnae, but both ML 433 and NHMUK OR46013 do and include and preserve olecranon processes with extended olecranon horns. This horn, associated with the large deltoid crests of the humerus of these species, suggests a large *musculus deltoideus*, which is indicative of strong humeral flexion and abduction in dacentrurines [107,111]. This would grant these dinosaurs the ability to anchor the anterior body and rapidly and forcefully pivot around the posterior body, so that the tail could quickly swing with large lateral forces and face attacking predators [107,111]. MG 4863 reveals some unique morphology of the tail of *Miragaia* compared to other stegosaurs and dinosaurs, as the vertebrae of the anterior third of it (with large neural spines and transverse processes) likely anchored greatly enlarged muscles for abduction, while the rest of the tail (with vestigial projections and processes) probably had little soft tissue and was better suited for quick flexible movements. The tail of MG 4863 may therefore suggest a whip-like specialization of its tail swing, as the lateral forces are mainly applied on the anterior section, while the mid and posterior section can swing at increased speeds. It is consensual that stegosaurs regularly used their thagomizer spines for defense against predators [112,113], in some cases with lethal results [114]: the aforementioned large olecranon horns, whip-like adaptation of the tail and greatly enlarged thagomizer spines of some dacentrurines may be indicative of a specialized and more derived tail defense technique among members of this clade, that has not been evidenced in any other stegosaur species. This may have been an adaptation to defend against exceptionally large theropod predators (such as *Torvosaurus*, that occurred both in Europe and the USA where dacentrurine remains are found), but these likely unique biomechanical capabilities can only be properly tested by further methods of reconstruction, including CAD assisted biomechanical testing and comparisons (as tested with *Kentrosaurus*; [8,107]). A more complete reconstruction of *Miragaia* and dacentrurines in general will be addressed in a future paper.

## Conclusions

The unpublished and unprepared fossil remains of the stegosaur from Atouguia da Baleia (MG 4863) were almost fully prepared, described, identified, and cataloged in the LNEG fossil collection. The specimen is ready to be displayed in Museu Geológico de Lisboa as planned, which will be the first mounting in Portugal of an original dinosaur fossil skeleton in life position.

It was concluded by inference of the available information that the specimen MG 4863 was found in 1959 by Georges Zbyszewski one kilometer NE of the center of Atouguia da Baleia (further possible data may confirm or correct this conclusion).

The fossil skeleton of MG 4863 was successfully separated from the unprepared remains of the holotype of *Dracopelta zbyszewskii* (MG 5787) it was mixed with, allowing the studies that are currently being carried out on this ankylosaur and in the future.

The description and comparison of the specimen MG 4863 is provided here and the specimen is conclusively classified as the stegosaur *Miragaia longicollum–*and not to its purportedly closest relative, *Dacentrurus armatus–*based on a total of 29 characteristics: four of the six autapomorphies after Mateus *et al*., 2009; 10 features shared by MG 4863 and the holotype of *M*. *longicollum* (ML 433) but not by any other stegosaur species (of these, only two could not be verified to be absent in the holotype of *D*. *armatus*, NHMUK OR46013); two features shared by MG 4863, ML 433, and some other species of stegosaurs, but distinct from NHMUK OR46013; seven features occur exclusively in MG 4863, differing from NHMUK OR46013 and all other stegosaur species (but could not be verified in ML 433 due to missing comparable skeletal elements); and 10 features that, although not exclusive to MG 4863 and not verifiable currently in ML 433, were found to distinguish it from NHMUK OR46013.

As such, MG 4863 is here conclusively classified as *Miragaia longicollum* and a revised diagnosis of *Miragaia longicollum* is given here based on these results: anterior tip of the premaxilla is drawn into a point; anterolateral rim of the premaxilla projects ventrally; at least 17 cervical vertebrae; cervical vertebrae with spinopostzygapophyseal laminae and an associated lower pair of medial ridges that pass laterally on the neural spine and culminate on the anteriormost projection on the base of it; mid and posterior cervical and anterior dorsal neural spines with transversely expanded apices; paired, slightly inwardly convex, triangular cervical dermal plates with a notch and a projection on the posterodorsal margin; cervical neural spines are positioned over the anterior half of the centrum and become progressively more anteriorly positioned passing posteriorly on the cervical series; cervical transverse processes more than half the axial length of the centrum in all but the anteriormost cervical vertebrae; outline in lateral view of cervical prezygapophyses round posteriorly and straight anteriorly with an anterodorsal notch; closed proximodorsal canal on the ribs of the first caudal vertebra; progressively more posteriorly inclined neural spines of anterior caudal vertebrae, inclined at less than 45º to the horizontal between Cd8 and Cd11; neural spine reduced to one fifth the height and width from the 10th to the 12th caudal vertebra, vestigial further posteriorly in the vertebral series; presence of longitudinal cord-like ridges in the femur shaft, two posteriorly and one anterolateral with distal bifurcation.

In total, 11 features were found that distinguish ML 433 from NHMUK OR46013 and 27 that distinguish MG 4863 from NHMUK OR46013 –in a combined total of 29 that distinguish either ML 433 or MG 4863 from NHMUK OR46013 and 10 that distinguish both ML 433 and MG 4863 from NHMUK OR46013 –evidencing that MG 4863 and ML 433 should not be classified in the same species as NHMUK OR46013, which clearly refutes the hypothesis by [56] that *Miragaia longicollum* could be synonymized with *Dacentrurus armatus*, so *Miragaia longicollum* is herein considered a valid species. Excluding autapomorphies, *M*. *longicollum* can therefore be distinguished from *D*. *armatus* as well for: transverse processes present in all caudal vertebrae; posterior caudal vertebrae with apple-shaped outline; all cervical centra have markedly concave ventral margins in lateral view; cervical transverse processes positioned posteroventrally to the prezygapophyses; absence of posterior expansion of the apices of cervical neural spines; anterior caudal ribs are straight and horizontal; neural arch of mid and posterior caudal vertebra is less than one third the height and width of the centrum; presence of an anteroposteriorly oriented rugose ridge with an anterior process dorsally in mid caudal transverse processes; presence in the caudal articular facets of a process ventral to the neural canal and concentric ridges culminating in it; presence of a point shaped process in the center of caudal articular facets; absence of ossified processes in chevron facets; closed obturator notch and deep proximal postpubis; ventral margin of anterior end of the pubis curves dorsally; tibia with thick shaft and distal end equally expanded medially and laterally; caudal dermal spines with greatly expanded base, smooth concave ventral side and sharp anterior and posterior edges.

Considering how relatively complete MG 4863 and ML 433 are, and that MG 4863 can be safely ascribed to *Miragaia longicollum*, the combination of the two specimens means that the skeleton of *Miragaia longicollum* is now one of the best represented among stegosaur species (with only the dorsal and caudal osteoderms mostly unknown), comparable to *Kentrosaurus aethiopicus* and *Huayangosaurus taibaii*. MG 4863 includes, in this case, the first representative and conclusively identified skeletal remains of the tail, pelvis and hindlimbs of *Miragaia longicollum*. Among other features newly observed, it can therefore be verified that *M*. *longicollum* had pelvis and hindlimbs mostly similar to other stegosaurs (particularly *D*. *armatus*), while the tail vertebrae present many unique features among stegosaurs. The large anterior caudal spine found of MG 4863 suggests also that *Miragaia* had large spines over most or the whole tail, similarly to stegosaurs such as *Kentrosaurus*.

The only current autapomorphy for *D*. *armatus* (according to [9] and [22]) and the two additional autapomorphies proposed recently [30] could not be verified or confirmed to be absent in MG 4863 or ML 433, therefore it is unclear after this research if these can be safely considered diagnostic of *D*. *armatus*. However, three other features present in NHMUK OR46013 were found to not be shared by ML 433, MG 4863, or any other known stegosaur species. As such, a possible revised diagnosis for *D*. *armatus* can be suggested here as well: the dorsal surface of the distal ischial shaft is straight; the anterior end of the prepubis expands ventrally; apex of cervical neural spines expand posteriorly; and cervical transverse processes borne at mid height of the prezygapophyses. This study also concludes that, although two descriptions of the holotype of *D*. *armatus* have already been published (namely [12] and [27]), a new full skeleton description of the specimen is required to properly diagnose the species and attempt to provide stability to this taxon, Dacentrurinae and its members.

Four features were found to be shared by NHMUK OR46013 with either ML 433 or MG4863 or both, but not by any other stegosaur species, supporting their close taxonomical relation. However, considering the 28 features that were found that can differentiate *M*. *longicollum* from *D*. *armatus*, this is more supportive of their common classification in the clade Dacentrurinae than of the hypothesized synonymization of the genera and species. As such, these shared features can be considered diagnostic of Dacentrurinae, namely: the dorsal vertebrae with centra wider than long; presence of an olecranon horn on the ulna; dorsal expansion of the anterior end of the pubis and the supracetabular process of the ilium that extends anteriorly beyond the anterior edge of the acetabulum. The presence of fusion of the cervical ribs with the cervical vertebrae is most likely also diagnostic of Dacentrurinae (as previously suggested by [22] and removed by [17]), despite that the existing cervical vertebrae of NHMUK OR46013 can not confirm this (see Discussion).

Four characteristics were found to be shared by MG 4863 and the holotype of *Alcovasaurus longispinus* (UW 20503) but not by any other stegosaur species, while another three are purportedly shared as well by the holotype of *D*. *armatus*. Particularly, all five features that were found to differentiate the posterior caudal vertebrae of *A*. *longispinus* from those of other stegosaurs (according to [18], two of them autapomorphies of the species) also occur in MG 4863. Of the five autapomorphies for *A*. *longispinus* [18], only one (the distally confined articular surface of the femur) could be verified to differ from MG 4863 or NHMUK OR46013. These shared features show that *A*. *longispinus* has a closer relation with *Dacentrurus* than with *Stegosaurus*, which by definition means that it can be classified as a dacentrurine stegosaur, suggesting particularly a close affinity to *Miragaia longicollum*. This affinity of *A*. *longispinus* with dacentrurines resolves its problematic phylogenetical placement (e.g., outside Eurypoda; [17]), confirming its suspected classification as a stegosaur [17,18]. It can not though be concluded currently if this grouping is more strongly supported than with *Tuojiangosaurus*, as briefly reported by [24]. However, since previous publications do not indicate UW 20503 to have more shared morphologies to *Tuojiangosaurus* than those found herein with MG 4863, a closer relation of *A*. *longispinus* to *M*. *longicollum* than to *T*. *multispinus* is still considered more likely to be valid (at least until a phylogenetical analysis of Stegosauria updated with the characters found herein confirms or discredits this).

If *Alcovasaurus* is considered a dacentrurine stegosaur, the mid and posterior caudal centra wider than tall, taller than long and with deeply concave lateral sides can also be diagnostic of Dacentrurinae, since these only occur in MG 4863, UW 20503, and purportedly also in NHMUK OR46013, but not in other stegosaur species. *Alcovasaurus longispinus* is the first credible dacentrurine stegosaur known from the American continent, first indication that the clade Dacentrurinae was shared across the Upper Jurassic Proto-North Atlantic and not confined to Europe as previously shown by its known fossil record, supporting too the hypotheses of ephemeral land-bridges at this time connecting the two continents.

Since of the five autapomorphies of *A*. *longispinus* (and three additional distinctive features) found by [18], at least five also occur in *M*. *longicollum* MG 4863, three supposedly in *D*. *armatus* NHMUK OR46013, and only one was found to be distinct from both, we here suggest the hypothesis that *A*. *longispinus* may by definition be a species of one of these two genera. If so, a classification to the genus *Miragaia* is more strongly supported, as at least four characters were found to be exclusive to MG 4863 and UW 20503, while distinct from NHMUK OR46013. If *A*. *longispinus* and *M*. *longicollum* are indeed congeneric, the genus *Miragaia* takes priority as it was named before (in 2009, [22]) *Alcovasaurus* (in 2016, [18]). In this case, a possible diagnosis for the genus *Miragaia* would be: transverse processes present in all caudal vertebrae; outline of mid and posterior caudal centra is apple-shaped, with deep depression of the neural canal; neural arch of mid and posterior caudal vertebrae one third or less the heigth and width of the centrum; lateral ossification of the posterior rim of the posteriormost caudal centra. Other characters may be also considered diagnostic of *Miragaia* with further comparisons in the future—such as the presence of slender and elongate tail spines ~90% the length of the femur, if they are confirmed to occur in specimens of *M*. *longicollum*. With this, the diagnosis of *Miragaia longispinus* is: femoral condylar articular confined almost exclusively to the distal surface; posterior dermal tail spines with subequal bases; posterior dermal tail spines widest at ~25% of length. However, a full updated specimen-level phylogenetic study of Stegosauria is still required to support these taxonomical conclusions, particularly to confirm if *M*. *longicollum* indeed had a closer relation with *A*. *longispinus* than with *D*. *armatus*, thus supporting the congenericity, as well as if *A*. *longispinus* has a closer relation to dacentrurines than to *Tuojiangosaurus*.

The new features herein found to be shared solely between MG 4863 and the holotype of *Hesperosaurus mjosi* further support the hypothesis by third authors [17] that these species have possibly a closer affinity than *M*. *longicollum* has to *D*. *armatus* or *H*. *mjosi* has to *S*. *stenops*, but the relation between these three taxa could not be properly evaluated without a full numerical cladistic analysis. It is though evident that *M*. *longicollum* and *H*. *mjosi* have an even closer affinity than previously thought, possibly closer than either has to *S*. *stenops*.

Of the specimens of *Dacentrurus* sp. in MG and from Spain, a number of features were found to be shared between these and MG 4863, but not by NHMUK OR46013 (for example, the cervical vertebra CPT-1330 with fused cervical ribs and concave ventral margin, and the femora from Pedras Muitas, Murteiras and Atalaia with one anterolateral and two posterior cords), but a few other characteristics were found to be more akin to NHMUK OR46013 than to MG 4863 or ML 433 (such as a straight dorsal margin of the ischium and open obturator notch of the pubis of the stegosaur from Pedras Muitas). As such, in all compared cases it is inconclusive at this point if these specimens could be classified to either *D*. *armatus* or *M*. *longicollum*. However, it is evident that these share more features with *M*. *longicollum* than previously thought and that with a full numerical phylogenetic analysis (incorporating as well the potentially “Rosetta-stone” specimen MG 4863) they likely could be more conclusively classified to either of these species or another species of dacentrurine stegosaurs.

## Supporting information

S1 Fig 3D pdf containing a 3D model of the dentary of MG 4863. Published under a CC BY license, with permission from Marco Marzola, original copyright 2017.
(ZIP)

S2 Fig 3D pdf containing a 3D model of cervical vertebrae two to five of MG 4863. Published under a CC BY license, with permission from Marco Marzola, original copyright 2017.
(ZIP)

S3 Fig 3D pdf containing a 3D model of cervical vertebra nine of MG 4863. Published under a CC BY license, with permission from Marco Marzola, original copyright 2017.
(ZIP)

S4 Fig 3D pdf containing a 3D model of cervical vertebrae 11 to 13 of MG 4863. Published under a CC BY license, with permission from Marco Marzola, original copyright 2017.
(ZIP)

S5 Fig 3D pdf containing a 3D model of caudal vertebra one of MG 4863. Published under a CC BY license, with permission from Marco Marzola, original copyright 2017.
(ZIP)

S6 Fig 3D pdf containing a 3D model of the spine of MG 4863. Published under a CC BY license, with permission from Marco Marzola, original copyright 2017.
(ZIP)

## References

[1] Galton PM, Upchurch P. Stegosauria In: Weishampel DB, Dodson P, Osmólska H, editors. The Dinosauria. 2nd ed Berkeley: University of California Press; 2004:343–62.

[2] Marsh OC. A new order of extinct reptilia (Stegosauria) from the Jurassic of the Rocky Mountains. Am J Sci. 3^rd^ series 1877;14:34–5.

[3] Farlow JO, Hayashi S, Tattersall GJ. Internal vascularity of the dermal plates of *Stegosaurus* (Ornithischia, Thyreophora). Swiss Journal of Geosciences. 2010 9 1;103(2):173–85.

[4] Main RP, De Ricqlès A, Horner JR, Padian K. The evolution and function of thyreophoran dinosaur scutes: implications for plate function in stegosaurs. Paleobiology. 2005 4;31(2):291–314.

[5] Padian K, Horner JR. The evolution of ‘bizarre structures’ in dinosaurs: biomechanics, sexual selection, social selection or species recognition?. Journal of Zoology. 2011 1 1;283(1):3–17.

[6] Saitta ET. Evidence for sexual dimorphism in the plated dinosaur *Stegosaurus mjosi* (Ornithischia, Stegosauria) from the Morrison Formation (Upper Jurassic) of western USA. PLoS One 2015; 10 (4): e0123503 10.1371/journal.pone.0123503 . 25901727 PMC4406738

[7] McWhinney LA, Rothschild BM, Carpenter K. Posttraumatic chronic osteomyelitis in *Stegosaurus* dermal spikes. The armored dinosaurs. 2001:141–56.

[8] Mallison H. Defense capabilities of *Kentrosaurus aethiopicus* Hennig, 1915. Palaeontologia Electronica. 2011 1 1;14(2):1–25.

[9] Maidment SCR, Norman DB, Barrett PM, Upchurch P. Systematics and phylogeney of Stegosauria (Dinosauria: Ornithischia). J Syst Palaeontol. 2008; 6:364–407.

[10] Mantell GA. XIII. On the structure of the jaws and teeth of the *Iguanodon*. Philosophical Transactions of the Royal Society of London. 1848 1 1;138:183–202.

[11] Atherstone WG. Geology of Uitenhage. Eastern Province Monthly Magazine. 1857 6;1(10):518–32.

[12] Owen R. A Monograph on the Fossil Reptilia of the Mesozoic Formations: Genera *Bothriospondylus*, *Cetiosaurus*, *Omosaurus*/by Owen. Palaeontographical Society; 1875.

[13] Sereno PC. A rationale for phylogenetic definitions, with application to the higher-level taxonomy of Dinosauria [41–83]. Neues Jahrbuch für Geologie und Paläontologie-Abhandlungen. 1998 11 10:41–83.

[14] Butler RJ, Upchurch P, Norman DB. The phylogeny of the ornithischian dinosaurs. Journal of Systematic Palaeontology. 2008 1 1;6(1):1–40.

[15] Marsh OC. Principal characters of American Jurassic dinosaurs. Part IX: The skull and dermal armor of *Stegosaurus*. Am J Sci. 3^rd^ series 1887;34:413–7.

[16] Brown B, Kaisen PC. The Ankylosauridae, a new family of armored dinosaurs from the Upper Cretaceous. Bulletin of the AMNH. 1908; v. 24, article 12.

[17] Raven TJ, Maidment SCR. A new phylogeny of Stegosauria (Dinosauria, Ornithischia). Palaeontology. 2017 5;60(3):401–18.

[18] Galton PM, Carpenter K. The plated dinosaur *Stegosaurus longispinus* Gilmore, 1914 (Dinosauria: Ornithischia; Upper Jurassic, western USA), type species of *Alcovasaurus* n. gen. Neues Jahrbuch für Geologie und Paläontologie-Abhandlungen. 2016 2 5;279(2):185–208.

[19] Maidment SCR, Brassey C, Barrett PM. The postcranial skeleton of an exceptionally complete individual of the plated dinosaur *Stegosaurus stenops* (Dinosauria: Thyreophora) from the Upper Jurassic Morrison Formation of Wyoming, USA. PloS one. 2015 10 14;10(10):e0138352 10.1371/journal.pone.0138352 26466098 PMC4605687

[20] Carpenter K, Miles CA, Cloward K. New primitive stegosaur from the Morrison Formation, Wyoming. In Carpenter K, editor. The Armored Dinosaurs Bloomington: Indiana University Press; 2001:55–75.

[21] Escaso F, Ortega F, Dantas P, Malafaia E, Pimentel NL, Pereda-Suberbiola X, et al New evidence of shared dinosaur across Upper Jurassic Proto-North Atlantic: *Stegosaurus* from Portugal. Naturwissenschaften 2007; 94: 367–374. 10.1007/s00114-006-0209-8 . 17187254

[22] Mateus O, Maidment SCR, Christiansen NA. A new long-necked ‘sauropod-mimic’ stegosaur and the evolution of the plated dinosaurs. P Roy Soc Lond B Bio. 2009;276:1815–21. 10.1098/rspb.2008.1909 PMC2674496 19324778

[23] Maidment SCR. Stegosauria: a historical review of the body fossil record and phylogenetic relationships. Swiss Journal of Geosciences. 2010 9 1;103(2):199–210.

[24] Raven T. The first phylogenetical super-matrix of the armoured dinosaurs (Ornithischia, Thyreophora). 78th Annual Meeting of the Society of Vertebrate Paleontology. Albuquerque: Journal of Vertebrate Paleontology, Program and Abstracts. 2018 Oct:201–2.

[25] Lucas FA. A new generic name for *Stegosaurus marshi*. Science. 1902;16:435 10.1126/science.16.402.435 17771815

[26] Leidy JO. Notices of extinct vertebrated animals discovered by Prof. E. Emmons. Proc. Acad. Nat. Sci. Philadelphia. 1856;8:255–6.

[27] Galton PM. British plated dinosaurs (Ornithischia, Stegosauridae). J Vertebr Paleontol. 1985;5:211–54.

[28] Buckland W. XXI—Notice on the *Megalosaurus* or great Fossil Lizard of Stonesfield. Transactions of the Geological Society of London. 1824 1 1;2(2):390–6.

[29] Galton PM. Postcranial remains of the stegosaurian dinosaur *Dacentrurus* from the Upper Jurassic of France and Portugal. Geologica et Paleontologica 1991;25:299–327.

[30] Escaso F, Ortega F, Sanz JU, Malafaia E. The systematic utility of the ilio-sacral blocks of the european stegosaur *Dacentrurus armatus*. 71st Annual Meeting of the Society of Vertebrate Paleontology. Journal of Vertebrate Paleontology, Program and Abstracts. 2011:105.

[31] Owen R. Monographs on the British Fossil Reptilia of the Mesozoic Formations. Part III. *Omosaurus* (continued). Monographs of the Palaeontographical Society. 1877 2 1;31(141):95–7.

[32] Nopcsa F. *Omosaurus lennieri*, un nouveau dinosaurian du Cap de la Hève. Bulletin de la Société Géologique de Normandie 1911;30:23–42. French.

[33] Gilmore CW. Osteology of the armored Dinosauria in the United States National Museum, with special reference to the genus *Stegosaurus*. United States National Museum Bulletin 1914;89:1–143.

[34] Dong ZM, Li XM, Zhou SW, Chang YH. [Stegosaurian remain from Zigeng (Tzekung), Zsechuan Province]. Vertebrata PalAsiatica. 1977 1 1;15(4):307. Chinese.

[35] de Lapparent AF, Zbyszewski G. Les dinosauriens du Portugal. Direction Générale des Mines et Services Géologiques; 1957. French.

[36] Zbyszewski G. Les ossements d’*Omosaurus* découverts près de Baleal (Peniche). Com. Serv. Geol. de Portugal. 1946;28:135–44. French.

[37] Mateus O. Dinossauros do Jurássico Superior de Portugal, com destaque para os saurísquios [dissertation]. Universidade Nova de Lisboa; 2005. Portuguese.

[38] Owen R. Monographs on the British Fossil Reptilia from the Oolitic Formations. Part First, Containing *Scelidosaurus harrisonii* and *Pliosaurus grandis*. Monographs of the Palaeontographical Society. 1861 12 1;13(56):1–4.

[39] Galton PM. A juvenile stegosaurian dinosaur, “*Astrodon pusillus*,” from the Upper Jurassic of Portugal, with comments on Upper Jurassic and Lower Cretaceous biogeography. Journal of Vertebrate Paleontology. 1981 12 1;1(3–4):245–56.

[40] Antunes MT, Mateus O. Dinosaurs of Portugal. Comptes Rendus Palevol. 2003 1 1;2(1):77–95.

[41] Escaso F, Ortega F, Dantas P, Malafaia E, Silva B, Sanz JL. Elementos postcraneales de *Dacentrurus* (Dinosauria: Stegosauria) del Jurásico Superior de Moçafaneira (Torres Vedras, Portugal). Cantera Paleontológica. 2007:157–71. Spanish.

[42] Marsh OC. Notice of new Jurassic reptiles. Am J Sci. 3^rd^ series 1879;18:501–5.

[43] Escaso F, Ortega F, Dantas P, Malafaia E, Silva B, Sanz JL. Estudio preliminar del material de estegosaurio de Vale Pombas (Portugal): nueva evidencia de Stegosaurus en el Jurásico Superior del suroeste europeo. Libro de resumenes XXIV Jornadas de la Sociedad Espanola de Paleontologia, Colunga. 2008:107–8. Spanish.

[44] Salminen J, Dinis J, Mateus O. Preliminary Magnetostratigraphy for the Jurassic–Cretaceous Transition in Porto da Calada, Portugal. In STRATI 2013 2014 (pp. 873–877). Springer, Cham.

[45] Guillaume A, Costa F, Mateus O. Stegosaur tracks from the Upper Jurassic of Portugal: new occurrences and perspectives. Ciências da Terra. Forthcoming 2019.

[46] Mateus O, Milàn J. Ichnological evidence for giant ornithopod dinosaurs in the Upper Jurassic Lourinha Formation, Portugal. Oryctos. 2008;8:47–52.

[47] Mateus O, Milàn J. A diverse Upper Jurassic dinosaur ichnofauna from central‐west Portugal. Lethaia. 2010 6;43(2):245–57.

[48] Mateus O, Milàn J, Romano M, Whyte MA. New finds of stegosaur tracks from the Upper Jurassic Lourinhã Formation, Portugal. Acta Palaeontologica Polonica. 2011 9;56(3):651–8.

[49] Casanovas-Cladellas ML, Santafé-Llopis JV, Pereda-Suberbiola X. Nuevo material de estegosaurios en el Cretácico Inferior de Valencia (Aras de Alpuente, localidad de Losilla I). Paleontologia i Evolució. 1995(28–29):269–74. Spanish.

[50] Casanovas-Cladellas ML, Santafé-Llopis JV. Presencia, por primera vez en España, de dinosaurios estegosaurios. Revista Española de Paleontología. 1995;10(1):83–9. Spanish.

[51] Casanovas-Cladellas ML, Santafé-Llopis JV, Santisteban-Bové C. *Dacentrurus armatus* (Stegosauria, Dinosauria) del Cretácico Inferior de Los Serranos (Valencia, España). Revista española de paleontología. 1995(2):273–83. Spanish.

[52] Casanovas-Cladellas ML, Santafé-Llopis JV, Santisteban-Bove C, Pereda-Suberbiola X. Estegosaurios (Dinosauria) del Jurasico superior-Cretacico inferior de la Comarca de los Serranos (Valencia, Espana). Revista Española de Paleontología No. extr. Homenaje al Prof. J. Truyols 1999:57–63. Spanish.

[53] Cobos A, Gascó F. New vertebral remains of the stegosaurian dinosaur *Dacentrurus* from Riodeva (Teruel, Spain). Sociedade Geológica de España. Geocaceta. 2013;53:17–20.

[54] Cobos A, Royo-Torres R, Alcalá L. 2008. Presencia del estegosaurio *Dacentrurus* en Riodeva (Teruel). Libro de resúmenes. XXIV Jornadas de la Sociedad Española de Paleontología. Museo del Jurásico de Asturias (MUJA), Colunga. 2008 10:89–90. Spanish.

[55] Cobos A, Royo-Torres R, Alcalá L, Luque L, Mampel L. Stegosaurian dinosaurs from the Villar del Arzobispo Formation of Teruel (Spain). In Symposium on Stegosauria Switzerland. Abstracts of the Scientific Meeting at the Sauriermuseum Aathal 2009:2.

[56] Cobos A, Royo-Torres R, Luque L, Alcalá L, Mampel L. An Iberian stegosaurs paradise: The Villar del Arzobispo Formation (Tithonian–Berriasian) in Teruel (Spain). Palaeogeography, Palaeoclimatology, Palaeoecology. 2010 7 1;293(1–2):223–36.

[57] Company J, Pereda-Suberbiola X, Ruiz-Omeñaca JI. [New stegosaurian (Ornithischia, Thyreophora) remains from Jurassic-Cretaceous transition beds of Valencia province (Southwestern Iberian Range, Spain)]. Journal of Iberian Geology. 2010 7 1;36(2):243. Spanish.

[58] Ruiz-Omeñaca JI. Restos de dinosaurios (Saurischia, Ornithischia) del Barremiense superior (Cretácico inferior) de Castellote (Teruel) en el Muséum Nacional d’Histoire Naturelle de París. Mas de las Matas. 2000;19:39–119. Spanish.

[59] Ruiz-Omeñaca JI, Pereda-Suberbiola X, Piñuela L, García Ramos JC. First evidence of stegosaurs (Dinosauria: Thyreophora) in the Vega Formation, Kimmeridgian, Asturias, N Spain. 2013

[60] Pereda-Suberbiola X, Galton PM, Torcida F, Huerta P, Izquierdo LÁ, Montero D, et al First Stegosaurian Dinosaur remains from the Early Cretaceous of Burgos (Spain), with a review of Cretaceous stegosaurs. Estudios Geológicos. 2003;55:267–72.

[61] Pereda-Suberbiola X, Galton PM, Ruiz-Omeñaca JI, Canudo JI. Dermal spines of stegosaurian dinosaurs from the lower Cretaceous (Hauterivian-Barremian) of Galve (Teruel, Aragon, Spain). Geogaceta. 2005;38:35–8.

[62] Mateus O, Maidment SCR, Christiansen NA. A new specimen aff. Dacentrurus armatus (Dinosauria: Stegosauridae) from the Late Jurassic of Portugal. In III Congreso Latinoamericano de Paleontología de Vertebrados–Neuquén, Patagonia, Argentina. Libro de Resúmenes. 2008:157.

[63] Dong ZM. [Dinosaurs from Wuerho]. Institute of Paleontology and Paleoanthropology Memoir 1973;11:45–52. Chinese.

[64] Sereno PC. The evolution of dinosaurs. Science. 1999 6 25;284(5423):2137–47. 10.1126/science.284.5423.2137 10381873

[65] Ortega F, Malafaia E, Escaso F, Pérez-García A, Dantas P. Faunas de répteis do Jurássico Superior de Portugal. Paleolusitana. 2009;1:43–56. Portuguese.

[66] Galton PM. Partial skeleton of *Dracopelta zbyszewskii* n. gen. and n. sp., an ankylosaurian dinosaur from the Upper Jurassic of Portugal. Géobios. 1980 1 1;13(3):451–7.

[67] Pereda-Suberbiola X, Dantas P, Galton PM, Sanz JL. Autopodium of the holotype of *Dracopelta zbyszewskii* (Dinosauria, Ankylosauria) and its type horizon and locality (Upper Jurassic: Tithonian, western Portugal). Neues Jahrbuch für Geologie und Paläontologie-Abhandlungen. 2005 3 17:175–96.

[68] Costa F, Silva T, Fernandes J, Calvo R, Mateus O. Retracing the history of a stegosaurian dinosaur discovery in Portugal and the importance of record-keeping in Palaentology. Abstract book of the XV Encuentro de Jóvenes Investigadores en Paleontología/XV Encontro de Jovens Investigadores em Paleontologia, Lisboa. 2017:119–24.

[69] Dantas P, Sanz JL, Silva CM, Ortega F, Santos VF, Cachão M. *Lourinhasaurus* n. gen. Novo dinossáurio saurópode do Jurássico superior (Kimeridgíano superior-Titoniano inferior) de Portugal. Actas Do V Congresso Nacional de Geologia—Com. Inst. Geol. Mineiro. 1998;84:91–4. Portuguese.

[70] Mocho P, Royo-Torres R, Ortega F. Phylogenetic reassessment of *Lourinhasaurus alenquerensis*, a basal Macronaria (Sauropoda) from the Upper Jurassic of Portugal. Zoological Journal of the Linnean Society. 2014 3 28;170(4):875–916.

[71] Mota TSA. Os Serviços Geológicos entre 1918 e 1974: da quase morte a uma nova vida. Ph.D. thesis, Universidade Nova de Lisboa, Lisboa. 2007. Portuguese.

[72] França JC, Zbyszewski G, Almeida FM. Carta geológica de Portugal. Notícia explicativa da Folha 26-C Peniche. Serviços Geológicos de Portugal, Lisbon. 1960:1–33. Portuguese.

[73] Mateus O. Late Jurassic dinosaurs from the Morrison Formation (USA), the Lourinha and Alcobaça formations (Portugal), and the Tendaguru Beds (Tanzania): a comparison. New Mexico Museum of Natural History and Science Bulletin. 2006;36:223–31.

[74] Mateus O, Dinis J, Cunha PP. The Lourinhã Formation: the Upper Jurassic to lower most Cretaceous of the Lusitanian Basin, Portugal–landscapes where dinosaurs walked. Ciências da Terra/Earth Sciences Journal. 2017;19(1):75–97.

[75] Manuppella G. Carta Geológica de Portugal. Noticia explicativa da folha 30-A: Lourinhã. Instituto Geológico e Mineiro. Departamento de Geologia, Lisbon; 1999:1–83. Portuguese.

[76] França JC, Zbyszewski G, Almeida FM. Carta Geológica de Portugal. Notícia explicativa da Folha 30-A Lourinhã. Serviços Geológicos de Portugal, Lisbon; 1961:1–27. Portuguese.

[77] Zbyszewski G, Almeida FM, Assunção CT. Carta Geológica de Portugal. Notícia explicativa da folha 30-C Torres Vedras. Serviços Geológicos de Portugal, Lisbon; 1955:1–33. Portuguese.

[78] Brikiatis L. Late Mesozoic North Atlantic land bridges. Earth-Science Reviews. 2016 Aug 1;159:47–57.

[79] Mateus O, Walen A, Antunes MT. The large theropod fauna of the Lourinhã Formation (Portugal) and its similarity to that of the Morrison Formation, with a description of a new species of *Allosaurus*. Paleontology and Geology of the Upper Jurassic Morrison Formation: Bulletin 36. 2006;36:123.

[80] Mateus O. Late Jurassic of Morrison Formation and Portugal tetrapods compared: a model to explain faunal exchange and similarity. 76th Annual Meeting of the Society of Vertebrate Paleontology. Salt Late City: Journal of Vertebrate Paleontology, Program and Abstracts. 2016 Oct:185.

[81] Lockley MG, Garcia-Ramos JC, Pinuela L, Avanzini M. A review of vertebrate track assemblages from the Late Jurassic of Asturias, Spain with comparative notes on coeval ichnofaunas from the western USA: implications for faunal diversity in siliciclastic facies assemblages. Oryctos. 2008;8:53–70.

[82] Galton PM, Jensen JA. A new large theropod dinosaur from the Upper Jurassic of Colorado. Brigham Young University Geology Studies. 1979;26(2):1–2.

[83] Marsh OC. ART. LIII.—Notice of New Dinosaurian Reptiles from the Jurassic formation. American Journal of Science and Arts (1820–1879). 1877 12 1;14(84):514.

[84] Marsh OC. Principal characters of American Jurassic dinosaurs; Part VIII, the order Theropoda. American Journal of Science. 1884 4 1(160):329–340.

[85] Jensen JA. Three new sauropod dinosaurs from the Upper Jurassic of Colorado. The Great Basin Naturalist. 1985 10 31:697–709.

[86] Owen R. Report on British fossil reptiles. Reports of the British Association for the Advancement of Science 1842;11:60–204.

[87] Seeley HG. I. On the classification of the fossil animals commonly named Dinosauria. Proceedings of the Royal Society of London. 1888 1 1;43(258–265):165–171.

[88] Nopcsa F. Die Dinosaurier der Siebenbürgischen Landesteile Ungarns. Mitteilungen aus dem Jahrbuche der KöniglichUngarischen Geologischen Reichsanstalt 1915;23:1–26. German.

[89] Marsh OC. Principal characters of American Jurassic dinosaurs. Part III. Am J Sci. 3^rd^ series 1880;19:253–259.

[90] Lydekker R. Catalogue of the fossil Reptilia and Amphibia in the British Museum, part IV, containing the orders Anomodontia, Ecaudata, Caudata and Labyrinthodontia. British Museum of Natural History, London 1890:294.

[91] Hennig E. *Kentrurosaurus aethiopicus*; die Stegosaurierfunde vom Tendaguru, Deutsch-Ostafrika. Palaeontographica-Supplementbände. 1925 1 1:101–254. German.

[92] Olshevsky G, Ford T. The origin and evolution of the stegosaurs. Dino-Frontline. 1993;4:64–103. Japanese.

[93] Ulansky RE. [Evolution of the stegosaurs (Dinosauria; Ornithischia)]. Dinologia. 2014:1–35. Russian.

[94] Ulansky RE. [*Natronasaurus longispinus*, 100 years with another name]. Dinologia. 2014:1–10d. Russian.

[95] Raven T. A new phylogeny of Stegosauria (Dinosauria: Ornithischia). Newsletter of the Palaeontological Association. 2016;93:64–8.

[96] Romer AS. Osteology of the Reptiles. Society for the Study of Amphibians and Reptiles; 1956.

[97] Galton PM. Skull bones and endocranial casts of stegosaurian dinosaur *Kentrosaurus* Hennig, 1915 from Upper Jurassic of Tanzania, East Africa. Geologica et Palaeontologica. 1988;22:123–43.

[98] Hennig E. *Kentrosaurus aethiopicus*, der Stegosauride des Tendaguru. Sitzungsberichte der Gesellschaft naturforschender Freunde zu Berlin. 1915;1915:219–47. German.

[99] Dong ZM, Tang ZL, Zhou SW. Note on the new mid-Jurassic stegosaur from Sichuan Basin, China. Vertebrata PalAsiatica. 1982 1 1;20(1):83. Chinese.

[100] Sereno PC, Dong ZM. The skull of the basal stegosaur *Huayangosaurus taibaii* and a cladistic diagnosis of Stegosauria. Journal of Vertebrate Paleontology. 1992 9 3;12(3):318–43.

[101] Maidment SCR, Woodruff DC, Horner JR. A new specimen of the ornithischian dinosaur *Hesperosaurus mjosi* from the Upper Jurassic Morrison Formation of Montana, USA, and implications for growth and size in Morrison stegosaurs. Journal of Vertebrate Paleontology. 2018 1 2;38(1):e1406366.

[102] Galton PM. Juveniles of the stegosaurian dinosaur *Stegosaurus* from the Upper Jurassic of North America. Journal of Vertebrate Paleontology. 1982 5 1;2(1):47–62.

[103] Galton PM. The postcranial anatomy of stegosaurian dinosaur *Kentrosaurus* from the Upper Jurassic of Tanzania, East Africa. Geologica et Palaeontologica. 1982;15:139–60.

[104] Dong Z, Zhou S, Zhang Y. [Dinosaurs from the Jurassic of Sichuan]. Palaeontologica Sinica. 1983;162(C23):1–51. Chinese.

[105] Hennig E. Ein Dentale von *Kentrurosaurus aethiopicus* Hennig. Palaeontographica-Supplementbände. Schweizerbart’sche Verlagsbuchhandlung; 1936;309–12. German.

[106] Ostrom JA, McIntosh JS. Marsh's Dinosaurs. The Collections from Como Bluff. 2nd ed New Haven: Yale University Press; 1999.

[107] Mallison H. CAD assessment of the posture and range of motion of *Kentrosaurus aethiopicus* Hennig 1915. Swiss J Geosci. 2010;103:211–33

[108] Ouyang H. [Discovery of *Gigantspinosaurus sichuanensis* and its scapular spine orientation]. In Abstracts and summaries for youth academic symposium on new discoveries and ideas in stratigraphic paleontology 1992 12;47(9). Chinese.

[109] Maidment SCR, Wei GB. A review of Late Jurassic stegosaurs from the People’s Republic of China. Geol Mag. 2006;143:621–34.

[110] Galton PM. Notes on plated dinosaurs (Ornithischia: Stegosauria), mostly on dermal armor from Middle and Upper Jurassic of England (also France, Iberia), with a revised diagnosis for Loricatosaurus priscus (Callovian, England). Neues Jahrb für Geol und Paläontologie-Abhandlungen. E. Schweizerbart’sche Verlagsbuchhandlung; 2016;282(1):1–25.

[111] Bakker RT. The Dinosaur Heresies: New theories unlocking the mystery of the dinosaurs and their extinction (481 pp.). New York: William Morrow 1986.

[112] Carpenter K, Sanders F, McWhinney LA, Wood L. Evidence of predator-prey relationships: examples for *Allosaurus* and *Stegosaurus*. In: Carpenter K, editor. The Carnivorous Dinosaurs Bloomington: Indiana University Press; 2005:325–50.

[113] McWhinney LA, Rothschild BM, Carpenter K. Posttraumatic chronic osteomyelitis in *Stegosaurus* dermal spikes. In: Carpenter K, editor. The Armored Dinosaurs Bloomington: Indiana University Press; 2001:141–156.

[114] Bakker RT, Zoehfeld KW, Mossbrucker MT. Stegosaurian Martial Arts: A Jurassic Carnivore Stabbed by a Tail Spike, Evidence for Dynamic Interactions between a Live Herbivore and a Live Predator. In Gelogical Society of America (GSA) Annual Meeting, 2014 Oct 21;221.

