# Supplementary material for: Dacentrurine stegosaurs (Dinosauria): A new specimen of Miragaia longicollum from the Late Jurassic of Portugal resolves taxonomical validity and shows the occurrence of the clade in North America
Source: PLoS One. 2019 Nov 13;14(11):e0224263. doi: 10.1371/journal.pone.0224263 (PMC6853308; doi:10.1371/journal.pone.0224263)
Supplement: S2 Fig — Published under a CC BY license, with permission from Marco Marzola, original copyright 2017. (ZIP) [file S2 Fig.pdf › S2 Fig.pdf]

5-8 - axis + cervicals 3, 4, 5
